# Aluminacyclopentanes in the synthesis of 3-substituted phospholanes and α,ω-bisphospholanes

**DOI:** 10.3762/bjoc.12.43

**Published:** 2016-03-02

**Authors:** Vladimir A D’yakonov, Alevtina L Makhamatkhanova, Rina A Agliullina, Leisan K Dilmukhametova, Tat’yana V Tyumkina, Usein M Dzhemilev

**Affiliations:** 1 Institute of Petrochemistry and Catalysis of Russian Academy of Sciences, Prospekt Oktyabrya, Ufa 450075, Russian Federation

**Keywords:** aluminacyclopentanes, Dzhemilev reaction, molybdenum complexes, phospholanes, zirconium complexes

## Abstract

An efficient one-pot process for the synthesis of 3-substituted phospholanes and α,ω-bisphospholanes was developed. The method involves the replacement of aluminium in aluminacyclopentanes, prepared in situ by catalytic cycloalumination of α-olefins and α,ω-diolefins, by phosphorus atoms on treatment with dichlorophosphines (R′PCl_2_). Hydrogen peroxide oxidation and treatment with S_8_ of the synthesized phospholanes and α,ω-bisphospholanes afforded the corresponding 3-alkyl(aryl)-1-alkyl(phenyl)phospholane 1-oxides, 3-alkyl(aryl)-1-alkyl(phenyl)phospholane 1-sulfides, bisphospholane 1,1'-dioxides, and bisphospholane 1,1'-disulfides in nearly quantitative yields. The complexes LMo(CO)_5_ (L = 3-hexyl-1-phenylphospholane, 3-benzyl-1-methylphospholane, 1,2-bis(1-phenylphospholan-3-yl)ethane, and 1,6-bis(1-phenylphospholan-3-yl)hexane were prepared by the reaction of 3-substituted phospholanes and α,ω-bisphospholanes with molybdenum hexacarbonyl. The structure of the complexes was proved by multinuclear ^1^H, ^13^C, and ^31^P spectroscopy.

## Introduction

A widely used approach for the synthesis of cyclic organophosphorus compounds (OPC) is the direct transformation of five-membered metallacarbocycles based on transition metals to phosphacarbocycles on treatment with phosphorus dihalides. For example, a method for the direct conversion of zirconacyclopentenes [1–4] and zirconacyclopentadienes [5] to substituted phospholenes [6] and phospholes [7] has been reported. This approach was used to obtain materials for light-emitting diodes (LEDs) [8], phosphorus-containing polymers [9–10], bisphospholes [11], bicyclodiphospholanes and spirobicyclodiphospholanes [12–13]. However, this method is faced with some practical complications related to the synthesis of the initial zirconacarbocycles: the reactions proceed at low temperatures (−78 °С) consuming stoichiometric amounts of expensive Cp_2_ZrCl_2_. In our opinion, the synthesis of cyclic OPC via catalytic cyclometallation reactions that we developed previously [14–17] would be free from the above-indicated drawbacks.

Furthermore, analysis of world literature demonstrated that the data on direct transformation of aluminacarbocycles [18] to cyclic OPCs are scarce, except for paper [19] in which this approach is implemented for simple olefins. Meanwhile, in our opinion, the development of these reactions would give rise to practically promising one-pot methods for the preparation of a broad range of cyclic and acyclic organophosphorus compounds of specified structure (phospholanes, phospholenes, phospholes and 1,2- and 1,4-diphosphorus compounds) that were difficult to synthesize or unknown before. In order to fill this gap and to extend the scope of applicability of the catalytic cycloalumination of unsaturated compounds, we continued studies along this line, which form the subject of this paper.

**Scheme 1:**
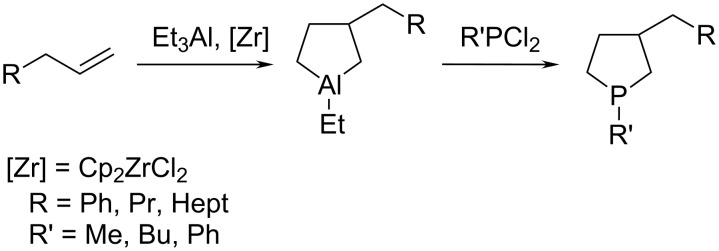
Synthesis of 3-substituted phospholanes according to earlier data [14–17].

Previously [19], it was shown for allylbenzene, hex-1-ene, and oct-1-ene that aluminacyclopentanes, prepared by the reaction of these alkenes with Et_3_Al in the presence of 5 mol % Cp_2_ZrCl_2_ (20 °С, 6–8 h), react in situ with RPCl_2_ (R = Me, Ph) in toluene for 30 min with replacement of the Al atom by a P atom to give the respective phospholanes in 79–84% yields (Scheme 1).

The present paper reports the results of experimental studies that further develop the earlier investigation about the substitution of phosphorus atoms for the Al atoms in aluminacyclopentanes with the goal to develop a preparative method for the synthesis of five-membered cyclic organophosphorus compounds.

## Results and Discussion

**Scheme 2:**
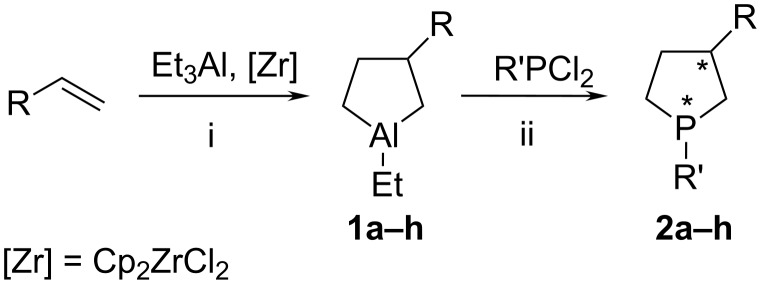
Synthesis of 3-substituted phospholanes.

First, we studied the effect of the structure of substituent in position 3 of the initial aluminacyclopentanes on the yield of target phospholanes. Under the selected conditions (toluene, 20–22 °С, 30 min), 3-alkyl-substituted aluminacyclopentanes **1a–c** react with phenyldichlorophosphine to give 3-butyl-1-phenylphospholane (**2a**), 3-hexyl-1-phenylphospholane (**2b**), or 3-octyl-1-phenylphospholane (**2c**) in ca. 92, 91, and 87% yields, respectively (Scheme 2, Table 1). The isolated organophosphorus compounds **2a–с** are 3:2 mixtures of diastereomers formed due to the presence of two asymmetric centres in the molecule at С-3 and P-1. The latter exists due to the high barrier for inversion of configuration at the phosphorus atom [20].

**Table 1:** Results of 3-substituted phospholanes ^a^.

Entry	R	R'	Product	Yield (%)	*trans*/*cis*

1	Bu	Ph	**2a^b^**	92	3/2
2	Hex	Ph	**2b**	91	3/2
3	Oct	Ph	**2c**	87	3/2
4	cyclohexyl	Ph	**2d**	93	3/2
5	cyclohexen-3-yl	Ph	**2e**	90	3/2
6	Bn	Ph	**2f^b^**	82	3/2
7	Bn	Me	**2g^b^**	84	2/1
8	Bn	Bu	**2h**	86	2/1

^a^Reaction conditions: (i) 10 mmol Et_3_Al, 5 mol % Cp_2_ZrCl_2_, toluene (25 mL), room temperature, 12 hours; (ii) 10 mmol R’PCl_2_, −5 °C to room temperature, 30 minutes. ^b^The cyclic OPCs have been previously synthesized and described in [19].

The diastereomeric mixture was identified by multinuclear ^1^H, ^13^C, and ^31^P NMR spectroscopy and conventionally designated as the *syn* and *anti* isomers in which, for example, the high-priority substituents at Р-1 and С-3 are proximate in the *syn* isomer. Using the homo- and heteronuclear 2D NMR spectroscopy (H,H-COSY, HSQC, HMBC), parameters of the NMR spectra of some compounds **2** were determined and proved to agree with published data [19]. Specifically, 1-phenyl-3-substituted phospholanes typically show ^31^Р NMR signals in area of −14 to −13 ppm, except for the 1-alkyl-3-benzylphospholane **2g** and **2h** isomers, for which the signals occur at δ_P_ = −34 ppm and −35 ppm, and −22 to −23 ppm, respectively. The presence of a magnetically active phosphorus atom in the five-membered heterocycle induces the doublet splitting of the carbon signals in the ^13^С NMR spectra of **2a–h** for both the P-substituent and the ring. The highest heteronuclear constants *J*(^31^P^13^C) for the ring, ^1^*J*_PC_ ≈ 10–20 Hz, are found for the α-carbon atoms (С-2 and С-5) of phospholanes **2a–h**.

Allylbenzene reacts with AlEt_3_ (5 mol % Cp_2_ZrCl_2_, 20 °С, 12 h) to give 3-benzyl-1-ethylaluminacyclopentane **1f**, which then reacts with phenyldichlorophosphine giving rise to 3-benzyl-1-phenylphospholane **2f** in a yield of 82% (Scheme 2).

When PhPCl_2_ is replaced by MePCl_2_ or BuPCl_2_, the reaction with 3-benzyl-1-ethylaluminacyclopentane **1f** affords the corresponding phospholanes **2g**,**h** with one of the isomers predominating (Table 1).

In the case of 3-cyclohexyl- and 3-(сyclohex-3-en-1-yl)-1-phenylphospholanes (**2d** and **2e**), the number of stereoisomers increases owing to the additional asymmetric centre С(1′) in the substituent, and the total yield of these compounds is 90–93%.

**Scheme 3:**
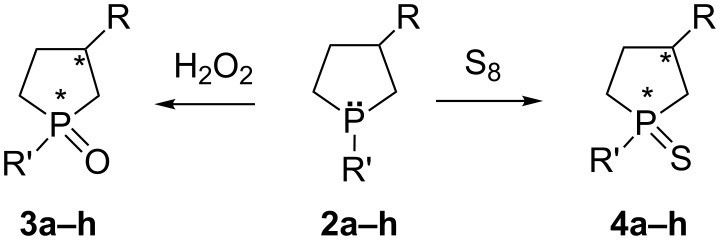
Synthesis of 3-substituted phospholane oxides and sulfides.

Phospholanes **2а–h** readily react with H_2_O_2_ in chloroform, owing to the presence of a lone electron pair at phosphorus to give phospholane 1-oxides **3а–h** in quantitative yields. The reaction of **2а–h** with S_8_ affords phospholane 1-sulfides **4а–h** also in quantitative yields (Scheme 3).

In the ^31^Р NMR spectra of phospholane oxides **3a–h** and phospholane sulfides **4a–h**, the phosphorus signal shifts downfield to ca. 57–70 ppm relative to the initial phospholanes, and the heteronuclear constants ^1^*J*(^31^P^13^C2) and ^1^*J*(^31^P^13^C5) observed in the ^13^С NMR spectra increase to ca. 53–66 Hz.

**Scheme 4:**
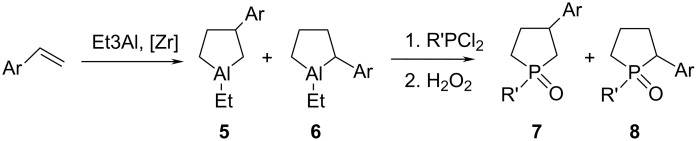
Synthesis of 3-substituted **7a–f** and 2-substituted **8a–f** phospholanes.

When styrene or 2-vinylnaphthalene reacts with AlEt_3_ in the presence of Cp_2_ZrCl_2_, apart from 3-phenyl(naphthyl)-1-ethylaluminacyclopentanes **5a–f**, the reaction mixture contains 2-phenyl(naphthyl)-1-ethylaluminacyclopentane **6a–f** [21]. Both regioisomers react in situ with phosphorus dihalides and hydrogen peroxide to afford 1-phenyl(alkyl)-2-arylphospholane oxides **7a–f** and 1-phenyl(alkyl)-3-arylphospholane oxides **8a–f** in 2:1 ratio in a 69–87% total yield (Table 2). The regioisomers were isolated by column chromatography (hexane/ethyl acetate/methanol = 5:3:1) and characterized in a separate fraction (Scheme 4).

**Table 2:** Results of 3-substituted and 2-substituted phospholanes.

Entry	Ar	R'	Product	Ratio	Yield (%)

1	Ph	Me	**7a, 8a**	2/1	84
2	Ph	Bu	**7b, 8b**	2/1	87
3	Ph	*t*-Bu	**7c, 8c**	2/1	81
4	Ph	Ph	**7d, 8d**	2/1	75
5	Naphtyl	Me	**7e, 8e**	2/1	72
6	Naphtyl	Ph	**7f, 8f**	2/1	69

It should be noted that the phosphorus signals of major intensity of 2-aryl phospholane oxides **8a**, **8b**, **8d**, **8e**, **8f**, corresponding to one of the stereoisomers, are shifted upfield by ca. 5–7 ppm with respect to those of 3-aryl-substituted phospholane oxides **7**. In contrast, the ^31^P NMR signal of the second isomer of **8** is shifted to lower field.

**Scheme 5:**
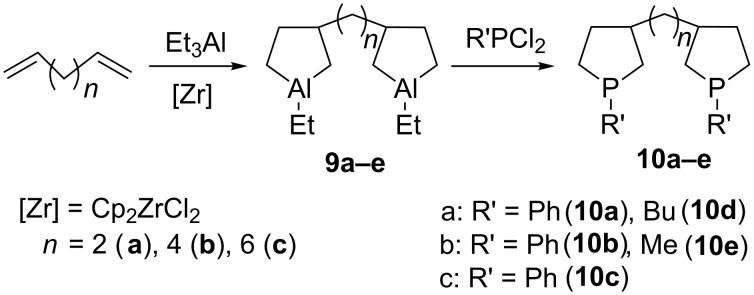
Synthesis of bisphospholanes.

For further development of this study, it appeared pertinent to apply our method in the preparation of α,ω-bisphospholane compounds by the reaction of phosphorus dihalides with bisaluminacyclopentanes (Scheme 5).

Thus bisaluminacyclopentane **9a**, synthesized by catalytic cycloalumination of 1,7-octadiene, was subjected without isolation to the substitution reaction with aryl(alkyl)dichlorophosphine with replacement of Al by Р (ca. 20 °С, 30 min) to give bisphospholane **10a** as a mixture of isomers in a total yield of 84–85%.

Under the selected conditions, bisaluminacyclopentanes **9b–e**, prepared by catalytic cycloalumination of 1,5-hexadiene and 1,9-decadiene, react with phenyl(methyl, butyl)dichlorophosphine to give 1,2-bis(1-phenylphospholan-3-yl)ethane (**10b**), 1,2-bis(1-butylphospholan-3-yl)ethane (**10d**), 1,4-bis(1-methylphospholan-3-yl)butane (**10e**), and 1,6-bis(1-phenylphospholan-3-yl)hexane (**10с**) in 85 % yields.

**Scheme 6:**
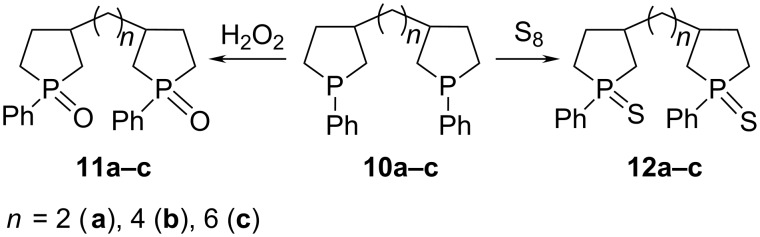
Synthesis of bisphospholane-1,1'-oxides and bisphospholane-1,1'-sulfides.

Similarly to phospholanes **2a–h,** the resulting bisphospholanes **10a–c** readily react with H_2_O_2_ in chloroform or with elemental sulfur to furnish the corresponding bisphospholane 1,1'-dioxides **11a–c** and bisphospholane 1,1'-disulfides **12a–c** (Scheme 6).

Diastereomeric cleavage of signals in the ^13^С NMR spectra of bisphospholanes observed only for compounds **10а**, **10d**, **11а**, **12а** in which the phospholane moieties are linked by two methylene groups, obviously as a result of mutual influence of the proximate asymmetric centres at C-3 and С-3′. Ratios of stereoisomers were determined by HPLC method as 2:1 (see Supporting Information File 1, Figure S1).

**Scheme 7:**
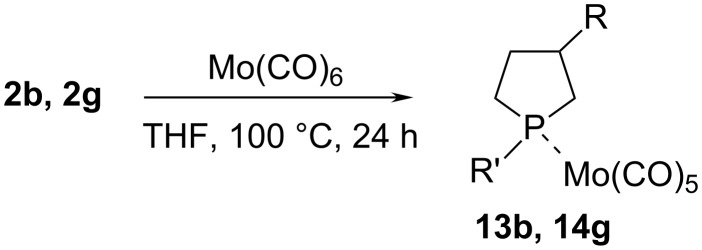
Synthesis of the molybdenum complex (3-hexyl(benzyl)-1-phenyl(methyl)phospholane)Mo(CO)_5_.

Organophosphorus compounds, including cyclic ones, are known to readily form complexes with transition metals, which are extensively studied and used in homogeneous catalytic reactions. Therefore, in order to study the properties of the cyclic OPC that we prepared and to demonstrate how they could be used as ligands, we investigated the reaction of 3-substituted phospholanes and bisphospholanes with Mo(CO)_6_, resulting in the preparation of molybdenum complexes. For example, 3-hexyl-1-phenylphospholane and 3-benzyl-1-methylphospholane react with Mo(CO)_6_ furnishing molybdenum complexes **13b** and **14g** (Scheme 7).

**Scheme 8:**
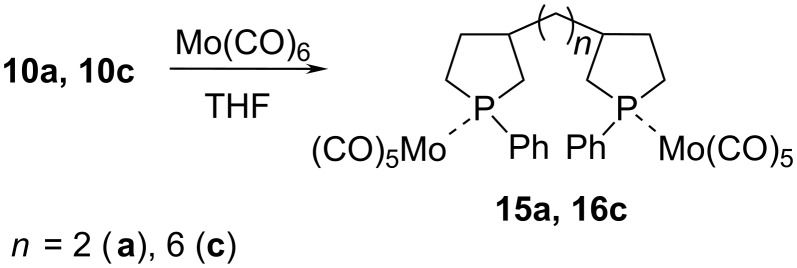
Synthesis of molybdenum complexes (1,2(1,6)-bis(1-phenylphospholan-3-yl)ethane(hexane))Mo(CO)_5_.

The complex formation is evidenced by changes in the NMR spectral parameters of compounds **13b** and **14g** in relation to the initial phospholanes. A typical feature is the downfield shift of the ^31^Р NMR signals. As a result, both compounds were found to exhibit close chemical shifts δ_P_ ≈ 25 ppm and δ_P_ ≈ 26 ppm for the diastereomers formed, irrespective of the nature of substituent R', which is indicative of equal pyramidality of the bonds of the phosphorus atom incorporated in the molybdenum complex. In addition, the С-5 signal in the ^13^С NMR spectra is shifted downfield by ca. 8 ppm and the constant *J*_PC5_ = 24.1 Hz (for example, for **13b**) is much higher not only compared to the corresponding atom in the initial phospholane **2b** but also than *J*_PC2_ = 3.0 Hz in compound **13b**. In view of the fact that the phosphorus–carbon constants obey the Karplus equation [22], this result suggests that the conformation of the five-membered heterocycle changes upon the formation of the complex with molybdenum hexacarbonyl. Similarly, bisphospholanes **10a** and **10c** react with Mo(CO)_6_ to form molybdenum complexes **15a** and **16c** (Scheme 8). The resulting molybdenum complexes are oily liquids. The structure of the complexes was also proved by spectral data.

## Conclusion

Thus, as a continuation of investigations dealing with the search for effective methods for the synthesis of five-membered cyclic organophosphorus compounds, we developed a preparative process for the one-pot conversion of aluminacyclopentanes, obtained in situ by catalytic cycloalumination of olefins or diolefins with AlEt_3_, to phospholanes, phospholane oxides or phospholane sulfides, including bisphospholanes and their derivatives in high yields.

The developed methods are distinguished by easy implementation of the reaction with the use of accessible reagents and monomers, which makes them quite promising for the use in both the laboratory practice and industry.

## Experimental

### Preparation of 3-alkyl(aryl)phospholanes (general procedure)

A glass reactor maintained under dry argon at 0 °C was successively charged, with stirring, with toluene (25 mL), Cp_2_ZrCl_2_ (0.298 g, 1 mmol), olefin (10 mmol), and AlEt_3_ (1.8 mL, 10 mmol) The mixture was warmed up to room temperature (ca. 20 °C) and stirred for 12 h. Then the reaction mixture was cooled down to −5 to −10 °С, alkyl(phenyl)dichlorophosphine (10 mmol) was added dropwise, and the mixture was stirred at room temperature for additional 30 min. Then the reaction mixture was treated with a saturated aqueous solution of NH_4_Cl and the reaction products were extracted with diethyl ether and dried with MgSO_4_. The solvent was evaporated and the target phospholanes were isolated by vacuum distillation. All operations were carried out in an argon flow.

The general procedure and analytical data for compounds **2a**, **2b**, **2f**. **2g** and **3a**, **3b**, **3f**, **3g** were previously described in [19].

3-Octyl-1-phenylphospholane (**2c**) in the mixture in a ratio of 3:2: Colorless oil (87%); bp 215–218 °C (9 torr); calcd for C_18_H_29_P: C, 78.22%; H, 10.58%; found: C, 78.3%; H, 10.6%; ^1^H NMR (400.13 MHz, CDCl_3_) δ 0.95 (t, ^3^*J* = 6.8 Hz, 6H, C(8')H), 1.27–1.63 (m, 32H, C(1')H, C(2')H, C(3')H, C(4')H, C(5')H, C(6′)H, C(7′))H, C(4)H_a_, C(2)H_a_), 1.94–2.03 (m, 4H, C(3)H, C(5)H_a_), 2.10–2.20 (m, 4H, C(2)H_b_, C(4)H_b_), 2.26 (m, 2H, C(5)H_b_), 7.24–7.30, 7.32–7.38, 7.42–7.48 (m, 10H, Ph); ^13^C NMR (100.62 MHz, CDCl_3_) δ 14.15 (C(8')), 22.70 (C(7')), 25.79 (*J*_PC_ = 12.1 Hz, C(5)), 26.60 (*J*_PC_ = 10.1 Hz, C(5)), 28.49, 28.61 (C(2')), 29.04, 29.05 (C(4')), 29.32 (C(5')), 29.57, 29.64 (C(3')), 31.63 (C(6')), 32.92 (*J*_PC_ = 13.1 Hz, C(2)), 33.16 (*J*_PC_ = 11.1 Hz, C(2)), 33.95 (*J*_PC_ = 3.0 Hz, C(4)), 34.24 (*J*_PC_ = 4.0 Hz, C(4)), 35.60 (*J*_PC_ = 3.0 Hz, C(1')), 35.86 (*J*_PC_ = 5.0 Hz, C(1')), 41.82 (*J*_PC_ = 4.0 Hz, C(3)), 43.02 (*J*_PC_ = 1.0 Hz, C(3)), 126.87 (C(9)), 127.90, 127.91 (*J*_PC_ = 5.0 Hz, C(8), C(10)), 130.07 (*J*_PC_ = 16.1 Hz, C(7), C(11)), 130.13 (*J*_PC_ = 15.1 Hz, C(7), C(11)), 142.47, 142.84 (*J*_PC_ = 23.1 Hz, C(6)) (P-Ph); ^31^P NMR (161.97 MHz, CDCl_3_) δ −13.5, −13.9; MALDI–TOF: *m*/*z* сalcd for C_18_H_30_P ([M + H]^+^): 277.4046; found: 277.4.

### Preparation of 3-alkyl(aryl)phospholane-1-oxides (general procedure)

A 30% solution of hydrogen peroxide (0.7 mL, 6 mmol) was slowly added dropwise with vigorous stirring to a solution of 3-alkyl(benzyl)-1-alkyl(phenyl)phospholane (5 mmol), synthesized as described above, in chloroform (10 mL) and the mixture was stirred for 1 h. Then the reaction mixture was washed with water (3 × 5 mL) and the organic layer was dried with MgSO_4_. The solvent was evaporated and the residue was chromatographed on silica gel (hexane/ethyl acetate/methanol 5:3:1). 3-Octyl-1-phenylphospholane-1-oxide (**3c**) in the mixture in a ratio of 3:2: Calcd for C_18_H_29_OP: C, 73.94; H, 10.00%; found: C, 73.7%; H, 9.8%; ^1^H NMR (400.13 MHz, CDCl_3_) δ 0.81 (t, ^3^*J* = 7.2 Hz, 6H, C(8')H), 1.17–1.60 (m, 30H, C(2)H_a_, C(4)H_a_, C(1')H, C(2')H, C(3')H, C(4')H, C(5')H, C(6')H, C(7')H), 1.70 (m, 1H, C(2)H_a_), 1.80 (m, 1H, C(4)H_a_), 1.87 (m, 1H, C(5)H_a_), 1.94–2.12 (m, 2H, C(3)H, C(5)H_a_), 2.14–2.35 (m, 7H, C(2)H_b_, C(3)H, C(4)H_b_, C(5)H_b_), 7.36–7.56, 7.60–7.78 (m, 10H, Ph); ^13^C NMR (100.62 MHz, CDCl_3_) δ 13.82 (C(8')), 22.35 (C(7')), 27.51, 27.60 (C(2')), 28.94, 28.96 (C(4')), 29.19 (*J*_PC_ = 66.4 Hz, C(5)), 29.21 (C(5')), 29.32, 29.36 (C(3')), 30.18 (*J*_PC_ = 66.4 Hz, C(5)), 30.99 (*J*_PC_ = 6.0 Hz, C(4)), 31.55 (C(6')), 31.93 (*J*_PC_ = 7.0 Hz, C(4)), 35.87 (*J*_PC_ = 55.3 Hz, C(2)), 35.99 (*J*_PC_ = 12.1 Hz, C(1')), 36.04 (*J*_PC_ = 14.1 Hz, C(1')), 36.16 (*J*_PC_ = 53.3 Hz, C(2)), 38.60, 40.21 (*J*_PC_ = 8.0 Hz, C(3)), 128.39 (*J*_PC_ = 12.1 Hz, C(8), C(10)), 129.55 (*J*_PC_ = 10.1 Hz, C(7), C(11)), 131.40 (*J*_PC_ = 3.0 Hz, C(9)), 133.90 (*J*_PC_ = 89.5 Hz, C(6)), 134.00 (*J*_PC_ = 90.5 Hz, C(6)) (P-Ph); ^31^P NMR (161.97 MHz, CDCl_3_) δ 59.7, 59.4; MALDI TOF: *m*/*z* calculated for C_18_H_30_OP ([M+ H]^+^): 293.4040; found: 293.5.

### General procedure for the preparation of 3-alkyl(aryl)phospholane-1-sulfides

Reactions were performed under argon. Sulfur (0.13 g, 4 mmol) was added with cooling to a solution of 3-alkyl(aryl)phospholane (4 mmol) (prepared as described above) in 10 mL toluene, and the mixture was stirred for 4 h. After filtration through a thin layer of silica gel the solvent was evaporated to give a colorless oil. 3-Hexyl-1-phenylphospholane-1-sulfide (**4b**) in the mixture in a ratio of 3:2: Calculated for C_16_H_25_PS: C, 68.53%; H, 8.99%; found: C, 68.4%; H, 8.0%; ^1^H NMR (400.13 MHz, CDCl_3_) δ 0.91 (t, ^3^*J* = 6.8 Hz, 6H, C(6')H), 1.25–1.43 (m, 16H, C(2')H, C(3')H, C(4')H, C(5')H), 1.43–1.61 (m, 5H, C(1')H, C(4)H_a_), 1.83–1.95 (m, 2H, C(2)H_a_, C(4)H_а_), 1.99 (m, 1H, C(2)H_a_), 2.11–2.52 (m, 8H, C(2)H_b_, C(3)H, C(4)H_b_, C(5)H_a_, C(5)H_b_), 2.60 (m, 1H, C(5)H_b_), 2.66 (m, 1H, C(2)H_b_), 7.42–7.53, 7.82–7.94 (m, 10H, Ph); ^13^C NMR (100.62 MHz, CDCl_3_) δ 13.77 (C(6')), 22.27, 22.29 (C(5')), 27.62, 27.68 (C(2')), 29.00 (C(3')), 31.41, 31.43 (C(4')), 31.86 (*J*_PC_ = 6.0 Hz, C(4)), 33.62 (*J*_PC_ = 4.0 Hz, C(4)), 35.44 (*J*_PC_ = 14.1 Hz, C(1')), 35.64 (*J*_PC_ = 53.3 Hz, C(5)), 35.71 (*J*_PC_ = 12.1 Hz, C(1')), 36.49 (*J*_PC_ = 53.3 Hz, C(5)), 39.74 (*J*_PC_ = 8.0 Hz, C(3)), 41.78 (*J*_PC_ = 6.0 Hz, C(3)), 41.94 (*J*_PC_ = 54.3 Hz, С(2)), 42.38 (*J*_PC_ = 53.3 Hz, C(2)), 128.30 (*J*_PC_ = 12.1 Hz, C(8), C(10)), 130.00 (*J*_PC_ = 11.1 Hz, C(7), C(11)), 131.07 (*J*_PC_ = 2.0 Hz, C(9)), 133.76, 133.84 (*J*_PC_ = 70.4 Hz, C(6)) (P-Ph); ^31^P NMR (161.97 MHz, CDCl_3_) δ 57.72; MALDI TOF: *m*/*z* сalculated for C_16_H_26_PS ([M + H]^+^): 281.4174; found: 281.3.

### General procedure for the preparation of Mo complexes **13b**, **14g**

Reactions was performed under argon using standard Schlenk techniques. A mixture of Mo(CO)_6_ (0.79 g, 3 mmol) and 3-hexyl(benzyl)-1-phenyl(methyl)phospholane **2b**, **2g** (3 mmol) was stirred under reflux in 15 mL THF for 24 h. The colorless solution became dark-brown during this time. The solvent was removed under vacuum and the residue was chromatographed on silica gel (hexane). The product was obtained as dark green thick liquid. (3-Hexyl-1-phenylphospholane)pentacarbonylmolybdenum (**13b**) in the mixture in a ratio of 1:1: Calcd for C_21_H_25_MoO_5_P: C, 52.08%; H, 5.20%; found: C, 52.2%; H, 5.3%; ^1^H NMR (400.13 MHz, CDCl_3_) δ 0.20–1.01 (s, 6H, C(6′)H), 1.30–1.56 (s, 20H, C(5′)H, C(3′)H, C(2′)H, C(4′)H, C(1′)H), 1.58–1.64 (m, 2H, C(4)H), 1.72–1.82 (m, 1H, C(2)H_a_), 1.93–2.02 (m, 1H, C(3)H), 2.06–2.27 (m, 4H, C(4)H_b_, C(2)H_b_, C(3)H), 2.41–2.57 (m, 3H, C(5)H_a_, C(5)H_b_), 2.58–2.80 (m, 3Н, С(5)H_b_, С(2)H_a_, С(2)H_b_), 7.23–7.26, 7.31–7.33, 7.37–7.44, 7.45–7.56 (m, 10Н, Ph); ^13^C NMR (100.62 MHz, CDCl_3_) δ 14.10 (C(6')), 22.66, 22.72 (C(5')), 28.39, 28.42 (C(3')), 29.37, 29.44 (C(2')), 31.45 (*J*_PC_ = 22.1 Hz, C(5)), 31.81 (C(4')), 32.16 (*J*_PC_ = 24.1 Hz, C(5)), 33.42, 34.13 (C(4)), 35.70 (*J*_PC_ = 8.0 Hz, C(1')), 35.75 (*J*_PC_ = 9.1 Hz, C(1')), 38.19 (C(2)), 38.41 (*J*_PC_ = 3.0 Hz, C(2)), 41.78 (*J*_PC_ = 1.0 Hz, C(3)), 42.30 (C(3)), 128.81, 128.88 (*J*_PC_ = 10.1 Hz), 128.95 (*J*_PC_ = 11.1 Hz), 129.08, 139.67 (*J*_PC_ = 27.2 Hz, C(6)), 140.24 (*J*_PC_ = 28.2 Hz, C(6)) (P-Ph), 205.75 (*J*_PC_ = 9.1 Hz, CO*_cis_*), 205.77 (*J*_PC_ = 9.1 Hz, CO*_cis_*), 210.51 (*J*_PC_ = 22.1 Hz, CO*_trans_*), 210.57 (*J*_PC_ = 21.1 Hz, CO*_trans_*); ^31^P NMR (161.97 MHz, CDCl_3_): δ 26.00, 25.20.

### General procedure for the preparation of Mo complexes **15a**, **16c**

Reactions were performed under argon using standard Schlenk techniques. A mixture of Mo(CO)_6_ (0.79 g, 3 mmol) and 1,2(1,6)-bis(1-phenylphospholan-3-yl)ethane(hexane) **10a, 10c** (3 mmol) was stirred under reflux in 15 mL THF for 24 h. The colorless solution became brown during this time. The solvent was removed under vacuum and the crude product was purified by silica gel column chromatography (hexane) giving a dark brown, thick liquid. 1,2-Bis(1-pentacarbonylmolybdenum-1-phenylphospholan-3-yl)ethane (**15a**) as a mixture of isomers. Calcd for C_32_H_28_Mo_2_O_10_P_2_: C, 46.51%; H, 3.42%; found: C, 46.5%; H, 3.5%; ^1^H NMR (400.13 MHz, CDCl_3_) δ 1.28–1.39 (m, 2H, C(4)H_a_, C(4)H_b_), 1.40–1.60 (m, 2H, C(4)H_a_, C(4)H_b_), 1.64–1.73 (m, 1H, C(2)H_a_), 1.84–1.94 (m, 1H, C(3)H), 1.98–2.22 (m, 6H, C(6′), C(2)H_b_, C(3)H), 2.35–2.58 (m, 3H, C(5)H_a_, C(5)H_b_, C(2)H_a_), 2.60–2.70 (m, 3H, C(5)H_a_, C(5)H_b_, С(2)H_b_), 7.34–7.56 (m, 10H, P-Ph); ^13^C NMR (100.62 MHz, CDCl_3_) δ 31.29, 31.35 (*J*_PC_ = 23.1 Hz, C(5)), 32.01 (*J*_PC_ = 23.1 Hz, C(5)), 32.05 (*J*_PC_ = 24.1 Hz, C(5)), 33.35, 33.40, 34.13, 34.17, 34.22 (С(4)), 34.37, 34.44, 34.52, 34.59 (C(6')), 38.31, 38.35, 38.37 (*J*_PC_ = 23.1 Hz, C(2)), 38.50, 38.54 (C(2)), 41.74, 41.82, 41.91, 42.36, 42.42 (C(3)), 128.84, 128.94, 129.03, 129.19, 139.44, 140.11 (*J*_PC_ = 28.2 Hz, C(6)) (Р-Ph), 205.74, 205.83 (CO*_cis_*), 210.49, 210.55 (*J*_PC_ = 22.1 Hz, CO*_trans_*); ^31^P NMR (161.97 MHz, CDCl_3_) δ 25.3, 26.3.

## Supporting Information

Detailed synthesis and characterization procedures are provided for all compounds synthesized and characterized. NMR spectra are provided for all compounds for which NMR data are reported.

File 1 Experimental details, characterization data of all products.

File 2 NMR spectra.
