# Supplementary material for: Aluminacyclopentanes in the synthesis of 3-substituted phospholanes and α,ω-bisphospholanes
Source: Beilstein J Org Chem. 2016 Mar 2;12:406–12. doi: 10.3762/bjoc.12.43 (PMC4902032; doi:10.3762/bjoc.12.43)
Supplement: File 1 — Experimental details, characterization data of all products. [file Beilstein_J_Org_Chem-12-406-s001.pdf]

Supporting Information

for

Aluminacyclopentanes in the synthesis of 3-substituted phospholanes and α,ω-bisphospholanes

Vladimir A. D'yakonov*, Alevtina L. Makhamatkhanova, Rina A. Agliullina, Leisan K. Dilmukhametova, Tat'yana V. Tyumkina, and Usein M. Dzhemilev

Address: Institute of Petrochemistry and Catalysis of Russian Academy of Sciences, Prospekt Oktyabrya, Ufa 450075, Russian Federation

Email: Vladimir D'yakonov* - DyakonovVA@gmail.com

*Corresponding author

Experimental details, characterization data of all products

General remarks

Chromatographic analyses were performed on a Shimadzu GC-9A instrument using a 2000 × 2 mm column, the SE-30 (5%) stationary phase on Chromaton N-AW-HMDS (0.125–0.160 mm), helium carrier gas (30 mL/min), and temperature programming from 50 to 300 °C at a 8 °C/min rate. For the chromatographic analyses

a Waters Breeze (Waters, USA) liquid chromatograph with spectrophotometric detector was used. As mobile phase acetonitrile/water 30:70% (v/v) was employed. Analysis was performed on a Kinetex C18 column, 250 × 4,6 mm; 5 micron (Phenomenex, USA) at an analytical wave length of 225 nm. The rate of the moving phase was 1 mL/min, injected sample volume was 5 microliter. Acetonitrile HPLC-gradient grade (Panreac, Spain) and bidistilled water were used to prepare the eluents and dissolution standards. The ^{1}H, ^{13}C, and ^{31}P NMR spectra were measured in $CDCl_3$ on a Bruker Avance-400 spectrometer (100.62 MHz for ^{13}C, 400.13 MHz for ^{1}H, and 161.97 MHz for ^{31}P). Mass spectra were run on a MALDI–TOF/TOF Autoflex-III instrument from Bruker with 2,5-dihydroxybenzoic (2,5-DHB) and α-cyano-4-hydroxycinnanic acid (HCCA) matrix in the reflection, positive ion mode. TLC was performed on Silufol UV-254 plates with a hexane/ethyl acetate/methanol (5:3:1) mixture as the eluent and I_2 for visualization. For column chromatography, Acros silica gel (0.060–0.200 mm) was used. The product yields were determined by GLC analysis using the internal standard (undecane). Reactions with organometallic compounds were performed in a dry argon flow. The solvents were dried and distilled immediately prior to use. Commercially available phosphines, Cp_2ZrCl_2 and Et_3Al (Aldrich) were used.

General procedure and characterization data for compounds **2a**, **2b**, **2f**. **2g** and **3a**, **3b**, **3f**, **3g** have been described previously [1].

Preparation of 3-alkyl(aryl)phospholanes (general procedure)

A glass reactor maintained under dry argon at 0 °C was successively charged, while stirring, with toluene (25 mL), Cp_2ZrCl_2 (0.298 g, 1 mmol), olefin (10 mmol), and $AlEt_3$ (1.8 mL, 10 mmol). The mixture was warmed up to room temperature (ca. 20 °C) and

stirred for 12 h. Then the reaction mixture was cooled down to –5 to −10 °C, alkyl(phenyl)dichlorophosphine (10 mmol) was added dropwise, and the mixture was stirred at room temperature for an additional 30 min. Then the reaction mixture was treated with a saturated aqueous solution of NH_4Cl and the reaction products were extracted with diethyl ether and dried with $MgSO_4$. The solvent was evaporated and the target phospholanes were isolated by vacuum distillation. All operations were carried out in an argon flow.

Preparation of 3-alkyl(aryl)phospholane 1-oxides (general procedure)

A 30% solution of hydrogen peroxide (0.7 mL, 6 mmol) was slowly added dropwise with vigorous stirring to a solution of 3-alkyl(benzyl)-1-alkyl(phenyl)phospholane (5 mmol), synthesized as described above, in chloroform (10 mL) and the mixture was stirred for 1 h. Then the reaction mixture was washed with water (3 × 5 mL) and the organic layer was dried with $MgSO_4$. The solvent was evaporated and the residue was chromatographed on silica gel (hexane/ethyl acetate/methanol 5:3:1).

Preparationof 3-alkyl(aryl)phospholane 1-sulfides(generalprocedure)

Reactions were performed under argon. Sulfur (0.13 g, 4 mmol) was added with cooling to a solution of 3-alkyl(aryl)phospholane (4 mmol) (prepared as described above) in 10 mL toluene, and the mixture was stirred for 4 h. After filtration through a thin layer of silica gel the solvent was evaporated to give a colorless oil.

3-Octyl-1-phenylphospholane (**2c**) of the mixture in ratio 3/2.

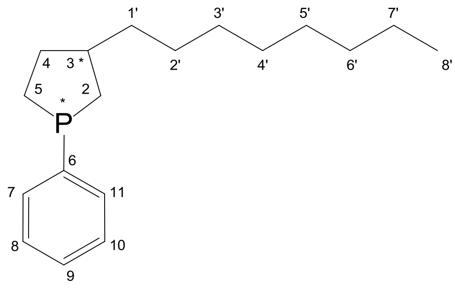

Colorless oil (87%), b.p. 215–218 ^{o}C (9 torr). Calculated for $C_{18}H_{29}P$: C, 78.22%; H, 10.58%. Found: C, 78.3%; H, 10.6%. ^{1}H NMR(400.13 MHz, $CDCl_3$): δ 0.95 (t, ^{3}J = 6.8 Hz, 6H, C(8')H), 1.27–

1.63 (m, 32H, C(1')H, C(2')H, C(3')H, C(4')H, C(5')H, C(6′)H, C(7′))H, C(4)H_a, C(2)H_a), 1.94–2.03 (m, 4H, C(3)H, C(5)H_a), 2.10–2.20 (m, 4H, C(2)H_b, C(4)H_b), 2.26 (m, 2H, C(5)H_b), 7.24–7.30, 7.32–7.38, 7.42–7.48 (m, 10H, Ph); ^{13}C NMR (100.62 MHz, $CDCl_3$): δ 14.15 (C(8')), 22.70 (C(7')), 25.79 (J_{PC} = 12.1 Hz,C(5)), 26.60 (J_{PC} = 10.1 Hz, C(5)), 28.49, 28.61 (C(2')), 29.04, 29.05 (C(4')), 29.32 (C(5')), 29.57, 29.64 (C(3')), 31.63 (C(6')), 32.92 (J_{PC} = 13.1 Hz, C(2)), 33.16 (J_{PC} = 11.1 Hz,C(2)), 33.95 (J_{PC} = 3.0 Hz, C(4)), 34.24 (J_{PC} = 4.0 Hz, C(4)), 35.60 (J_{PC} = 3.0 Hz, C(1')), 35.86 (J_{PC} = 5.0 Hz, C(1')), 41.82 (J_{PC} = 4.0 Hz, C(3)), 43.02 (J_{PC} = 1.0 Hz, C(3)), 126.87 (C(9)), 127.90, 127.91 (J_{PC} = 5.0 Hz,C(8), C(10)), 130.07 (J_{PC} = 16.1 Hz, C(7), C(11)), 130.13 (J_{PC} = 15.1 Hz, C(7), C(11)), 142.47, 142.84 (J_{PC} = 23.1 Hz, C(6)) (P-Ph); ^{31}P NMR (161.97 MHz, $CDCl_3$): δ -13.5, -13.9; MALDI TOF: *m*/*z* calculated for $C_{18}H_{30}P$ ($[M + H]^+$): 277.4046; found: 277.4.

3-Cyclohexyl-1-phenylphospholane (**2d**) of the mixture in ratio 3/2.

Colorless oil (93%), b.p. 198–203 ^oC (8 torr). Calculated for $C_{16}H_{23}P$: C, 78.01%; H, 9.41%. Found: C, 77.8%; H, 9.2%. ^{1}H NMR (400.13 MHz, $CDCl_3$): δ 0.94–1.12 (m, 4H, C(2')H_a, C(6')H_a), 1.14–1.43 (m, 8H, C(1')H, C(3')H_a, C(5')H_a, C(4)H_a), 1.52 (m, 1H, C(2)H_a), 1.57–2.02 (m, 18H, C(2)H_a, C(3)H, C(4)H_b, C(2')H_b, C(3')H_b, C(4')H, C(5)H_a, C(5')H_b, C(6')H_b), 2.07–2.36 (m, 4H, C(2)H_b, C(4)H_b, C(5)H_b), 2.43 (m, 1H, C(2)H_b), 7.25–7.31, 7.33–7.39, 7.43–7.50 (m, 10H, Ph); ^{13}C NMR (100.62 MHz, $CDCl_3$): δ 26.26 (J_{PC} = 11.1 Hz, C(5)), 26.54, 26.56, 26.57, 26.59 (C(3'), C(5'), 26.65 (C(4')), 26.93 (J_{PC} = 9.1 Hz, C(5)), 31.06 (J_{PC} = 14.1 Hz, C(2)), 31.09 (J_{PC} = 11.1 Hz, C(2)), 31.84, 31.86, 31.91 (C(4), 32.06, 32.14, 32.30, 32.57 (C(2'), C(6'), 43.13 (J_{PC} = 3.0 Hz, C(1')), 43.48 (J_{PC} = 5.0 Hz, C(1')), 48.20 (J_{PC} = 5.0 Hz, C(3)), 49.48 (J_{PC} = 2.0 Hz, C(3)), 127.17, 127.20 (C(9)), 128.24, 128.26 (J_{PC}= 5.0 Hz, C(8), C(10)), 130.35,

130.51 (J_{PC} = 16.1 Hz, C(7), C(11)), 142.94, 143.18 (J_{PC} = 23.1 Hz, C(6)) (P-Ph); ^{31}P NMR (161.97 MHz, $CDCl_3$): δ -14.7, -14.5, -14.4, -14.3; MALDI TOF: *m*/*z* calculated for $C_{16}H_{24}P$ ($[M + H]^+$): 247.3355; found: 247.4.

3-(Cyclohex-3-en-1-yl)-1-phenylphospholane (**2e**) of the mixture in ratio 3/2.

Colorless oil (90%), b.p. 209–214°C (8 torr). Calculated for $C_{16}H_{21}P$: C, 78.66%; H, 8.66%. Found: C, 78.5%; H, 8.4%. ^{1}H NMR (400.13 MHz, $CDCl_3$): δ 1.26–2.06 (m, 16H, C(1')H, C(2)H_a, C(2')H_b, C(3)H, C(2')H_a, C(4)H_a, C(5)H_a, C(6')H_a,), 2.07–2.39 (m, 11H, C(2)H_b, C(3')H, C(4)H_b, C(5)H_b, C(6')H_b), 2.47 (m, 1H, C(2)H_b), 5.17–5.30 (m, 4H, C(4')H, C(5')H), 7.27–7.33, 7.35–7.40,7.45–7.52 (m, 10H, Ph); ^{13}C NMR (100.62 MHz, $CDCl_3$): δ 25.26, 25.29, 25.32, 25.40 (C(3'), 25.90 (J_{PC} = 12.1 Hz, C(5)), 25.96 (J_{PC} = 11.1 Hz, C(5)), 26.62 (J_{PC} = 10.1 Hz, C(5)), 26.70 (J_{PC} = 9.1 Hz, C(5)), 27.55, 27.64, 27.86, 28.01 (C(2')), 30.50, 30.56 (C(6')), 30.56 (J_{PC} = 14.1 Hz, C(2)), 30.68 (J_{PC} = 11.1 Hz, C(2)), 30.82 (C(6')), 30.84 (J_{PC} = 13.1 Hz, C(2)), 30.93 (J_{PC} = 11.1 Hz, C(2)), 31.18 (C(6')), 31.59 (J_{PC} = 2.0 Hz, C(4)), 31.66 (J_{PC} = 2.0 Hz, C(4)), 31.72 (J_{PC} = 4.0 Hz, C(4)), 38.89 (J_{PC} = 3.0 Hz, C(1')), 38.92 (J_{PC} = 3.0 Hz, C(1')), 39.13 (J_{PC} = 5.0 Hz, C(1')), 39.40 (J_{PC} = 4.0 Hz, C(1')), 47.31, 47.36 (J_{PC} = 4.0 Hz, C(3)), 48.46, 48.51 (J_{PC} = 2.0 Hz, C(3)), 126.20 (C(4')), 126.24, 126.25 (C(4')), 126.60, 126.71, 126.72 (C(5')), 126.97, 126.98 (C(9)), 128.00, 128.02 (J_{PC} = 5.0 Hz, C(8), C(10)), 130.07 (J_{PC} = 15.1 Hz, C(7), C(11)), 130.08, 130.23 (J_{PC} = 14.1 Hz, C(7), C(11)),142.51 (J_{PC}= 22.1 Hz, C(6)), 142.55,142.74, 142.77 (J_{PC}= 23.1 Hz, C(6)); ^{31}P NMR (161.97 MHz, $CDCl_3$): δ -14.1, -14.0, -14.5; MALDI TOF: *m*/*z* calculated for $C_{16}H_{22}P$ ($[M + H]^+$): 245.3196; found: 245.2.

3-Benzyl-1-butylphospholane (**2h**) of the mixture in ratio 2/1.

Colorless oil (86%), b.p. 177–180 ^{o}C (9 torr). Calculated for $C_{15}H_{23}P$: C, 76.89%; H, 9.89%. Found: C, 76.7%; H, 9.7%. ^{1}H NMR (400.13 MHz, $CDCl_3$): δ 0.88–0.99 (m, 6H, C(9)H), 1.07 (m, 1H, C(2)H_a), 1.28–1.48 (m, 15H, C(2)H_a, C(4)H_a, C(5)H, C(6)H, C(8)H), 1.53–1.65 (m, 5H, C(2)H_b, C(7)H), 1.92–2.13 (m, 4H, C(2)H_b, C(3)H, C(4)H_b), 2.31 (m, 1H, C(3)H), 2.62–2.73 (m, 2H, C(1′)H), 2.73–2.83 (m, 2H, C(1′)H), 7.15–7.26, 7.27–7.35 (m, 10H, Ph); ^{13}C NMR (100.62 MHz, $CDCl_3$): δ 13.86, 13.88 (C(9)), 24.26, 24.30 (J_{PC} = 12.1 Hz, C(8)), 24.80 (J_{PC} = 11.1 Hz, C(5)), 24.84 (J_{PC} = 9.1 Hz, C(5)), 29.00, 29.25 (J_{PC} = 15.1 Hz, C(7)), 29.53 (J_{PC} = 15.1 Hz, C(6)), 31.81 (J_{PC} = 11.1 Hz, C(2)), 32.31 (J_{PC} = 13.1 Hz, C(2)), 33.45 (J_{PC} = 4.0 Hz, C(4)), 34.09 (J_{PC} = 2.0 Hz, C(4)), 41.94 (J_{PC} = 4.0 Hz, C(1')), 42.64 (J_{PC} = 5.0 Hz, C(1')), 43.47 (J_{PC} = 4.0 Hz, C(3)), 45.14 (J_{PC} = 2.0 Hz, C(3)), 125.78, 128.21, 128.23, 128.74, 128.81, 141.61 (CH_2-Ph); ^{31}P NMR (161.97 MHz, $CDCl_3$): δ -23.7, -22.9; MALDI TOF: *m*/*z* calculated for $C_{15}H_{24}P$ ($[M + H]^+$): 235.3248; found: 235.1.

3-Octyl-1-phenylphospholane-1-oxide (**3c**) of the mixture in ratio 3/2.

Calculated for $C_{18}H_{29}OP$: C, 73.94; H, 10.00%. Found: C, 73.7%; H, 9.8%. ^{1}H NMR (400.13 MHz, $CDCl_3$): δ 0.81 (t, ^{3}J = 7.2 Hz, 6H, C(8')H), 1.17–1.60 (m, 30H, C(2)H_a, C(4)H_a, C(1')H, C(2')H, C(3')H, C(4')H, C(5')H, C(6')H, C(7')H), 1.70 (m, 1H, C(2)H_a), 1.80 (m, 1H, C(4)H_a), 1.87 (m, 1H, C(5)H_a), 1.94–2.12 (m, 2H, C(3)H, C(5)H_a), 2.14–2.35 (m, 7H, C(2)H_b, C(3)H, C(4)H_b, C(5)H_b), 7.36–7.56, 7.60–7.78 (m, 10H, Ph); ^{13}C NMR (100.62 MHz, $CDCl_3$): δ 13.82 (C(8')), 22.35 (C(7')), 27.51, 27.60 (C(2')), 28.94, 28.96 (C(4')), 29.19 (J_{PC} = 66.4 Hz, C(5)), 29.21 (C(5')), 29.32, 29.36 (C(3')), 30.18 (J_{PC} = 66.4 Hz, C(5)), 30.99 (J_{PC} = 6.0 Hz, C(4)), 31.55 (C(6')), 31.93 (J_{PC} = 7.0 Hz C(4)), 35.87 (J_{PC} = 55.3 Hz, C(2)), 35.99 (J_{PC} = 12.1 Hz, C(1')), 36.04 (J_{PC} = 14.1 Hz,

C(1')), 36.16 (J_{PC} = 53.3 Hz, C(2)), 38.60, 40.21 (J_{PC} = 8.0 Hz, C(3)), 128.39 (J_{PC} = 12.1 Hz, C(8), C(10)), 129.55 (J_{PC} = 10.1 Hz, C(7), C(11)), 131.40 (J_{PC} = 3.0 Hz, C(9)), 133.90 (J_{PC} = 89.5 Hz, C(6)), 134.00 (J_{PC} = 90.5 Hz, C(6)) (P-Ph); ^{31}P NMR (161.97 MHz, $CDCl_3$): δ 59.7, 59.4; MALDI TOF: *m*/*z* calculated for $C_{18}H_{30}OP$ ([M + H]$^+$): 293.4040; found: 293.5.

3-Cyclohexyl-1-phenylphospholane-1-oxide (**3d**) of the mixture in ratio 3/2.

Calculated for $C_{16}H_{23}OP$: C, 73.26%; H, 8.84%. Found: C, 72.1%; H, 8.6%. ^{1}H NMR (400.13 MHz, $CDCl_3$): δ 0.78-0.97 (m, 4H, C(2')H_a, C(6')H_b), 0.98-1.21 (m, 8H, C(3')H_b, C(4')H_a, C(5')H_a, C(1')H), 1.22-1.34 (m, 1H, C(4)H_a), 1.36-1.48 (m, 1H, C(2)H_a), 1.50-1.82 (m, 14H, C(2')H_b, C(3')H_a, C(4')H_b, C(5')H_b, C(6')H_a, C(4)H_a, C(2)H_a, C(5)H_a, C(3)H), 1.86-2.32 (m, 8H, C(5)H_a, C(5)H_b, C(2)H_b, C(4)H_b, C(3)H), 7.32–7.44, 7.57–7.66 (m, 10H, Ph); ^{13}C NMR (100.62 MHz, $CDCl_3$): δ 25.78 (C(4')), 25.95, 25.98 (C(3'), C(5')), 28.00, 29.29 (J_{PC} = 6.0 Hz, C(4)), 29.33, 30.16 (J_{PC} = 66.4 Hz, C(5)), 30.59, 30.65, 30.92, 31.08 (C(2'), C(6')), 33.28 (J_{PC} = 69.4 Hz, C(2)), 34.01 (J_{PC} = 67.4 Hz, C(2)), 42.79, 42.87 (J_{PC} = 12.1 Hz, C(1')), 44.23, 45.69 (J_{PC} = 8.1 Hz, C(3)), 128.23 (J_{PC} = 11.1 Hz, C(8), C(10)), 129.43 (J_{PC} = 9.1 Hz, C(7), C(11)), 131.21 (J_{PC} = 2.0 Hz, C(9)), 134.06 (J_{PC} = 89.5 Hz, C(6)) (P-Ph); ^{31}P NMR (161.97 MHz, $CDCl_3$): δ 59.4; MALDI TOF: *m*/*z* calculated for $C_{16}H_{24}OP$ ([M + H]$^+$): 263.3349; found: 263.4.

3-(Cyclohex-3-en-1-yl)-1-phenylphospholane-1-oxide (**3e**) of the mixture in ratio 3/2.

Calculated for $C_{16}H_{21}OP$: C, 73.82%; H, 8.13%. Found: C, 73.7%; H, 8.0%. ^{1}H NMR (400.13 MHz, $CDCl_3$): δ 1.13 (m, 2H, C(2')H_a), 1.20–1.48 (m, 4H, C(2)H_a, C(4)H_a, C(1')H), 1.53–1.78 (m, 8H, C(2)H_a, C(4)H_a, C(5)H_a, C(3)H, C(2')H_b, C(6')H_b, C(6')H_a),

1.79–2.33 (m, 14H, C(2)H_b, C(3)H, C(4)H_b, C(3')H, C(5)H_a, C(6')H_b, C(5)H_b), 5.43–5.55 (m, 4H, C(4')H, C(5')H), 7.26–7.38, 7.52–7.62 (m, 10H, Ph); ^{13}C NMR (100.62 MHz, $CDCl_3$): δ 25.02 (C(3')), 26.56, 26.59, 27.04, 27.09 (C(2')), 28.34, 28.36 (J_{PC} = 6.0 Hz, C(4)), 29.38, 29.46 (J_{PC} = 66.4 Hz, C(5)), 29.61, 29.64 (J_{PC} = 6.0 Hz, C(4)), 29.73, 29.94, 29.96 (C(6')), 30.36, 30.38 (J_{PC} = 66.4 Hz, C(5)), 33.56, 33.79, 34.18, 34.44 (J_{PC} = 68.4 Hz, C(2)), 39.05 (J_{PC} = 12.1 Hz, C(1')), 39.15, 39.28, 39.41 (J_{PC} = 13.1 Hz, C(1')), 43.80, 43.85, 45.12, 44.14 (J_{PC} = 8.1 Hz, C(3)), 125.65, 125.72 (C(5')), 126.84, 126.86, 126.90 (C(4')), 128.50 (J_{PC} = 12.1 Hz, C(8), C(10)), 129.67 (J_{PC} = 10.1 Hz, C(7), C(11)), 131.48 (J_{PC} = 2.0 Hz, C(9)), 134.31, 134.34 (J_{PC} = 89.5 Hz, C(6)) (P-Ph); ^{31}P NMR (161.97 MHz, $CDCl_3$): δ 58.7, 58.4; MALDI TOF: *m*/*z* calculated for $C_{16}H_{22}OP$ ($[M + H]^+$): 261.3190; found: 261.4.

3-Benzyl-1-butylphospholane-1-oxide (**3h**) of the mixture in ratio 2/1.

Calculated for $C_{15}H_{23}OP$: C, 71.97%; H, 9.26%. Found: C, 71.8%; H, 9.0%. ^{1}H NMR (400.13 MHz, $CDCl_3$): δ 0.78–0.86 (m, 6H, C(9)H), 1.08 (m, 1H, C(4)H_a), 1.14 (m, 1H, C(2)H_a), 1.26–1.38 (m, 4H, C(8)H), 1.41 (m, 1H, C(2)H_a), 1.44–1.55 (m, 4H, C(7)H), 1.57–1.70 (m, 5H, C(5)H_a, C(6)H), 1.79–1.97 (m, 8H, C(5)H, C(2)H_b, C(4)H_a, C(4)H_b, C(5)H_b, C(3)H), 2.02 (m, 1H, C(4)H_b), 2.41 (m, 1H, C(3)H), 2.50–2.58 (m, 2H, C(1′)H), 2.58–2.65 (m, 2H, C(1′)H), 6.98–7.05, 7.05–7.12, 7.12–7.20, 7.28 (m, 10H, Ph); ^{13}C NMR (100.62 MHz, $CDCl_3$): δ 13.51, 13.53 (C(9)), 23.85 (C(8)), 23.96 (J_{PC} = 4.0 Hz, C(7)), 23.98 (C(8)), 24.06 (J_{PC} = 5.0 Hz, C(7)), 26.50 (J_{PC} = 64.4 Hz, C(5)), 27.79 (J_{PC} = 62.4 Hz, C(5)), 30.15 (J_{PC} = 5.0 Hz, C(4)), 30.50 (J_{PC} = 6.0 Hz, C(4)), 30.75, 30.84 (J_{PC} = 62.4 Hz, C(6)), 32.79 (J_{PC} = 65.4 Hz, C(2)), 33.50 (J_{PC} = 64.4 Hz, C(2)), 40.17 (J_{PC} = 8.1 Hz, C(3)), 40.43 (J_{PC} = 10.1 Hz, C(3)), 41.00, 42.09 (J_{PC} = 12.1 Hz, C(1')), 126.16 (C(5′)), 128.30, 128.33 (C(4′), C(6′)), 128.65, 128.72 (C(3′), C(7′)),

139.42, 139.66 (C(2′)) (CH_2-Ph); ^{31}P NMR (161.97 MHz, $CDCl_3$): δ 69.9, 70.3; MALDI TOF: *m*/*z* calculated for $C_{15}H_{24}OP$ ($[M + H]^+$): 251.3242; found: 251.5.

1,3-Diphenylphospholane-1-oxide (**7d**) of the mixture in ratio 2/1.

Calculated for $C_{16}H_{17}OP$: C, 74.99%; H, 6.69%. Found: C, 74.8%; H, 6.7%. ^{1}H NMR (400.13 MHz, $CDCl_3$): δ 1.90 (m, 1H, C(4)H_a), 1.96–2.08 (m, 3H, C(2)H_a, C(5)H_a), 2.14–2.32 (m, 4H, C(2)H_b, C(4)H_b, C(5)H_a, C(5)H_b), 2.34-2.60 (m, 4H, C(2)H_b, C(4)H_b, C(5)H_b), 3.21, 3.62 (m, 2H, C(3)H), 7.23, 7.29, 7.30, 7.35 (m, 10H, Ph), 7.46, 7.56, 7.73, 7.84 (m, 10H, P-Ph); ^{13}C NMR (100.62 MHz, $CDCl_3$): δ 30.76 (J_{PC}= 65.4 Hz, C(5)), 31.90 (J_{PC} = 64.4 Hz, C(5)), 33.62, 34.32 (J_{PC} = 4.0 Hz, C(4)), 38.18 (J_{PC} = 67.4 Hz, C(2)), 38.41 (J_{PC} = 66.4 Hz, C(2)), 44.60 (J_{PC} = 10.1 Hz, C(3)), 46.08 (J_{PC} = 11.1 Hz, C(3)), 127.65, 127.75 (C(2'), C(6')), 128.03, 128.05 (C(3'), C(5')), 129.78, 129.80 (C(4')), 129.86 (J_{PC} = 12.1 Hz, C(8), C(10)), 130.94 (J_{PC} = 10.1 Hz, C(7), C(11)), 132.96 (J_{PC} = 3.0 Hz, C(9)), 134.82, 134.96 (J_{PC} = 90.5 Hz, C(6)), 143.59, 143.62 (J_{PC} = 14.1 Hz, C(1')); ^{31}P NMR (161.97 MHz, $CDCl_3$): δ 57.9, 58.7; MALDI TOF: *m*/*z* calculated for $C_{16}H_{18}OP$ ($[M + H]^+$): 257.2873; found: 257.1.

1,2-Diphenyl-1-phospholane-1-oxide (**8d**) of the mixture in ratio 2/1.

Calculated for $C_{16}H_{17}OP$: C, 74.99%; H, 6.69%. Found: C, 74.8%; H, 6.7%. ^{1}H NMR (400.13 MHz, $CDCl_3$): δ 2.07-2.18 (m, 1H, C(3)H_a), 2.23-2.40 (m, 7H, C(4)H_a, C(4)H_b, C(5)H_a, C(5)H_b), 2.41-2.49 (m, 4H, C(3)H_a, C(3)H_b, C(5)H_b), 3.57-3.62, 3.63-3.68 (m, 2H, C(2)H), 6.96-7.07, 7.16-7.31, 7.33-7.44, 7.48-7.58, 7.66-7.78 (m, 20H, Ph); ^{13}C NMR (100.62 MHz, $CDCl_3$): δ 22.00, 23.12 (J_{PC} = 4.0 Hz, C(4)), 26.01 (J_{PC} = 66.4 Hz, C(5)), 28.94 (J_{PC} = 15.1 Hz, C(3)), 29.75 (J_{PC} = 67.4 Hz, C(5)), 32.42 (J_{PC} = 11.1 Hz, C(3)), 48.88 (J_{PC} = 64.4 Hz, C(2)), 49.40 (J_{PC} = 62.4 Hz, C(2)), 126.25 (J_{PC} = 3.0 Hz),

126.89 (J_{PC} = 4.0 Hz), 127.89 (J_{PC} = 11.1 Hz), 128.02, 128.04, 128.39, 128.44, 128.64 (J_{PC} = 17.1 Hz), 128.76 (J_{PC} = 17.1 Hz), 129.79 (J_{PC} = 11.1 Hz), 130.05 (J_{PC} = 9.1 Hz), 130.66 (J_{PC} = 9.1 Hz), 131.45 (J_{PC} = 3.0 Hz), 131.72 (J_{PC} = 3.0 Hz), 132.37 (J_{PC} = 3.0 Hz), 135.23 (J_{PC} = 3.0 Hz); ^{31}P NMR (161.97 MHz, $CDCl_3$): δ 53.5, 60.7; MALDI TOF: *m/z* calculated for $C_{16}H_{18}OP$ ($[M + H]^+$): 257.2873; found: 257.1.

3-Naphthyl-1-phenylphospholane-1-oxide (**7f**) of the mixture in ratio 2/1.

Calculated for $C_{20}H_{19}OP$: C, 78.41%; H, 6.25%. Found: C, 78.4%; H, 6.3%. ^{1}H NMR (400.13 MHz, $CDCl_3$): δ 1.96-2.06 (m, 1H, C(4)H_a), 2.08-2.18 (m, 3H, C(5)H_a,C(2)H_a), 2.24-2.36 (m, 2H, C(5)H_b, C(5)H_a), 2.40-2.70 (m, 4H, C(5)H_b, C(4)H_a, C(4)H_b), 2.74-3.03 (m, 2H, C(2)H_b), 3.37-3.44 (m, 1H, C(3)H), 3.77-3.88 (m, 1H, C(2)H), 7.37-7.49, 7.58-7.68, 7.69-7.73, 7.74-7.76 (m, 14H, C-Naphtyl), 7.78-7.87 (m, 10H, Ph); ^{13}C NMR (100.62 MHz, $CDCl_3$): δ 29.65 (J_{PC} = 65.5 Hz, C(5)), 30.76 (J_{PC} = 64.9 Hz, C(5)), 32.37 (J_{PC} = 4.4 Hz, C(4)), 33.05 (J_{PC} = 4.2 Hz, C(4)), 36.99 (J_{PC} = 66.9 Hz, C(2)), 37.31 (J_{PC} = 65.9 Hz, C(2)), 43.57, 45.03 (J_{PC} = 9.9 Hz, C(3)), 124.64, 124.91, 124.99, 125.63, 126.14, 126.17, 127.46, 127.50, 128.30, 128.36, 128.66 (J_{PC} = 11.2 Hz, C(8), C(10)), 129.79 (J_{PC} = 9.8 Hz, C(7), C(11)), 131.74 (J_{PC} = 2.0 Hz), 132.30, 132.34, 133.28, 133.29, 133.80 (J_{PC} = 91.5 Hz, C(6)), 133.95 (J_{PC} = 90.5 Hz, C(6)), 139.86 (J_{PC} = 13.1 Hz, C(1')), 139.88 (J_{PC} = 15.1 Hz, C(1')); ^{31}P NMR (161.97 MHz, $CDCl_3$): δ 57.5, 58.2; MALDI TOF: *m/z* calculated for $C_{20}H_{20}OP$ ($[M + H]^+$): 307.338; found: 307.1.

2-Naphthyl-1-phenylphospholane-1-oxide (**8f**) of the *trans-* or *cis-* compound.

Calculated for $C_{20}H_{19}OP$: C, 78.41%; H, 6.25%. Found: C, 78.4%; H, 6.3%. ^{1}H NMR (400.13 MHz, $CDCl_3$): δ 1.86–2.00 (m, 1H, C(4)H_a), 2.25-2.39 (m, 1H, C(5)H_a), 2.40-

2.54 (m, 3H, C(4)H_b, C(5)H_b, C(3)H_a), 2.56-2.64 (m, 1H, C(3)H_b), 3.25-3.37 (m, 1H, C(2)H), 7.30–7.37, 7.39–7.45, 7.47-7.58 (m, 7H, C-Naphtyl), 7.66–7.71, 7.73–7.79, 7.82-7.88 (m, 5H, P-Ph); ^{13}C NMR (100.62 MHz, $CDCl_3$): δ 23.39 (J_{PC} = 4.0 Hz, C(4)), 30.15 (J_{PC} = 66.4 Hz, C(5)), 32.79 (J_{PC} = 11.1 Hz, C(3)), 49.32 (J_{PC} = 64.4 Hz, C(2)), 125.56, 125.92, 126.91 (J_{PC} = 4.0 Hz), 127.11 (J_{PC} = 6.0 Hz), 127.60, 127.70, 128.12, 128.77 (J_{PC} = 11.1 Hz), 130.26 (J_{PC} = 10.1 Hz), 131.84 (J_{PC} = 2.0 Hz), 133.03 (J_{PC} = 96.6 Hz), 140.07 (J_{PC} = 10.1 Hz); ^{31}P NMR (161.97 MHz, $CDCl_3$): δ 53.2; MALDI TOF: *m*/*z* calculated for $C_{20}H_{20}OP$ ([M + H]$^+$): 307.338; found: 307.1.

3-Naphthyl-1-methylphospholane-1-oxide (**7e**) of the mixture in ratio 2/1.

Calculated for $C_{15}H_{17}OP$: C, 73.76%; H, 7.01%. Found: C, 73.8%; H, 7.0%. ^{1}H NMR (400.13 MHz, $CDCl_3$): δ 1.56-1.79 (m, 10H, C(6), C(4)H_a, C(4)H_b, C(2)H_a), 1.93-2.21 (m, 6H, C(5)H_a, C(5)H_b, C(4)H_a, C(4)H_b), 2.24-2.43 (m, 2H, C(2)H_b), 3.50-3.60 (m, 2H, C(3)), 7.25-7.30, 7.32-7.35, 7.36-47 (m, 14H, C- Naphtyl); ^{13}C NMR (100.62 MHz, $CDCl_3$): δ 16.45, 17.49 (J_{PC} = 62.4 Hz, C(6)), 29.30 (J_{PC} = 65.4 Hz, C(5)), 30.07 (J_{PC} = 64.4 Hz, C(5)), 31.68 (J_{PC} = 5.0 Hz, C(4)), 32.41 (J_{PC} = 3.0 Hz, C(4)), 35.75 (J_{PC} = 66.4 Hz, C(2)), 36.40 (J_{PC} = 64.4 Hz, C(2)), 43.57 (J_{PC} = 11.1 Hz, C(3)), 43.74 (J_{PC} = 9.0 Hz, C(3)), 124.70, 124.88 (J_{PC} = 6.0 Hz), 125.04, 125.10, 125.59, 125.79, 126.01, 126.33, 126.94 (J_{PC} = 4.0 Hz), 127.15 (J_{PC} = 6.0 Hz), 127.62, 128.18, 128.42, 128.47, 128.52, 132.42, 132.49 (J_{PC} = 6.0 Hz), 133.41, 133.51, 133.77 (J_{PC} = 4.0 Hz), 139.98 (J_{PC} = 14.1 Hz), 140.17 (J_{PC} = 14.1 Hz) (Aromatic signals for the compounds **7e** and **8e** overlap); ^{31}P NMR (161.97 MHz, $CDCl_3$): δ 64.6; MALDI TOF: *m*/*z* calculated for $C_{15}H_{18}OP$ ([M + H]$^+$): 245.2686; found: 245.1.

2-Naphthyl-1-methylphospholane-1-oxide (**8e**) of the mixture in ratio 2/1.

Calculated for $C_{15}H_{17}OP$: C, 73.76%; H, 7.01%. Found: C, 73.8%; H, 7.0%. ^{1}H NMR (400.13 MHz, $CDCl_3$): δ 1.04-1.09 (m, 3H, C(6)), 1.56-1.70 (m, 3H, C(6)), 1.92-2.21 (m, 8H, C(4)H_a, C(4)H_b, C(5)H_a, C(5)H_b), 2.24-2.43 (m, 4H, C(3)H_a, C(3)H_b), 2.83-2.90 (m, 2H, C(2)), 7.56-7.60, 7.61-7.66, 7.69-7.81 (m, 14H, C- Naphtyl); ^{13}C NMR (100.62 MHz, $CDCl_3$): δ 14.15, 17.60 (J_{PC} = 63.4 Hz, C(6)), 21.97 (J_{PC} = 5.0 Hz, C(4)), 22.24 (J_{PC} = 4.0 Hz, C(4)), 27.46 (J_{PC} = 65.4 Hz, C(5)), 28.45 (J_{PC} = 64.4 Hz, C(5)), 32.96 (J_{PC} = 11.1 Hz, C(3), 124.70, 124.88 (J_{PC} = 6.0 Hz), 125.04, 125.10, 125.59, 125.79, 126.01, 126.33, 126.94 (J_{PC} = 4.0 Hz), 127.15 (J_{PC} = 6.0 Hz), 127.62, 128.18, 128.42, 128.47, 128.52, 132.42, 132.49 (J_{PC} = 6.0 Hz), 133.41, 133.51, 133.77 (J_{PC} = 4.0 Hz), 139.98 (J_{PC} = 14.1 Hz), 140.17 (J_{PC} = 14.1 Hz) (Aromatic signals for the compounds **7e** and **8e** overlap); ^{31}P NMR (161.97 MHz, $CDCl_3$): δ 60.8, 66.9; MALDI TOF: *m/z* calculated for $C_{15}H_{18}OP$ ($[M + H]^+$): 245.2686; found: 245.1.

3-Phenyl-1-methylphospholane-1-oxide (**7a**) of the mixture in ratio 2/1.

Calculated for $C_{11}H_{15}OP$: C, 68.03%; H, 7.78%. Found: C, 68.1%; H, 7.8%. ^{1}H NMR (400.13 MHz, $CDCl_3$): δ 1.68, 1.70 (d, J_{PC} = 12.0 Hz, 6H, C(6)H), 1.62-1.82 (m, 6H, C(5)H_a, C(4)H_a, C(2)H_a), 1.91-2.21 (m, 2H, C(5)H_b), 2.23-2.41 (m, 4H, C(4)H_b, C(2)H_b), 3.44-3.51 (m, 2H, C(3)H), 7.19-7.26, 7.27-7.34 (m, 10H, Ph); ^{13}C NMR (100.62 MHz, $CDCl_3$): δ 17.46(J_{PC} = 62.4 Hz, C(6)), 17.59 (J_{PC} = 63.4 Hz, C(6)), 27.28 (J_{PC} = 65.4 Hz, C(5)), 28.43 (J_{PC} = 64.4 Hz, C(5)), 31.79 (J_{PC} = 5.0 Hz, C(4)), 32.54 (J_{PC} = 4.0 Hz, C(4)), 35.85 (J_{PC} = 66.4 Hz, C(2)), 36.44 (J_{PC} = 65.4 Hz, C(2)), 43.53 (J_{PC} = 11.1 Hz, C(3)), 43.63 (J_{PC} = 9.1 Hz, C(3)), 126.52, 126.63, 126.93, 126.99, 128.71, 128.74, 142.51 (J_{PC} = 15.1 Hz), 142.75 (J_{PC} = 14.1 Hz); ^{31}P NMR

(161.97 MHz, $CDCl_3$): δ 64.7, 65.1; MALDI TOF: *m/z* calculated for $C_{11}H_{14}OP$ ([M - H]$^+$): 193.21; found: 193.1.

2-Phenyl-1-methylphospholane-1-oxide (**8a**) of the mixture in ratio 2/1.

Calculated for $C_{11}H_{15}OP$: C, 68.03%; H, 7.78%. Found: C, 68.1%; H, 7.8%. ^{1}H NMR (400.13 MHz, $CDCl_3$): δ 1.68, 1.70 (d, J_{PC} = 12.4 Hz, 6H, C(6)H), 1.50-1.60 (s, 3H, C(6)H), 1.49-1.62 (m, 2H, C(4)H_a, C(4)H_b), 1.66-1.78 (m, 1H, C(5)H), 1.80-1.90 (m, 1H, C(3)H_a), 1.97-2.33 (m, 8H, C(4)H_a, C(4)H_b, C(5)H_b, C(5)H_a, C(3)H_a, C(3)H_b), 2.65-2.78 (m, 2H, C(2)H), 7.10-7.22, 7.24-7.34 (m, 10H, Ph); ^{13}C NMR (100.62 MHz, $CDCl_3$): δ 14.03 (J_{PC} = 50.3 Hz, C(6)), 16.35 (J_{PC} = 50.3 Hz, C(6)), 21.92 (J_{PC} = 3.0 Hz, C(4)), 22.19 (J_{PC} = 4.0 Hz, C(4)), 27.28 (J_{PC} = 51.3 Hz, C(5)), 28.55 (J_{PC} = 11.1 Hz, C(3)), 29.21 (J_{PC} = 52.3 Hz, C(5)), 32.98 (J_{PC} = 8.0 Hz, C(3)), 47.61 (J_{PC} = 51.3 Hz, C(2)), 47.86 (J_{PC} = 50.3 Hz, C(2)), 126.91, 126.98, 128.62, 128.66, 128.71, 128.84, 135.84 (J_{PC} = 4.0 Hz), 136.06 (J_{PC} = 2.0 Hz); ^{31}P NMR (161.97 MHz, $CDCl_3$): δ 60.3; MALDI TOF: *m/z* calculated for $C_{11}H_{14}OP$ ([M - H]$^+$): 193.21; found: 193.1.

3-Phenyl-1-butylphospholane-1-oxide (**7b**) of the mixture in ratio 2/1.

Calculated for $C_{14}H_{21}OP$: C, 71.16%; H, 8.96%. Found: C, 71.2%; H, 9.0%. ^{1}H NMR (400.13 MHz, $CDCl_3$): δ 0.82-1.00 (s, 6H, C(9)H), 1.38-1.50 (m, 4H, C(7), C(8)), 1.57-1.78 (m, 7H, C(7), C(8), C(5)H_a, C(2)H_a), 1.79-2.13 (m, 8H, C(5)H_a, C(5)H_b, C(6)H_a, C(6)H_b, C(4)H_a), 2.15-2.34 (m, 5H, C(5)H_b, C(4)H_b, C(2)H_b), 3.37-3.50 (m, 2H, C(3)H), 7.16-7.25, 7.26-7.33 (m, 10H, Ph); ^{13}C NMR (100.62 MHz, $CDCl_3$): δ 14.61, 14.67 (C(9)), 25.05, 25.19, 25.22, 25.26 (C(7), C(8)), 28.13 (J_{PC} = 60.3 Hz, C(5)), 29.46 (J_{PC} = 61.4 Hz, C(5)), 31.85, 31.95 (J_{PC} = 62.4 Hz, C(6)), 33.00 (J_{PC} = 5.0 Hz, C(4)), 33.45 (J_{PC} = 4.0 Hz, C(4)), 35.59 (J_{PC} = 64.4 Hz, C(2)), 35.98 (J_{PC} = 63.4 Hz,

C(2)), 44.39 (J_{PC} = 9.1 Hz, C(3)), 45.01 (J_{PC} = 11.1 Hz, C(3)), 127.57, 127.70, 127.97, 128.04, 129.75, 129.78, 143.57 (J_{PC} = 14.1 Hz), 143.86 (J_{PC} = 13.1 Hz); ^{31}P NMR (161.97 MHz, $CDCl_3$): δ 69.7; MALDI TOF: *m*/*z* calculated for $C_{14}H_{22}OP$ ([M + H]$^+$): 237.2897; found:237.1.

2-Phenyl-1-butylphospholane-1-oxide (**8b**) of the mixture in ratio 2/1.

Calculated for $C_{14}H_{21}OP$: C, 71.16%; H, 8.96%. Found: C, 71.2%; H, 9.0%. ^{1}H NMR (400.13 MHz, $CDCl_3$): δ 0.73-0.84 (s, 3H, C(9)), 0.90-1.03 (s, 3H, C(9)), 1.36-1.53 (m, 8H, C(4)H_a, C(7), C(8), C(6)H_a, C(5)H_a), 1.56-1.68 (m, 6H, C(4)H_b, C(7), C(8)), 1.78-1.96 (m, 2H, C(3)H_a, C(5)H_b), 2.11-2.22 (m, 6H, C(6)H_a, C(6)H_b, C(5)H_a, C(5)H_b, C(3)H_b), 2.25-2.45 (m, 2H, C(3)H_a, C(3)H_b), 2.78-2.89 (m, 2H, C(2)H), 7.25-7.30, 7.33-7.38 (m, 10H, Ph); ^{13}C NMR (100.62 MHz, $CDCl_3$): δ 13.66, 13.59, 13.66 (C(9)), 22.19 (J_{PC} = 3.0 Hz), 23.13 (J_{PC} = 4.0 Hz), 23.98, 24.05, 24.08, 24.16, 24.26, 24.29 (C(7), C(8)), 26.15 (J_{PC} = 50.3 Hz, C(6)), 27.68 (J_{PC} = 50.3 Hz, C(5)), 27.88 (J_{PC} = 50.3 Hz, C(6)), 29.02 (J_{PC} = 11.1 Hz, C(3)), 30.24 (J_{PC} = 49.3 Hz, C(5)), 33.63 (J_{PC} = 8.0 Hz, C(3)), 46.12 (J_{PC} = 49.3 Hz, C(2)), 48.28 (J_{PC} = 47.3 Hz, C(2)), 126.80, 127.11 (J_{PC} = 3.0 Hz), 128.57, 128.74, 128.82, 128.86, 136.15 (J_{PC} = 2.0 Hz), 136.38 (J_{PC} = 3.0 Hz); ^{31}P NMR (161.97 MHz, $CDCl_3$): δ 63.5, 63.6; MALDI TOF: *m*/*z* calculated for $C_{14}H_{22}OP$ ([M + H]$^+$): 237.2897; found: 237.1.

3-Phenyl-1-*tert*-butylphospholane-1-oxide (**7c**) of the mixture in ratio 2/1.

Calculated for $C_{14}H_{21}OP$: C, 71.16%; H, 8.96%. Found: C, 71.2%; H, 8.9%. ^{1}H NMR (400.13 MHz, $CDCl_3$): δ 0.81-1.29 (d, 18H, C(7), C(8), C(9)), 1.39-1.52 (m, 2H, C(4)H_a), 1.68-1.94 (m, 4H, C(4)H_b, C(5)H_a), 2.07-2.23 (m, 3H, C(5)H_b, C(3)H), 2.25-2.43 (m, 1H, C(3)H), 2.87-3.01 (m, 2H, C(2)H_a), 3.49-3.63 (m, 2H, C(2)H_b), 7.14-

7.23, 7.24-7.33, 7.34-7.37, 7.84-8.07 (m, 10H, C-Ph); ^{13}C NMR (100.62 MHz, $CDCl_3$): δ 23.88, 24.63 (J_{PC} = 59.3 Hz, C(5)), 25.23 (C(9)), 25.53 (C(8)), 25.57 (C(7)), 31.88 (J_{PC} = 60.3 Hz, C(2)), 33.08 (J_{PC} = 53.3 Hz, C(2)), 33.73 (J_{PC} = 66.4 Hz, C(6)), 34.08 (J_{PC} = 3.0 Hz, C(4)), 44.11 (J_{PC} = 8.0 Hz, C(3)), 46.36 (J_{PC} = 8.0 Hz, C(3)), 127.52, 127.58, 127.67, 127.88,129.36, 143.99 (J_{PC} = 13.1 Hz); ^{31}P NMR (161.97 MHz, $CDCl_3$): δ 80.3; MALDI TOF: *m*/*z* calculated for $C_{14}H_{22}OP$ ($[M + H]^+$): 237.2897; found:237.1.

2-Phenyl-1-*tert*-butylphospholane-1-oxide (**8c**) of the mixture in ratio 2/1.

Calculated for $C_{14}H_{21}OP$: C, 71.16%; H, 8.96%. Found: C, 71.2%; H, 8.9%. ^{1}H NMR (400.13 MHz, $CDCl_3$): δ 0.81-1.29 (d, 18H, C(7), C(8), C(9)), 1.54-1.66 (m, 2H, C(4)H_a, C(4)H_b), 1.68-1.94 (m, 2H,C(5)H_a, C(5)H_b), 2.07-2.23 (m, 3H, C(5)H_b, C(2)H), 2.25-2.43 (m, 3H, C(2)H, C(4)H_a, C(4)H_b), 3.38-3.53 (m, 4H, C(3)H_a, C(3)H_b), 7.14-7.23, 7.24-7.33, 7.34-7.37, 7.84-8.07 (m, 10H, C-Ph); ^{13}C NMR (100.62 MHz, $CDCl_3$): δ 22.92, 23.84 (J_{PC} = 3.0 Hz, C(4)), 25.23 (C(9)), 25.20, 25.57 (C(7), C(8)), 25.83 (J_{PC} = 59.3 Hz, C(5)), 26.29 (J_{PC} = 58.3 Hz, C(5)), 30.78 (J_{PC} = 12.1 Hz, C(3)), 32.93 (J_{PC} = 63.4 Hz, C(6)), 35.27 (J_{PC} = 59.3 Hz, C(6)), 36.33 (J_{PC} = 9.1 Hz, C(3)), 42.73 (J_{PC} = 58.3 Hz, C(2)), 51.20 (J_{PC} = 53.3 Hz, C(2)), 128.64 (J_{PC} = 3.0 Hz), 129.44 (J_{PC} = 2.0 Hz), 129.68, 130.38 (J_{PC} = 5.0 Hz), 136.68 (J_{PC} = 2.0 Hz), 138.42 (J_{PC} = 5.0 Hz); ^{31}P NMR (161.97 MHz, $CDCl_3$): δ 73.5; MALDI TOF: *m*/*z* calculated for $C_{11}H_{16}OP$ ($[M + H]^+$): 237.2897; found: 237.1.

3-Butyl-1-phenylphospholane-1-sulfide (**4a**) of the mixture in ratio 3/2.

Calculated for $C_{14}H_{21}PS$: C, 66.63%; H, 8.39%. Found: C, 66.6%; H, 8.3%. ^{1}H NMR (400.13 MHz, $CDCl_3$): δ 0.84 (t, ^3J = 6.2 Hz, 6H, C(4')H), 1.22–1.35 (m, 8H, C(2')H,

C(3')H), 1.36–1.57 (m, 5H, C(1')H, C(4)H_a), 1.75–1.89 (m, 2H, C(2)H_a, C(4)H_a), 1.93 (m, 1H, C(2)H_a), 2.06–2.41 (m, 7H, C(3)H, C(2)H_b, C(4)H_b, C(5)H_a, C(5)H_b), 2.41 (m, 1H, C(3)H), 2.54 (m, 1H, C(5)H_b), 2.60 (m, 1H, C(2)H_b), 7.38–7.48, 7.80–7.88 (m, 10H, Ph); ^{13}C NMR (100.62 MHz, $CDCl_3$): δ 14.05 (C(4')), 22.67 (C(3')), 22.71 (C(3')), 30.11 (C(2')), 30.18 (C(2')), 32.16 (J_{PC} = 6.0 Hz, C(4)), 33.91 (J_{PC} = 4.0 Hz, C(4)), 35.35 (J_{PC} = 14.1 Hz, C(1')), 35.67 (J_{PC} = 12.1 Hz, C(1')), 36.43 (J_{PC} = 53.3 Hz, C(5)), 36.77 (J_{PC} = 54.3 Hz, C(5)), 40.01 (J_{PC} = 7.0 Hz, C(3)), 41.97 (J_{PC} = 6.0 Hz, C(3)), 42.23, 42.67 (J_{PC} = 53.3 Hz, C(2)), 129.77(J_{PC} = 12.1 Hz, C(8), C(10)), 131.45 (J_{PC} = 10.1 Hz, C(7), C(11)), 132.55 (C(9)), 135.15, 135.22 (J_{PC} = 70.4 Hz, C(6)) (P-Ph); ^{31}P NMR (161.97 MHz, $CDCl_3$): δ 57.7; MALDI TOF: *m*/*z* calculated for $C_{14}H_{22}PS$([M + H]$^+$): 253.3642; found: 253.3.

3-Hexyl-1-phenylphospholane-1-sulfide (**4b**) of the mixture in ratio 3/2.

Calculated for $C_{16}H_{25}PS$: C, 68.53%; H, 8.99%. Found: C, 68.4%; H, 8.0%. ^{1}H NMR (400.13 MHz, $CDCl_3$): δ 0.91 (t, ^3J = 6.8 Hz, 6H, C(6')H), 1.25–1.43 (m, 16H, C(2')H, C(3')H, C(4')H, C(5')H), 1.43–1.61 (m, 5H, C(1')H, C(4)H_a), 1.83–1.95 (m, 2H, C(2)H_a, C(4)H_a), 1.99 (m, 1H, C(2)H_a), 2.11–2.52 (m, 8H, C(2)H_b, C(3)H, C(4)H_b, C(5)H_a, C(5)H_b), 2.60 (m, 1H, C(5)H_b), 2.66 (m, 1H, C(2)H_b), 7.42–7.53, 7.82–7.94 (m, 10H, Ph); ^{13}C NMR (100.62 MHz, $CDCl_3$): δ 13.77 (C(6')), 22.27, 22.29 (C(5')), 27.62, 27.68 (C(2')), 29.00 (C(3')), 31.41, 31.43 (C(4')), 31.86 (J_{PC} = 6.0 Hz, C(4)), 33.62 (J_{PC} = 4.0 Hz, C(4)), 35.44 (J_{PC} = 14.1 Hz, C(1')), 35.64 (J_{PC} = 53.3 Hz, C(5)), 35.71 (J_{PC} = 12.1 Hz, C(1')), 36.49 (J_{PC} = 53.3 Hz, C(5)), 39.74 (J_{PC} = 8.0 Hz, C(3)), 41.78 (J_{PC} = 6.0 Hz, C(3)), 41.94 (J_{PC} = 54.3 Hz, C(2)), 42.38 (J_{PC} = 53.3 Hz, C(2)), 128.30 (J_{PC} = 12.1 Hz, C(8), C(10)), 130.00 (J_{PC} = 11.1 Hz, C(7), C(11)), 131.07 (J_{PC} = 2.0 Hz, C(9)), 133.76, 133.84 (J_{PC} = 70.4 Hz, C(6)) (P-Ph); ^{31}P NMR (161.97 MHz,

$CDCl_3$): δ 57.7; MALDI TOF: *m/z* calculated for $C_{16}H_{26}PS$ ($[M + H]^+$): 281.4174; found: 281.3.

3-Octyl-1-phenylphospholane-1-sulfide (**4c**) of the mixture in ratio 3/2.

Calculated for $C_{18}H_{29}PS$: C, 70.09%; H, 9.48%. Found: C, 70.0%; H, 9.4%. ^1H NMR (400.13 MHz, $CDCl_3$): δ 0.86 (t, ^3J = 6.8 Hz, 6H, C(8')H), 1.19–1.37 (m, 24H, C(2')H, C(3')H, C(4')H, C(5')H, C(6')H, C(7')H), 1.38–1.58 (m, 5H, C(1')H, C(4)H_a), 1.78–1.92 (m, 2H, C(2)H_a, C(4)H_a), 1.96 (m, 1H, C(2)H_a), 2.07–2.39 (m, 7H, C(3)H, C(4)H_b, C(5)H_b, C(5)H_a, C(2)H_b), 2.45 (m, 1H, C(3)H), 2.58 (m, 1H, C(5)H_b), 2.63 (m, 1H, C(2)H_b), 7.42–7.51, 7.82–7.91 (m, 10H, Ph); ^{13}C NMR (100.62 MHz, $CDCl_3$): δ 13.78 (C(8')), 22.31 (C(7')), 27.63, 27.68 (C(2')), 28.88 (C(4')), 28.88 (C(5')), 29.13, 29.15 (C(4')), 29.31 (C(3')), 31.50 (C(6')), 31.84 (J_{PC} = 5.0 Hz, C(4)), 33.60 (J_{PC} = 4.0 Hz, C(4)), 35.41 (J_{PC} = 14.1 Hz, C(1')), 35.61 (J_{PC} = 53.3 Hz, C(5)), 35.68 (J_{PC} = 12.1 Hz, C(1')), 36.46 (J_{PC} = 53.3 Hz, C(5)), 39.72 (J_{PC} = 7.0 Hz, C(3)), 41.75 (J_{PC} = 6.0 Hz, C(3)), 41.91, 42.35 (J_{PC} = 54.3 Hz, C(2)), 128.27 (J_{PC} = 12.1 Hz, C(8), C(10)), 129.96 (J_{PC} = 10.1 Hz, C(7), C(11)), 131.05 (J_{PC} = 3.0 Hz, C(9)), 133.71, 133.79 (J_{PC} = 70.4 Hz, C(6)) (P-Ph); ^{31}P NMR (161.97 MHz, $CDCl_3$): δ 57.7; MALDI TOF: *m/z* calculated for $C_{18}H_{30}PS$ ($[M + H]^+$): 309.4706; found:309.2.

3-Cyclohexyl-1-phenylphospholane-1-sulfide (**4d**) of the mixture in ratio 3/2.

Calculated for $C_{16}H_{23}PS$: C, 69.03%; H, 8.33%. Found: C, 68.8%; H, 8.1%. ^1H NMR (400.13 MHz, $CDCl_3$): δ 0.80–0.95 (m, 4H, C(2')H_a, C(6')H_a), 0.96–1.25 (m, 8H, C(1')H, C(3')H_a, C(4')H_a, C(5')H_a), 1.43 (m, 1H, C(4)H_a), 1.49–1.85 (m, 13H, C(2)H_a, C(4)H_a, C(3)H, C(2')H_{2b}, C(3')H_b, C(4')H_b, C(5')H_b, C(6')H_b), 1.93 (m, 1H, C(2)H_a), 2.04–2.36 (m, 7H, C(2)H_b, C(3)H, C(4)H_b, C(5)H_a, C(5)H_b), 2.47 (m, 2H, C(2)H_b,

C(5)H_b), 7.31–7.40, 7.73–7.82 (m, 10H, Ph); ^{13}C NMR (100.62 MHz, $CDCl_3$): δ 25.83 (C(3'), C(5′)), 25.97, 25.99 (C(4')), 29.23 (J_{PC} = 6.0 Hz, C(4)), 30.92 (C(2′), C(6′)), 31.13 (J_{PC} = 4.0 Hz, C(4)), 35.56, 36.35 (J_{PC} = 53.3 Hz, C(5)), 39.80 (J_{PC} = 55.3 Hz, C(2)), 40.42 (J_{PC} = 54.3 Hz, C(2)), 42.49 (J_{PC} = 13.1 Hz, C(1')), 42.77 (J_{PC} = 12.1 Hz, C(1')), 45.36 (J_{PC} = 7.0 Hz, C(3)), 47.49 (J_{PC} = 5.0 Hz, C(3)), 128.21 (J_{PC} = 12.1 Hz, C(8), C(10)), 129.91, 129.94 (J_{PC} = 10.1 Hz, C(7), C(11)), 131.00 (J_{PC} = 3.0 Hz, C(9)), 133.67, 133.70 (J_{PC} = 70.4 Hz, C(6)) (P-Ph); ^{31}P NMR (161.97 MHz, $CDCl_3$): δ 57.2; MALDI TOF: *m*/*z* calculated for $C_{16}H_{24}PS$ ($[M + H]^+$): 279.4015; found: 279.392.

3-(Cyclohex-3-en-1-yl)-1-phenylphospholane-1-sulfide (**4e**) of the mixture in ratio 3/2. Calculated for $C_{16}H_{21}PS$: C, 69.53%; H, 7.66%. Found: C, 69.5%; H, 7.5%. ^{1}H NMR (400.13 MHz, $CDCl_3$): δ 1.23 (m, 2H, C(2')H_a), 1.44–1.63 (m, 4H, C(1')H, C(4)H_a, C(1')H), 1.65–2.45 (m, 21H, C(2')H_b, C(3')H, C(6')H, C(2)H_a, C(2)H_b, C(3)H, C(4)H_b, C(4)H_a, C(5)H_a, C(5)H_b), 2.55 (m, 1H, C(5)H_b), 2.59 (m, 1H, C(2)H_b), 5.52–5.66 (m, 4H, C(4′)H, C(5′)H), 7.33–7.48, 7.76–7.90 (m, 10H, Ph); ^{13}C NMR (100.62 MHz, $CDCl_3$): δ 24.85, 24.87 (C(3')), 26.66, 27.00 (C(2')), 29.32, 29.37 (J_{PC} = 4.0 Hz, C(4)), 29.83, 29.88 (C(6′)), 31.21, 31.24 (J_{PC} = 4.0 Hz, C(4)), 35.49, 35.57, 36.30, 36.33 (J_{PC} = 53.3 Hz, C(5)), 38.63 (J_{PC} = 14.1 Hz, C(1')), 38.76 (J_{PC} = 12.1 Hz, C(1')), 38.86 (J_{PC} = 14.1 Hz, C(1')), 39.04 (J_{PC} = 12.1 Hz, C(1')), 39.77, 40.01 (J_{PC} = 55.3 Hz, C(2)), 40.27, 40.61 (J_{PC} = 54.3 Hz, C(2)), 44.70 (J_{PC} = 7.0 Hz, C(3)), 44.80 (J_{PC} = 8.0 Hz, C(3)), 46.64, 46.84 (J_{PC} = 5.0 Hz, C(3)), 125.46, 125.49, 125.53 (C(5′)), 126.67, 126.69, 126.74 (C(4′)), 128.30 (J_{PC} = 12.1 Hz, C(8), C(10)), 129.95, 129.98 (J_{PC} = 10.1 Hz, C(7), C(11)), 131.11 (J_{PC} = 3.0 Hz, C(9)), 133.62, 133.65 (J_{PC} = 70.4 Hz, C(6)) (P-Ph); ^{31}P NMR (161.97 MHz, $CDCl_3$): δ 57.2; MALDI TOF: *m*/*z* calculated for $C_{16}H_{22}PS$ ($[M + H]^+$): 277.3856; found: 277.4.

3-Benzyl-1-phenylphospholane-1-sulfide (**4f**) of the mixture in ratio 2/1.

Calculated for $C_{17}H_{19}PS$: C, 71.30%; H, 6.69%. Found: C, 71.2%; H, 6.5%. ^{1}H NMR (400.13 MHz, $CDCl_3$): δ 1.60 (s, 1H, C(4)H_a), 1.97–2.11 (m, 3H, C(2)H_a, C(4)H_a), 2.17–2.55 (m, 6H, C(2)H_b, C(3)H, C(4)H_b, C(5)H_a), 2.55–2.68 (m, 3H, C(5)H_b, C(2)H_b), 2.77–2.89 (m, 5H, C(1′)H, C(3)H), 7.12–7.24, 7.24–7.32 (m, 10H, CH_2-Ph), 7.39–7.53, 7.80–7.91 (m, 10H, P-Ph); ^{13}C NMR (100.62 MHz, $CDCl_3$): δ 32.06 (J_{PC} = 5.0 Hz, C(4)), 33.56 (J_{PC} = 3.0 Hz, C(4)), 35.77, 36.69 (J_{PC} = 53.3 Hz, C(5)), 41.66 (J_{PC} = 13.1 Hz, C(1')), 41.77 (J_{PC} = 7.0 Hz, C(3)), 41.86 (J_{PC} = 53.3 Hz, C(2)), 42.03 (J_{PC} = 12.1 Hz, C(1')), 42.32 (J_{PC} = 53.3 Hz, C(2)), 43.70 (J_{PC} = 7.0 Hz, C(3)), 126.43, 128.55, 128.89, 139.52, 139.82 (CH_2-Ph), 128.69 (J_{PC} = 12.1 Hz, C(8), C(10)), 130.32, 130.35 (J_{PC} = 11.1 Hz, C(7), C(11)), 131.52 (C(9)), 133.90, 133.94 (J_{PC} = 70.4 Hz, C(6)) (P-Ph); ^{31}P NMR (161.97 MHz, $CDCl_3$): δ 57.6, 57.4; MALDI TOF: *m*/*z* calculated for $C_{17}H_{20}PS$ ($[M + H]^+$): 287.3805; found: 287.3.

3-Benzyl-1-methylphospholane-1-sulfide (**4g**) of the mixture in ratio 2/1.

Calculated for $C_{12}H_{17}PS$: C, 64.26%; H, 7.64%. Found: C, 64.2%; H, 7.5%. ^{1}H NMR (400.13 MHz, $CDCl_3$): δ 1.35 (m, 1H, C(4)H_a), 1.60–1.80 (m, 9H, C(2)H_a, C(4)H_a, C(6)H_6), 1.83–2.30 (m, 9H, C(3)H, C(2)H_b, C(4)H_b, C(5)H_a, C(5)H_b), 2.51 (m, 1H, C(3)H), 2.59–2.75 (m, 4H, C(1')H), 7.05–7.12, 7.12–7.18, 7.19–7.26 (m,10H, P-Ph); ^{13}C NMR (100.62 MHz, $CDCl_3$): δ 22.19, 22.37 (J_{PC} = 48.3 Hz, C(6)), 31.59, 32.04 (J_{PC} = 5.0 Hz, C(4)), 35.12 (J_{PC} =52.3 Hz, C(5)), 39.89, 40.62 (J_{PC}= 53.3 Hz, C(2)), 41.58 (J_{PC} = 12.1 Hz, C(1')), 41.60 (J_{PC}= 13.1 Hz, C(1')), 41.76, 42.15 (J_{PC}= 7.0 Hz, C(3)), 126.33, 128.44, 128.46, 128.73, 128.79, 139.42, 139.63 (CH_2-Ph); ^{31}P NMR (161.97 MHz, $CDCl_3$): δ 55.9; MALDI TOF: *m*/*z* calculated for $C_{12}H_{18}PS$ ($[M + H]^+$): 225.3031; found: 225.4.

3-Benzyl-1-buthylphospholane-1-sulfide (**4h**) of the mixture in ratio 2/1.

Calculated for $C_{15}H_{23}PS$: C, 67.63%; H, 8.70%. Found: C, 67.5%; H, 8.6%. ^{1}H NMR (400.13 MHz, $CDCl_3$): δ 0.85–1.92 (m, 6H, C(9)H), 1.25–1.43 (m, 5H, C(4)H_a, C(8)H), 1.51–1.66 (m, 6H, C(2)H_a, C(7)H), 1.72 (m, 1H, C(4)H_a), 1.76–1.90 (m, 4H, C(6)H), 1.92–2.06 (m, 5H, C(2)H_b, C(4)H_b, C(5)H_a), 2.08–2.15 (m, 2H, C(3)H, C(4)H_b), 2.19 (m, 1H, C(2)H_b), 2.24 (m, 1H, C(5)H_b), 2.50 (m, 1H, C(3)H), 2.60–2.72 (m, 4H, C(1')H), 7.05–7.11, 7.12–7.17, 7.18–7.25 (m, 10H, CH_2-Ph); ^{13}C NMR (100.62 MHz, $CDCl_3$): δ 13.71, 13.74 (C(9)), 23.70 (J_{PC} = 15.1 Hz, C(8)), 24.97, 25.06 (J_{PC} = 3.0 Hz, C(7)), 31.49 (J_{PC} = 4.0 Hz, C(4)), 32.24 (J_{PC} = 3.0 Hz, C(4)), 33.17 (J_{PC} = 51.3 Hz, C(5)), 33.70 (J_{PC} = 50.3 Hz, C(5)), 33.82 (J_{PC} = 46.3 Hz, C(6)), 34.03 (J_{PC} = 46.3 Hz, C(6)), 38.79, 39.31 (J_{PC} = 52.3 Hz, C(2)), 41.46 (J_{PC} = 7.0 Hz, C(3)), 41.67 (J_{PC} = 12.1 Hz, C(1')), 42.36 (J_{PC} = 7.0 Hz, C(3)), 126.32, 128.45, 128.76, 128.81, 139.52, 139.75 (CH_2-Ph); ^{31}P NMR (161.97 MHz, $CDCl_3$): δ 63.2; MALDI TOF: *m*/*z* calculated for $C_{15}H_{24}PS$ ($[M + H]^+$): 267.3908; found: 267.1.

Preparation of bis(1-phenyl(alkyl)phospholanes) (general procedure)

A glass reactor maintained under dry argon at 0 °C was successively charged, with stirring, with toluene (20 mL), Cp_2ZrCl_2 (0.149 g, 0.5 mmol), diene (5 mmol), and $AlEt_3$ (5.5 mL, 30 mmol) The mixture was warmed up to room temperature (~20 °C) and stirred for 12 h. Then the reaction mixture was cooled down to -5 – -10 ^{o}C, alkyl(phenyl)dichlorophosphine (30 mmol) was added dropwise, and the mixture was stirred at room temperature for an additional 30 min. Then the reaction mixture was treated with a saturated aqueous solution of NH_4Cl and the reaction products were extracted with diethyl ether and dried with $MgSO_4$. The solvent was evaporated and

the target phospholanes were isolated by vacuum distillation. All operations were carried out in an argon flow.

3,3'-Ethane-1,2-bis(1-phenylphospholane) (**10a**) of the mixture isomers.

Calculated for $C_{22}H_{28}P_2$: C, 74.6%; H, 8.0%. Found: C, 75.0%; H, 7.9%. ^{1}H NMR (400.13 MHz, $CDCl_3$): δ 1.25–1.60 (m, 8H, C(2)H_a, C(4)H_a, C(6′)H), 1.82–2.03 (m, 4H, C(3)H, C(5)H_a), 2.05–2.32 (m, 5H, C(2)H_b, C(4)H_b, C(5)H_b), 2.34–2.47 (m, H, C(2)H_b), 7.25–7.31, 7.33–7.39, 7.40–7.48 (m, 10H, Ph); ^{13}C NMR (100.62 MHz, $CDCl_3$): δ 25.99 (J_{PC} = 12.1 Hz, C(5)), 26.80 (J_{PC} = 10.1 Hz, C(5)), 32.22 (J_{PC} = 13.1 Hz, C(2)), 33.26 (J_{PC} = 12.1 Hz, C(2)), 33.49 (J_{PC} = 10.8 Hz, C(2)), 33.53 (J_{PC} = 10.9 Hz, C(2)), 34.25, 34.58 (C(4)), 34.97, 35.05, 35.07, 35.24 (C(6′)), 42.35, 43.46, 43.55 (C(3)), 127.28 (J_{PC} = 2.0 Hz, C(9)), 128.29 (J_{PC} = 4.0 Hz, C(9)), 130.42 (J_{PC} = 15.1 Hz, C(8), C(10)), 142.64, 143.09(J_{PC} = 23.1 Hz, C(6)) (P-Ph); ^{31}P NMR (161.97 MHz, $CDCl_3$): δ -13.4, -13.9.

3,3'-Butane-1,4-bis(1-phenylphospholane) (**10b**) of the mixture isomers.

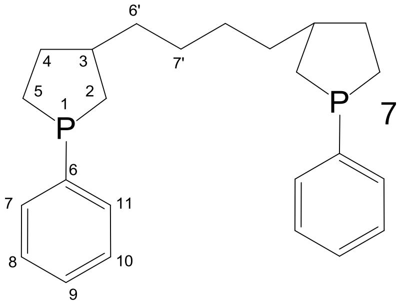

Calculated for $C_{24}H_{32}P_2$: C, 75.37%; H, 8.43%. Found: C, 75.5%; H, 8.5%. ^{1}H NMR (400.13 MHz, $CDCl_3$): δ 1.25–1.42 (m, 10H, C(4)H_a, C(6′)H, C(7′)H), 1.42–1.60 (m, 2H, C(2)H_a), 1.83–2.01 (m, 4H, C(3)H, C(5)H_a), 2.02–2.18 (m, 4H, C(2)H_b, C(4)H_b, C(5)H_b), 2.18–2.30 (m, H, C(5)H_b), 2.33–2.46 (m, H, C(2)H_b), 7.23–7.28, 7.30–7.36, 7.40–7.47 (m, 10H, Ph); ^{13}C NMR (100.62 MHz, $CDCl_3$): δ 26.02 (J_{PC} = 11.1 Hz, C(5)), 26.82 (J_{PC} = 9.1 Hz, C(5)), 28.91, 28.95, 29.02, 29.06 (C(7′)), 32.81 (J_{PC} = 12.1 Hz, C(2)), 33.07 (J_{PC} = 11.1 Hz, C(2)), 33.88 (C(4)), 34.19 (J_{PC} = 3.0 Hz, C(4)), 35.43 (J_{PC} = 3.0 Hz, C(6′)), 35.72 (J_{PC} = 5.0 Hz,

C(6′)), 41.67, 41.69, 41.72, 41.74,42.87 (C(3)), 126.97 (C(9)), 127.94 (J_{PC} = 5.0 Hz, C(9)), 130.06 (J_{PC} = 16.1 Hz, C(8), C(10)), 130.13 (J_{PC} = 16.1 Hz, C(7), C(11)), 142.28, 142.72 (J_{PC} = 23.1 Hz, C(6)) (P-Ph); ^{31}P NMR (161.97 MHz, $CDCl_3$): δ -13.6, -13.2.

3,3'-Hexane-1,6-bis(1-phenylphospholane) (**10c**)of the mixture isomers.

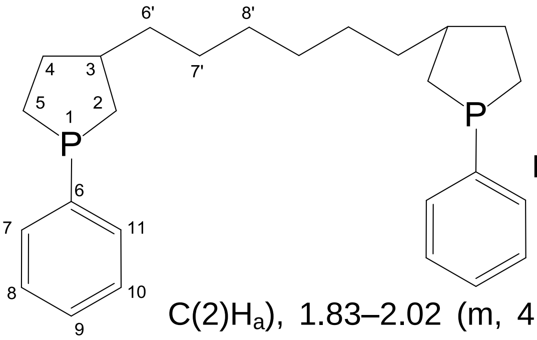

Calculated for $C_{26}H_{36}P_2$: C, 76.07%; H, 8.84%. Found: C, 76.2%; H, 8.9%. ^{1}H NMR (400.13 MHz, $CDCl_3$,): δ 1.27–1.47 (m, 14H, C(4)H_a, C(6′)H, C(7′)H, C(8′)H), 1.48–1.64 (m, 2H, C(2)H_a), 1.83–2.02 (m, 4H, C(3)H, C(5)H_a), 2.03–2.21 (m, 4H, C(2)H_b, C(4)H_b, C(5)H_b), 2.21–2.33 (m, H, C(5)H_b), 2.36–2.50 (m, 1H, C(2)H_b), 7.25–7.30, 7.33–7.38, 7.40–7.41, 7.43–7.49 (m, 10H, Ph); ^{13}C NMR (100.62 MHz, $CDCl_3$): δ 27.10, 27.13 (J_{PC} = 11.1 Hz, C(5)), 27.89, 27.90 (J_{PC} = 9.1 Hz, C(5)), 29.78, 29.84, 29.89, 29.95 (C(7′)), 30.84, 30.91 (C(8′)), 34.24 (J_{PC} = 12.1 Hz, C(2)), 34.49 (J_{PC} = 11.1 Hz, C(2)), 35.28 (J_{PC} = 3.0 Hz, C(4)), 35.59 (J_{PC} = 4.0 Hz, C(4)), 36.89, 36.92, 36.96, 37.14, 37.18, 37.23 (C(6′)), 43.15, 43.17 (J_{PC} = 4.0 Hz, C(3)), 44.31, 44.33, 44.35 (C(3)), 128.31 (C(9)), 129.29 (J_{PC} = 5.0 Hz, C(9)), 131.45 (J_{PC} = 15.1 Hz, C(8), C(10)), 131.51 (J_{PC} = 16.1 Hz, C(7), C(11)), 143.65 (J_{PC} = 22.1 Hz, C(6)), 143.95 (J_{PC} = 23.1 Hz, C(6)) (P-Ph); ^{31}P NMR (161.97 MHz, $CDCl_3$): δ -13.6, -13.2.

3,3'-Ethane-1,2-bis(1-butylphospholane) (**10d**)of the mixture isomers.

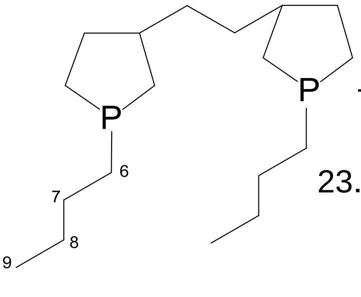

Calculated for $C_{18}H_{36}P_2$: C, 68.76%; H, 11.54%. Found: C, 76.2%; H, 8.9%. ^{31}P NMR (161.97 MHz, $CDCl_3$): δ -23.3, -23.8.

3,3'-Butane-1,4-bis(1-methylphospholane) (**10e**)of the mixture isomers.

Calculated for $C_{14}H_{28}P_2$: C, 65.09%; H, 10.93%. Found: C, 65.1%; H, 11.0%. ^{1}H NMR (400.13 MHz, $CDCl_3$): δ 1.03–1.09 (m, 6H, C(6)H), 1.19-1.49 (m, 16H, C(7')H, C(2)H, C(4)H, C(6')H), 1.51-1.71 (m, 4H, C(5)H), 1.74-1.87, 2.02-2.15 (m, 2H, C(3)H; ^{13}C NMR (100.62 MHz, $CDCl_3$): δ 13.89, 14.06, 14.17, 14.22 (C(6)), 26.88, 26.98, 27.04, 27.11 (C(5)), 29.24, 29.32, 29.57, 29.78 (C(7')), 31.31, 31.66, 31.96, 32.00 (C(2)), 33.34, 33.37, 33.89, 33.97 (C(4)), 35.80, 35.82, 36.74, 36.78 (C(6')), 41.18, 41.23, 43.85, 43.87 (C(3)); ^{31}P NMR (161.97 MHz, $CDCl_3$): δ -34.9, -35.0, -35.2.

Preparation of bis(1-phenyl(alkyl)phospholanes)-1-oxides (general procedure)

A 30% solution of hydrogen peroxide (0.7 mL, 6 mmol) was slowly added dropwise with vigorous stirring to a solution of bis(1-phenyl(alkyl)phospholanes) (5 mmol), synthesized as described above, in chloroform (10 mL) and the mixture was stirred for 1 h. Then the reaction mixture was washed with water (3 × 5 mL) and the organic layer was dried with $MgSO_4$. The solvent was evaporated and the residue was chromatographed on silica gel (hexane/ethyl acetate/methanol 5:3:1).

3,3'-Ethane-1,2-bis(1-phenylphospholane)-1,1'-dioxide (**11a**)of the mixture isomers.

Calculated for $C_{22}H_{28}P_2O_2$: C, 68.38%; H, 7.30%. Found: C, 68.6%; H, 7.4%. ^{1}H NMR (400.13 MHz, $CDCl_3$): δ 1.29–1.65 (m, 6H, C(2)H_a, C(4)H_a, C(6')H), 1.65–1.73 (m, 1H, C(2)H_a), 1.73–1.84 (m, 1H, C(4)H_a), 1.85–1.95 (m, 1H, C(5)H_a), 1.96–2.08 (m, 2H, C(3)H, C(5)H_a), 2.08–2.41 (m, 7H, C(2)H_b, C(3)H, C(4)H_b, C(5)H_b), 7.43–7.52, 7.66–7.73 (m, 10H, Ph); ^{13}C NMR (100.62 MHz, $CDCl_3$): δ 29.36, 29.39 (J_{PC} = 67.4 Hz, C(5)), 29.44, 29.46 (J_{PC} = 68.4 Hz, C(5)), 30.44 (J_{PC} = 66.4 Hz, C(5)), 30.84,

30.91, 30.95, 31.01, 32.13, 32.19, 32.26, 32.30 (C(4)), 33.86, 33.99, 34.06, 34.19, 34.25, 34.31, 34.38, 34.45 (C(6′)), 35.91, 35.94 (J_{PC} = 67.4 Hz, C(2)), 35.97, 36.00 ($^1J_{PC}$ = 66.4 Hz, C(2)), 36.29, 36.34, 36.39, 36.45 (J_{PC} = 68.4 Hz, C(2)), 38.72, 38.76, 38.80, 38.86, 38.96, 39.05, 39.10, 39.18 (C(3)), 39.75, 39.83, 39.91, 40.09, 40.18 (C(3)), 128.36 (J_{PC} = 11.1 Hz), 129.48 (J_{PC} = 9.0 Hz), 131.37 (J_{PC} = 5.0 Hz), 133.87 (J_{PC} = 90.5 Hz), 133.95 (J_{PC} = 89.5 Hz) (Ph); ^{31}P NMR (161.97 MHz, $CDCl_3$): δ 59.2; MALDI TOF: *m/z* calculated for $C_{22}H_{29}P_2O_2$ ([M + H]$^+$): 387.404; found: 387.2.

3,3'-Butane-1,4-bis(1-phenylphospholane)-1,1'-dioxide (**11b**) of the mixture isomers. Calculated for $C_{24}H_{32}P_2O_2$: C, 69.55%; H, 7.78%. Found: C, 69.6%; H, 7.9%. ^{1}H NMR (400.13 MHz, $CDCl_3$): δ 1.20–1.48 (m, 10H, C(2)H_a, C(4)H_a, C(6')H, C(7')H), 1.52–1.63 (m, 1H, C(2)H_a), 1.63–1.75 (m, 1H, C(4)H_a), 1.77–1.97 (m, 3H, C(3)H, C(5)H_a), 1.97–2.32 (m, 7H, C(2)H_b, C(3)H, C(4)H_b, C(5)H_b), 7.34–7.43, 7.58–7.66 (m, 10H, Ph); ^{13}C NMR (100.62 MHz, $CDCl_3$): δ 27.81, 27.87, 27.92, 28.01 (C(7′)), 29.43, 30.46 (J_{PC} = 66.4 Hz, C(5)), 30.79, 30.83, 30.86, 30.89, 30.92 (C(4)), 35.87, 35.89 (J_{PC} = 56.3 Hz, C(2)), 36.30, 36.27, 36.23, 36.18, 36.10, 35.99 (C(6′)), 36.37, 35.41 (J_{PC} = 56.3 Hz, C(2)), 40.02, 39.99, 39.96, 39.91, 38.74, 38.68, 38.66, 38.62 (C(3)), 128.47 (J_{PC} = 12.1 Hz), 129.74 (J_{PC} = 10.1 Hz), 131.41 (J_{PC} = 2.0 Hz), 134.47 (J_{PC} = 90.5 Hz), 134.59 (J_{PC} = 89.5 Hz) (Ph); ^{31}P NMR (161.97 MHz, $CDCl_3$): δ 59.4; MALDI TOF: *m/z* calculated for $C_{24}H_{31}P_2O_2$ ([M - H]$^-$): 413.4572; found: 413.4.

3,3'-Hexane-1,6-bis(1-phenylphospholane)-1,1'-dioxide (**11c**) of the mixture isomers. Calculated for $C_{26}H_{36}P_2O_2$: C, 70.57%; H, 8.20%. Found: C, 70.7%; H, 8.3%. ^{1}H NMR (400.13 MHz, $CDCl_3$): δ 1.02–1.45 (m, 14H, C(2)H_a, C(4)H_a, C(6')H, C(7')H, C(8')H), 1.47–1.60 (m, 1H, C(2)H_a), 1.60–1.68 (m, 1H, C(4)H_a), 1.71–1.80 (m, 1H,

C(5)H_a), 1.82–2.26 (m, 9H, C(2)H_b, C(3)H, C(4)H_b, C(5)H_b), 7.23–7.46, 7.52–7.70 (m, 10H, Ph); ^{13}C NMR (100.62 MHz, $CDCl_3$): δ 28.68, 28.76 (C(7′)), 30.41 (J_{PC} = 67.4 Hz, C(5)), 30.56, 30.75 (C(8′)), 31.46 (J_{PC} = 67.4 Hz, C(5)), 31.82, 31.88, 33.09, 33.13 (C(4)), 36.80 (J_{PC} = 68.4 Hz, C(2)), 37.14 (C(6′)), 37.35 (J_{PC} = 67.4 Hz, C(2)), 37.43 (C(6′)), 39.72, 39.80 (C(3)), 40.96, 41.04 (C(3)), 129.49 (J_{PC} = 11.1 Hz), 130.70 (J_{PC} = 9.0 Hz), 132.46, 135.37, 135.47 (J_{PC} = 89.5 Hz) (Ph); ^{31}P NMR (161.97 MHz, $CDCl_3$): δ 59.2; MALDI TOF: *m*/*z* calculated for $C_{26}H_{35}P_2O_2$([M - H]$^+$): 441.510; found: 441.1.

General procedure for preparation of bis(1-phenyl(alkyl)phospholanes)-1-sulfides

Reactions were performed under argon. Sulfur (0.13 g, 4 mmol) was added with cooling to a solution of bis(1-phenyl(alkyl)phospholanes) (4 mmol) (prepared as described above) in 10 mL toluene, and the mixture was stirred for 4 h. After filtration through a thin layer of silica gel the solvent was evaporated to give colorless oil.

3,3'-Ethane-1,2-bis(1-phenylphospholane)-1,1'-disulfide (**12a**) of the mixture isomers. Calculated for $C_{22}H_{28}P_2S_2$: C, 63.13%; H, 6.74%. Found: C, 63.3%; H, 6.8%. ^{1}H NMR (400.13 MHz, $CDCl_3$): δ 1.49–1.71 (m, 5H, C(4)H_a, C(6')H), 1.87–2.05 (m, 3H, C(2)H_a, C(4)H_a), 2.14–2.53 (m, 8H, C(2)H_b, C(3)H, C(4)H_b, C(5)H_a, C(5)H_b), 2.58–2.72 (m, 2H, C(2)H_b, C(5)H_b), 7.47–7.56, 7.85–7.93 (m, 10H, Ph); ^{13}C NMR (100.62 MHz, $CDCl_3$): δ 32.14, 33.93 (C(4)), 34.16, 34.23, 34.37, 34.49, 35.55 (C(6′)), 35.74 (J_{PC} = 54.3 Hz, C(5)), 36.61 (J_{PC} = 53.3 Hz, C(5)), 39.50, 39.58, 39.66, 39.76, 39.80, 39.84, 39.89, 39.97, 41.36, 41.40, 41.44, 41.46, 41.64, 41.68, 41.71, 41.74 (C(3)), 41.82, 41.98 (J_{PC} = 54.4 Hz, C(2)), 42.47, 42.60 ($^1J_{PC}$ = 55.4 Hz, C(2)), 128.36 (J_{PC} =

12.1 Hz), 129.96 (J_{PC} = 10.1 Hz), 131.19 (J = 3.0 Hz), 133.47,133.53 (J_{PC} = 70.4 Hz) (Ph); ^{31}P NMR (161.97 MHz, $CDCl_3$): δ 57.5.

3,3'-Butane-1,4-bis(1-phenylphospholane)-1,1'-disulfide (**12b**) of the mixture isomers. Calculated for $C_{24}H_{32}P_2S_2$: C, 64.55%; H, 7.22%. Found: C, 64.6%; H, 7.3%. ^{1}H NMR (400.13 MHz, $CDCl_3$): δ 1.29–1.57 (m, 9H, C(4)H_a, C(6')H, C(7')H), 1.78–1.98 (m, 3H, C(2)H_a, C(4)H_a), 2.06–2.45 (m, 8H, C(2)H_b, C(3)H, C(4)H_b, C(5)H_a, C(5)H_b), 2.51–2.65 (m, 2H, C(2)H_b, C(5)H_b), 7.41–7.49, 7.80–7.88 (m, 10H, Ph); ^{13}C NMR (100.62 MHz, $CDCl_3$): δ 28.02 (C(7′)), 32.14 (J_{PC} = 4.0 Hz, C(4)), 33.89 (J_{PC} = 2.0 Hz, C(4)), 35.47, 35.56, 35.58, 35.67, 35.71, 35.77, 35.83 (C(6′)), 35.83, 36.71 (J_{PC} = 53.3 Hz, C(5)), 39.90, 39.96, 40.02, 41.87 (C(3)), 42.23 (J_{PC} = 53.3 Hz, C(2)), 42.58 (J_{PC} = 54.3 Hz, C(2)), 128.30 (J_{PC} = 12.1 Hz), 129.93 (J_{PC} = 10.1 Hz), 131.11 (J = 3.0 Hz), 133.56, 133.62 (J_{PC} = 70.4 Hz) (Ph); ^{31}P NMR (161.97 MHz, $CDCl_3$): δ 57.6; MALDI TOF: m/z calculated for $C_{24}H_{33}P_2S_2$ ($[M + H]^+$): 447.5904; found: 447.1.

3,3'-Hexane-1,6-bis(1-phenylphospholane)-1,1'-disulfide (**12c**) of the mixture isomers.

Calculated for $C_{26}H_{36}P_2S_2$: C, 65.79%; H, 7.64%. Found: C, 65.7%; H, 7.5%. ^{1}H NMR (400.13 MHz, $CDCl_3$): δ 1.23–1.36 (m, 8H, C(7')H, C(8')H), 1.38–1.55 (m, 5H, C(4)H_a, C(6')H), 1.76–1.99 (m, 3H, C(2)H_a, C(4)H_a), 2.07–2.45 (m, 8H, C(2)H_b, C(3)H, C(4)H_b, C(5)H_a, C(5)H_b), 2.50–2.64 (m, 2H, C(2)H_b, C(5)H_b), 7.40–7.46, 7.79–7.88 (m, 10H, Ph); ^{13}C NMR (100.62 MHz, $CDCl_3$): δ 28.88 (C(7′)), 29.47, 29.50, 29.53 (C(8′)), 32.15 (J_{PC} = 6.0 Hz, C(4)), 36.91 (J_{PC} = 2.0 Hz, C(4)), 35.57, 35.70, 35.88, 35.99 (C(6′)), 35.88, 36.75 (J_{PC} = 53.3 Hz, C(5)), 40.00 (J_{PC} = 7.0 Hz, C(3)), 41.98 (C(3)), 42.23 (J_{PC} = 55.3 Hz, C(2)), 42.64 (J_{PC} = 54.3 Hz, C(2)), 128.61 (J_{PC} =

11.1 Hz), 130.28 (J_{PC} = 11.1 Hz), 131.41, 133.96, 134.04 (J_{PC} = 70.4 Hz) (P-Ph); ^{31}P NMR (161.97 MHz, $CDCl_3$): δ 57.7; MALDI TOF: *m*/*z* calculated for $C_{26}H_{37}P_2S_2$ ([M + H]$^+$): 475.6436; found: 475.2.

Synthesis of a Mo Complex 13b, 14g

Reactions were performed under argon using standard Schlenk techniques. A mixture of $Mo(CO)_6$ (0.79 g, 3 mmol) and 3-hexyl(benzyl)-1-phenyl(methyl)phospholane **2b**, **2g** (0.75 g, (0.58 g), 3 mmol) was stirred under reflux in 15 ml THF for 24 h. The colorless solution became dark-brown during this time. The solvent was removed under vacuum and the residue was chromatographed on silica gel (hexane) gave **13b**, **14g** as dark green thick liquid in 36%, 23% yield (0.27g, 0.13 g).

(3-Hexyl-1-phenylphospholane)pentacarbonylmolybdenum (**13b**) of the mixture in ratio 1/1.

Calculated for $C_{21}H_{25}MoO_5P$: C, 52.08%; H, 5.20%. Found: C, 52.2%; H, 5.3%. ^{1}H NMR (400.13 MHz, $CDCl_3$): δ 0.20-1.01 (s, 6H, C(6′)H), 1.30-1.56 (s, 20H, C(5′)H, C(3′)H, C(2′)H, C(4′)H, C(1′)H), 1.58-1.64 (m, 2H, C(4)H), 1.72-1.82 (m, 1H, C(2)H_a), 1.93-2.02 (m, 1H, C(3)H), 2.06-2.27 (m, 4H, C(4)H_b, C(2)H_b, C(3)H), 2.41-2.57 (m, 3H, C(5)H_a, C(5)H_b), 2.58-2.80 (m, 3H, C(5)H_b, C(2)H_a, C(2)H_b), 7.23-7.26, 7.31-7.33, 7.37-7.44, 7.45-7.56 (m, 10H, Ph); ^{13}C NMR (100.62 MHz, $CDCl_3$): δ 14.10 (C(6')), 22.66, 22.72 (C(5')), 28.39, 28.42 (C(3')), 29.37, 29.44 (C(2')), 31.45 (J_{PC} = 22.1 Hz, C(5)), 31.81 (C(4')), 32.16 (J_{PC} = 24.1 Hz, C(5)), 33.42, 34.13 (C(4)), 35.70 (J_{PC} = 8.0 Hz, C(1')), 35.75 (J_{PC} = 9.1 Hz, C(1')), 38.19 (C(2)), 38.41 (J_{PC} = 3.0 Hz, C(2)), 41.78 (J_{PC} = 1.0 Hz, C(3)), 42.30 (C(3)), 128.81, 128.88 (J_{PC} = 10.1 Hz),

128.95 (J_{PC} = 11.1 Hz), 129.08, 139.67 (J_{PC} = 27.2 Hz, C(6)), 140.24 (J_{PC} = 28.2 Hz, C(6)) (P-Ph), 205.75 (J_{PC} = 9.1 Hz, CO_{cis}), 205.77 (J_{PC} = 9.1 Hz, CO_{cis}), 210.51 (J_{PC} = 22.1 Hz, CO_{trans}), 210.57 (J_{PC} = 21.1 Hz, CO_{trans}); ^{31}P NMR (161.97 MHz, $CDCl_3$): δ 26.0, 25.2.

(3-Benzyl-1-methylphospholane)pentacarbonylmolybdenum (**14g**) of the mixture in ratio 1/1.

Calculated for $C_{17}H_{17}MoO_5P$: C, 47.68%; H, 4.00%. Found: C, 47.7%; H, 3.9%. ^{1}H NMR (400.13 MHz, $CDCl_3$): δ 1.45–1.58 (m, 6H, C(6)), 1.86–2.00 (m, 4H, C(5)H_a, C(4)H_a), 2.02–2.18 (m, 6H, C(5)H_b, C(4)H_b, C(1′)H), 2.23–2.43 (m, 4H, C(1′)H, C(3)H), 2.70–2.79 (m, 2H, C(2)H_a), 2.82–2.90 (m, 2H, C(2)H_b), 7.12–7.18, 7.20–7.26, 7.27–7.35 (m, 10H, Ph); ^{13}C NMR (100.62 MHz, $CDCl_3$): δ 19.67 (J_{PC} = 20.1 Hz C(6)), 19.87 (J_{PC} = 19.1 Hz C(6)), 31.82 (J_{PC} = 23.1 Hz, C(5)), 32.44 (J_{PC} = 24.1 Hz, C(5)), 32.98, 33.60 (C(4)), 38.41, 38.60(J_{PC} = 24.1 Hz, C(1')), 41.68 (J_{PC} = 8.0 Hz, C(2)), 41.92 (J_{PC} = 9.0 Hz, C(2)), 43.42, 44.13 (C(3)), 126.40, 126.42, 128.51, 128.57, 128.65, 139.61, 140.02 (CH_2-Ph), 205.69 (J_{PC} = 9.1 Hz, CO_{cis}), 205.72 (J_{PC} = 9.1 Hz, CO_{cis}), 209.17 (CO_{trans}), 209.39 (CO_{trans}); ^{31}P NMR (161.97 MHz, $CDCl_3$): δ 10.9.

Synthesis of a Mo Complex 15a, 16c.

Reactions were performed under argon using standard Schlenk techniques. A mixture of $Mo(CO)_6$ (0.79 g, 3 mmol) and 3,3'-ethane(hexane)-1,2(1,6)-bis(1-phenylphospholane) **10a, 10c**(1.1 g, (1.2 g), 3 mmol) was stirred under reflux in 15mL THF for 24 h. The colorless solution became brown during this time. The solvent was removed under vacuum and the crude product was purified by silica gel

column chromatography (hexane) gave **15a, 16c** as dark brown thick liquid with 24%, 26% yield (0.26 g, 0.31 g).

3,3'-Ethane-1,2-bis(1-phenylphospholane)-1,1'-di(pentacarbonylmolybdenum) (**15a**) of the mixture isomers.

Calculated for $C_{32}H_{28}Mo_2O_{10}P_2$: C, 46.51%; H, 3.42%. Found: C, 46.5%; H, 3.5%. ^1H NMR (400.13 MHz, $CDCl_3$): δ 1.28-1.39 (m, 2H, C(4)H_a, C(4)H_b), 1.40-1.60 (m, 2H, C(4)H_a, C(4)H_b), 1.64-1.73 (m, 1H, C(2)H_a), 1.84-1.94 (m, 1H, C(3)H), 1.98-2.22 (m, 6H, C(6′), C(2)H_b, C(3)H), 2.35-2.58 (m, 3H, C(5)H_a, C(5)H_b, C(2)H_a), 2.60-2.70 (m, 3H, C(5)H_a, C(5)H_b, C(2)H_b), 7.34-7.56 (m, 10H, P-Ph); ^{13}C NMR (100.62 MHz, $CDCl_3$): δ 31.29, 31.35 (J_{PC} = 23.1 Hz, C(5)), 32.01 (J_{PC} = 23.1 Hz, C(5)), 32.05 (J_{PC} = 24.1 Hz, C(5)), 33.35, 33.40, 34.13, 34.17, 34.22 (C(4)), 34.37, 34.44, 34.52, 34.59 (C(6')), 38.31, 38.35, 38.37 (J_{PC} = 23.1 Hz, C(2)), 38.50, 38.54 (C(2)), 41.74, 41.82, 41.91, 42.36, 42.42 (C(3)), 128.84, 128.94, 129.03, 129.19, 139.44, 140.11 (J_{PC} = 28.2 Hz, C(6)) (P-Ph), 205.74, 205.83 (CO_{cis}), 210.49, 210.55 (J_{PC} = 22.1 Hz, CO_{trans}); ^{31}P NMR (161.97 MHz, $CDCl_3$): δ 25.3, 26.3.

3,3'-Hexane-1,6-bis(1-phenylphospholane)-1,1'-di(pentacarbonylmolybdenum) (**16c**) of the mixture isomers.

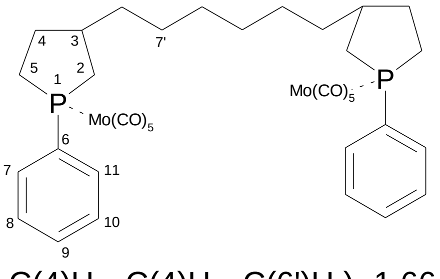

Calculated for $C_{36}H_{36}Mo_2O_{10}P_2$: C, 49.00%; H, 4.11%. Found: C, 49.1%; H, 4.1%. ^1H NMR (400.13 MHz, $CDCl_3$): δ 1.06-1.18 (m, 2H, C(4)H_a, C(4)H_b), 1.20-1.39 (s, 8H, C(7'), C(8')), 1.43-1.52 (m, 4H, C(4)H_a, C(4)H_b, C(6')H,), 1.66-1.77 (m, 1H, C(2)H_a), 1.88-2.00 (m, 1H, C(3)H), 2.03-2.20 (m, 2H, C(2)H_b, C(3)H), 2.35-2.49 (m, 4H, C(5)H_a, C(5)H_b, C(6')H), 2.51-2.74

(m, 4H, C(5)H_a, C(5)H_b, C(2)H_a, C(2)H_b), 7.30-7.60 (m, 10H, P-Ph); ^{13}C NMR (100.62 MHz, $CDCl_3$): δ 29.57, 29.71, 29.77, 29.81 (C(7')), 29.90 (C(8')), 31.43 (J_{PC} = 22.1 Hz, C(5)), 32.14 (J_{PC} = 23.1 Hz, C(5)), 35.63, 35.67, 35.71, 35.76 (C(4), C(6')), 38.26 (J_{PC}= 20.1 Hz, C(2)), 38.32 (J_{PC}= 16.1 Hz, C(2)), 41.74, 42.26 (C(3)), 128.85, 128.93, 129.03, 130.04, 131.56, 131.66, 139.56, 139.84, 140.14, 140.41 (P-Ph), 205.49 (J_{PC} = 9.1 Hz, CO_{cis}), 205.79 (J_{PC} = 9.4 Hz, CO_{cis}), 210.56 (J_{PC} = 21.8 Hz, CO_{trans}); ^{31}P NMR (161.97 MHz, $CDCl_3$): δ 25.2, 26.0.

References

[1] D'yakonov, V. A.; Makhamatkhanova, A. L.; Tyumkina, T. V.; Dzhemilev, U. M. *Rus. Chem. Bull.* **2012**, *61*, 1556–1559.
