# Supplementary material for: Aluminacyclopentanes in the synthesis of 3-substituted phospholanes and α,ω-bisphospholanes
Source: Beilstein J Org Chem. 2016 Mar 2;12:406–12. doi: 10.3762/bjoc.12.43 (PMC4902032; doi:10.3762/bjoc.12.43)

Supporting Information

for

Aluminacyclopentanes in the synthesis of 3-substituted phospholanes and α,ω-bisphospholanes

Vladimir A. D’yakonov*, Alevtina L. Makhamatkhanova, Rina A. Agliullina, Leisan K. Dilmukhametova, Tat’yana V. Tyumkina, and Usein M. Dzhemilev

Address: Institute of Petrochemistry and Catalysis of Russian Academy of Sciences, Prospekt Oktyabrya, Ufa 450075, Russian Federation

Email: Vladimir D’yakonov* - DyakonovVA@gmail.com

*Corresponding author

NMR spectra

^{1}H NMR ($CDCl_3$): **2b**

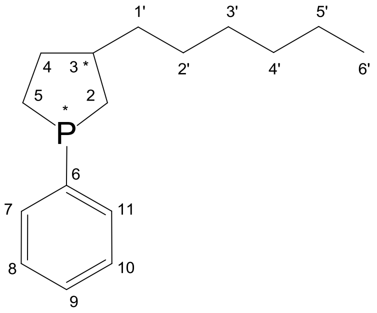

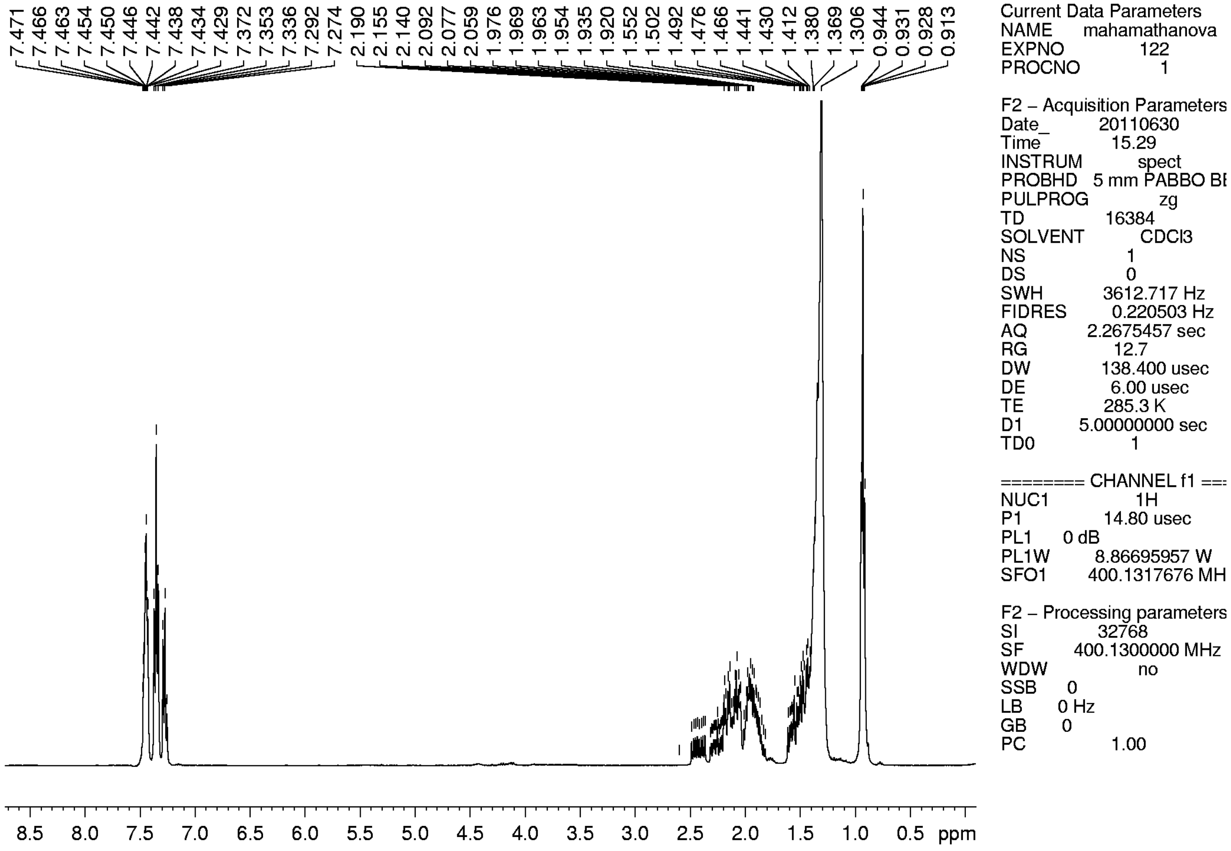

^{13}C NMR ($CDCl_3$): **2b**

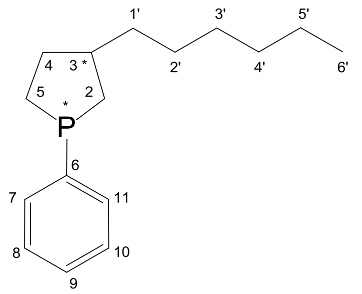

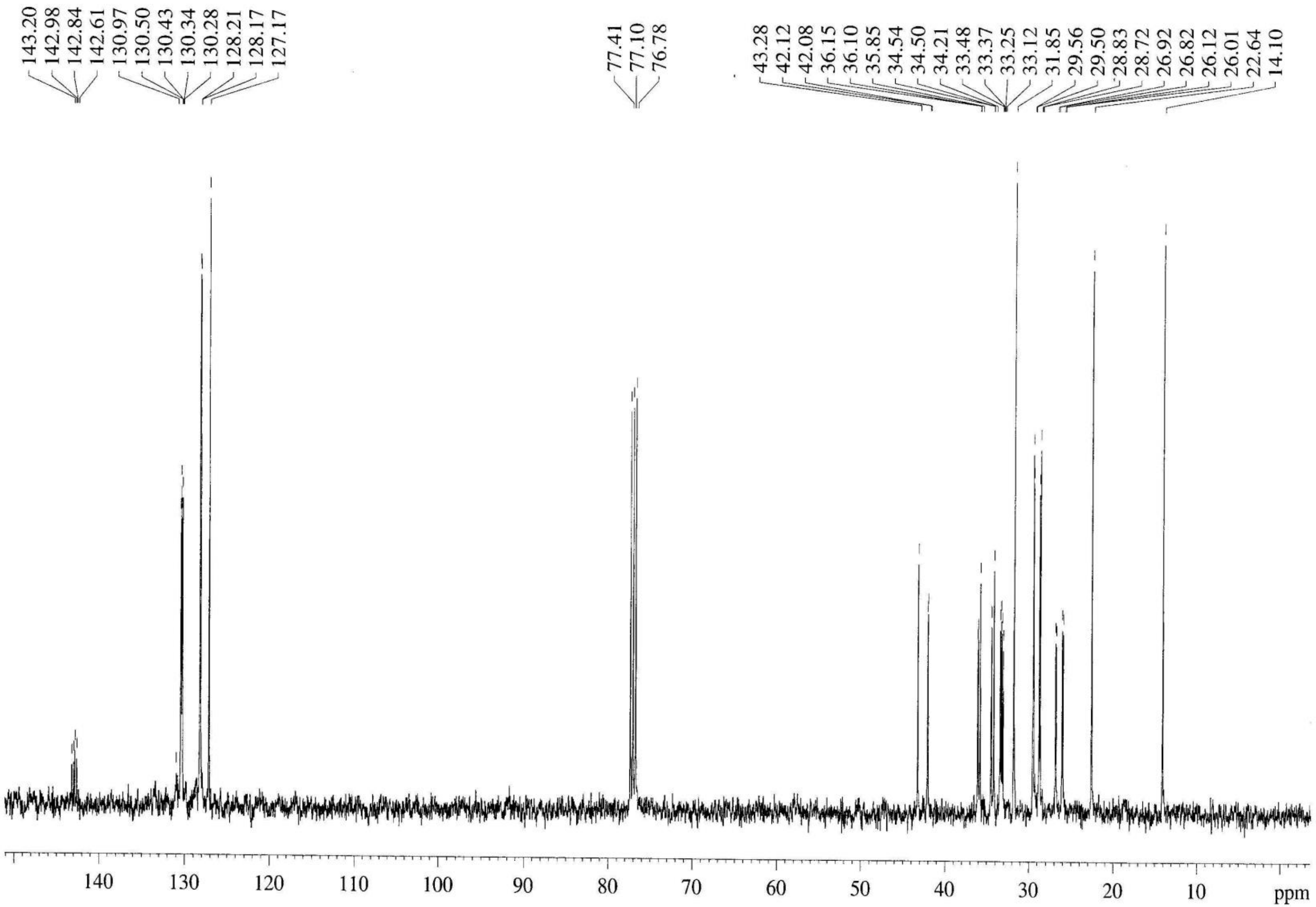

^{31}P NMR ($CDCl_3$): **2b**

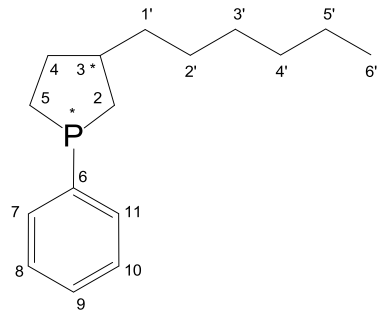

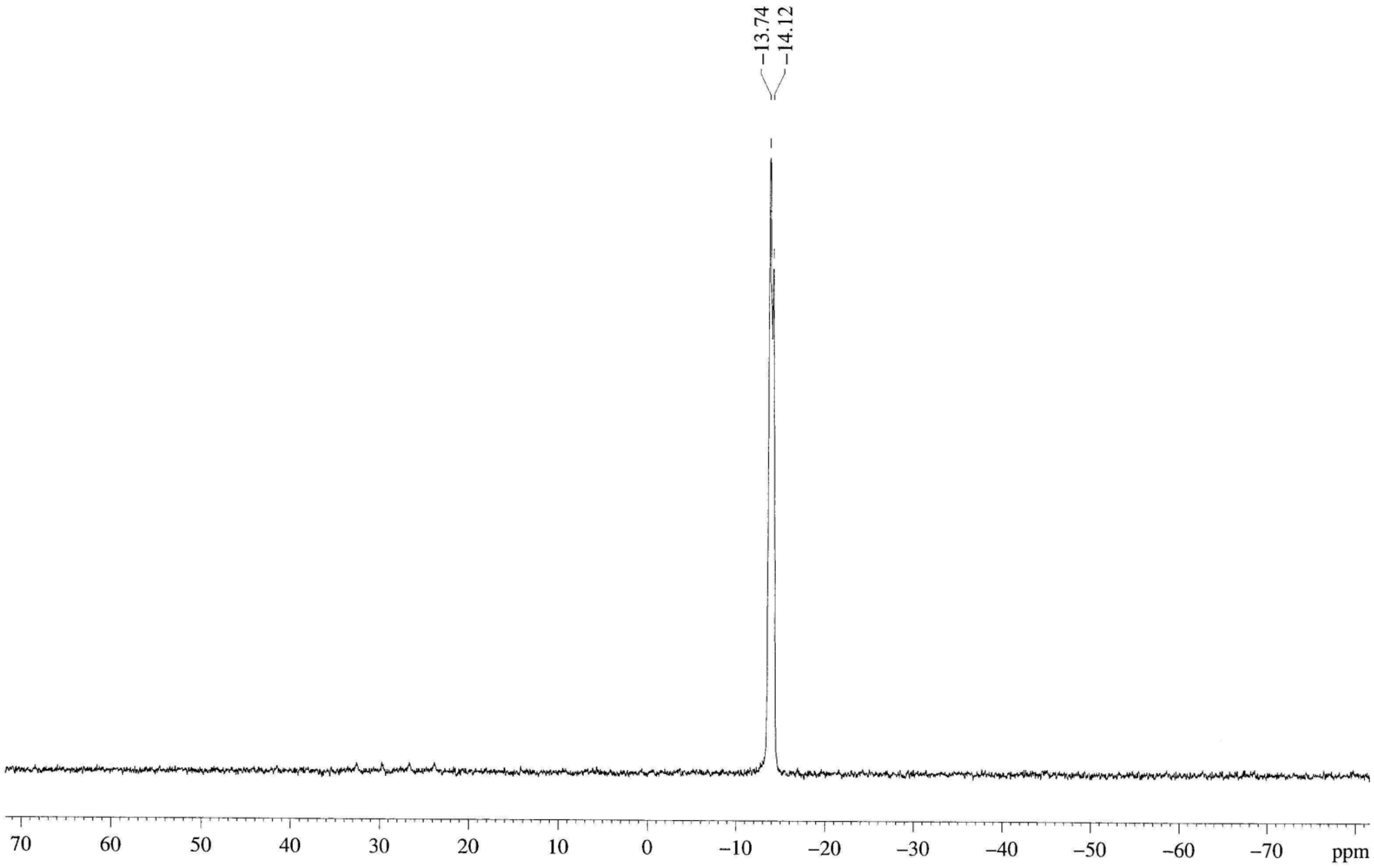

^1H NMR ($CDCl_3$): **2c**

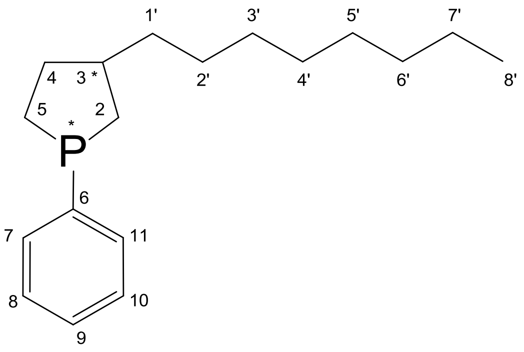

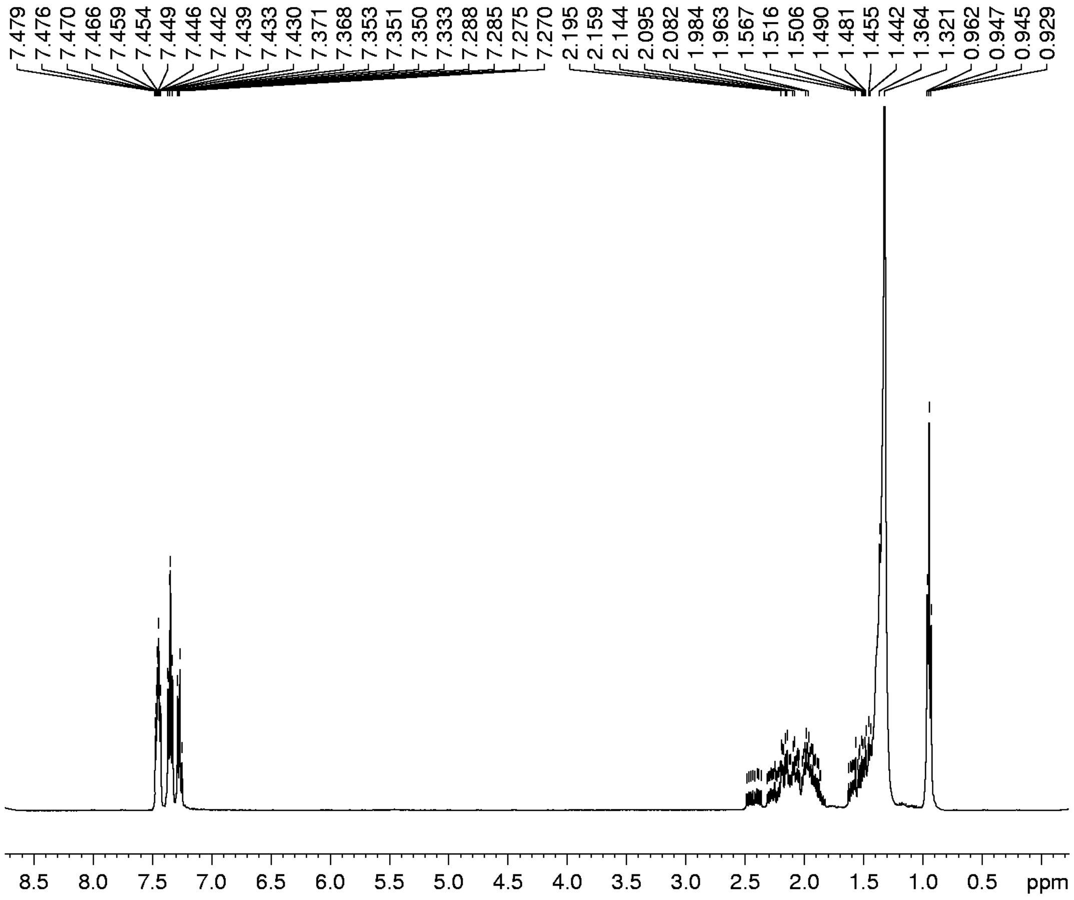

Current Data Parameters
NAME MRC–12
EXPNO 1
PROCNO 1

F2 – Acquisition Parameters
Date_ 20121207
Time 13.27
INSTRUM spect
PROBHD 5 mm PABBO BE
PULPROG zg30
TD 16384
SOLVENT CDCl3
NS 1
DS 0
SWH 3591.954 Hz
FIDRES 0.219235 Hz
AQ 2.2806528 sec
RG 22.6
DW 139.200 usec
DE 6.00 usec
TE 297.8 K
D1 1.00000000 sec
TD0 1

======== CHANNEL f1 ===
NUC1 1H
P1 14.80 usec
PL1 0 dB
PL1W 8.86695957 W
SFO1 400.1317034 MHz

F2 – Processing parameters
SI 32768
SF 400.1300000 MHz
WDW no
SSB 0
LB 0 Hz
GB 0
PC 1.00

^{13}C NMR ($CDCl_3$): **2c**

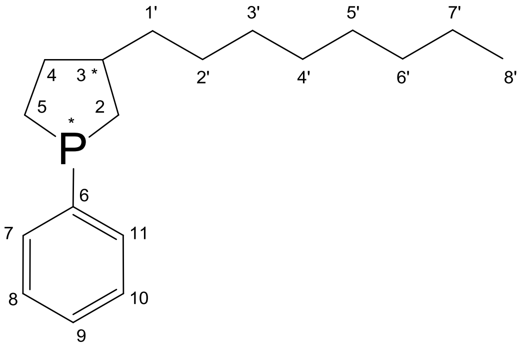

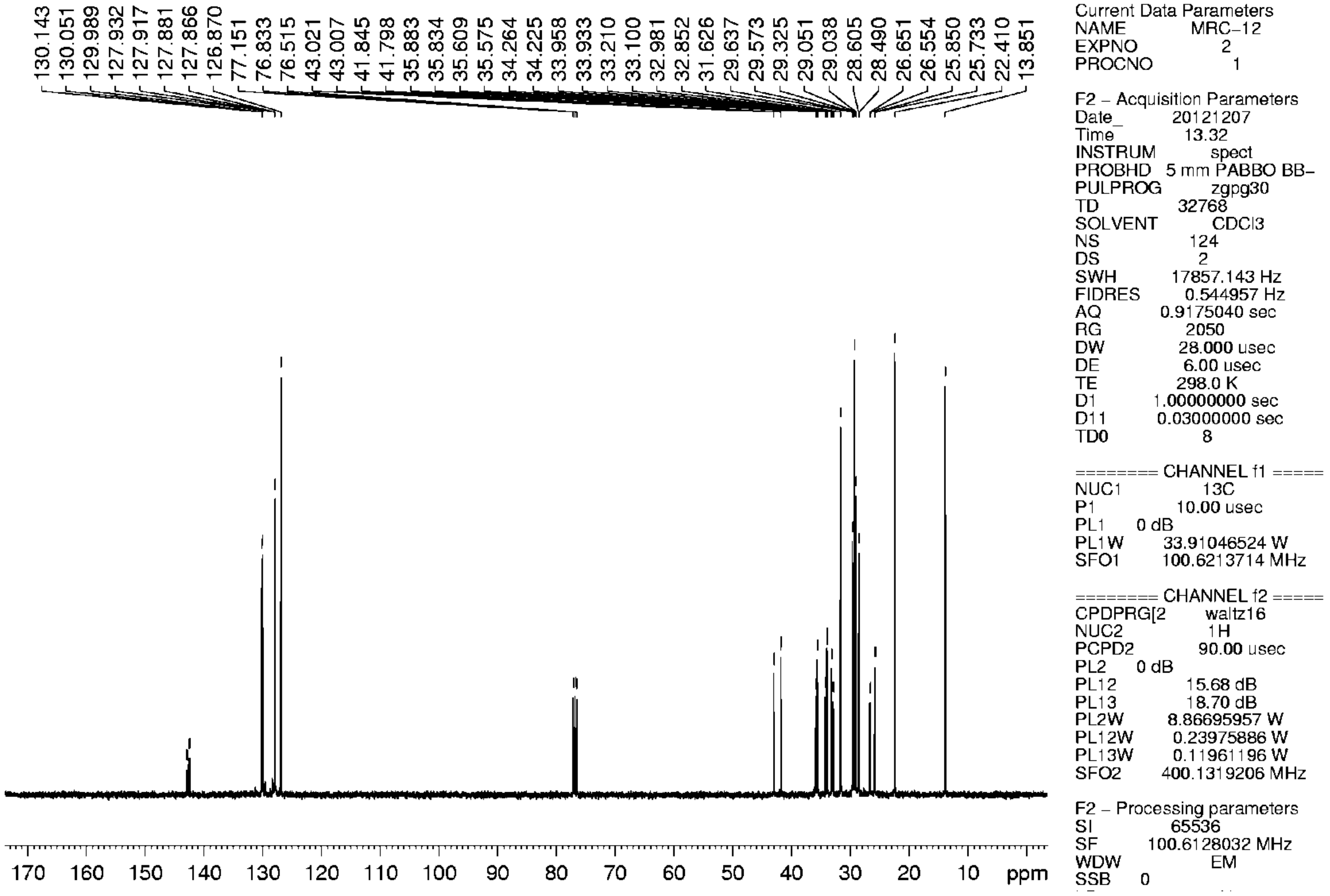

^{31}P NMR ($CDCl_3$): **2c**

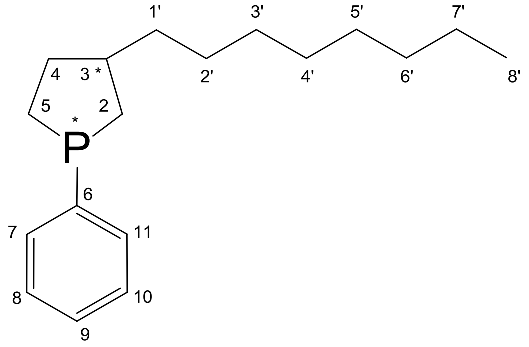

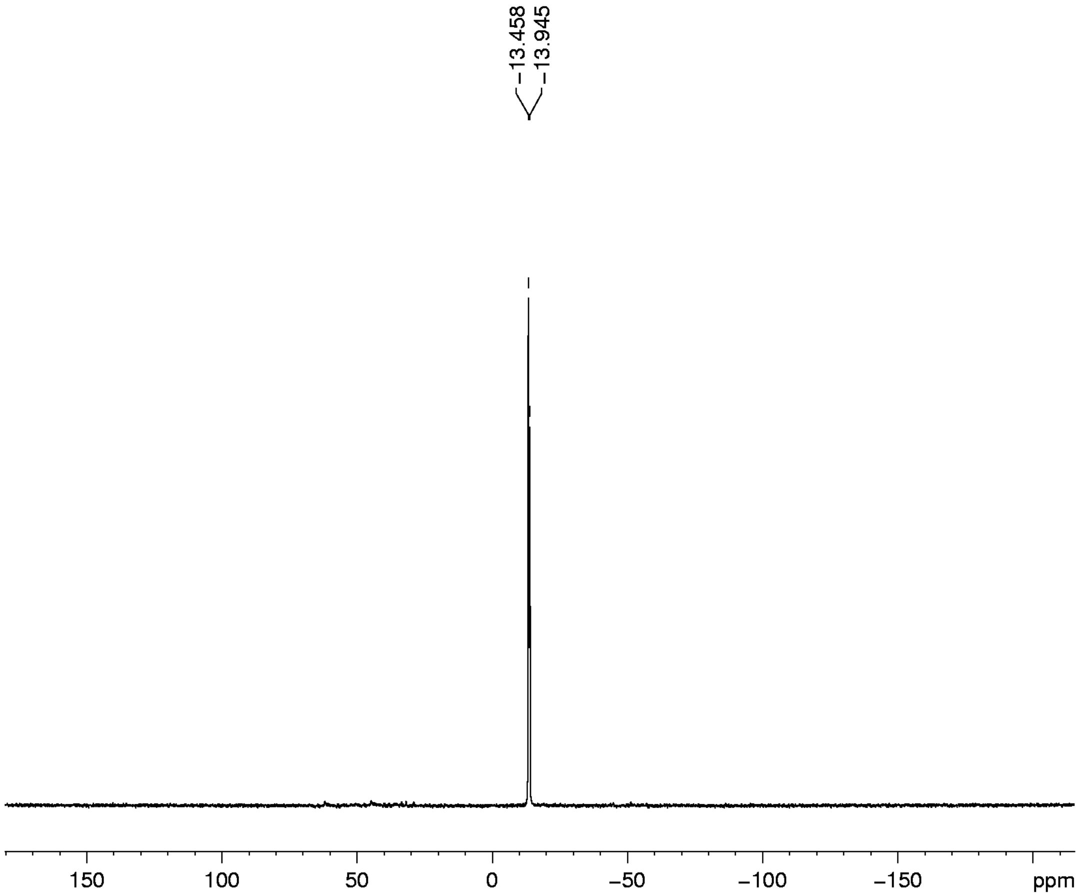

Current Data Parameters
NAME MRC−12
EXPNO 6
PROCNO 1

F2 − Acquisition Parameters
Date_ 20121207
Time 14.06
INSTRUM spect
PROBHD 5 mm PABBO B[illegible]
PULPROG zg
TD 32768
SOLVENT CDCl3
NS 30
DS 0
SWH 64102.563 Hz
FIDRES 1.956255 Hz
AQ 0.2555904 sec
RG 2050
DW 7.800 usec
DE 6.00 usec
TE 297.9 K
D1 2.00000000 sec
TD0 1

======== CHANNEL f1 ===
NUC1 31P
P1 9.10 usec
PL1 0 dB
PL1W 24.94303322 W
SFO1 161.9727429 MH[illegible]

F2 − Processing parameters
SI 16384
SF 161.9755930 MHz
WDW EM
SSB 0
LB 5.00 Hz
GB 0
PC 1.40

^{1}H NMR ($CDCl_3$): **2d**

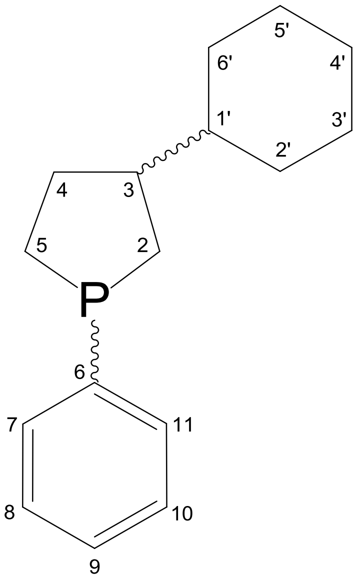

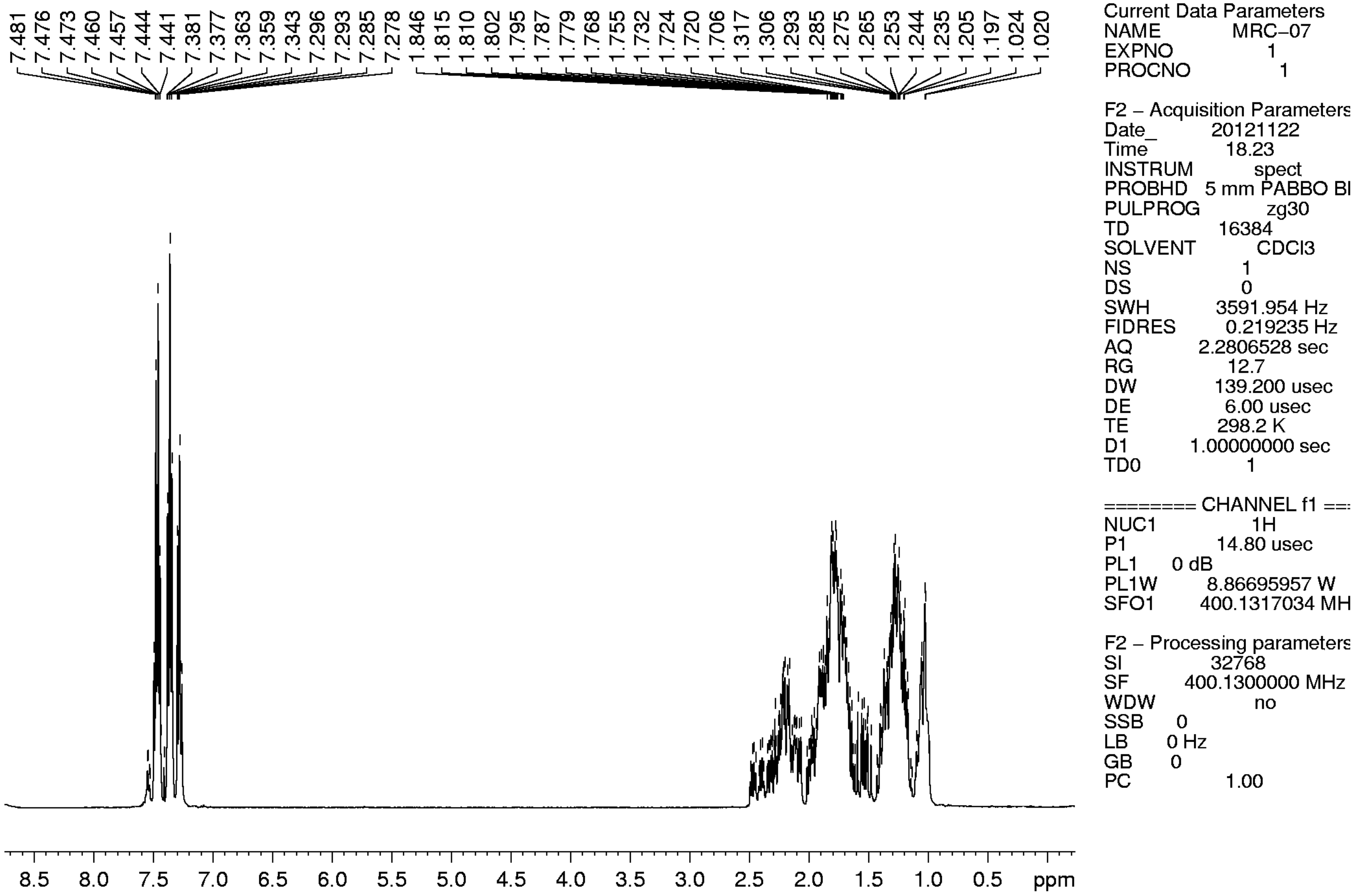

^{13}C NMR ($CDCl_3$): **2d**

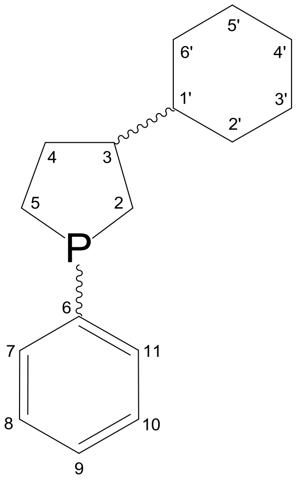

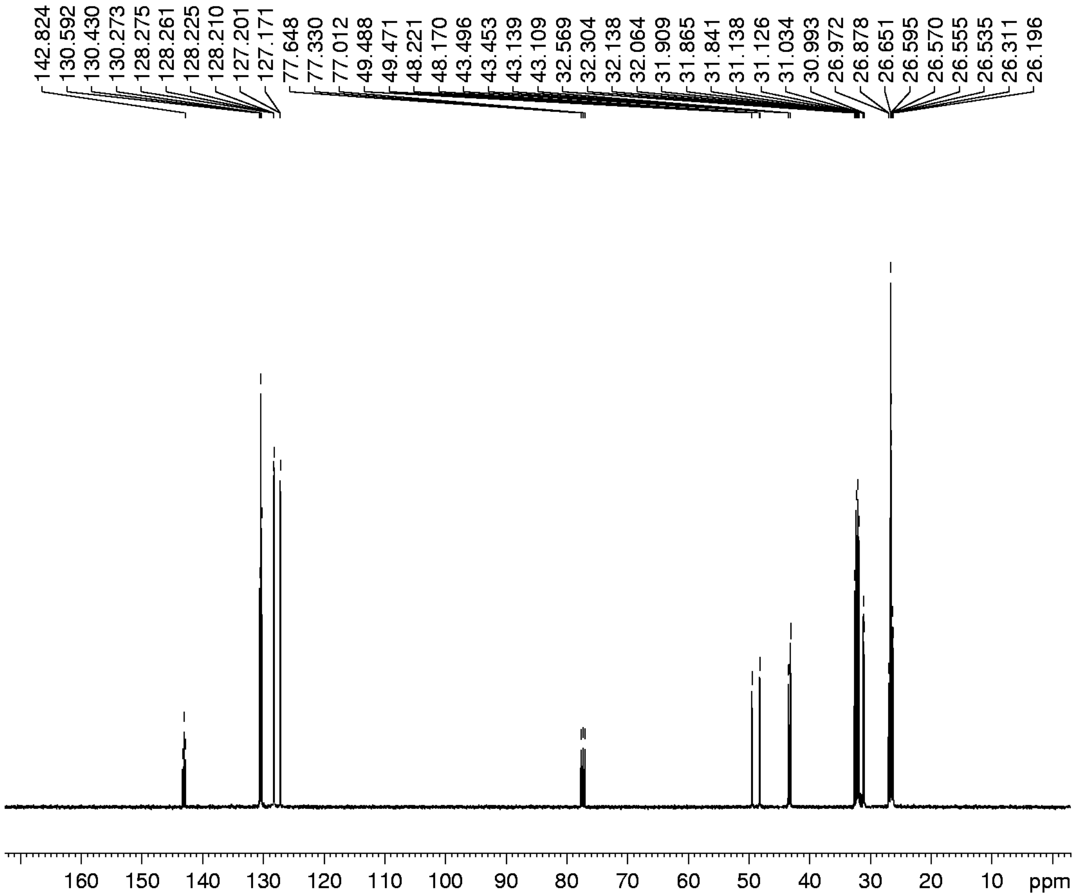

Current Data Parameters
NAME MRC−07
EXPNO 2
PROCNO 1

F2 − Acquisition Parameters
Date_ 20121122
Time 18.24
INSTRUM spect
PROBHD 5 mm PABBO BB−
PULPROG zgpg30
TD 32768
SOLVENT CDCl3
NS 231
DS 2
SWH 23148.148 Hz
FIDRES 0.706425 Hz
AQ 0.7077888 sec
RG 2050
DW 21.600 usec
DE 6.00 usec
TE 298.3 K
D1 1.00000000 sec
D11 0.03000000 sec
TD0 8

======== CHANNEL f1 ====
NUC1 13C
P1 10.00 usec
PL1 0 dB
PL1W 33.91046524 W
SFO1 100.6230315 MHz

======== CHANNEL f2 ====
CPDPRG[2 waltz16
NUC2 1H
PCPD2 90.00 usec
PL2 0 dB
PL12 15.68 dB
PL13 18.70 dB
PL2W 8.86695957 W
PL12W 0.23975886 W
PL13W 0.11961196 W
SFO2 400.1319206 MHz

F2 − Processing parameters
SI 65536
SF 100.6127727 MHz
WDW EM
SSB 0

^{31}P NMR ($CDCl_3$): **2d**

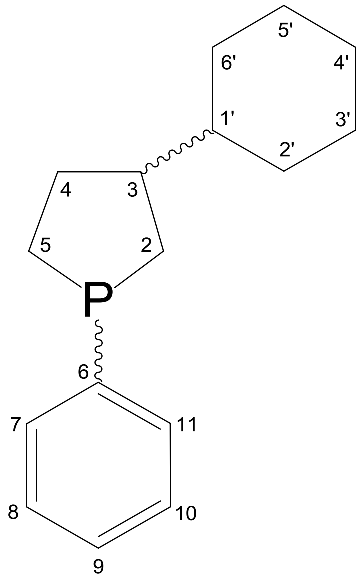

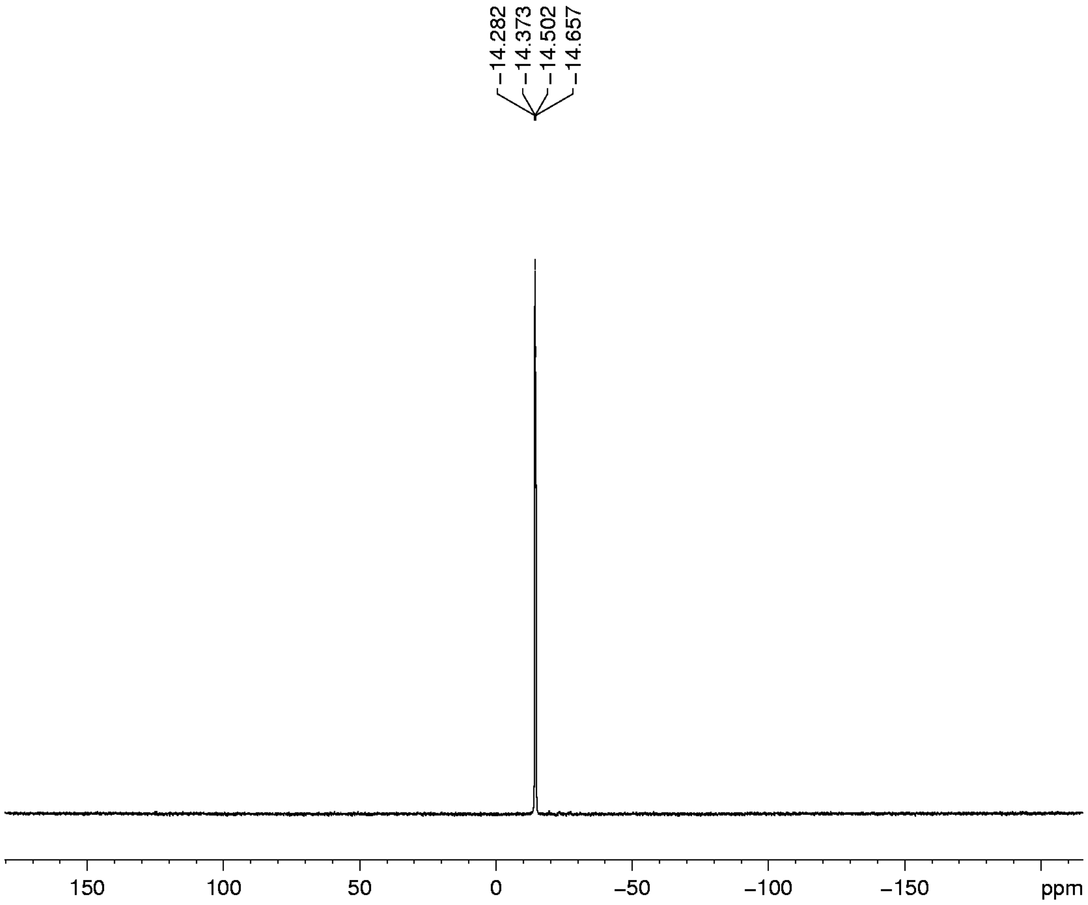

Current Data Parameters
NAME MRC–07
EXPNO 6
PROCNO 1

F2 – Acquisition Parameters
Date_ 20121122
Time 18.17
INSTRUM spect
PROBHD 5 mm PABBO BB
PULPROG zg
TD 32768
SOLVENT CDCl3
NS 32
DS 0
SWH 64102.563 Hz
FIDRES 1.956255 Hz
AQ 0.2555904 sec
RG 2050
DW 7.800 usec
DE 6.00 usec
TE 298.2 K
D1 2.00000000 sec
TD0 1

======== CHANNEL f1 ===
NUC1 31P
P1 9.10 usec
PL1 0 dB
PL1W 24.94303322 W
SFO1 161.9727429 MH

F2 – Processing parameters
SI 16384
SF 161.9755930 MHz
WDW EM
SSB 0
LB 5.00 Hz
GB 0
PC 1.40

COSYHH: **2d**

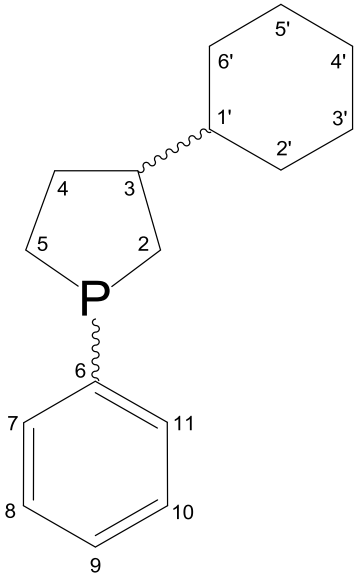

5'
6'
4'
1'
3'
2'
4
3
5
2
P
6
7
11
8
10
9

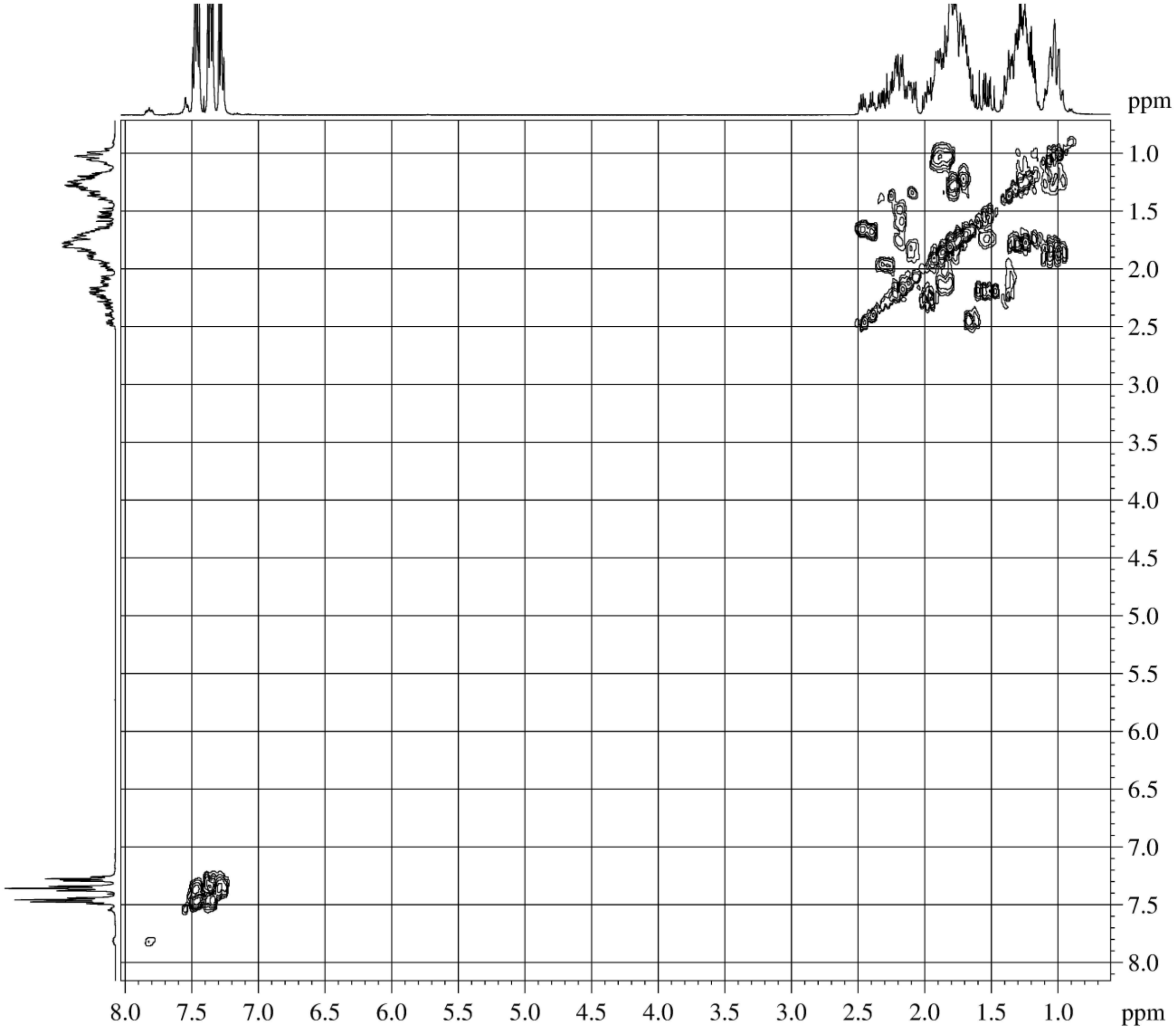

ppm
1.0
1.5
2.0
2.5
3.0
3.5
4.0
4.5
5.0
5.5
6.0
6.5
7.0
7.5
8.0
8.0
7.5
7.0
6.5
6.0
5.5
5.0
4.5
4.0
3.5
3.0
2.5
2.0
1.5
1.0
ppm

HSQC: **2d**

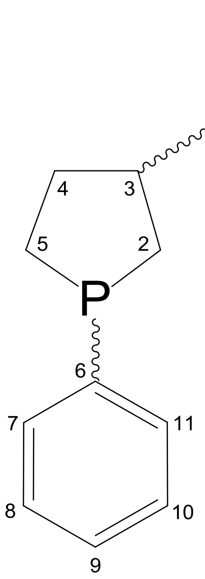
5'
6'
4'
1'
3'
2'
4
3
5
2
P
6
7
11
8
10
9

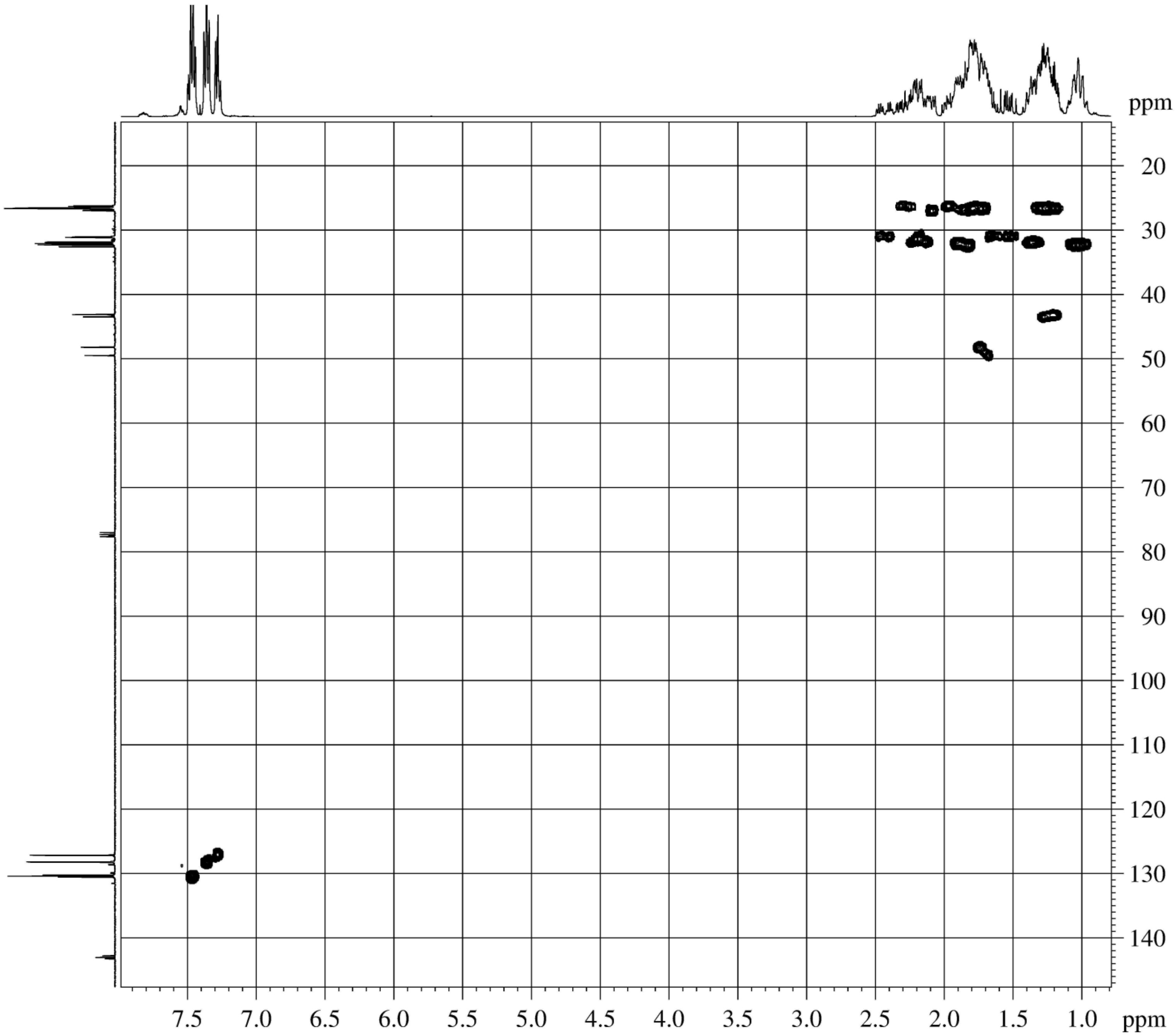
ppm
20
30
40
50
60
70
80
90
100
110
120
130
140
7.5 7.0 6.5 6.0 5.5 5.0 4.5 4.0 3.5 3.0 2.5 2.0 1.5 1.0 ppm

HMBC: **2d**

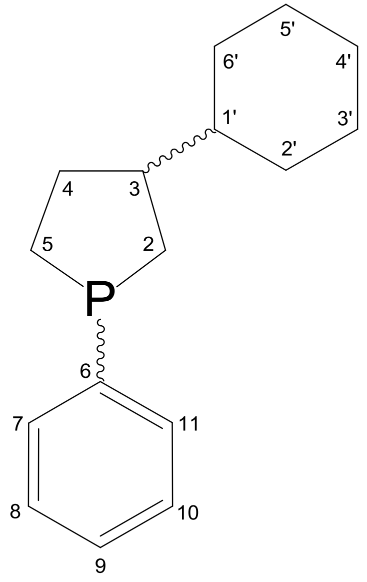

P
1'
2'
3'
4'
5'
6'
2
3
4
5
6
7
8
9
10
11

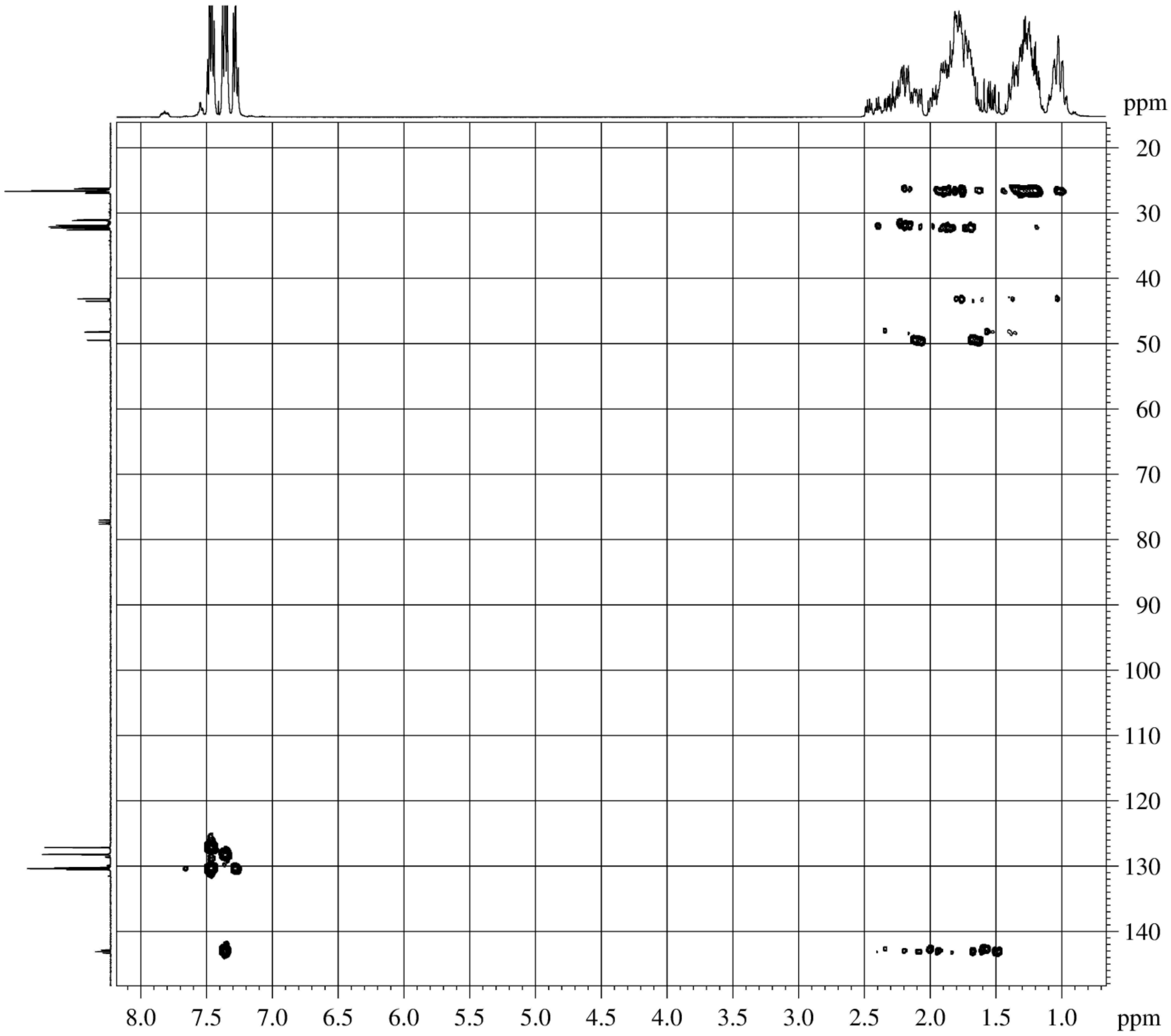

ppm
20
30
40
50
60
70
80
90
100
110
120
130
140
8.0
7.5
7.0
6.5
6.0
5.5
5.0
4.5
4.0
3.5
3.0
2.5
2.0
1.5
1.0
ppm

^{1}H NMR ($CDCl_3$): **2e**

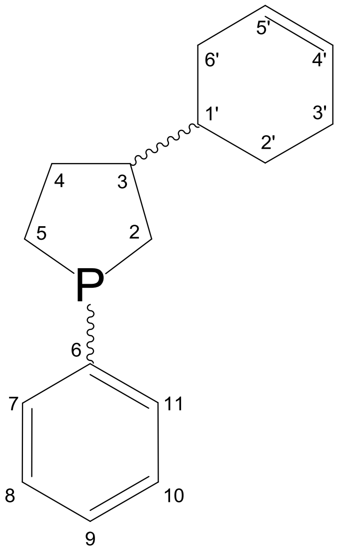

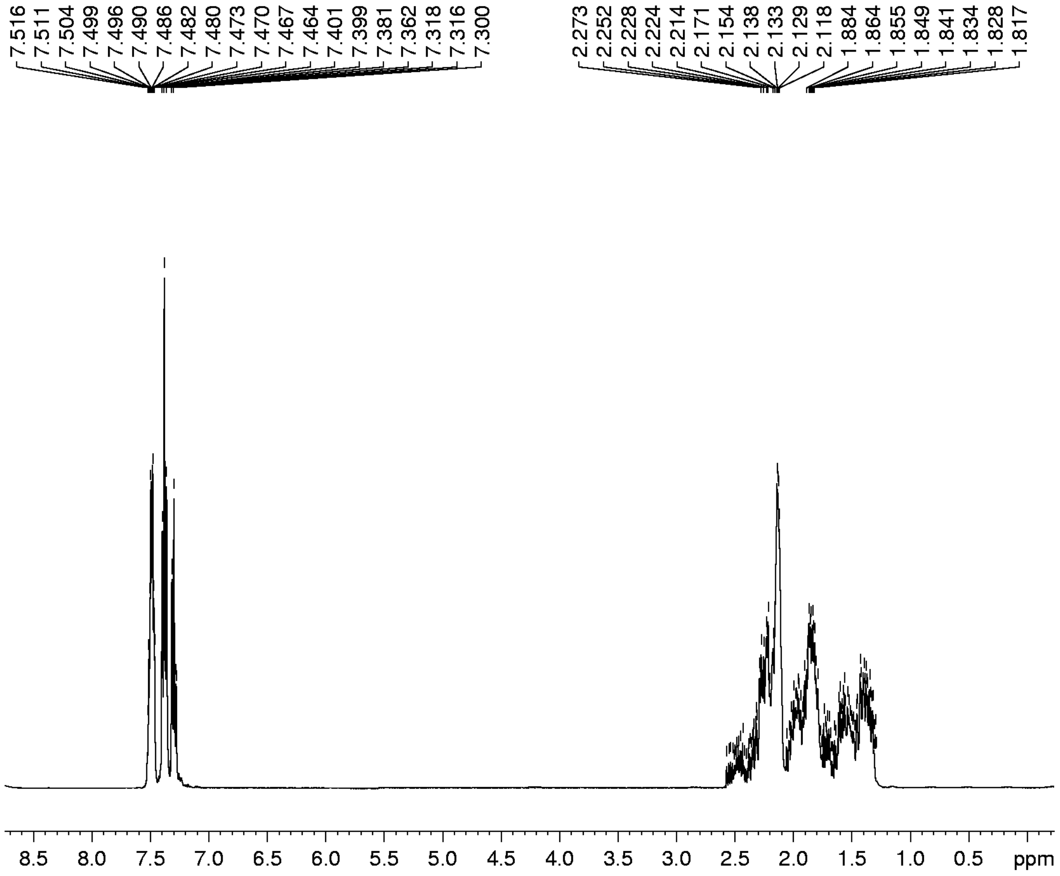

Current Data Parameters
NAME MRC–08
EXPNO 1
PROCNO 1

F2 – Acquisition Parameters
Date_ 20121122
Time 19.02
INSTRUM spect
PROBHD 5 mm PABBO BE
PULPROG zg30
TD 16384
SOLVENT CDCl3
NS 1
DS 0
SWH 3591.954 Hz
FIDRES 0.219235 Hz
AQ 2.2806528 sec
RG 12.7
DW 139.200 usec
DE 6.00 usec
TE 298.3 K
D1 1.00000000 sec
TD0 1

======== CHANNEL f1 ===
NUC1 1H
P1 14.80 usec
PL1 0 dB
PL1W 8.86695957 W
SFO1 400.1317034 MH:

F2 – Processing parameters
SI 32768
SF 400.1300000 MHz
WDW no
SSB 0
LB 0 Hz
GB 0
PC 1.00

^{13}C NMR ($CDCl_3$): **2e**

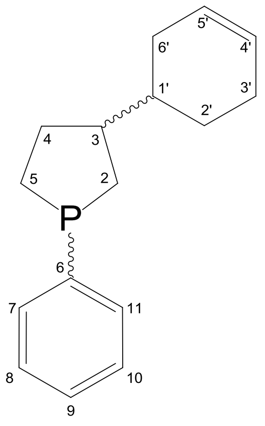

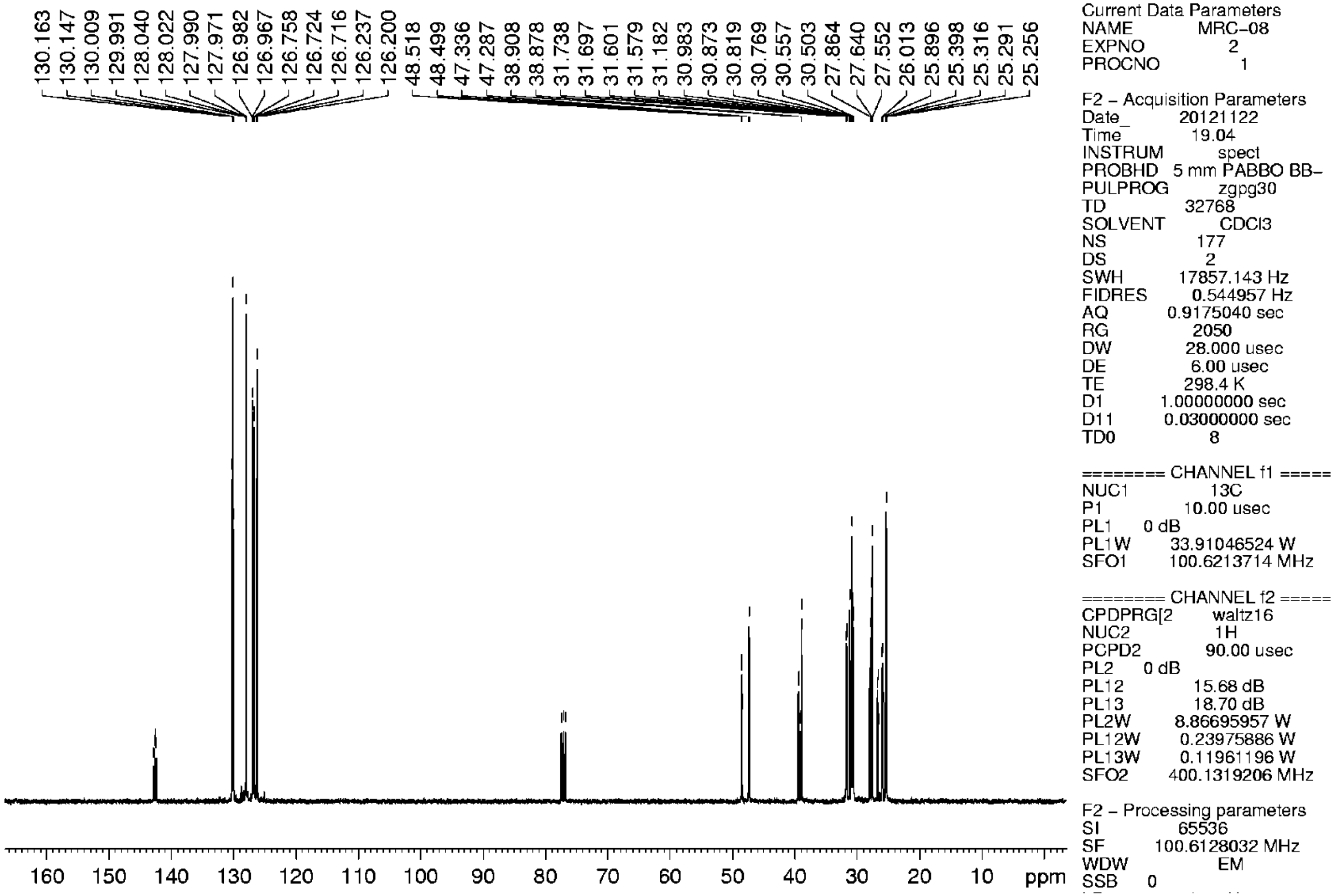

^{31}P NMR ($CDCl_3$): **2e**

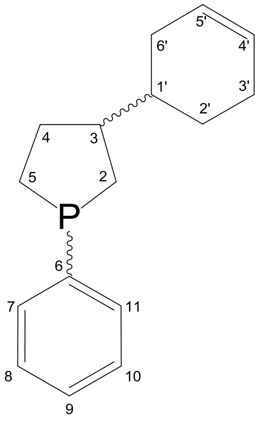

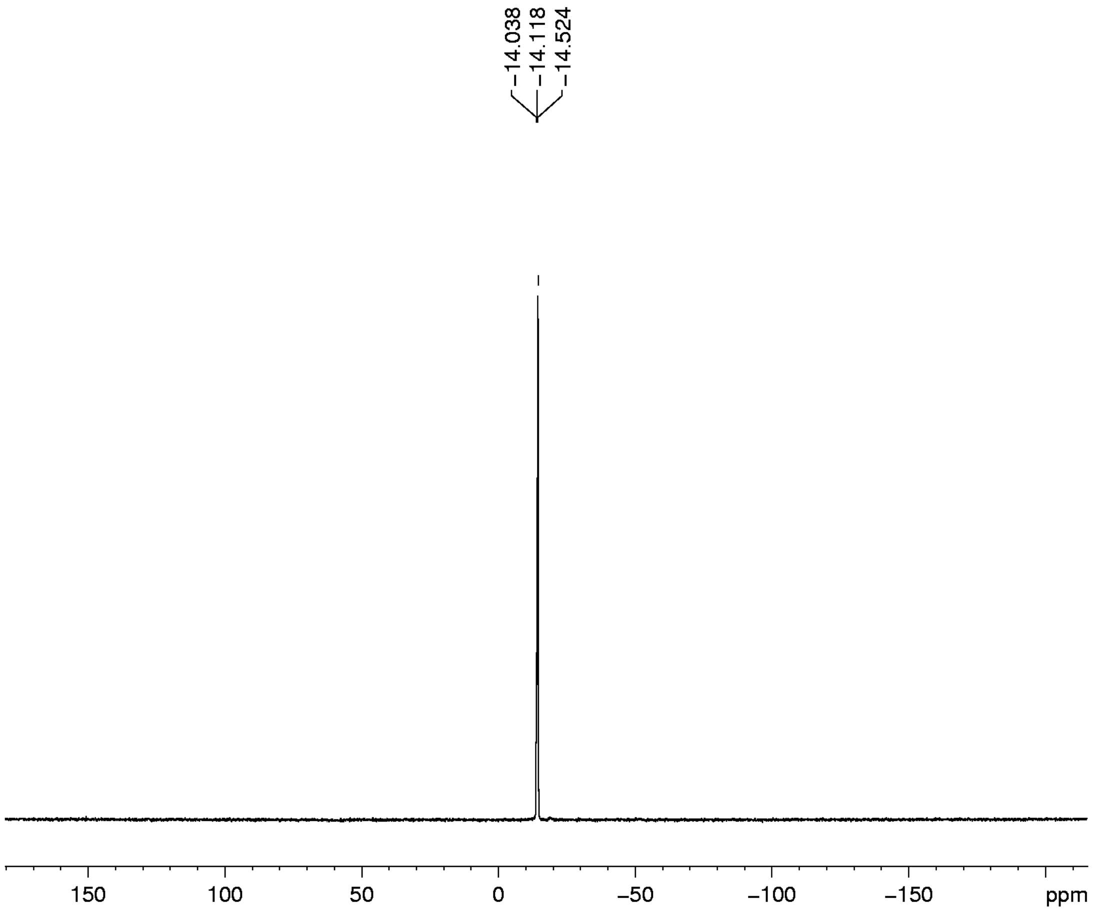

Current Data Parameters
NAME MRC−08
EXPNO 6
PROCNO 1

F2 − Acquisition Parameters
Date_ 20121122
Time 18.13
INSTRUM spect
PROBHD 5 mm PABBO BI
PULPROG zg
TD 32768
SOLVENT CDCl3
NS 18
DS 0
SWH 64102.563 Hz
FIDRES 1.956255 Hz
AQ 0.2555904 sec
RG 2050
DW 7.800 usec
DE 6.00 usec
TE 298.1 K
D1 2.00000000 sec
TD0 1

======== CHANNEL f1 ==
NUC1 31P
P1 9.10 usec
PL1 0 dB
PL1W 24.94303322 W
SFO1 161.9727429 MH

F2 − Processing parameters
SI 16384
SF 161.9755930 MHz
WDW EM
SSB 0
LB 5.00 Hz
GB 0
PC 1.40

COSYHH: **2e**

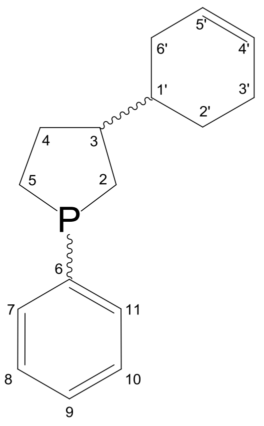

P
1'
2'
3'
4'
5'
6'
2
3
4
5
6
7
8
9
10
11

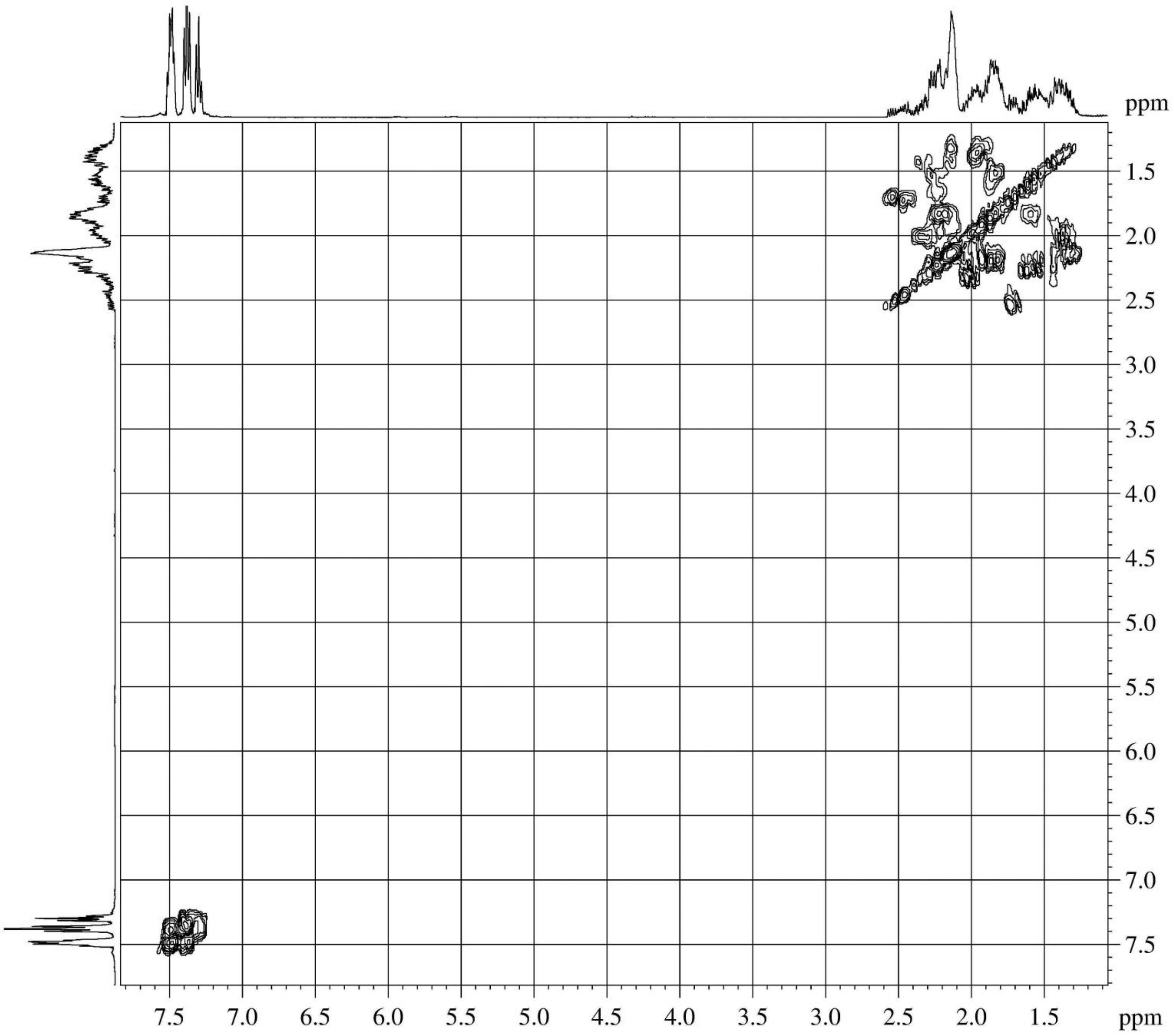

ppm
1.5
2.0
2.5
3.0
3.5
4.0
4.5
5.0
5.5
6.0
6.5
7.0
7.5
ppm
7.5
7.0
6.5
6.0
5.5
5.0
4.5
4.0
3.5
3.0
2.5
2.0
1.5
ppm

HSQC: **2e**

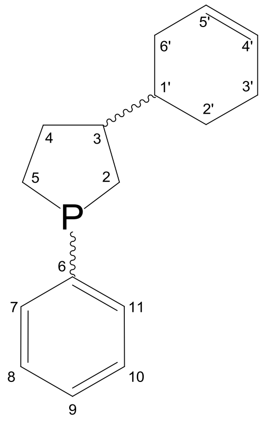

5'
6'
4'
1'
3'
2'
4
3
5
2
P
6
7
11
8
10
9

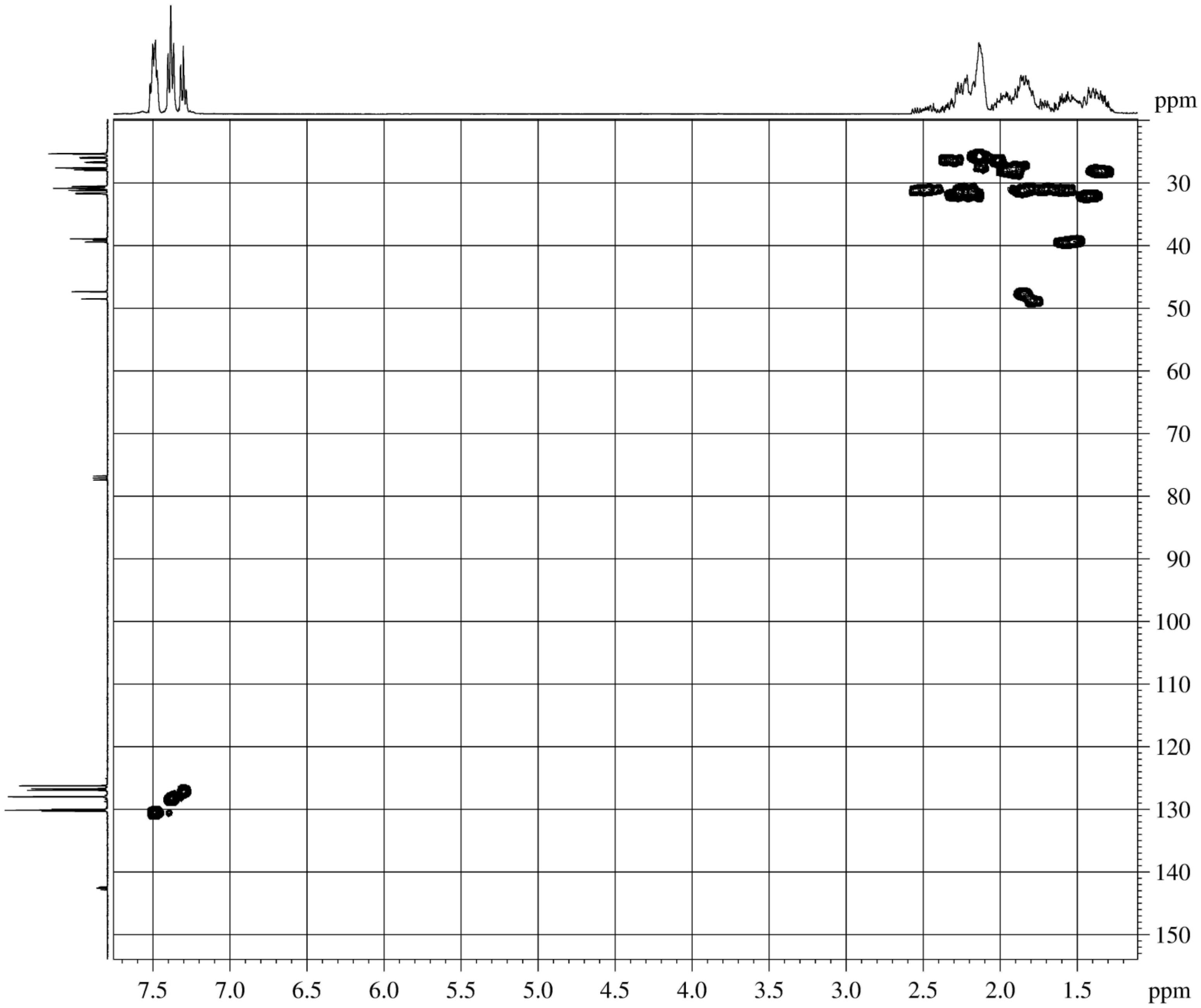

ppm
30
40
50
60
70
80
90
100
110
120
130
140
150
7.5
7.0
6.5
6.0
5.5
5.0
4.5
4.0
3.5
3.0
2.5
2.0
1.5
ppm

HMBC: **2e**

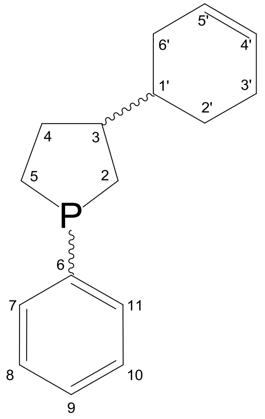
5'
6'
4'
1'
3'
2'
4
3
5
2
P
6
7
11
8
10
9

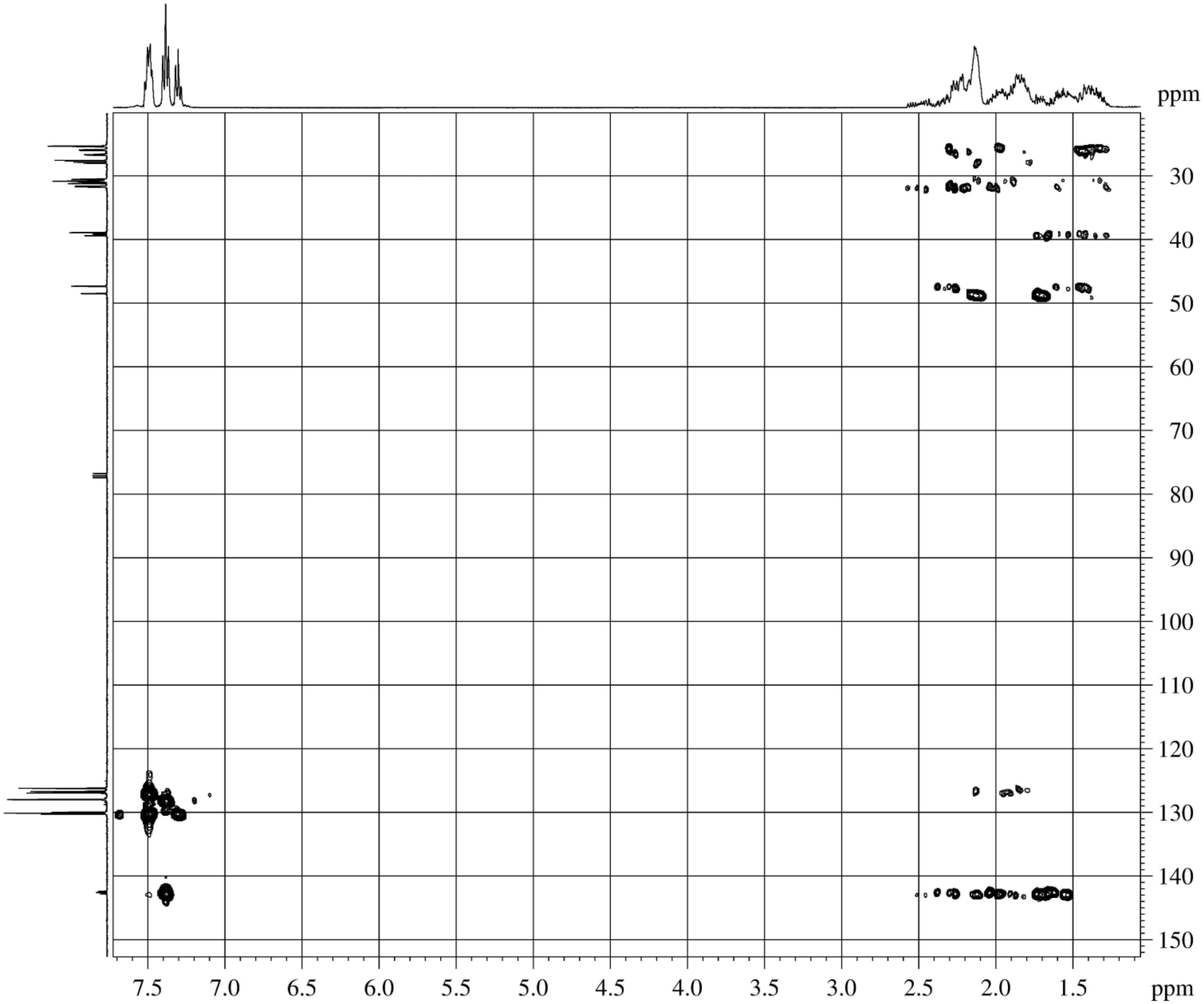
ppm
30
40
50
60
70
80
90
100
110
120
130
140
150
7.5
7.0
6.5
6.0
5.5
5.0
4.5
4.0
3.5
3.0
2.5
2.0
1.5
ppm

^{1}H NMR ($CDCl_3$): **2f**

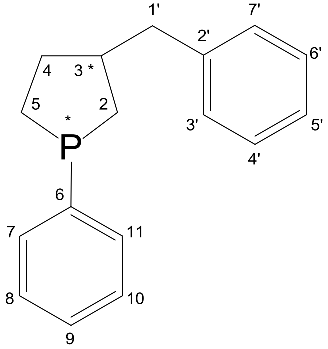

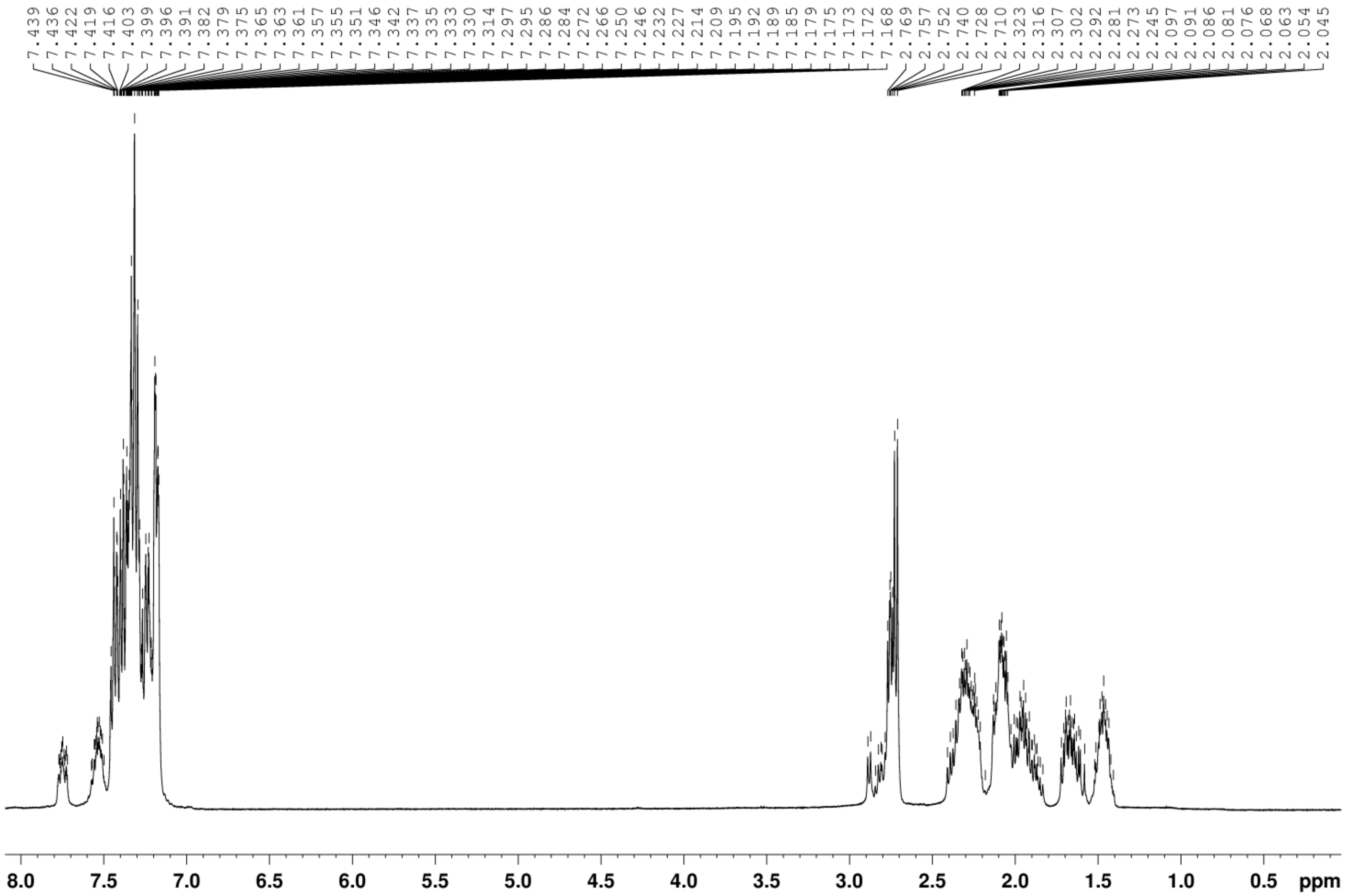

^{13}C NMR ($CDCl_3$): **2f**

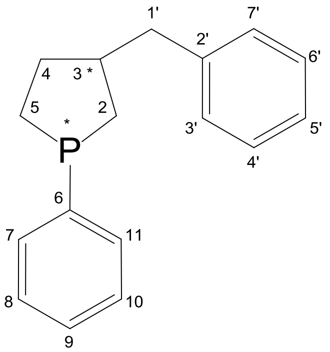

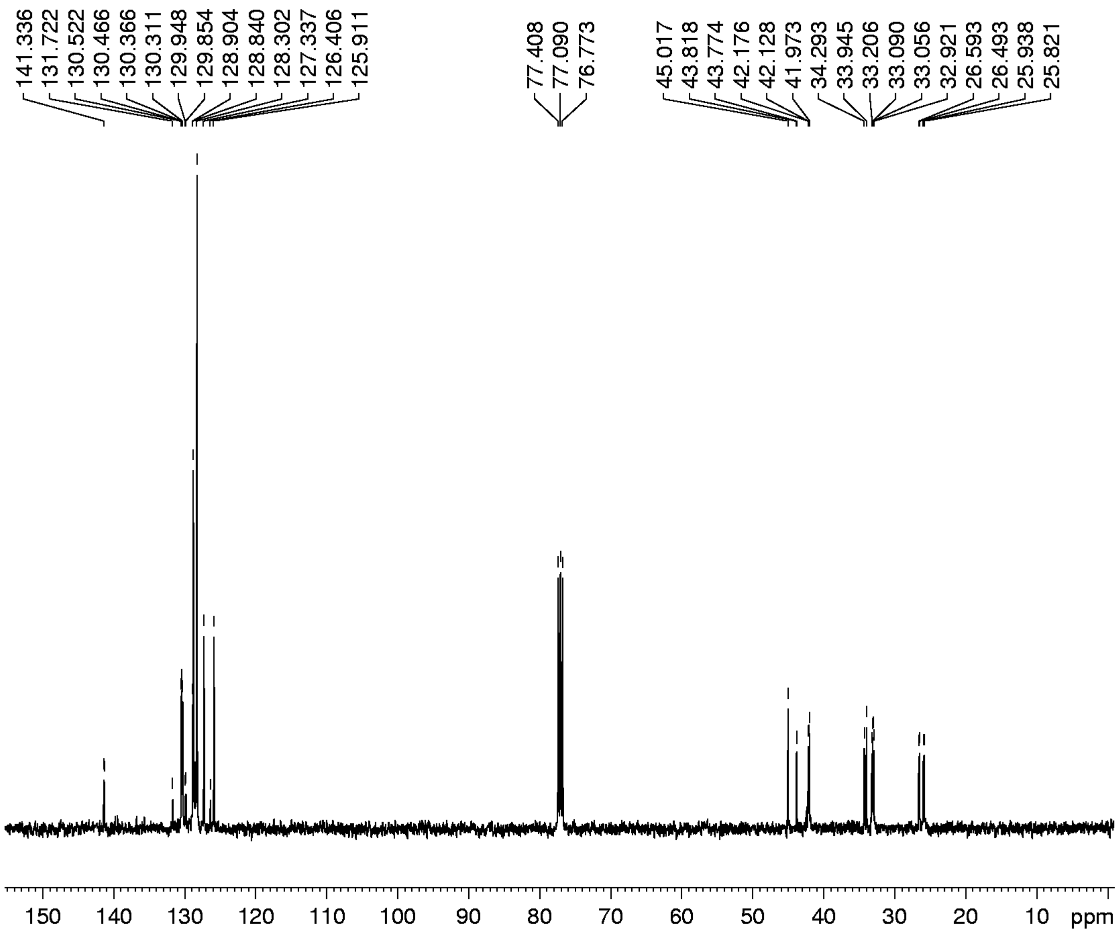

Current Data Parameters
NAME mahamathanova
EXPNO 69
PROCNO 1

F2 – Acquisition Parameters
Date_ 20110602
Time 12.41
INSTRUM spect
PROBHD 5 mm PABBO BB-
PULPROG zgpg30
TD 32144
SOLVENT CDCl3
NS 140
DS 0
SWH 16891.893 Hz
FIDRES 0.525507 Hz
AQ 0.9514624 sec
RG 2050
DW 29.600 usec
DE 30.00 usec
TE 299.1 K
D1 1.00000000 sec
D11 0.03000000 sec
TD0 1

======== CHANNEL f1 ====
NUC1 13C
P1 10.00 usec
PL1 0 dB
PL1W 33.91046524 W
SFO1 100.6199552 MHz

======== CHANNEL f2 ====
CPDPRG[2 waltz16
NUC2 1H
PCPD2 90.00 usec
PL2 0 dB
PL12 15.68 dB
PL13 18.70 dB
PL2W 8.86695957 W
PL12W 0.23975886 W
PL13W 0.11961196 W
SFO2 400.1317606 MHz

F2 – Processing parameters
SI 32768
SF 100.6127690 MHz
WDW EM
SSB 0

^{31}P NMR ($CDCl_3$): **2f**

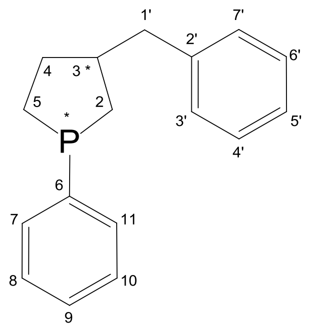

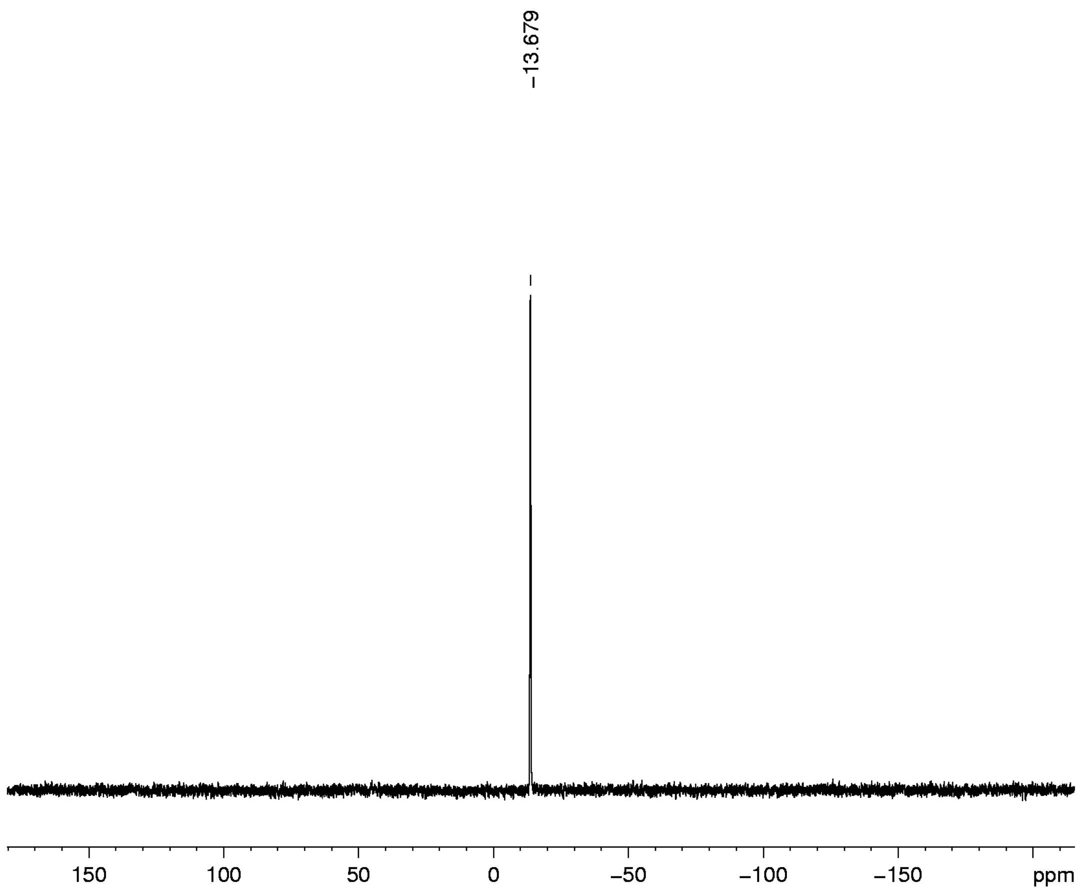

Current Data Parameters
NAME mahamathanova
EXPNO 74
PROCNO 1

F2 – Acquisition Parameters
Date_ 20110602
Time 13.25
INSTRUM spect
PROBHD 5 mm PABBO BE
PULPROG zg
TD 32768
SOLVENT CDCl3
NS 65
DS 0
SWH 64102.563 Hz
FIDRES 1.956255 Hz
AQ 0.2555904 sec
RG 2050
DW 7.800 usec
DE 6.00 usec
TE 298.6 K
D1 2.00000000 sec
TD0 1

======== CHANNEL f1 ===
NUC1 31P
P1 9.10 usec
PL1 0 dB
PL1W 24.94303322 W
SFO1 161.9727429 MH:

F2 – Processing parameters
SI 16384
SF 161.9755930 MHz
WDW EM
SSB 0
LB 5.00 Hz
GB 0
PC 1.40

^{1}H NMR ($CDCl_3$): **2g**

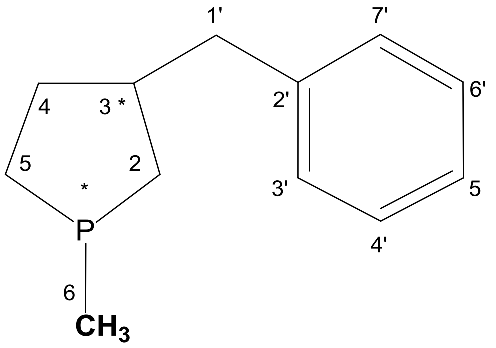

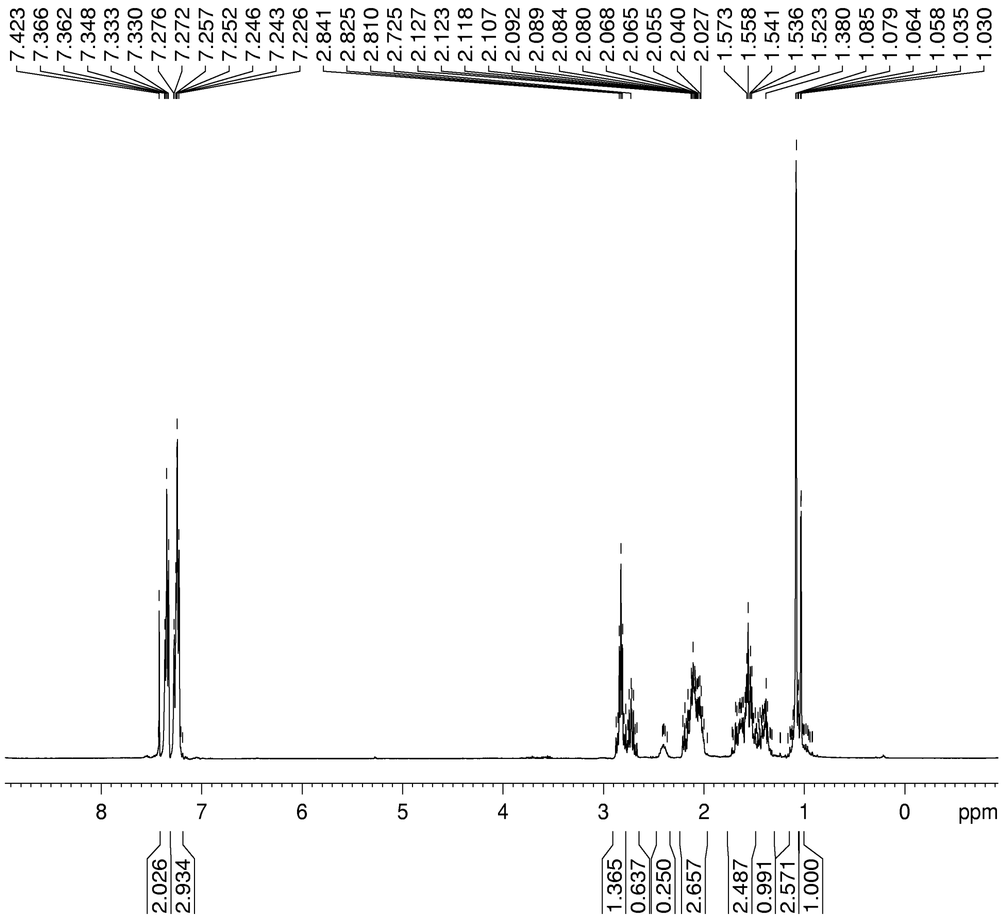

Current Data Parameters
NAME KRA–288
EXPNO 1
PROCNO 1

F2 – Acquisition Parameters
Date_ 20141010
Time 13.11
INSTRUM spect
PROBHD 5 mm PABBO BB–
PULPROG zg30
TD 16384
SOLVENT CDCl3
NS 1
DS 0
SWH 3955.696 Hz
FIDRES 0.241437 Hz
AQ 2.0709376 sec
RG 18
DW 126.400 usec
DE 6.00 usec
TE 299.4 K
D1 1.00000000 sec
TD0 1

======== CHANNEL f1 ======
SFO1 400.1316062 MHz
NUC1 1H
P1 14.80 usec
PLW1 8.86695862 W

F2 – Processing parameters
SI 32768
SF 400.1300003 MHz
WDW no
SSB 0
LB 0 Hz
GB 0
PC 1.00

^{13}C NMR (CDCl$_3$): **2g**

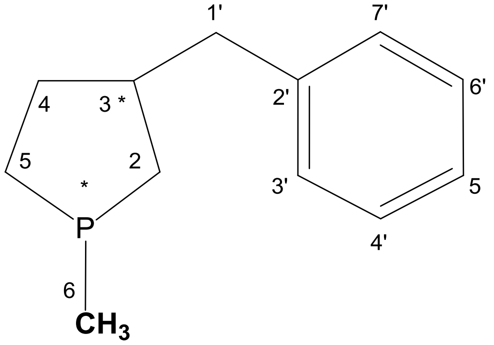

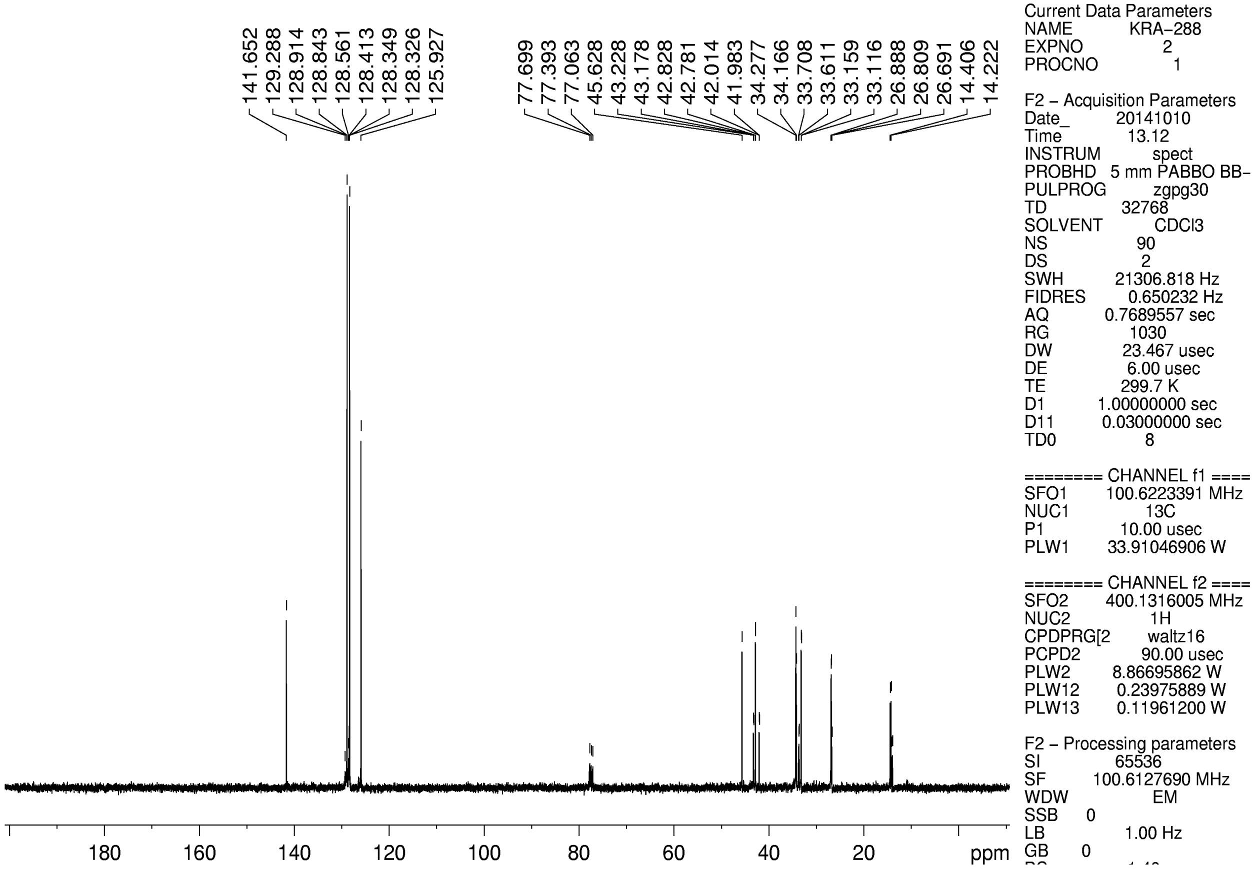

^{31}P NMR ($CDCl_3$): **2g**

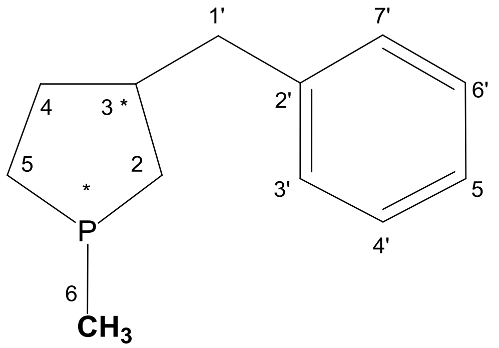

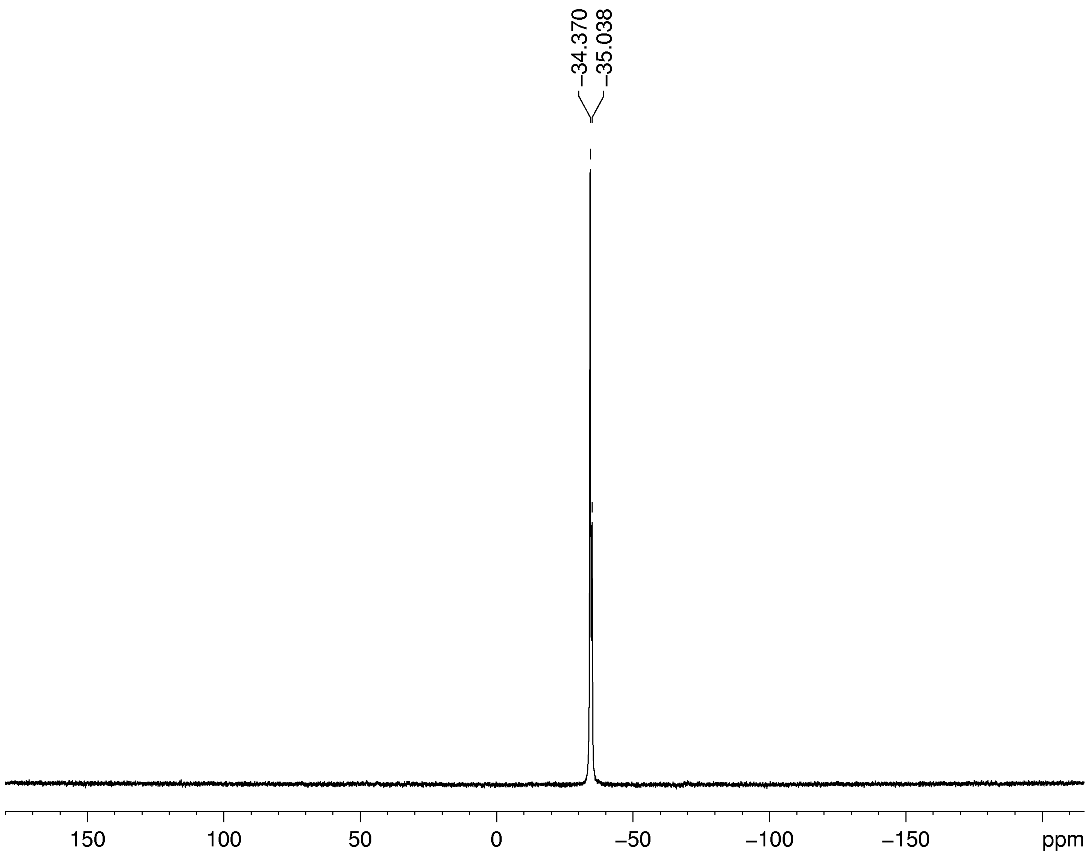

Current Data Parameters
NAME KRA−288
EXPNO 7
PROCNO 1

F2 − Acquisition Parameters
Date_ 20141010
Time 13.08
INSTRUM spect
PROBHD 5 mm PABBO BB−
PULPROG zg
TD 32768
SOLVENT CDCl3
NS 19
DS 0
SWH 64102.563 Hz
FIDRES 1.956255 Hz
AQ 0.2555904 sec
RG 1620
DW 7.800 usec
DE 6.50 usec
TE 299.4 K
D1 2.00000000 sec
TD0 1

======== CHANNEL f1 ====
SFO1 161.9727429 MHz
NUC1 31P
P1 10.00 usec
PLW1 25.00000000 W

F2 − Processing parameters
SI 16384
SF 161.9755930 MHz
WDW EM
SSB 0
LB 5.00 Hz
GB 0
PC 1.40

^{1}H NMR ($CDCl_3$): **2h**

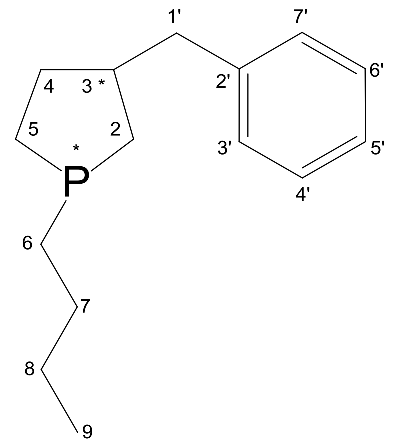

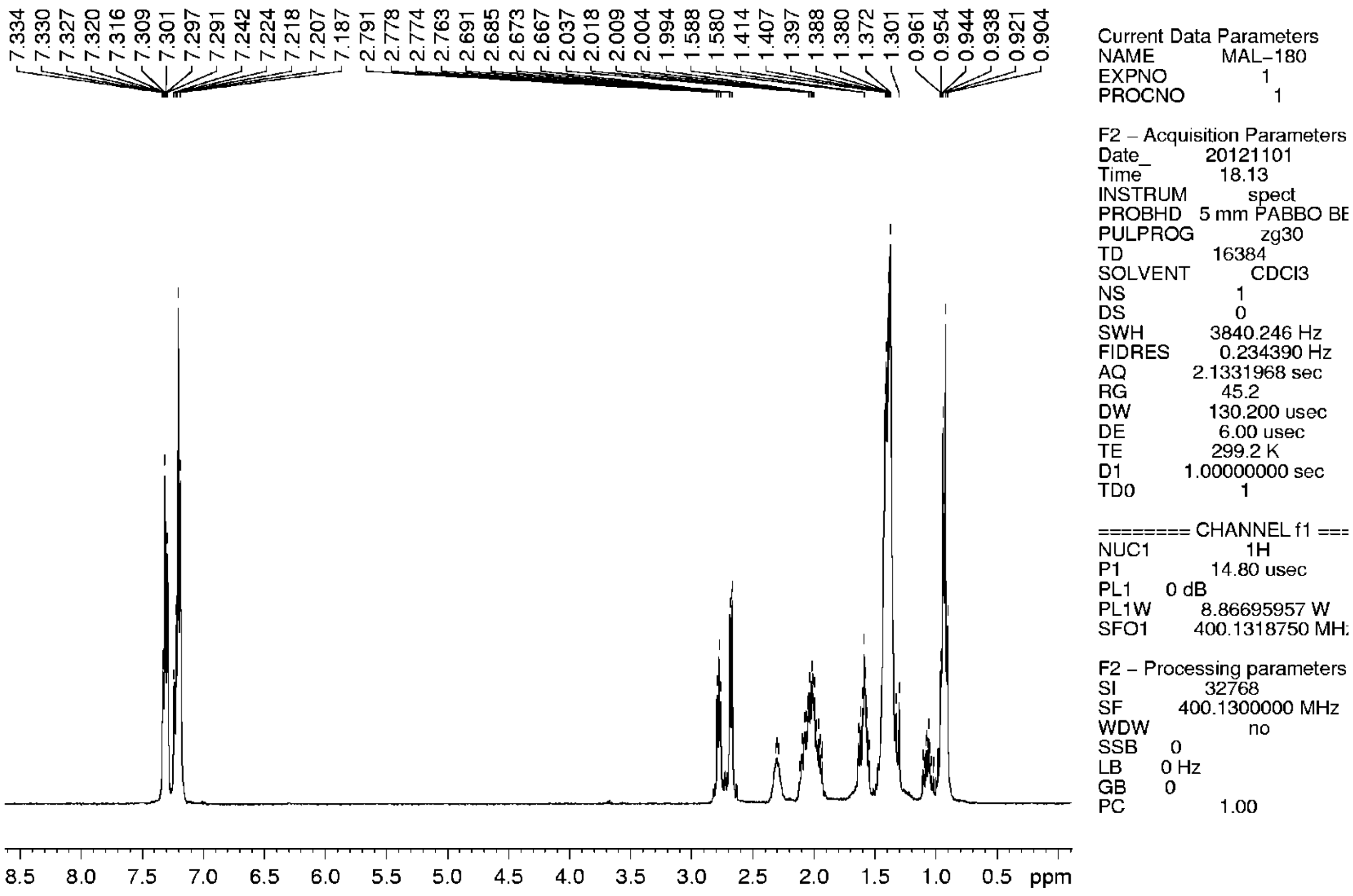

^{13}C NMR ($CDCl_3$): **2h**

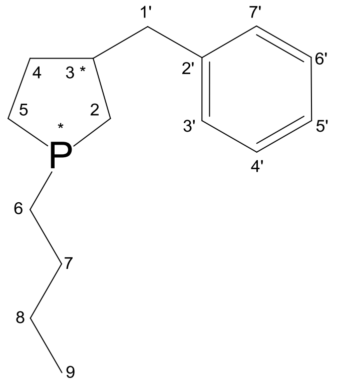

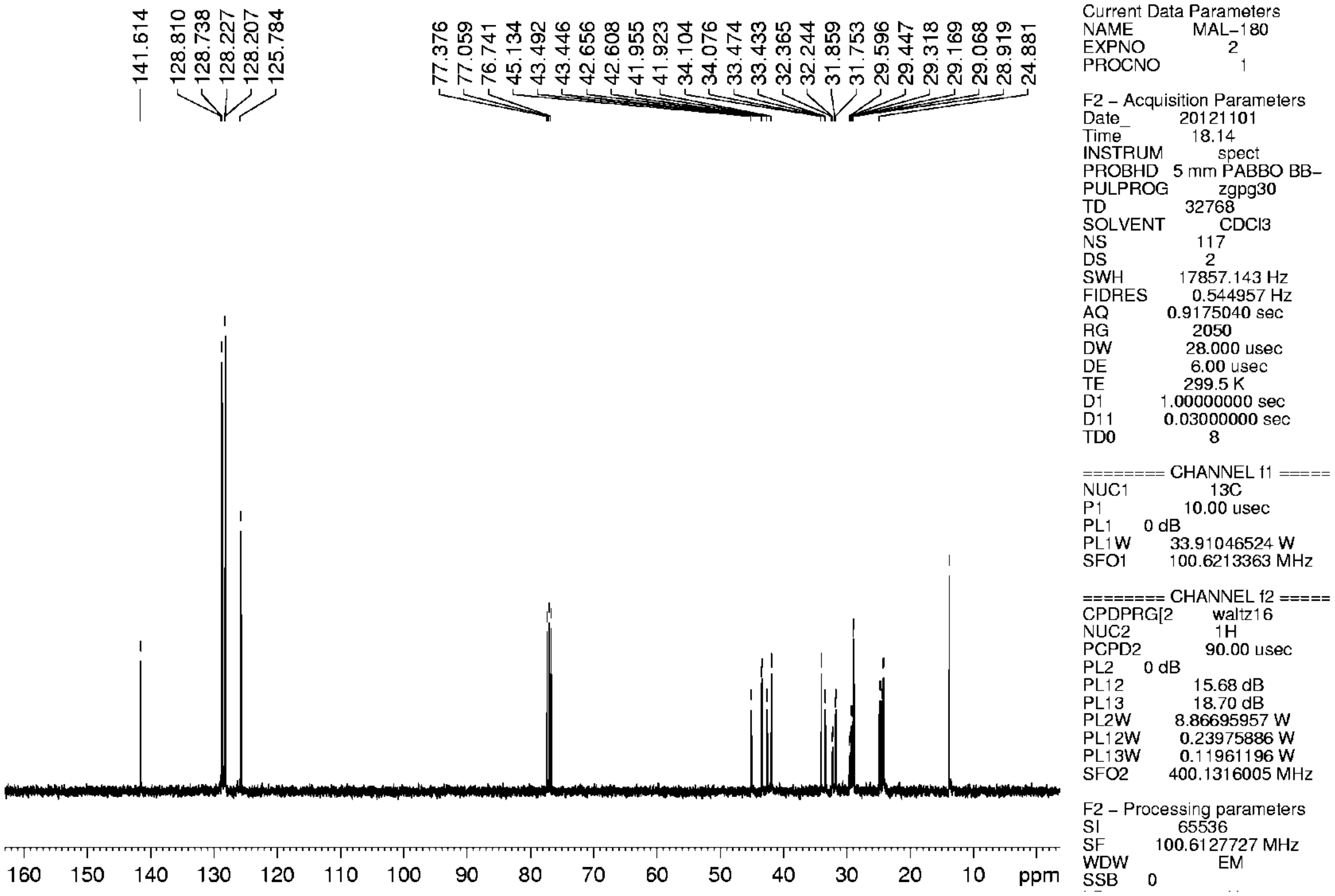

^{31}P NMR ($CDCl_3$): **2h**

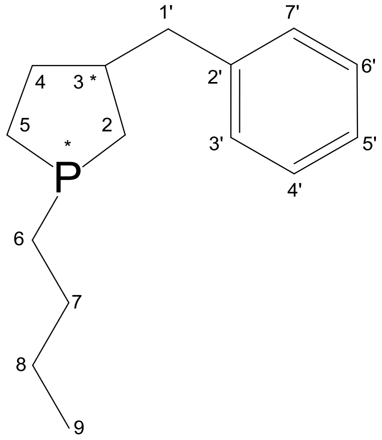

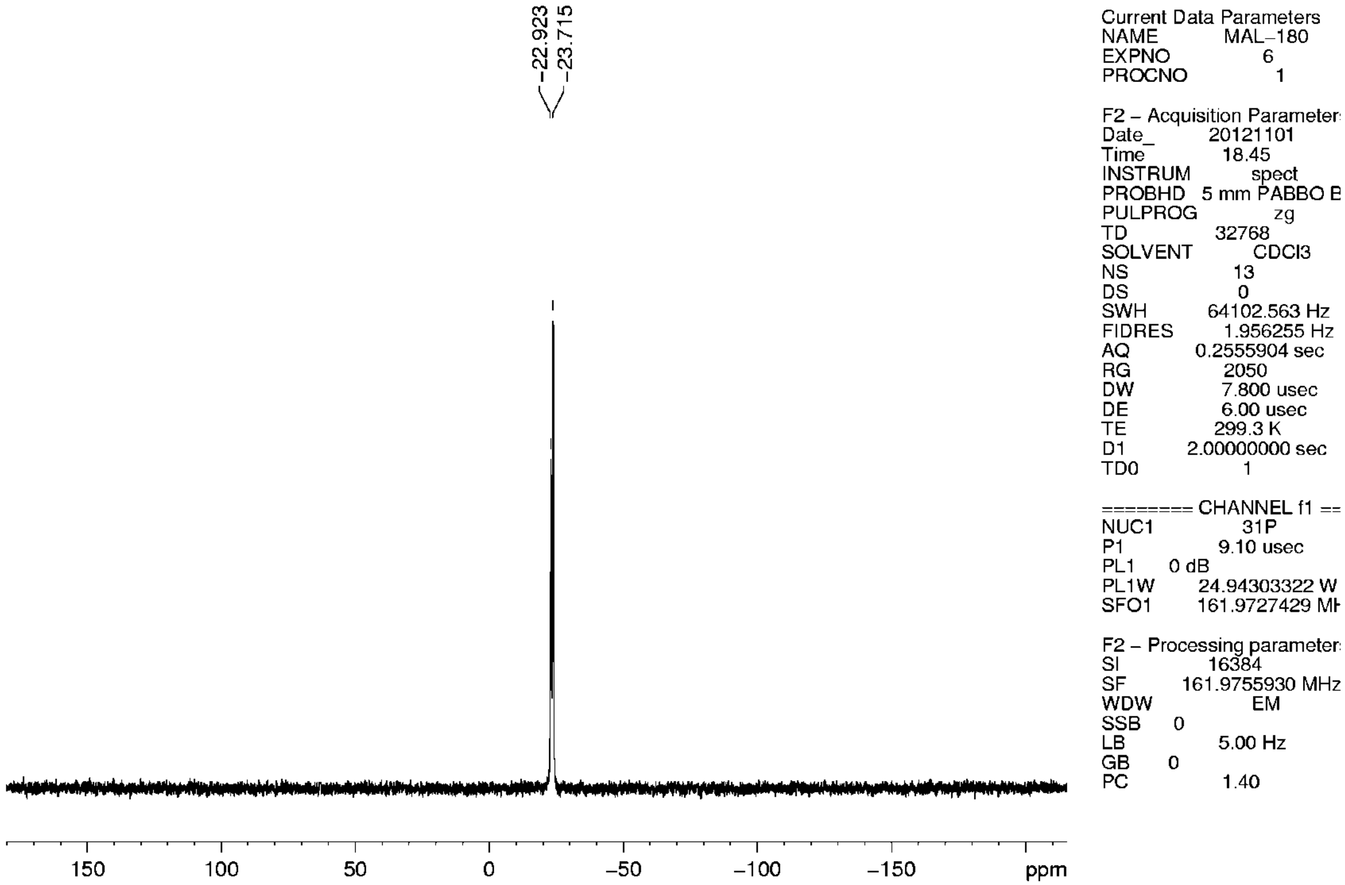

^{1}H NMR ($CDCl_3$): **3a**

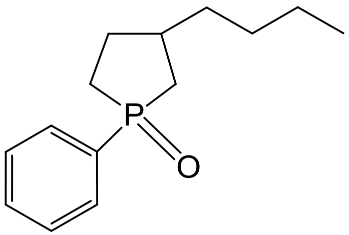

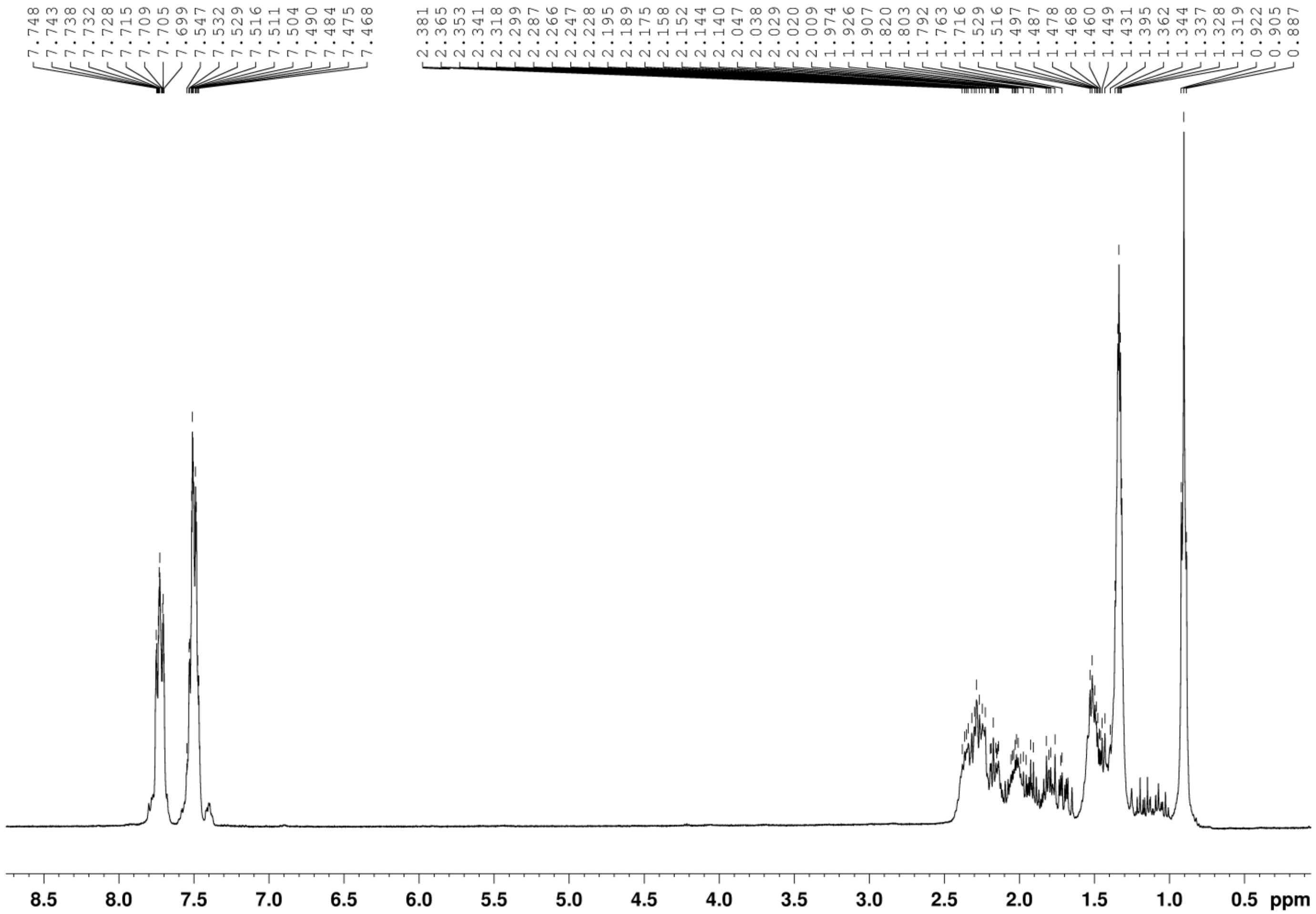

^{13}C NMR ($CDCl_3$): **3a**

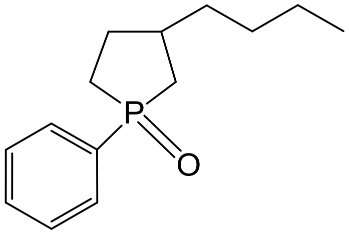

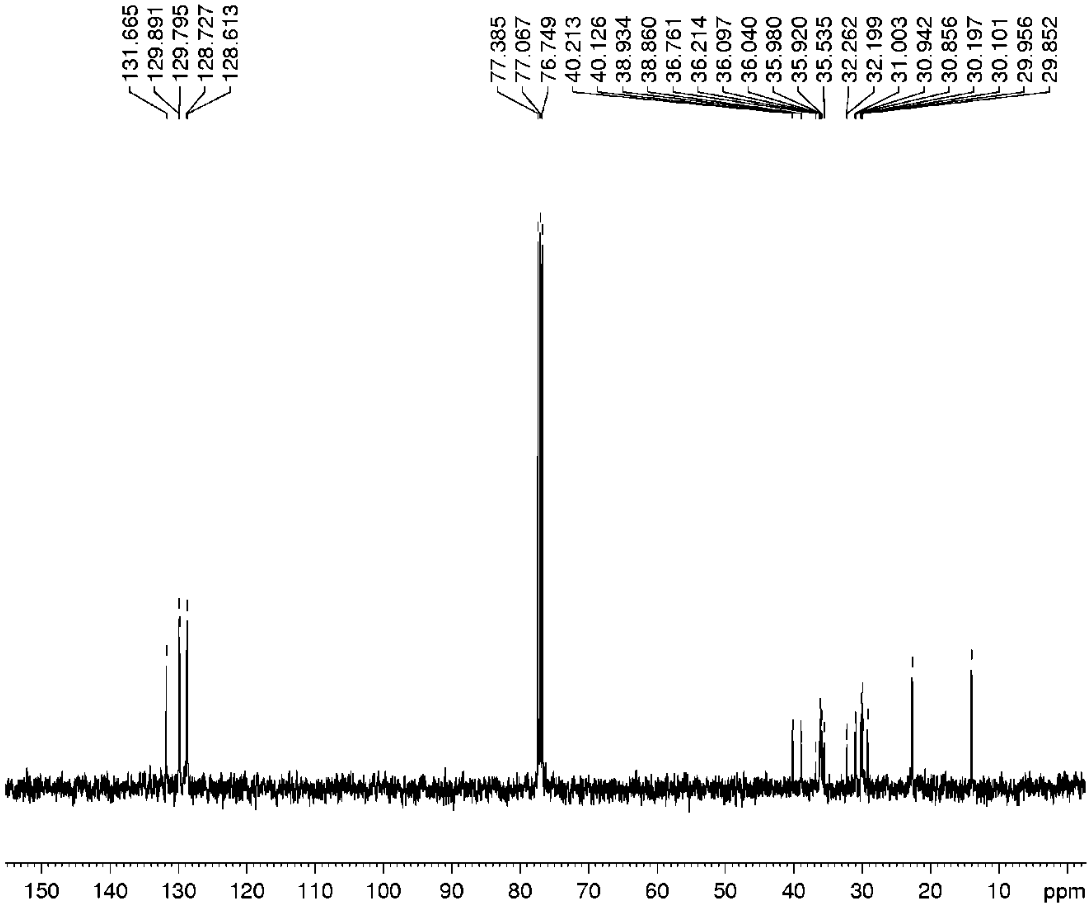

Current Data Parameters
NAME mahamathanova
EXPNO 116
PROCNO 1

F2 - Acquisition Parameters
Date_ 20110630
Time 14.08
INSTRUM spect
PROBHD 5 mm PABBO BB
PULPROG zgpg30
TD 32144
SOLVENT CDCl3
NS 92
DS 0
SWH 16891.893 Hz
FIDRES 0.525507 Hz
AQ 0.9514624 sec
RG 2050
DW 29.600 usec
DE 30.00 usec
TE 299.3 K
D1 1.00000000 sec
D11 0.03000000 sec
TD0 1

======== CHANNEL f1 ====
NUC1 13C
P1 10.00 usec
PL1 0 dB
PL1W 33.91046524 W
SFO1 100.6199552 MHz

======== CHANNEL f2 ====
CPDPRG[2 waltz16
NUC2 1H
PCPD2 90.00 usec
PL2 0 dB
PL12 15.68 dB
PL13 18.70 dB
PL2W 8.86695957 W
PL12W 0.23975886 W
PL13W 0.11961196 W
SFO2 400.1317606 MHz

F2 - Processing parameters
SI 32768
SF 100.6127690 MHz
WDW EM
SSB 0

^{31}P NMR ($CDCl_3$): **3a**

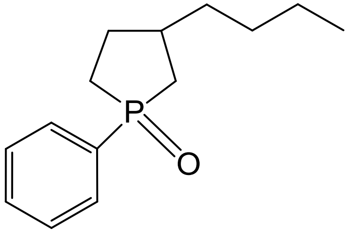

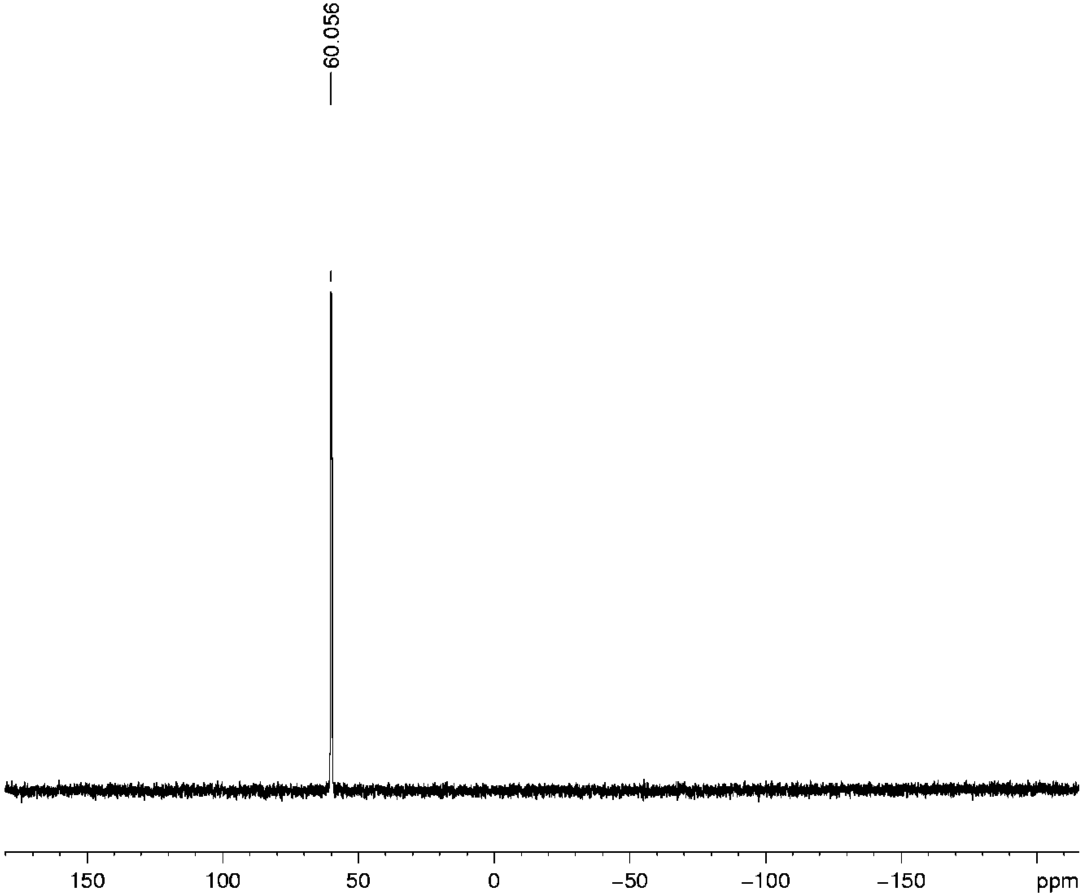

Current Data Parameters
NAME mahamathanova
EXPNO 121
PROCNO 1

F2 – Acquisition Parameters
Date_ 20110630
Time 14.35
INSTRUM spect
PROBHD 5 mm PABBO BB
PULPROG zg
TD 32768
SOLVENT CDCl3
NS 7
DS 0
SWH 64102.563 Hz
FIDRES 1.956255 Hz
AQ 0.2555904 sec
RG 2050
DW 7.800 usec
DE 6.00 usec
TE 299.4 K
D1 2.00000000 sec
TD0 1

======== CHANNEL f1 ===
NUC1 31P
P1 9.10 usec
PL1 0 dB
PL1W 24.94303322 W
SFO1 161.9727429 MHz

F2 – Processing parameters
SI 16384
SF 161.9755930 MHz
WDW EM
SSB 0
LB 5.00 Hz
GB 0
PC 1.40

^1H NMR ($CDCl_3$): **3b**

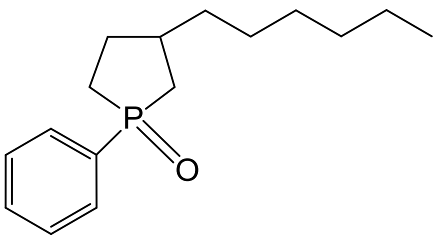

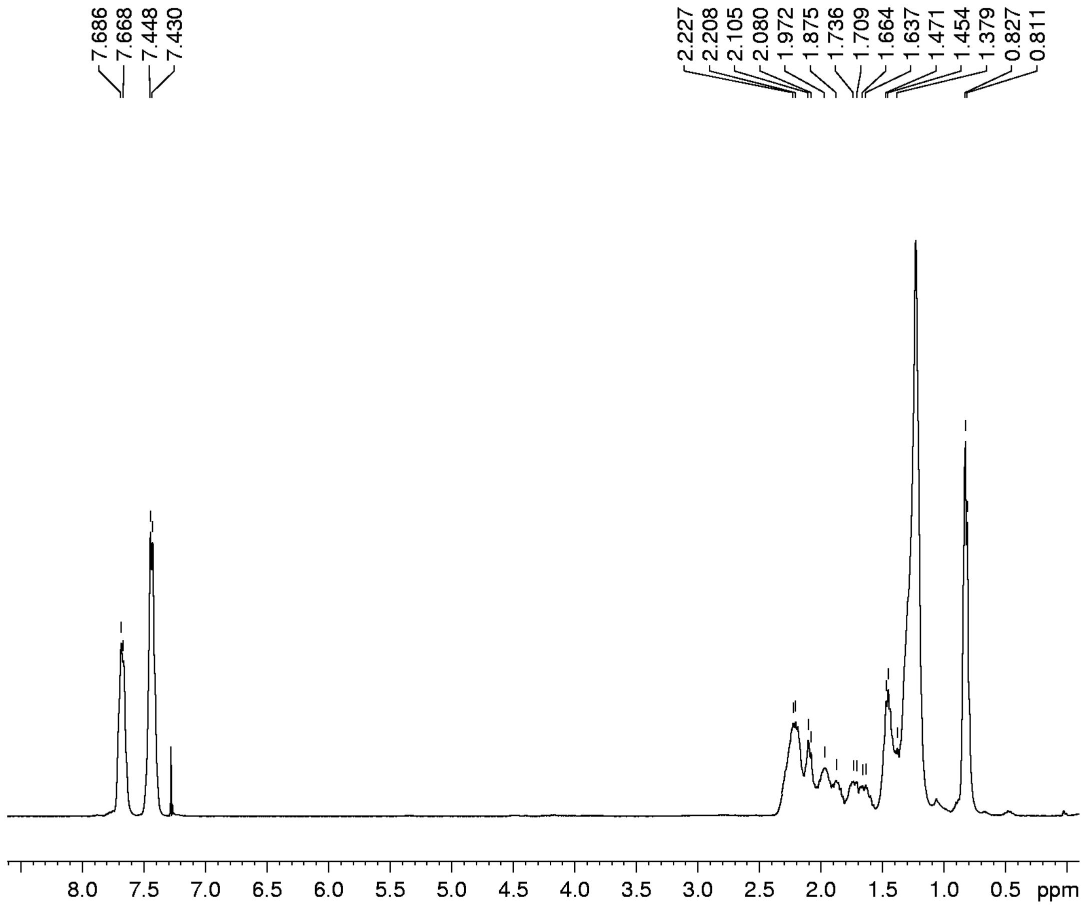

Current Data Parameters
NAME mahamathanova
EXPNO 26
PROCNO 1

F2 - Acquisition Parameters
Date_ 20110428
Time 19.58
INSTRUM spect
PROBHD 5 mm PABBO BB
PULPROG zg
TD 16384
SOLVENT CDCl3
NS 1
DS 0
SWH 3612.717 Hz
FIDRES 0.220503 Hz
AQ 2.2675457 sec
RG 14.2
DW 138.400 usec
DE 6.00 usec
TE 297.6 K
D1 5.00000000 sec
TD0 1

======== CHANNEL f1 ===
NUC1 1H
P1 14.80 usec
PL1 0 dB
PL1W 8.86695957 W
SFO1 400.1317676 MHz

F2 - Processing parameters
SI 32768
SF 400.1300000 MHz
WDW no
SSB 0
LB 0 Hz
GB 0
PC 1.00

^{13}C NMR ($CDCl_3$): **3b**

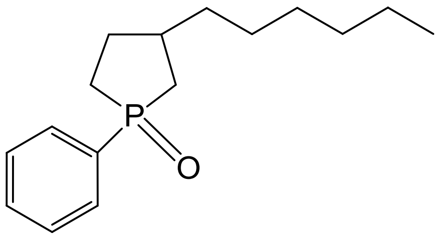

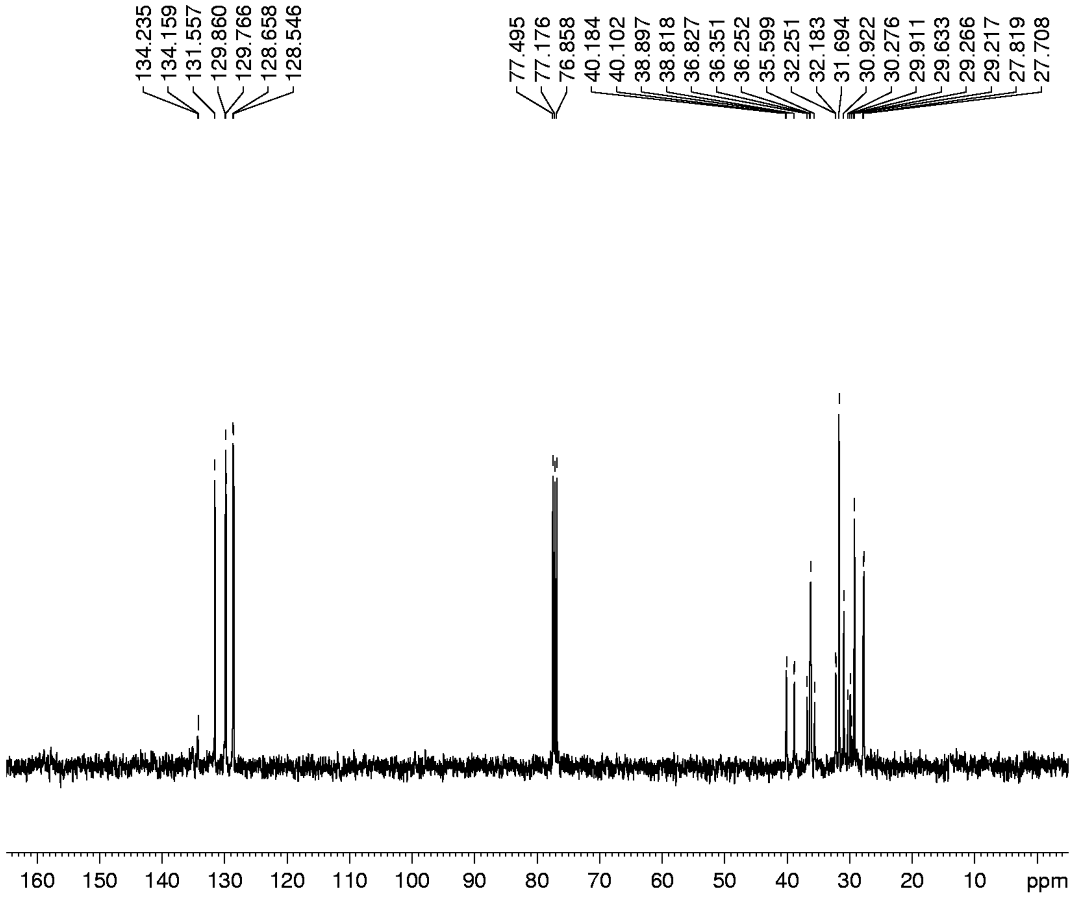

Current Data Parameters
NAME mahamathanova
EXPNO 28
PROCNO 1

F2 - Acquisition Parameters
Date_ 20110428
Time 20.02
INSTRUM spect
PROBHD 5 mm PABBO BB-
PULPROG zgpg30
TD 32144
SOLVENT CDCl3
NS 63
DS 0
SWH 23809.523 Hz
FIDRES 0.740714 Hz
AQ 0.6750240 sec
RG 2050
DW 21.000 usec
DE 30.00 usec
TE 297.7 K
D1 1.00000000 sec
D11 0.03000000 sec
TD0 1

======== CHANNEL f1 ====
NUC1 13C
P1 10.00 usec
PL1 0 dB
PL1W 33.91046524 W
SFO1 100.6232301 MHz

======== CHANNEL f2 ====
CPDPRG[2 waltz16
NUC2 1H
PCPD2 90.00 usec
PL2 0 dB
PL12 15.68 dB
PL13 18.70 dB
PL2W 8.86695957 W
PL12W 0.23975886 W
PL13W 0.11961196 W
SFO2 400.1317606 MHz

F2 - Processing parameters
SI 32768
SF 100.6127690 MHz
WDW EM
SSB 0

^{31}P NMR ($CDCl_3$): **3b**

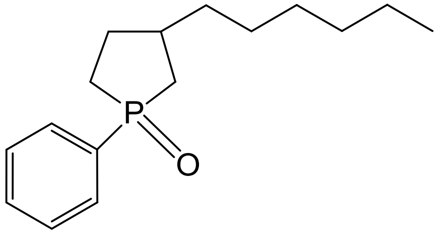

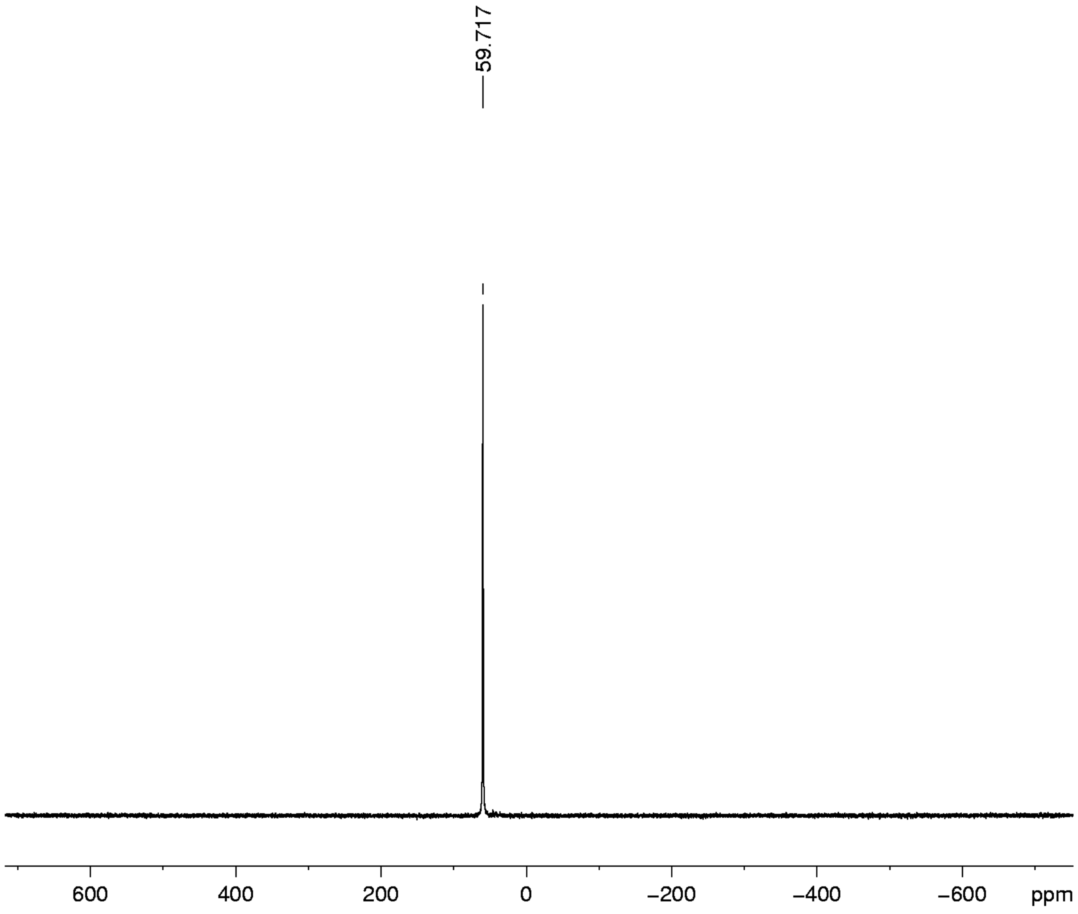

Current Data Parameters
NAME mahamathanova
EXPNO 30
PROCNO 1

F2 – Acquisition Parameters
Date_ 20110428
Time 20.07
INSTRUM spect
PROBHD 5 mm PABBO BB-
PULPROG zg
TD 32768
SOLVENT CDCl3
NS 13
DS 0
SWH 238095.234 Hz
FIDRES 7.266090 Hz
AQ 0.0688128 sec
RG 1820
DW 2.100 usec
DE 6.00 usec
TE 297.6 K
D1 2.00000000 sec
TD0 1

======== CHANNEL f1 ====
NUC1 31P
P1 9.10 usec
PL1 0 dB
PL1W 24.94303322 W
SFO1 161.9727429 MHz

F2 – Processing parameters
SI 16384
SF 161.9755930 MHz
WDW EM
SSB 0
LB 5.00 Hz
GB 0
PC 1.40

^{1}H NMR ($CDCl_3$): **3c**

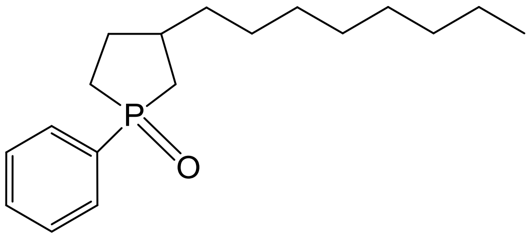

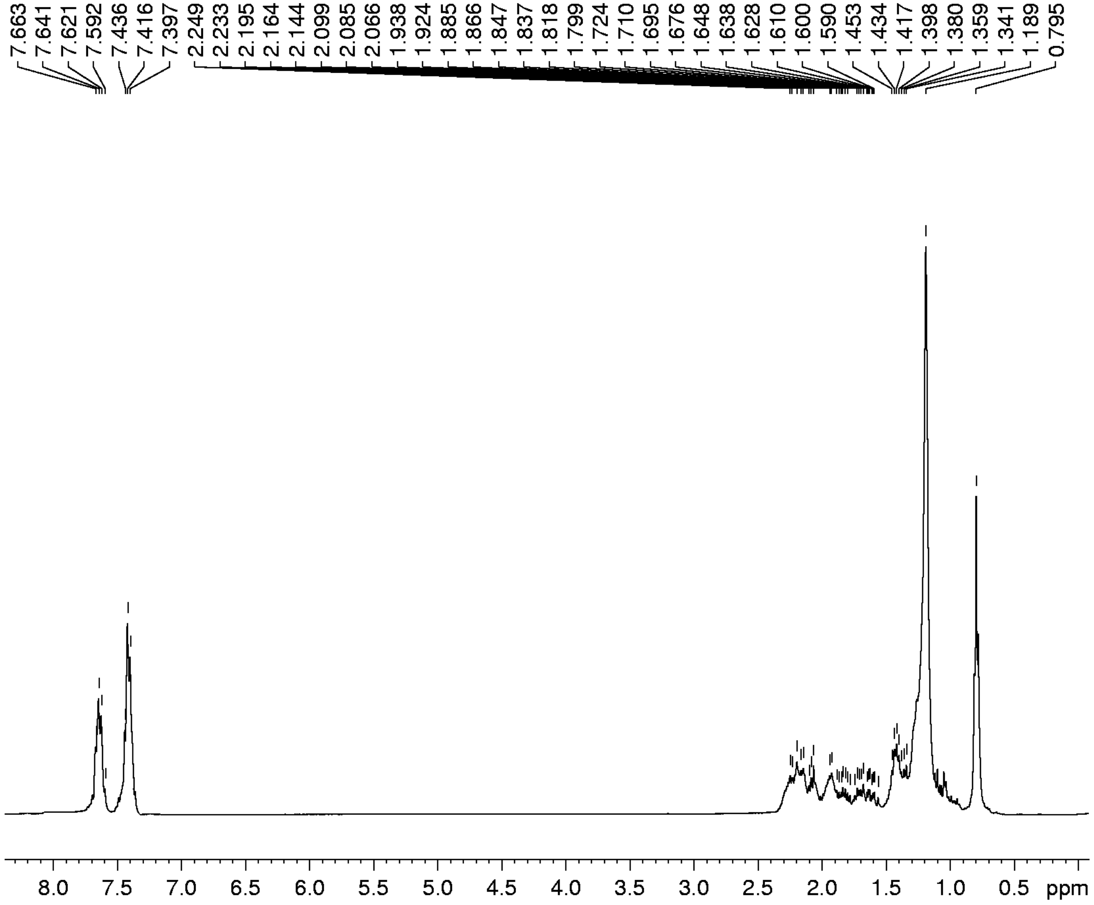

Current Data Parameters
NAME mahamathanova
EXPNO 32
PROCNO 1

F2 – Acquisition Parameters
Date_ 20110428
Time 20.20
INSTRUM spect
PROBHD 5 mm PABBO BE
PULPROG zg
TD 16384
SOLVENT CDCl3
NS 1
DS 0
SWH 3612.717 Hz
FIDRES 0.220503 Hz
AQ 2.2675457 sec
RG 10
DW 138.400 usec
DE 6.00 usec
TE 297.5 K
D1 5.00000000 sec
TD0 1

======== CHANNEL f1 ===
NUC1 1H
P1 14.80 usec
PL1 0 dB
PL1W 8.86695957 W
SFO1 400.1317676 MH:

F2 – Processing parameters
SI 32768
SF 400.1300000 MHz
WDW no
SSB 0
LB 0 Hz
GB 0
PC 1.00

^{13}C NMR ($CDCl_3$): **3c**

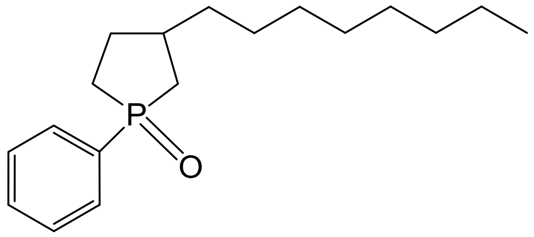

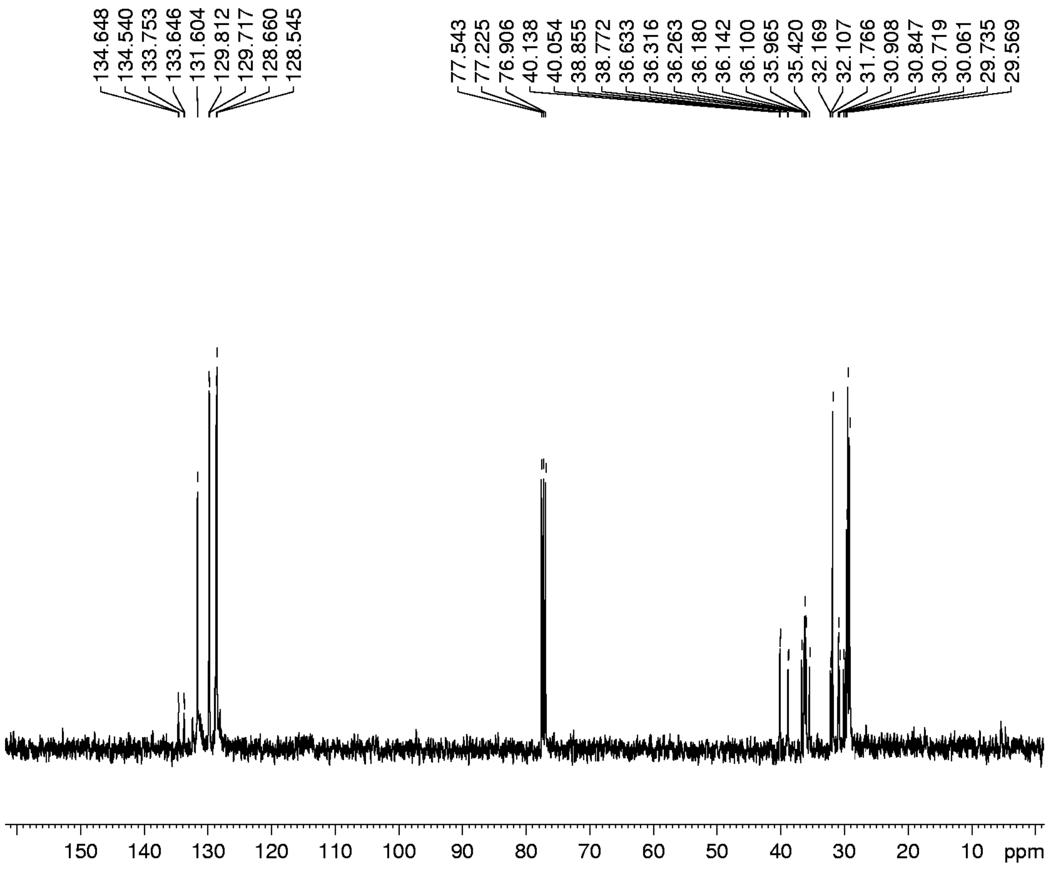

Current Data Parameters
NAME mahamathanova
EXPNO 34
PROCNO 1

F2 - Acquisition Parameters
Date_ 20110428
Time 20.22
INSTRUM spect
PROBHD 5 mm PABBO BB-
PULPROG zgpg30
TD 32144
SOLVENT CDCl3
NS 36
DS 0
SWH 23809.523 Hz
FIDRES 0.740714 Hz
AQ 0.6750240 sec
RG 2050
DW 21.000 usec
DE 30.00 usec
TE 297.5 K
D1 1.00000000 sec
D11 0.03000000 sec
TD0 1

======== CHANNEL f1 ====
NUC1 13C
P1 10.00 usec
PL1 0 dB
PL1W 33.91046524 W
SFO1 100.6232301 MHz

======== CHANNEL f2 ====
CPDPRG[2 waltz16
NUC2 1H
PCPD2 90.00 usec
PL2 0 dB
PL12 15.68 dB
PL13 18.70 dB
PL2W 8.86695957 W
PL12W 0.23975886 W
PL13W 0.11961196 W
SFO2 400.1317606 MHz

F2 - Processing parameters
SI 32768
SF 100.6127690 MHz
WDW EM
SSB 0

^{31}P NMR ($CDCl_3$): **3c**

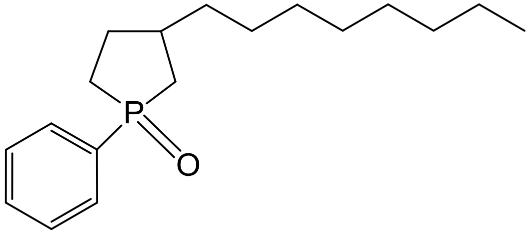

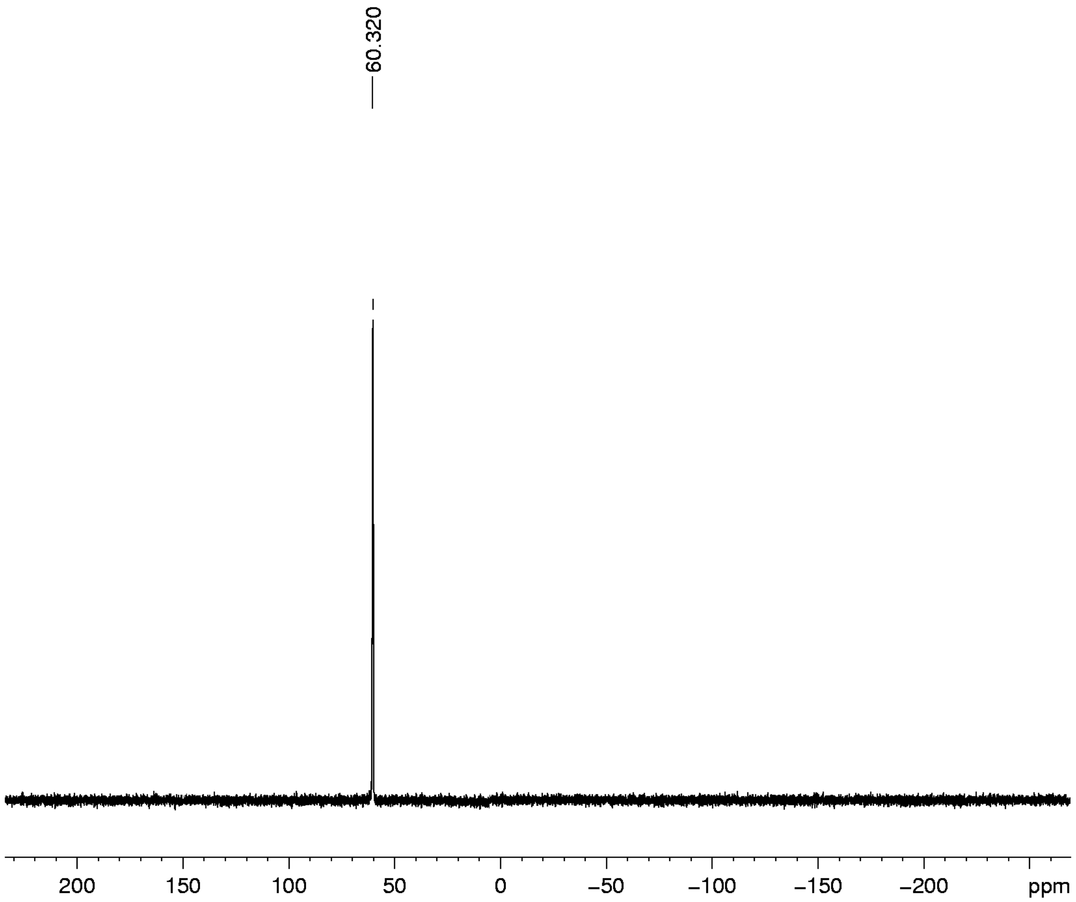

Current Data Parameters
NAME mahamathanova
EXPNO 35
PROCNO 1

F2 – Acquisition Parameters
Date_ 20110428
Time 20.17
INSTRUM spect
PROBHD 5 mm PABBO BB-
PULPROG zg
TD 32768
SOLVENT CDCl3
NS 3
DS 0
SWH 81521.742 Hz
FIDRES 2.487846 Hz
AQ 0.2009771 sec
RG 1820
DW 6.133 usec
DE 6.00 usec
TE 297.5 K
D1 2.00000000 sec
TD0 1

======== CHANNEL f1 ====
NUC1 31P
P1 9.10 usec
PL1 0 dB
PL1W 24.94303322 W
SFO1 161.9727429 MHz

F2 – Processing parameters
SI 16384
SF 161.9755930 MHz
WDW no
SSB 0
LB 0 Hz
GB 0
PC 1.40

^{1}H NMR ($CDCl_3$): **3d**

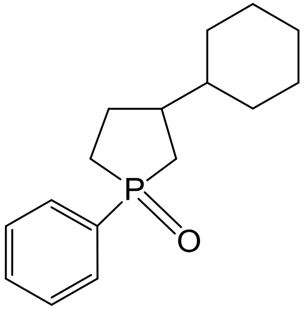

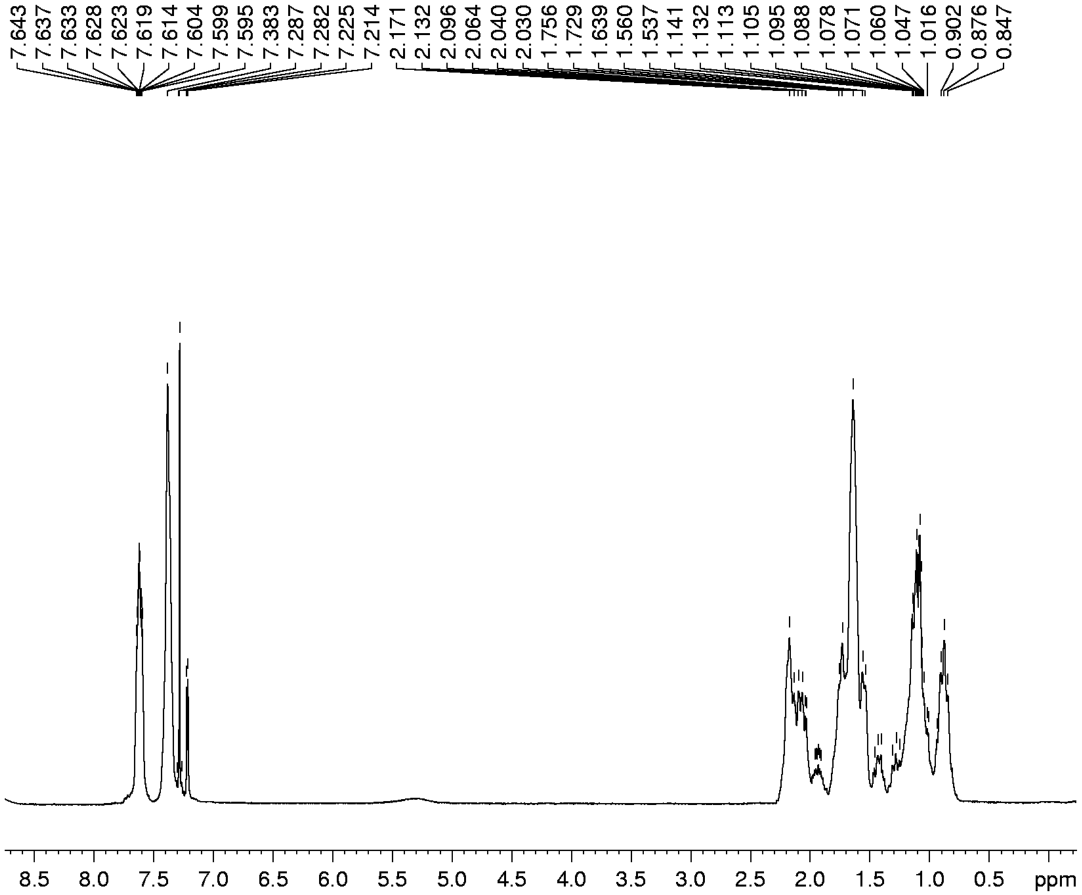

Current Data Parameters
NAME MRC–16
EXPNO 1
PROCNO 1

F2 – Acquisition Parameters
Date_ 20121207
Time 16.15
INSTRUM spect
PROBHD 5 mm PABBO BE
PULPROG zg30
TD 16384
SOLVENT CDCl3
NS 1
DS 0
SWH 3591.954 Hz
FIDRES 0.219235 Hz
AQ 2.2806528 sec
RG 25.4
DW 139.200 usec
DE 6.00 usec
TE 297.8 K
D1 1.00000000 sec
TD0 1

======== CHANNEL f1 ===
NUC1 1H
P1 14.80 usec
PL1 0 dB
PL1W 8.86695957 W
SFO1 400.1317034 MH:

F2 – Processing parameters
SI 32768
SF 400.1300000 MHz
WDW no
SSB 0
LB 0 Hz
GB 0
PC 1.00

^{13}C NMR ($CDCl_3$): **3d**

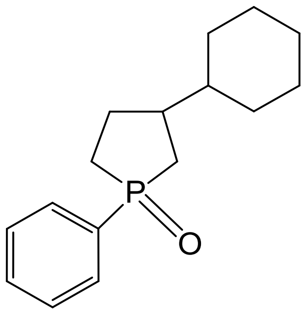

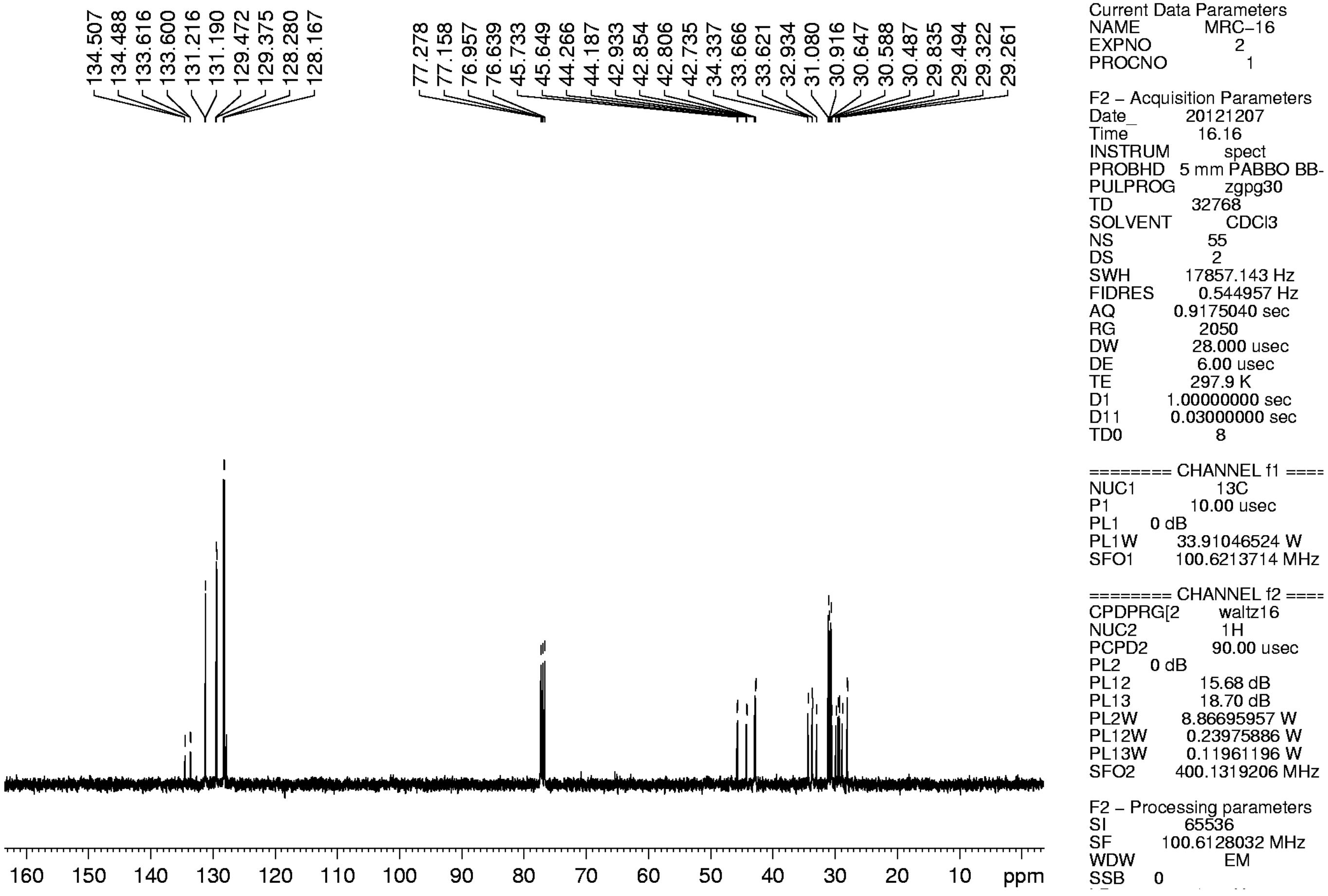

^{31}P NMR ($CDCl_3$): **3d**

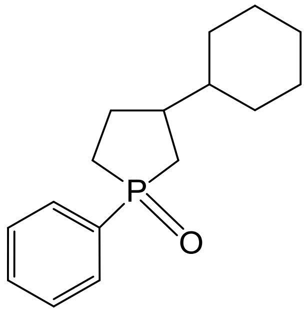

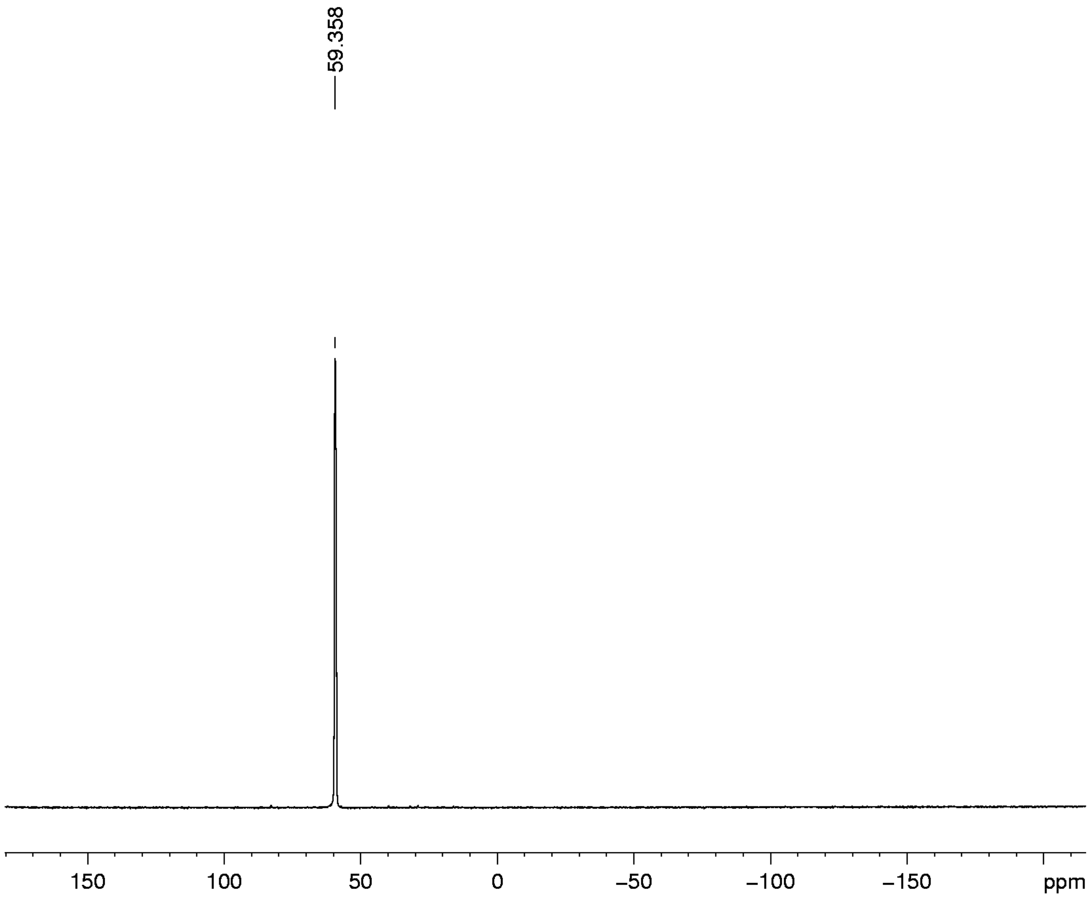

Current Data Parameters
NAME MRC−16
EXPNO 6
PROCNO 1

F2 − Acquisition Parameters
Date_ 20121207
Time 17.11
INSTRUM spect
PROBHD 5 mm PABBO B
PULPROG zg
TD 32768
SOLVENT CDCl3
NS 839
DS 0
SWH 64102.563 Hz
FIDRES 1.956255 Hz
AQ 0.2555904 sec
RG 1820
DW 7.800 usec
DE 6.00 usec
TE 297.9 K
D1 2.00000000 sec
TD0 1

======== CHANNEL f1 ==
NUC1 31P
P1 9.10 usec
PL1 0 dB
PL1W 24.94303322 W
SFO1 161.9727429 MH

F2 − Processing parameters
SI 16384
SF 161.9755930 MHz
WDW EM
SSB 0
LB 5.00 Hz
GB 0
PC 1.40

^{1}H NMR ($CDCl_3$): **3e**

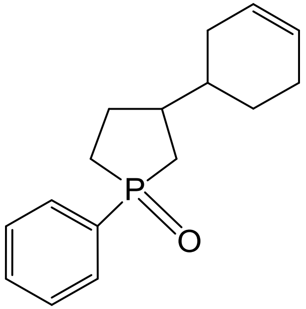

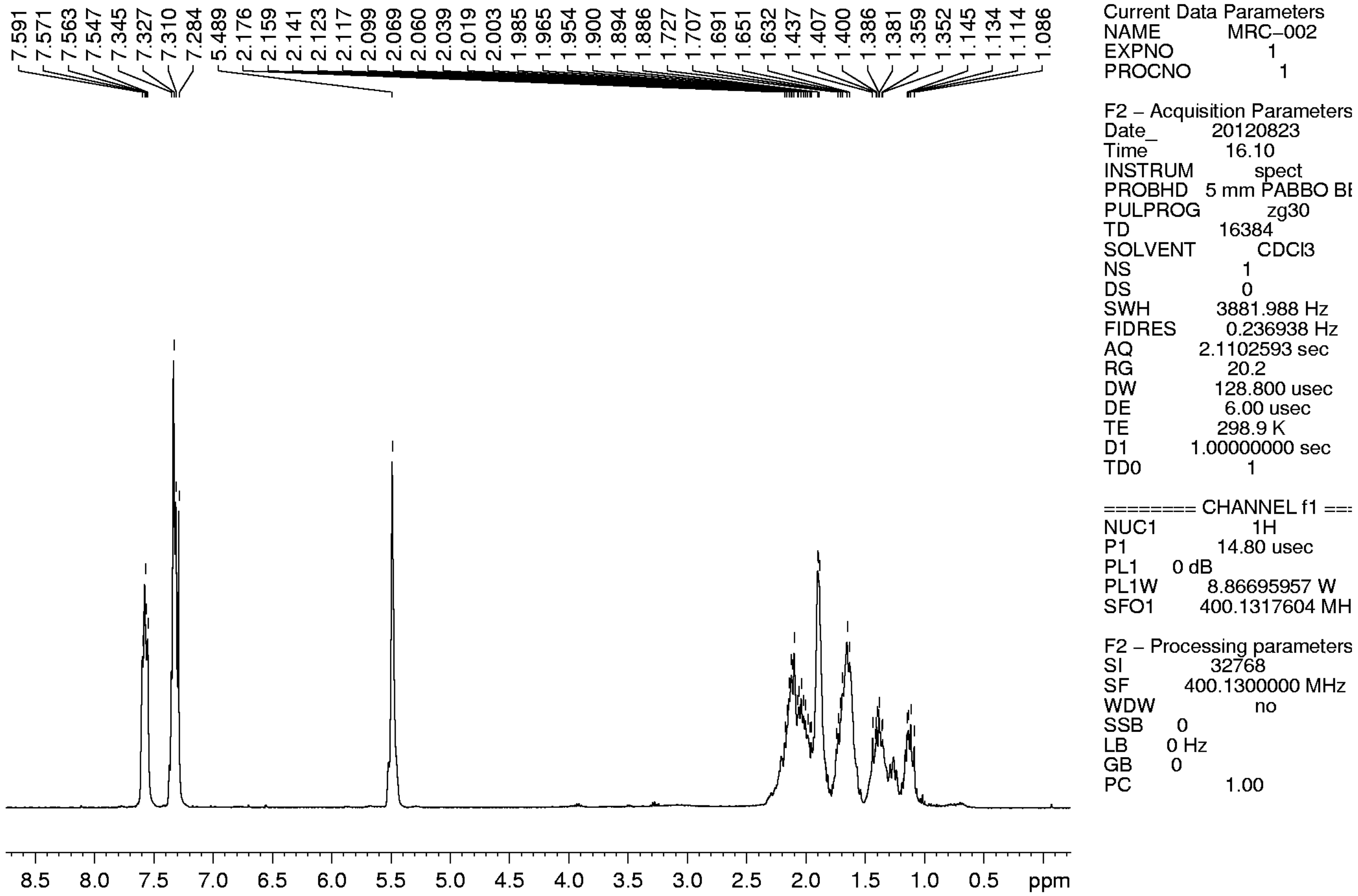

^{13}C NMR ($CDCl_3$): **3e**

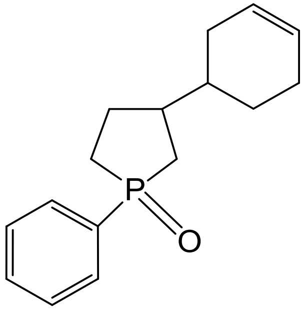

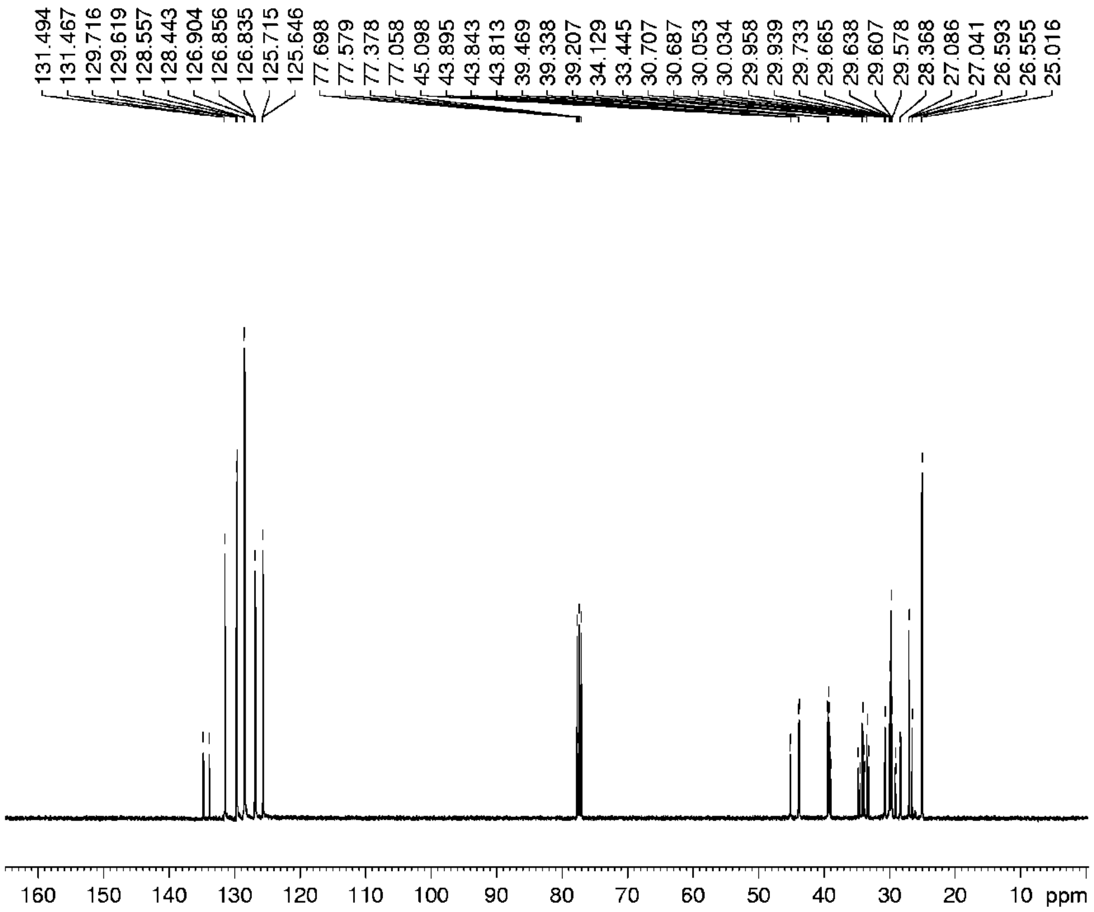

Current Data Parameters
NAME MRC-002
EXPNO 2
PROCNO 1

F2 - Acquisition Parameters
Date_ 20120823
Time 16.11
INSTRUM spect
PROBHD 5 mm PABBO BB-
PULPROG zgpg30
TD 32768
SOLVENT CDCl3
NS 275
DS 2
SWH 17857.143 Hz
FIDRES 0.544957 Hz
AQ 0.9175040 sec
RG 2050
DW 28.000 usec
DE 6.00 usec
TE 299.0 K
D1 1.00000000 sec
D11 0.03000000 sec
TD0 8

======== CHANNEL f1 =====
NUC1 13C
P1 10.00 usec
PL1 0 dB
PL1W 33.91046524 W
SFO1 100.6216730 MHz

======== CHANNEL f2 =====
CPDPRG[2 waltz16
NUC2 1H
PCPD2 90.00 usec
PL2 0 dB
PL12 15.68 dB
PL13 18.70 dB
PL2W 8.86695957 W
PL12W 0.23975886 W
PL13W 0.11961196 W
SFO2 400.1316005 MHz

F2 - Processing parameters
SI 65536
SF 100.6127727 MHz
WDW EM
SSB 0

^{31}P NMR ($CDCl_3$): **3e**

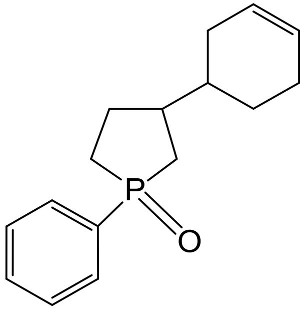

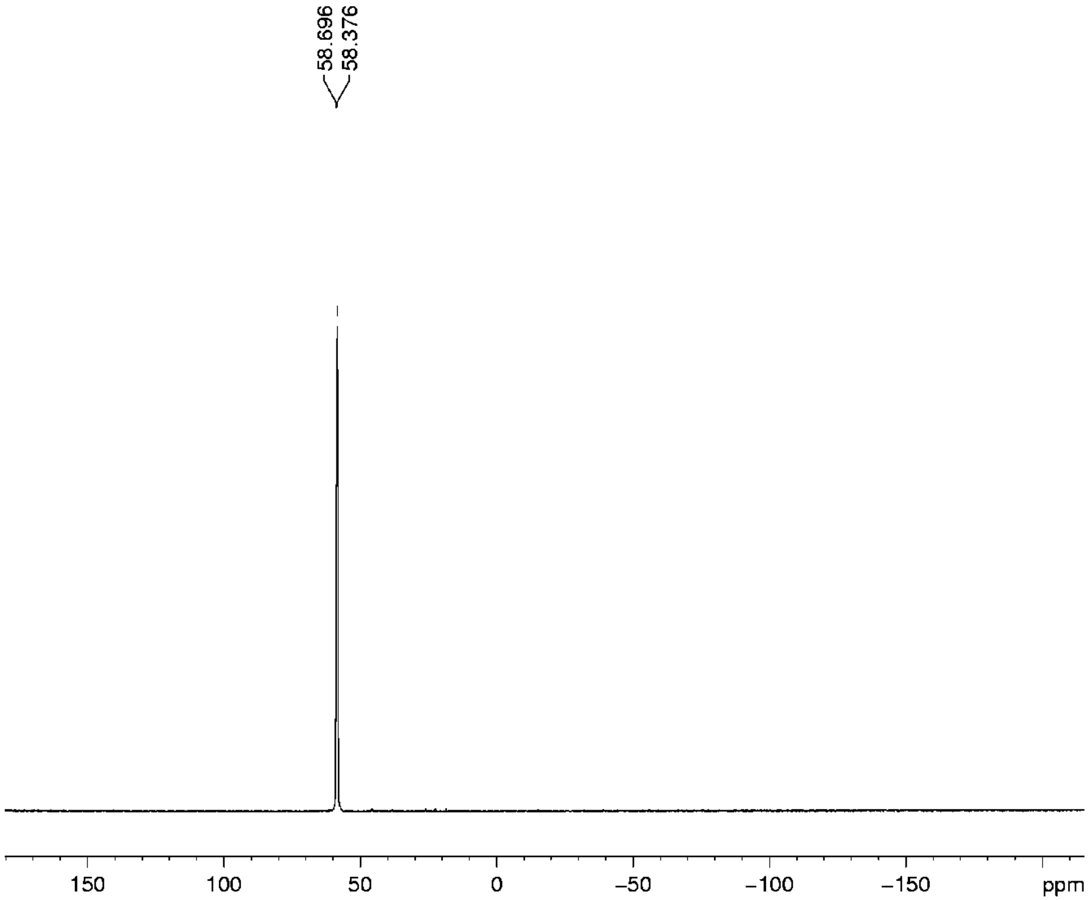

Current Data Parameters
NAME MRC−002
EXPNO 7
PROCNO 1

F2 − Acquisition Parameters
Date_ 20120823
Time 17.13
INSTRUM spect
PROBHD 5 mm PABBO BB
PULPROG zg
TD 32768
SOLVENT CDCl3
NS 13
DS 0
SWH 64102.563 Hz
FIDRES 1.956255 Hz
AQ 0.2555904 sec
RG 2050
DW 7.800 usec
DE 6.00 usec
TE 299.2 K
D1 2.00000000 sec
TD0 1

======== CHANNEL f1 ===
NUC1 31P
P1 9.10 usec
PL1 0 dB
PL1W 24.94303322 W
SFO1 161.9727429 MHz

F2 − Processing parameters
SI 16384
SF 161.9755930 MHz
WDW EM
SSB 0
LB 5.00 Hz
GB 0
PC 1.40

^{1}H NMR ($CDCl_3$): **3f**

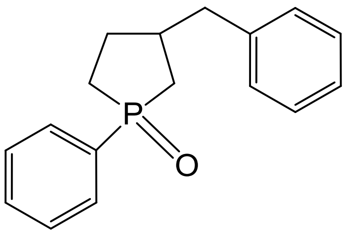

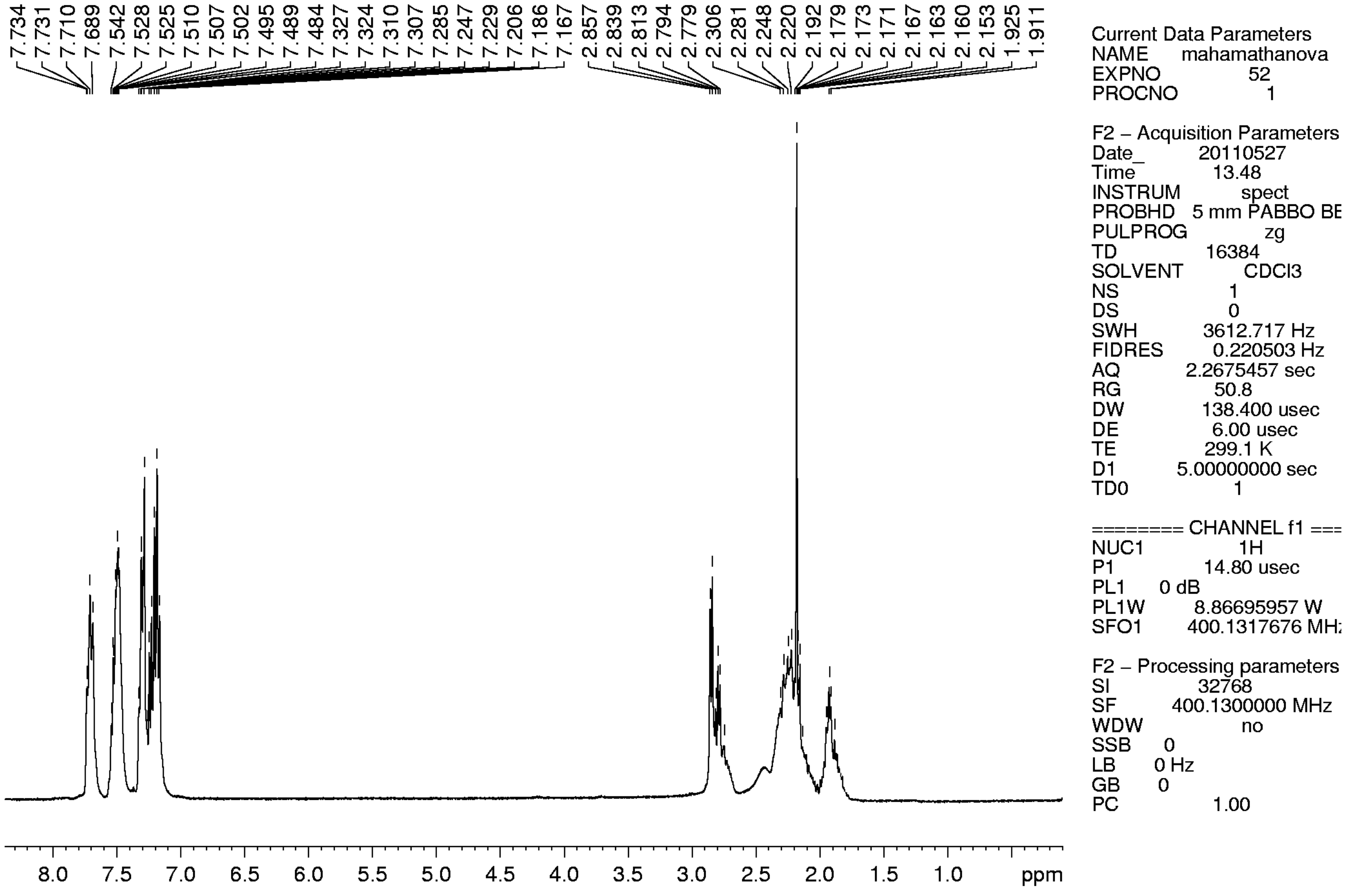

^{13}C NMR ($CDCl_3$): **3f**

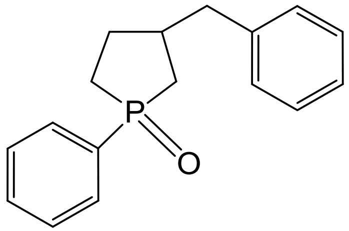

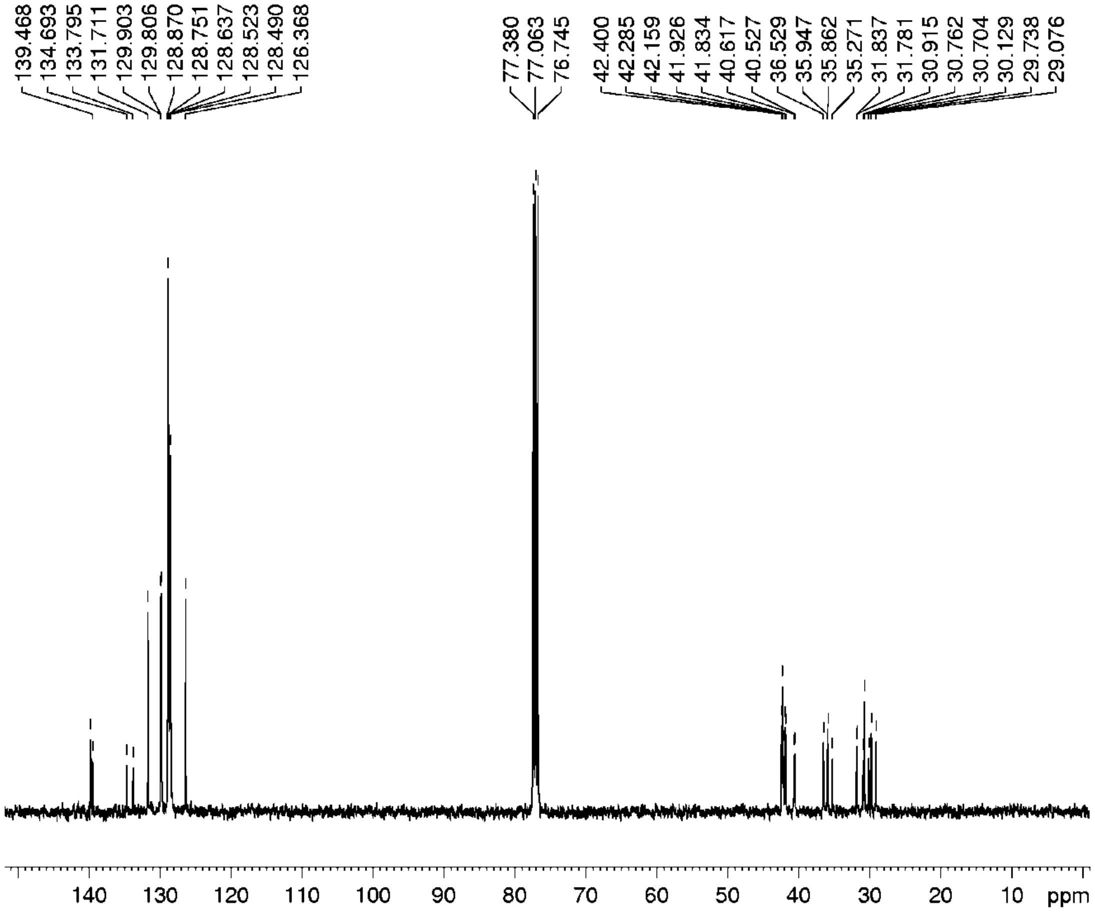

Current Data Parameters
NAME mahamathanova
EXPNO 53
PROCNO 1

F2 - Acquisition Parameters
Date_ 20110527
Time 14.44
INSTRUM spect
PROBHD 5 mm PABBO BB-
PULPROG zgpg30
TD 32144
SOLVENT CDCl3
NS 634
DS 0
SWH 16891.893 Hz
FIDRES 0.525507 Hz
AQ 0.9514624 sec
RG 2050
DW 29.600 usec
DE 30.00 usec
TE 300.0 K
D1 1.00000000 sec
D11 0.03000000 sec
TD0 1

======== CHANNEL f1 ====
NUC1 13C
P1 10.00 usec
PL1 0 dB
PL1W 33.91046524 W
SFO1 100.6199552 MHz

======== CHANNEL f2 ====
CPDPRG[2 waltz16
NUC2 1H
PCPD2 90.00 usec
PL2 0 dB
PL12 15.68 dB
PL13 18.70 dB
PL2W 8.86695957 W
PL12W 0.23975886 W
PL13W 0.11961196 W
SFO2 400.1317606 MHz

F2 - Processing parameters
SI 32768
SF 100.6127690 MHz
WDW EM
SSB 0

^{31}P NMR ($CDCl_3$): **3f**

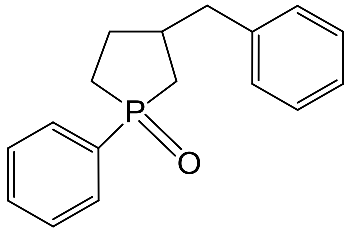

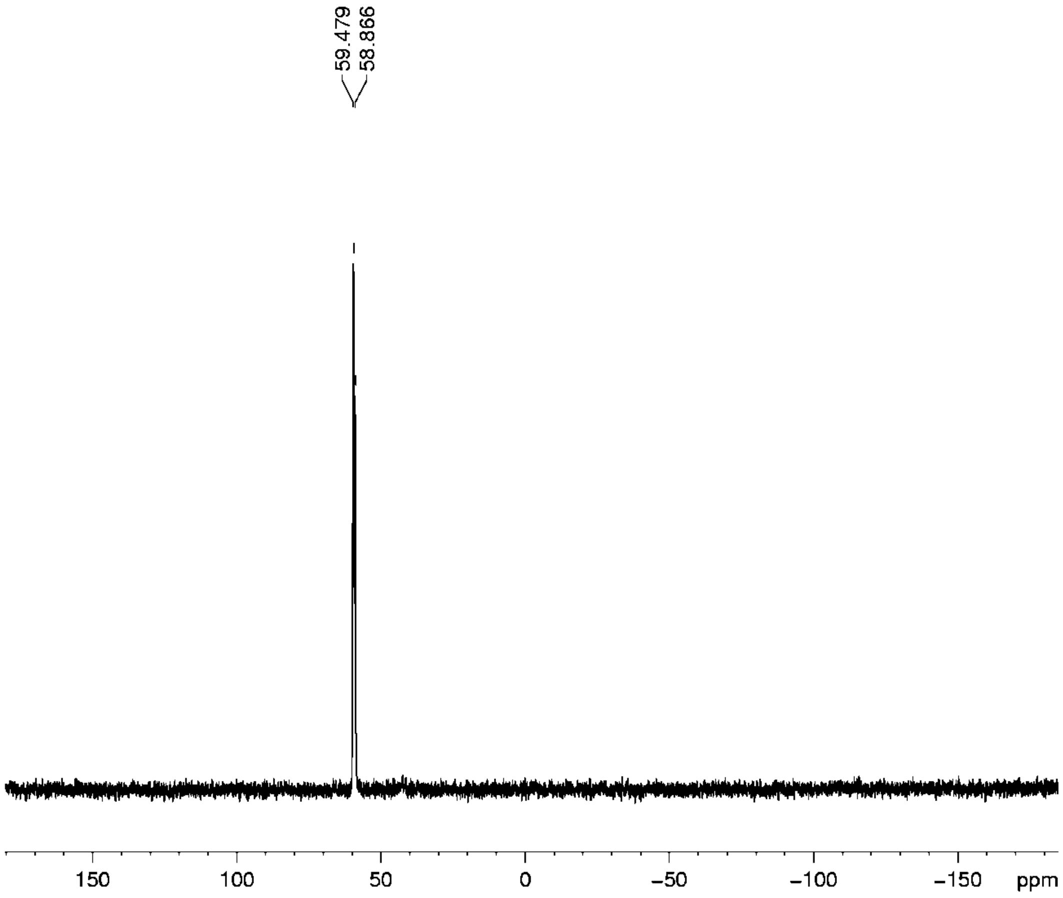

Current Data Parameters
NAME mahamathanova
EXPNO 58
PROCNO 1

F2 – Acquisition Parameters
Date_ 20110527
Time 14.47
INSTRUM spect
PROBHD 5 mm PABBO BB-
PULPROG zg
TD 32768
SOLVENT CDCl3
NS 6
DS 0
SWH 64102.563 Hz
FIDRES 1.956255 Hz
AQ 0.2555904 sec
RG 2050
DW 7.800 usec
DE 6.00 usec
TE 299.5 K
D1 2.00000000 sec
TD0 1

======== CHANNEL f1 ====
NUC1 31P
P1 9.10 usec
PL1 0 dB
PL1W 24.94303322 W
SFO1 161.9727429 MHz

F2 – Processing parameters
SI 16384
SF 161.9755930 MHz
WDW EM
SSB 0
LB 5.00 Hz
GB 0
PC 1.40

^{1}H NMR ($CDCl_3$): **3g**

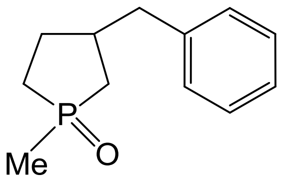

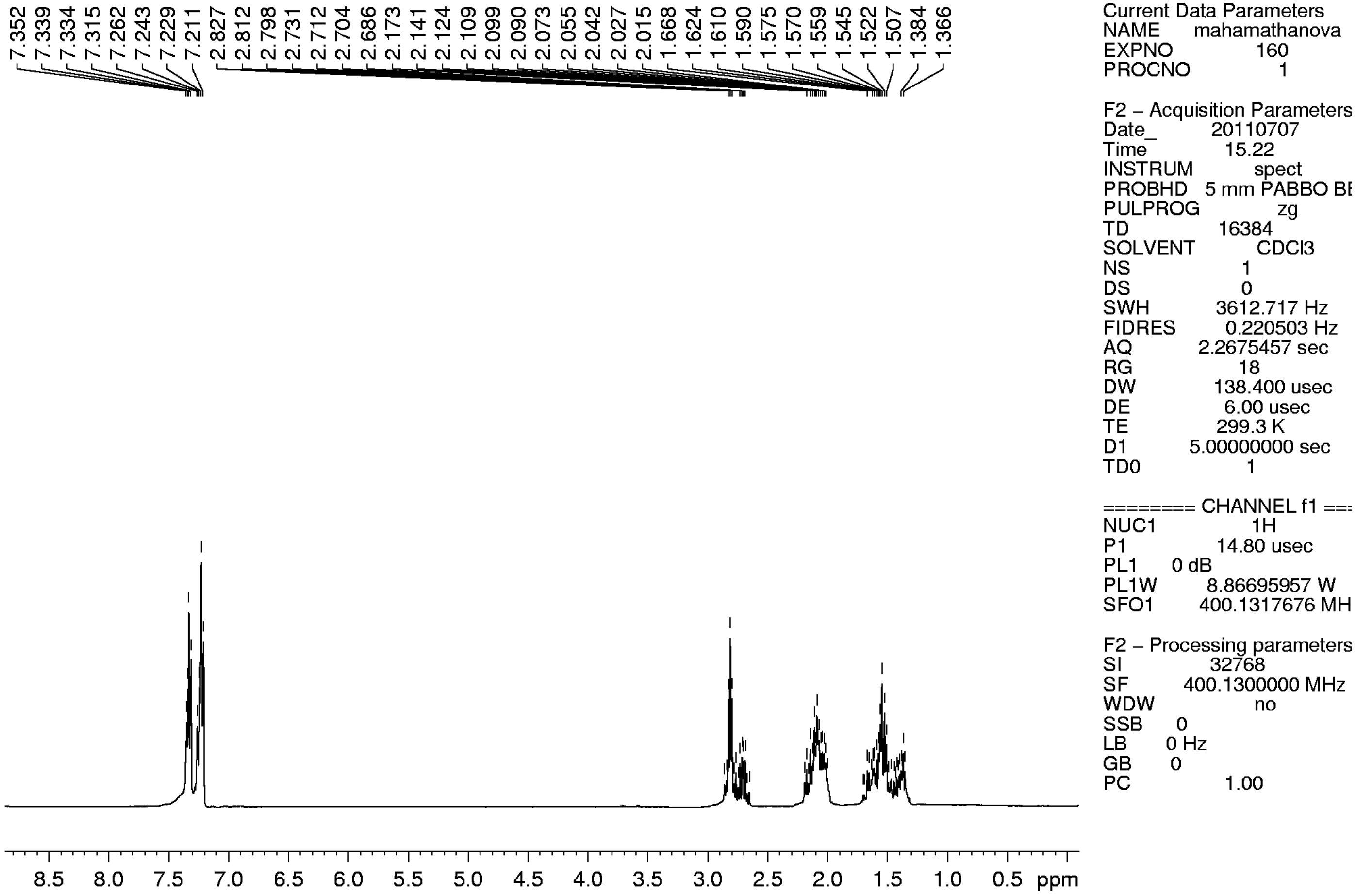

^{13}C NMR ($CDCl_3$): **3g**

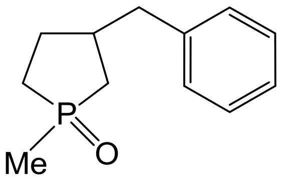

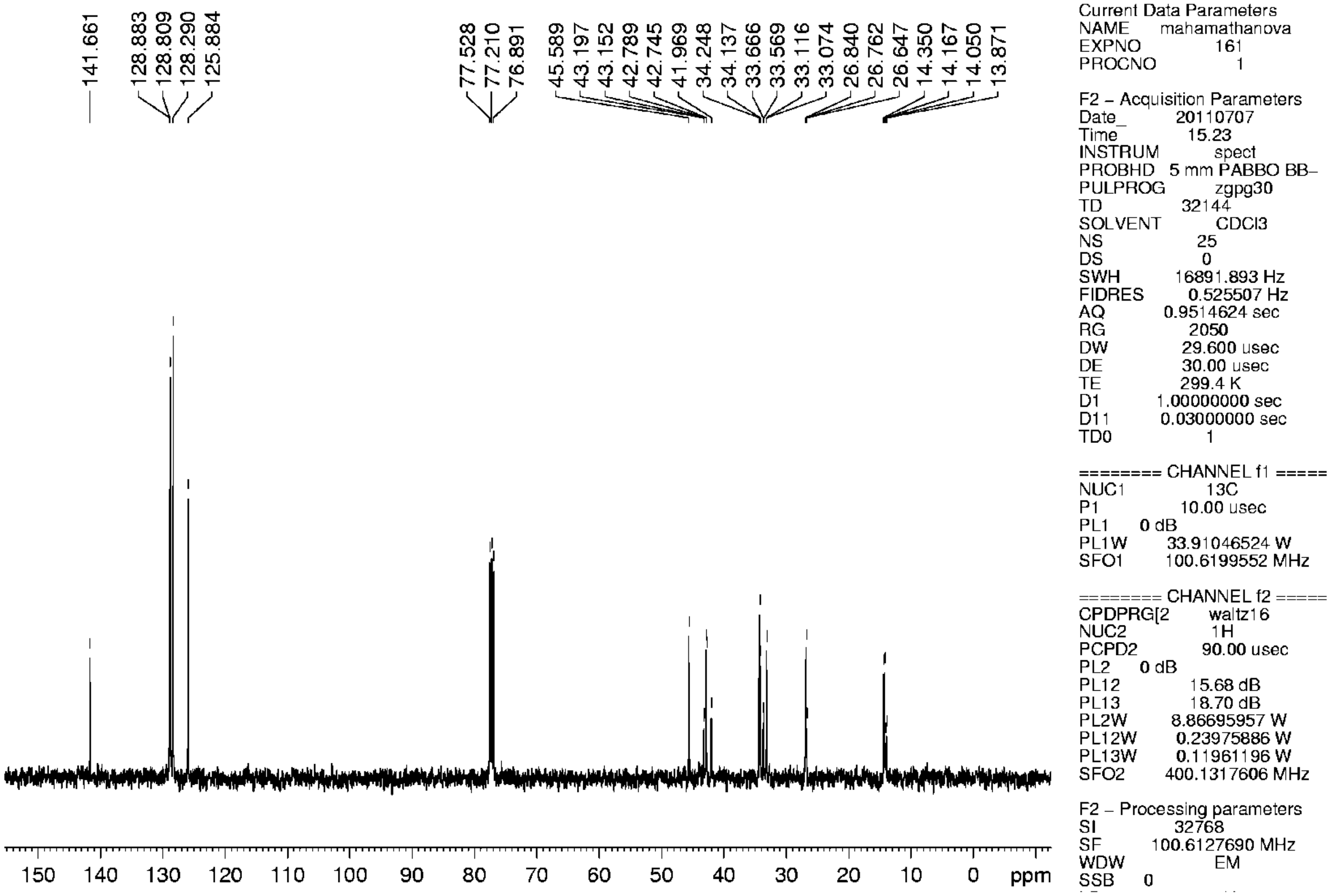

^{31}P NMR ($CDCl_3$): **3g**

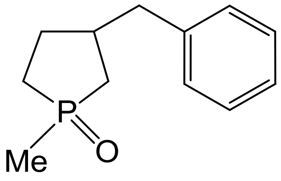

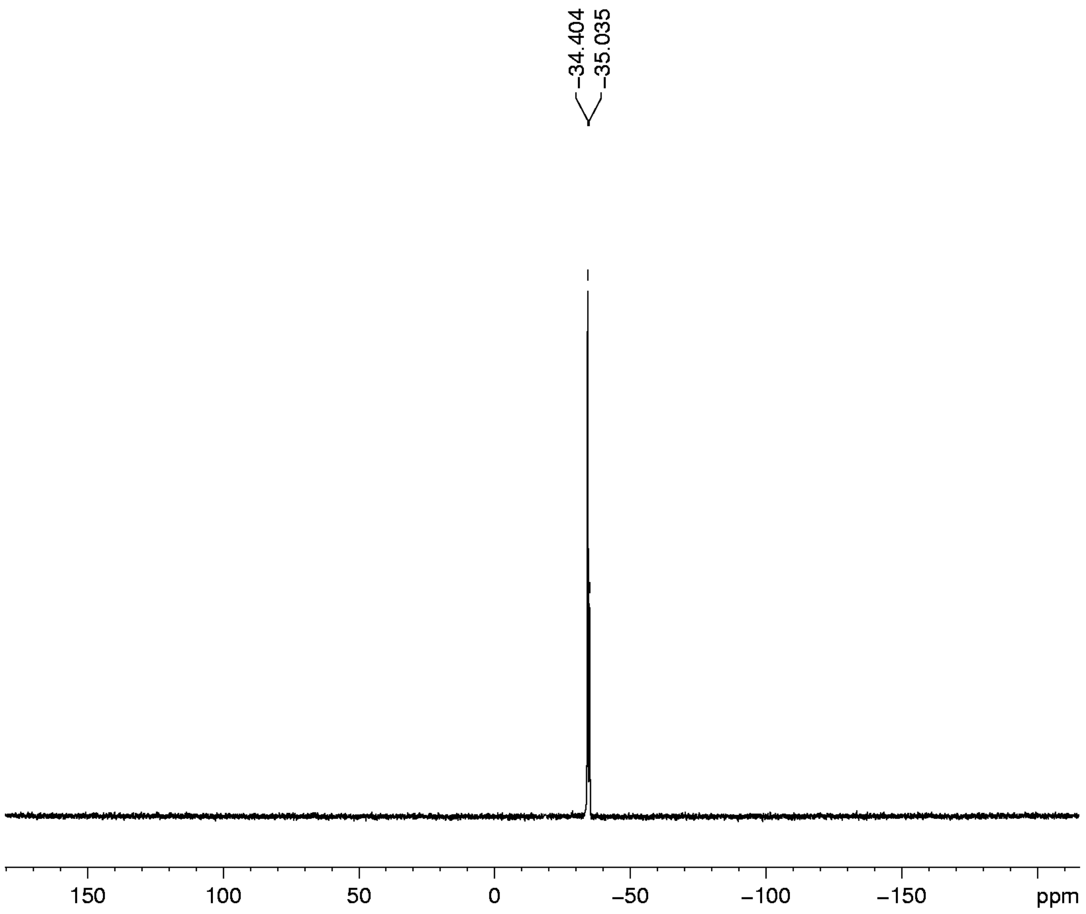

Current Data Parameters
NAME mahamathanova
EXPNO 165
PROCNO 1

F2 – Acquisition Parameters
Date_ 20110707
Time 15.56
INSTRUM spect
PROBHD 5 mm PABBO BB-
PULPROG zg
TD 32768
SOLVENT CDCl3
NS 9
DS 0
SWH 64102.563 Hz
FIDRES 1.956255 Hz
AQ 0.2555904 sec
RG 1290
DW 7.800 usec
DE 6.00 usec
TE 299.2 K
D1 2.00000000 sec
TD0 1

======== CHANNEL f1 ====
NUC1 31P
P1 9.10 usec
PL1 0 dB
PL1W 24.94303322 W
SFO1 161.9727429 MHz

F2 – Processing parameters
SI 16384
SF 161.9755930 MHz
WDW EM
SSB 0
LB 5.00 Hz
GB 0
PC 1.40

^{1}H NMR ($CDCl_3$): **3h**

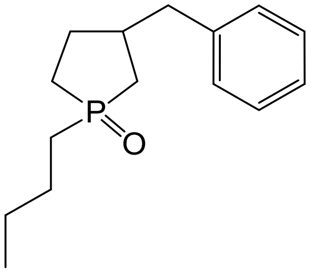

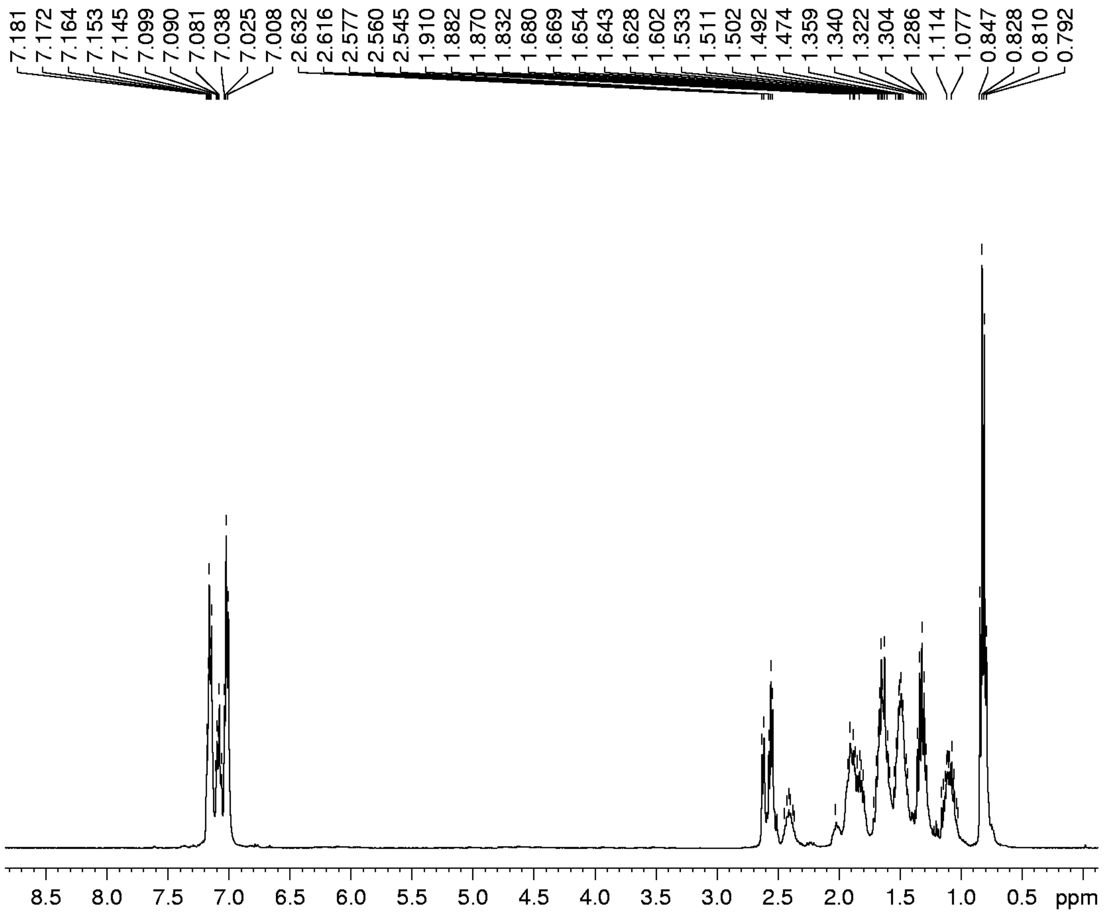

Current Data Parameters
NAME SRE–7
EXPNO 1
PROCNO 1

F2 – Acquisition Parameters
Date_ 20120517
Time 14.01
INSTRUM spect
PROBHD 5 mm PABBO BE
PULPROG zg30
TD 16384
SOLVENT CDCl3
NS 1
DS 0
SWH 4618.227 Hz
FIDRES 0.281874 Hz
AQ 1.7738410 sec
RG 16
DW 108.267 usec
DE 6.00 usec
TE 298.7 K
D1 1.00000000 sec
TD0 1

======== CHANNEL f1 ===
NUC1 1H
P1 14.80 usec
PL1 0 dB
PL1W 8.86695957 W
SFO1 400.1319527 MH:

F2 – Processing parameters
SI 32768
SF 400.1300000 MHz
WDW EM
SSB 0
LB 0.10 Hz
GB 0
PC 1.00

^{13}C NMR ($CDCl_3$): **3h**

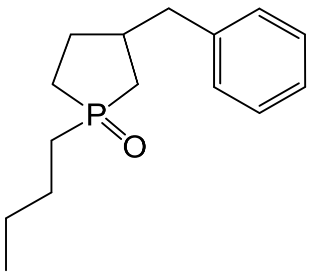

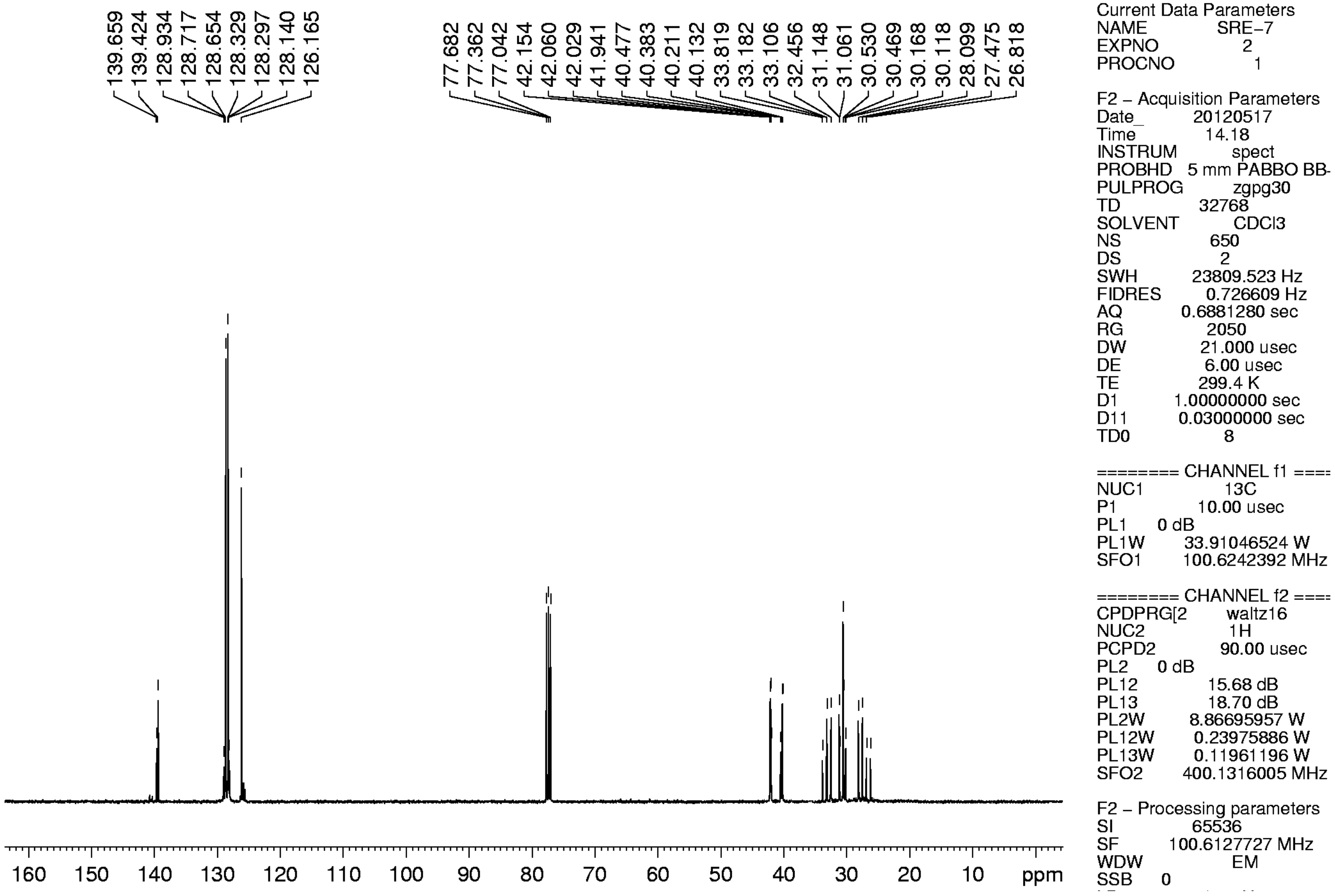

^{31}P NMR ($CDCl_3$): **3h**

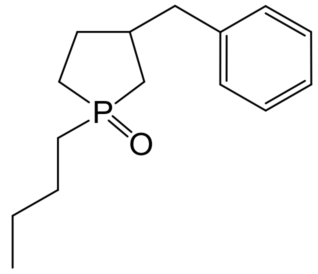

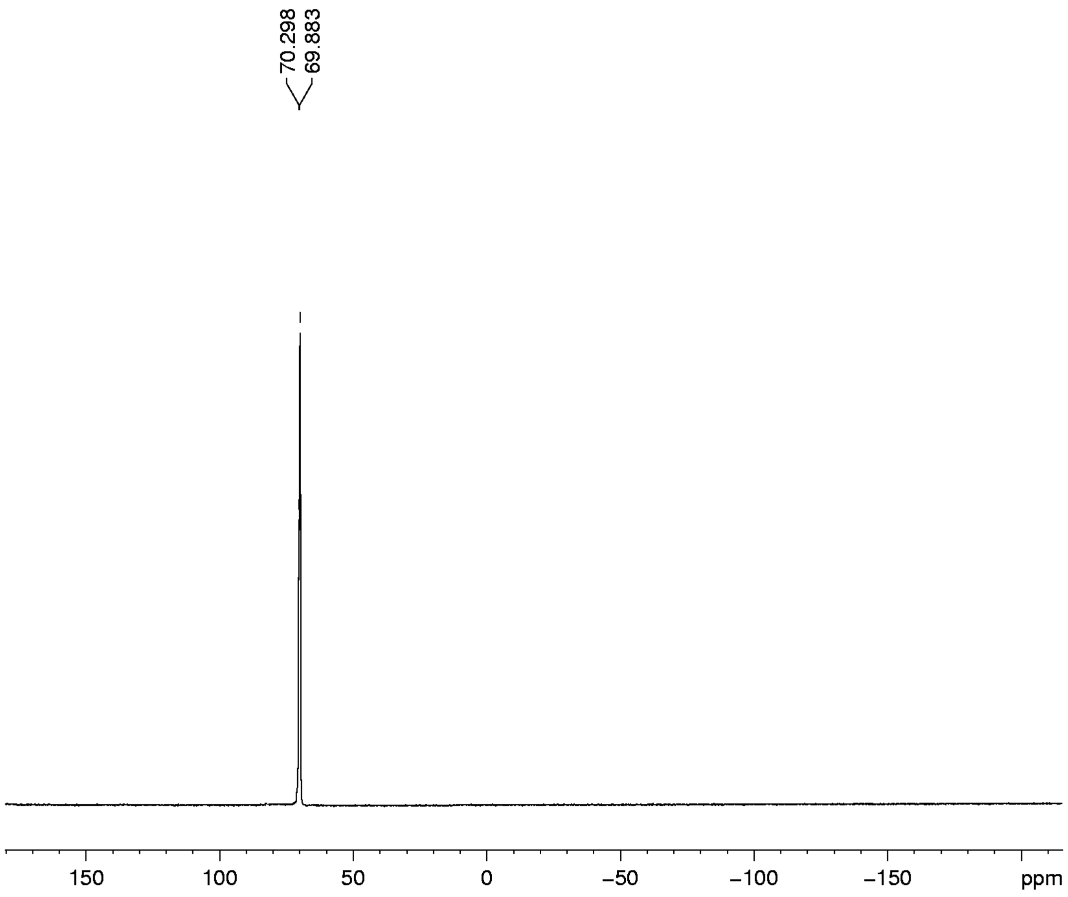

Current Data Parameters
NAME SRE−7
EXPNO 7
PROCNO 1

F2 − Acquisition Parameters
Date_ 20120517
Time 15.01
INSTRUM spect
PROBHD 5 mm PABBO BB−
PULPROG zg
TD 32768
SOLVENT CDCl3
NS 34
DS 0
SWH 64102.563 Hz
FIDRES 1.956255 Hz
AQ 0.2555904 sec
RG 2050
DW 7.800 usec
DE 6.00 usec
TE 298.8 K
D1 2.00000000 sec
TD0 1

======== CHANNEL f1 =====
NUC1 31P
P1 9.10 usec
PL1 0 dB
PL1W 24.94303322 W
SFO1 161.9727429 MHz

F2 − Processing parameters
SI 16384
SF 161.9755930 MHz
WDW EM
SSB 0
LB 5.00 Hz
GB 0
PC 1.40

^1H NMR ($CDCl_3$): **7e, 8e**

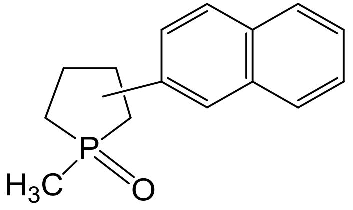

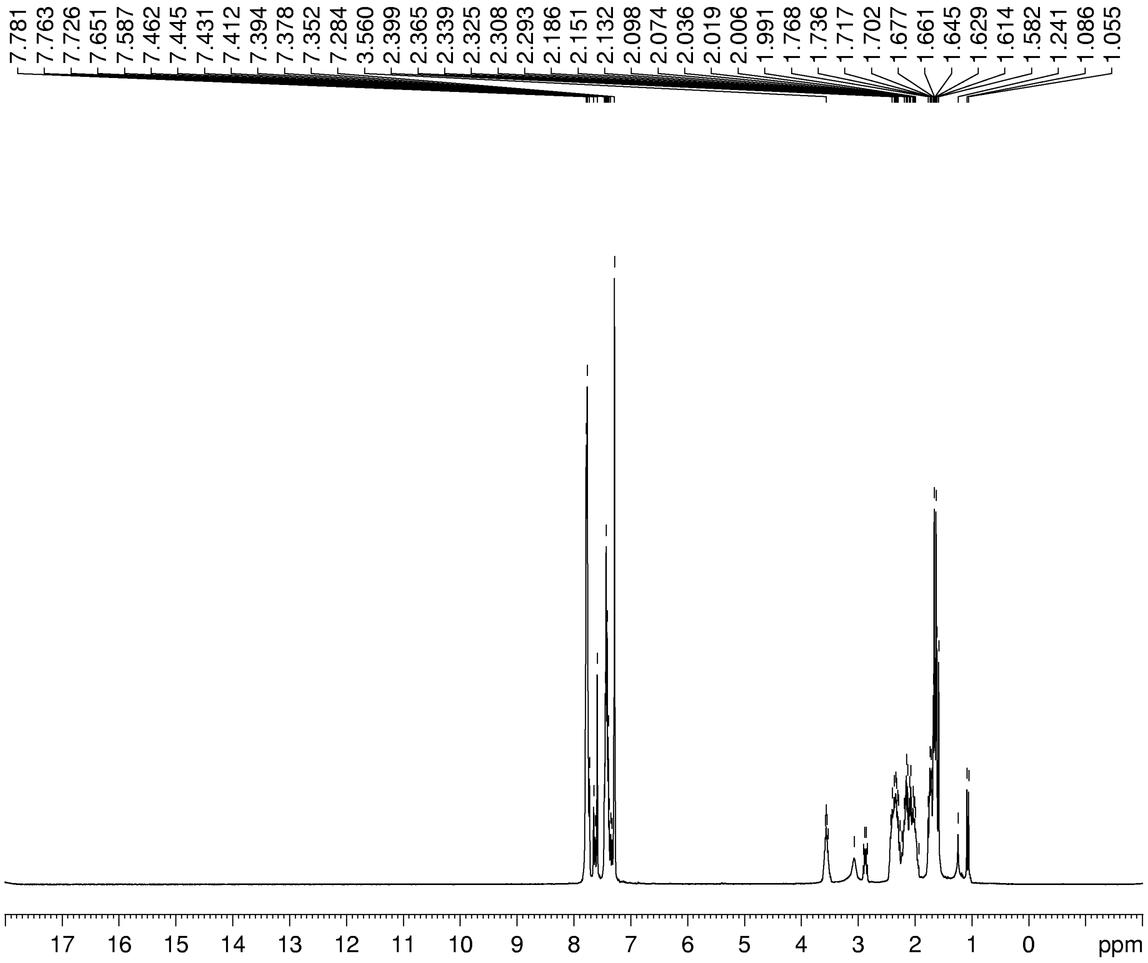

Current Data Parameters
NAME KRA-342-30
EXPNO 1
PROCNO 1

F2 - Acquisition Parameters
Date_ 20150514
Time 15.33
INSTRUM spect
PROBHD 5 mm PABBO BB-
PULPROG zg30
TD 16384
SOLVENT CDCl3
NS 1
DS 0
SWH 8012.820 Hz
FIDRES 0.489064 Hz
AQ 1.0223616 sec
RG 36
DW 62.400 usec
DE 6.50 usec
TE 297.9 K
D1 1.00000000 sec
TD0 1

======== CHANNEL f1 ====
SFO1 400.1332010 MHz
NUC1 1H
P1 14.80 usec
PLW1 8.89999962 W

F2 - Processing parameters
SI 16384
SF 400.1300000 MHz
WDW no
SSB 0
LB 0 Hz
GB 0
PC 1.00

^{13}C NMR ($CDCl_3$): **7e, 8e**

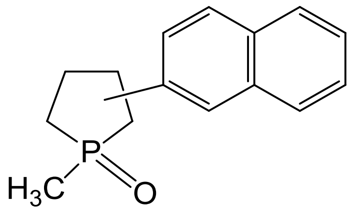

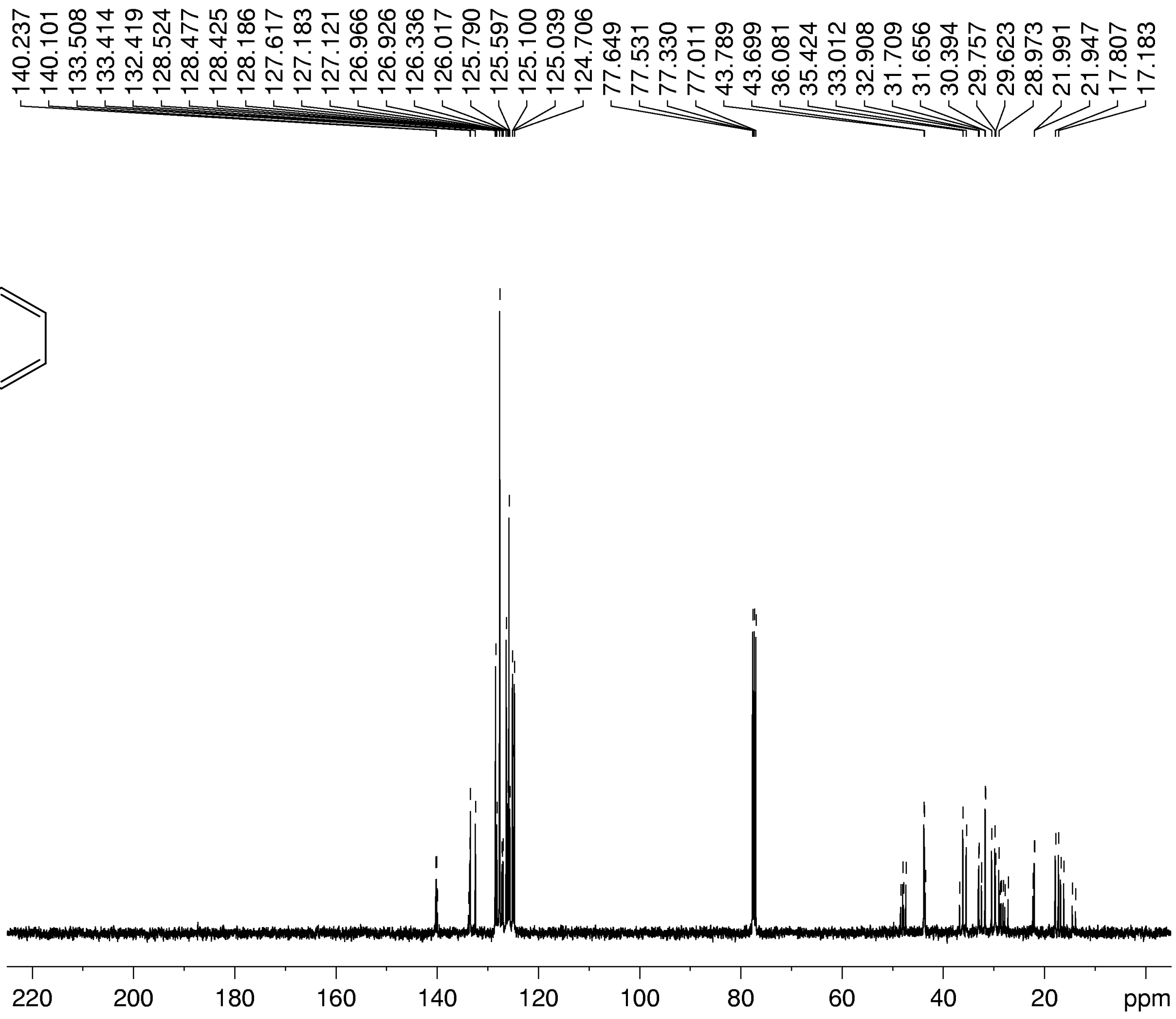

Current Data Parameters
NAME KRA−342−30
EXPNO 13
PROCNO 1

F2 − Acquisition Parameters
Date_ 20150514
Time 15.34
INSTRUM spect
PROBHD 5 mm PABBO BB−
PULPROG zgpg30
TD 32768
SOLVENT CDCl3
NS 96
DS 2
SWH 23148.148 Hz
FIDRES 0.706425 Hz
AQ 0.7077888 sec
RG 2050
DW 21.600 usec
DE 6.50 usec
TE 298.0 K
D1 1.00000000 sec
D11 0.03000000 sec
TD0 8

======== CHANNEL f1 =====
SFO1 100.6238364 MHz
NUC1 13C
P1 10.00 usec
PLW1 34.00000000 W

======== CHANNEL f2 =====
SFO2 400.1316005 MHz
NUC2 1H
CPDPRG[2 waltz16
PCPD2 90.00 usec
PLW2 8.89999962 W
PLW12 0.24067000 W
PLW13 0.19495000 W

F2 − Processing parameters
SI 65536
SF 100.6127689 MHz
WDW EM
SSB 0
LB 2.00 Hz
GB 0
PC [illegible]

^{31}P NMR ($CDCl_3$): **7e, 8e**

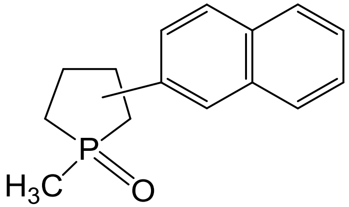

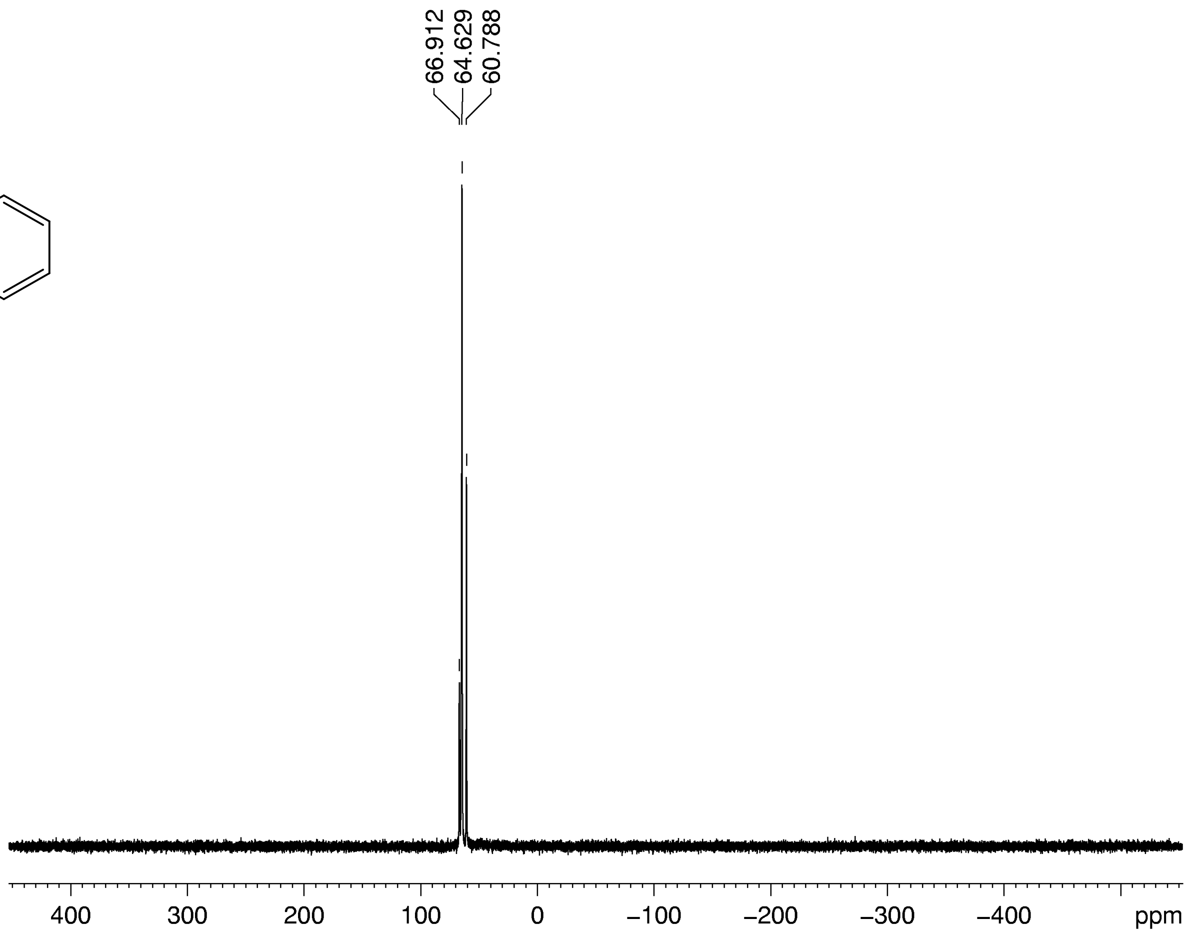

Current Data Parameters
NAME KRA−342−30
EXPNO 31
PROCNO 1

F2 − Acquisition Parameters
Date_ 20150514
Time 15.31
INSTRUM spect
PROBHD 5 mm PABBO BB−
PULPROG zg30
TD 65536
SOLVENT CDCl3
NS 32
DS 4
SWH 163043.484 Hz
FIDRES 2.487846 Hz
AQ 0.2009771 sec
RG 2050
DW 3.067 usec
DE 6.50 usec
TE 297.9 K
D1 2.00000000 sec
TD0 1

======== CHANNEL f1 ========
SFO1 161.9674942 MHz
NUC1 31P
P1 10.00 usec
PLW1 25.00000000 W

F2 − Processing parameters
SI 32768
SF 161.9755930 MHz
WDW EM
SSB 0
LB 1.00 Hz
GB 0
PC 1.40

^{1}H NMR ($CDCl_3$): **8f**

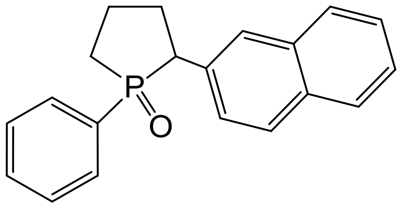

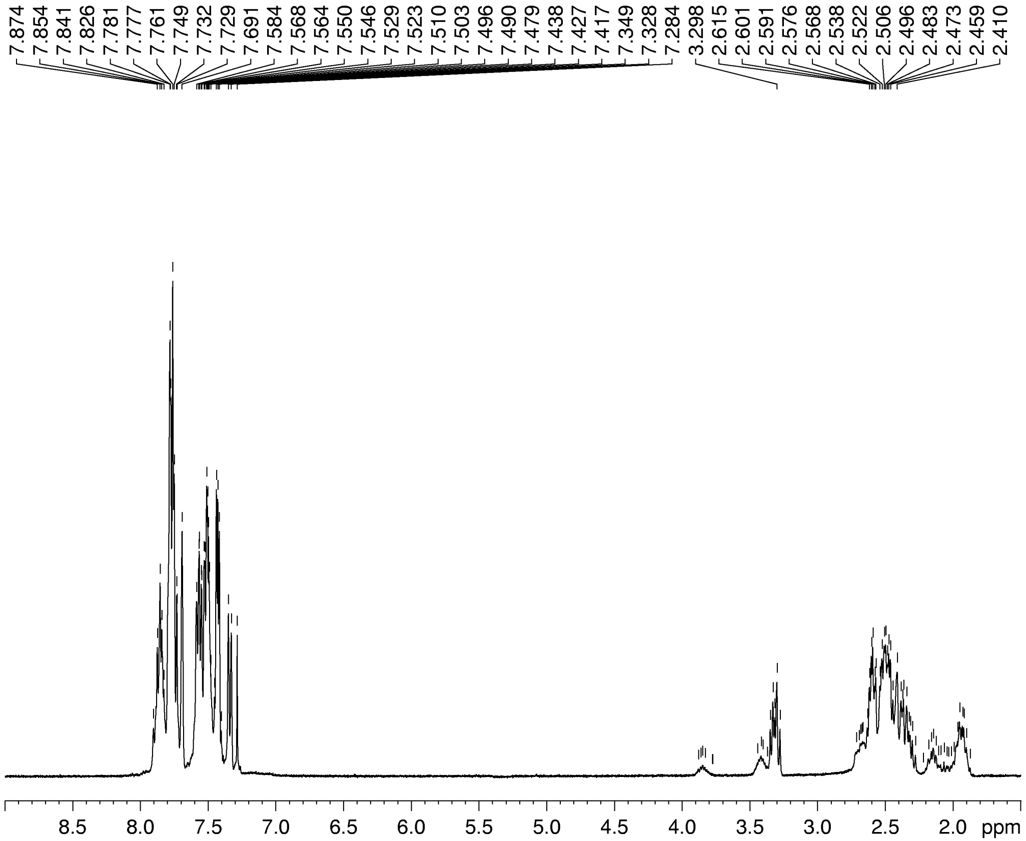

Current Data Parameters
NAME KRA-333-3-30
EXPNO 1
PROCNO 1

F2 - Acquisition Parameters
Date_ 20150420
Time 10.30
INSTRUM spect
PROBHD 5 mm PABBO BE
PULPROG zg30
TD 16384
SOLVENT CDCl3
NS 1
DS 0
SWH 4901.961 Hz
FIDRES 0.299192 Hz
AQ 1.6711680 sec
RG 101
DW 102.000 usec
DE 6.50 usec
TE 298.8 K
D1 1.00000000 sec
TD0 1

======== CHANNEL f1 ===
SFO1 400.1322177 MH:
NUC1 1H
P1 14.80 usec
PLW1 8.89999962 W

F2 - Processing parameters
SI 16384
SF 400.1300000 MHz
WDW no
SSB 0
LB 0 Hz
GB 0
PC 1.00

^{13}C NMR ($CDCl_3$): **8f**

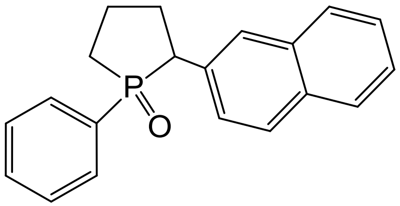

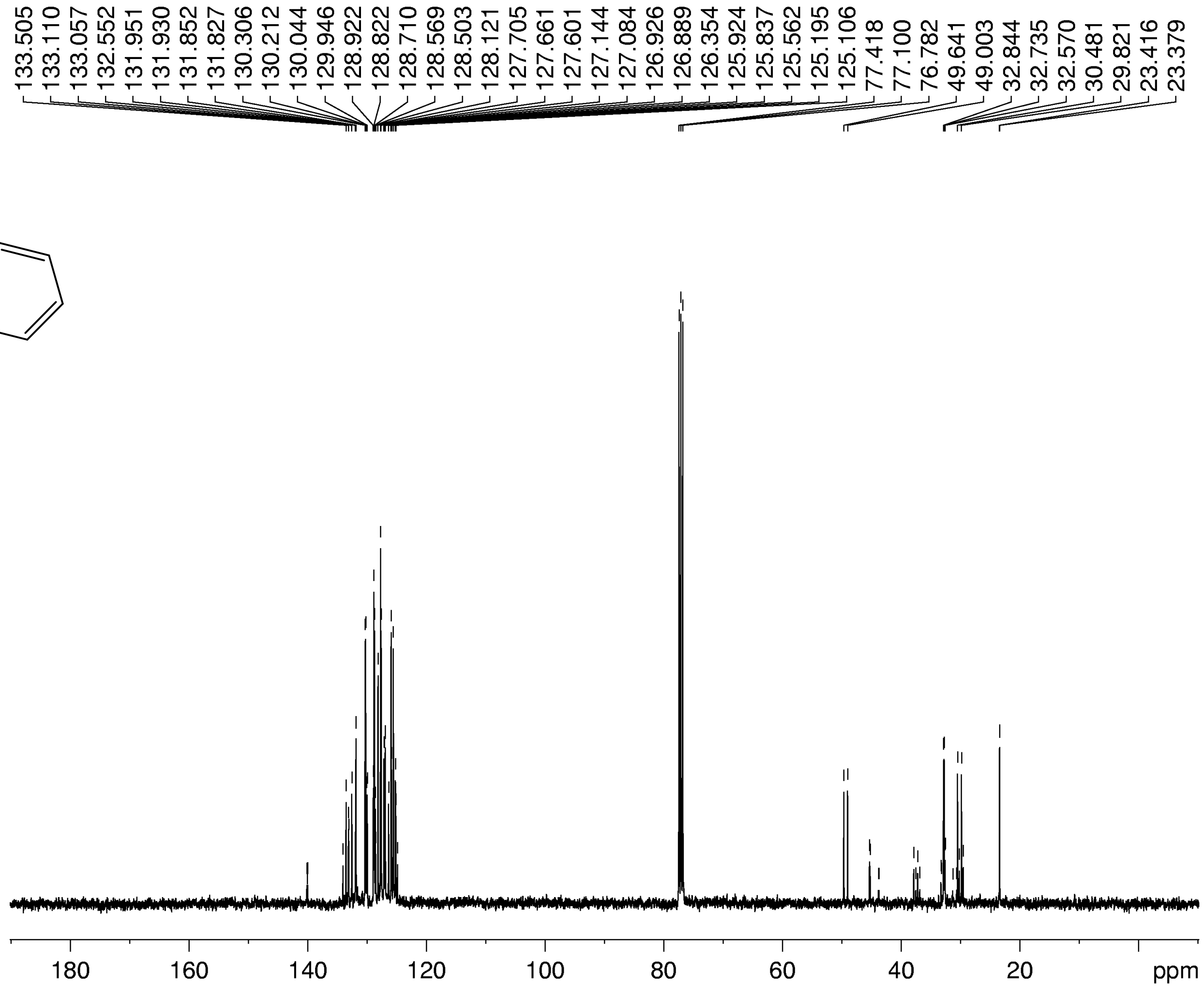

Current Data Parameters
NAME KRA-333-3-30
EXPNO 13
PROCNO 1

F2 - Acquisition Parameters
Date_ 20150420
Time 10.32
INSTRUM spect
PROBHD 5 mm PABBO BB
PULPROG zgpg30
TD 16384
SOLVENT CDCl3
NS 658
DS 2
SWH 20161.291 Hz
FIDRES 1.230548 Hz
AQ 0.4063232 sec
RG 2050
DW 24.800 usec
DE 6.50 usec
TE 298.9 K
D1 1.00000000 sec
D11 0.03000000 sec
TD0 8

======== CHANNEL f1 ===
SFO1 100.6218241 MHz
NUC1 13C
P1 10.00 usec
PLW1 34.00000000 W

======== CHANNEL f2 ===
SFO2 400.1322007 MHz
NUC2 1H
CPDPRG[2 waltz16
PCPD2 90.00 usec
PLW2 8.89999962 W
PLW12 0.24067000 W
PLW13 0.19495000 W

F2 - Processing parameters
SI 65536
SF 100.6127692 MHz
WDW EM
SSB 0
LB 2.00 Hz
GB 0
[illegible]

^{31}P NMR ($CDCl_3$): **8f**

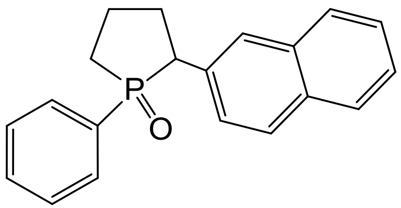

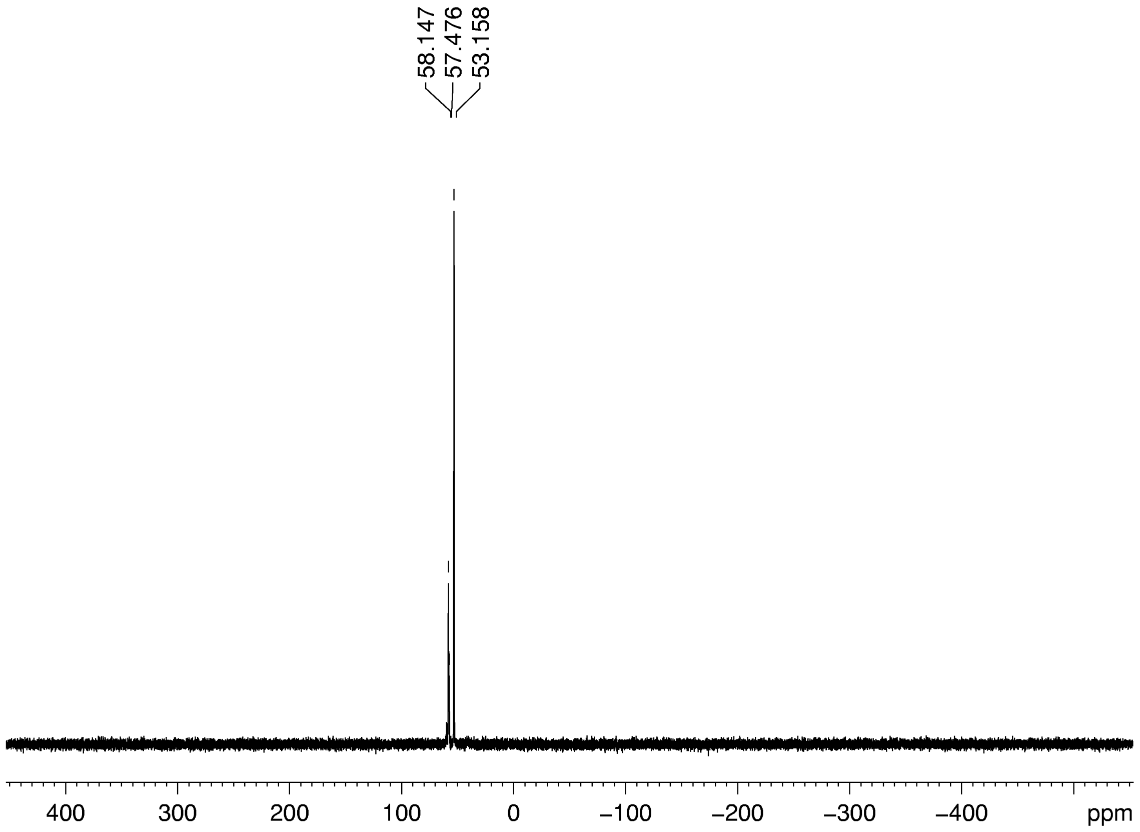

Current Data Parameters
NAME KRA−333−3−30
EXPNO 31
PROCNO 1

F2 − Acquisition Parameters
Date_ 20150420
Time 11.04
INSTRUM spect
PROBHD 5 mm PABBO BB−
PULPROG zg30
TD 65536
SOLVENT CDCl3
NS 64
DS 4
SWH 163043.484 Hz
FIDRES 2.487846 Hz
AQ 0.2009771 sec
RG 2050
DW 3.067 usec
DE 6.50 usec
TE 298.9 K
D1 2.00000000 sec
TD0 1

======== CHANNEL f1 ====
SFO1 161.9674942 MHz
NUC1 31P
P1 10.00 usec
PLW1 25.00000000 W

F2 − Processing parameters
SI 32768
SF 161.9755930 MHz
WDW EM
SSB 0
LB 1.00 Hz
GB 0
PC 1.40

^{1}H NMR ($CDCl_3$): **4a**

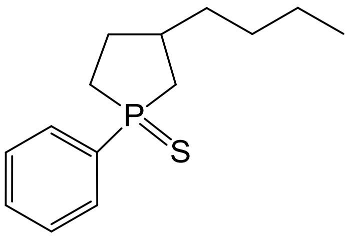

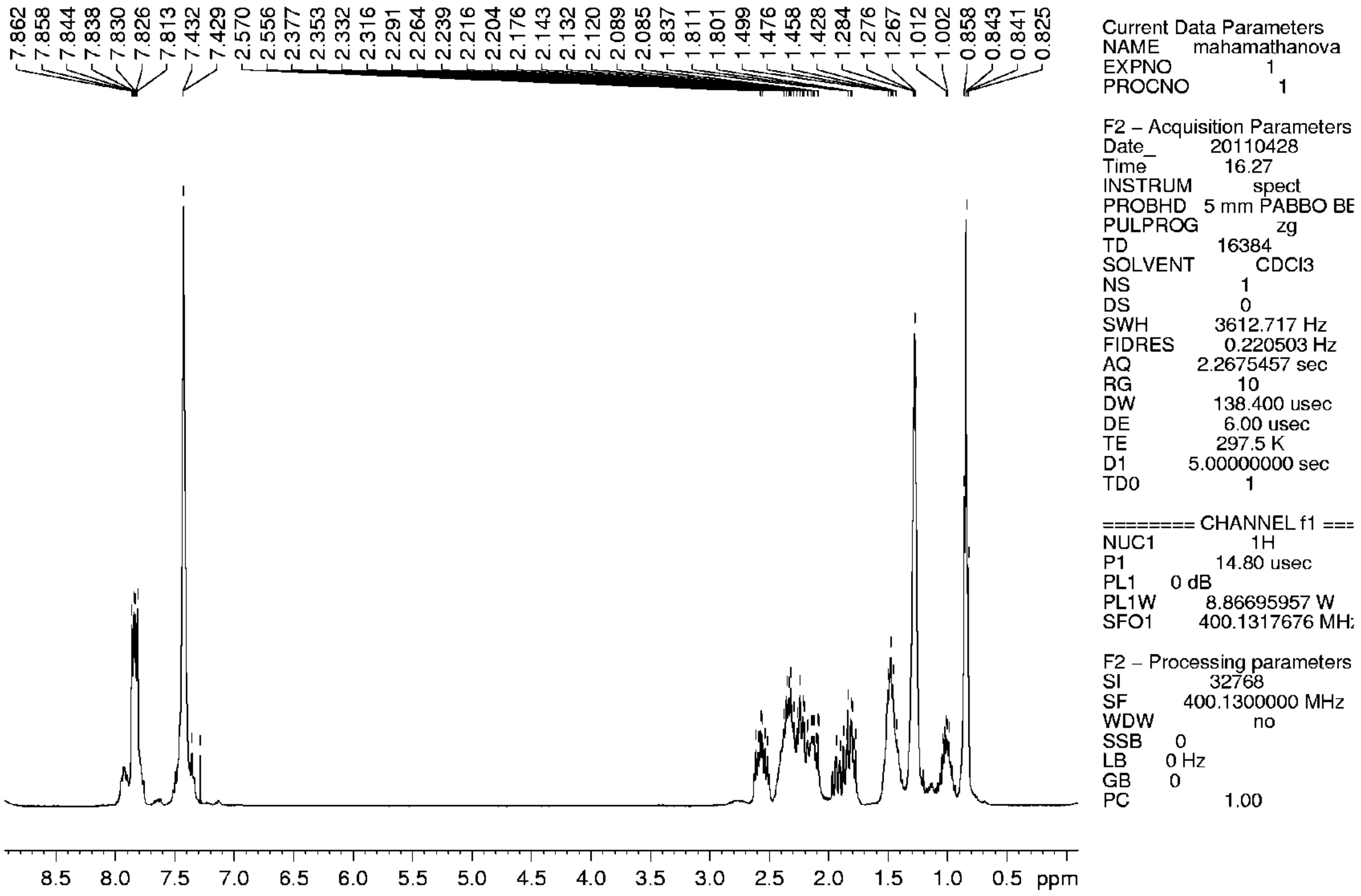

^{13}C NMR ($CDCl_3$): **4a**

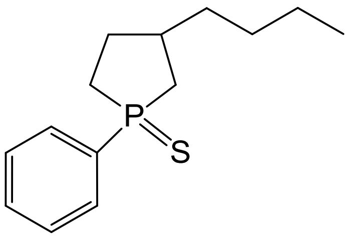

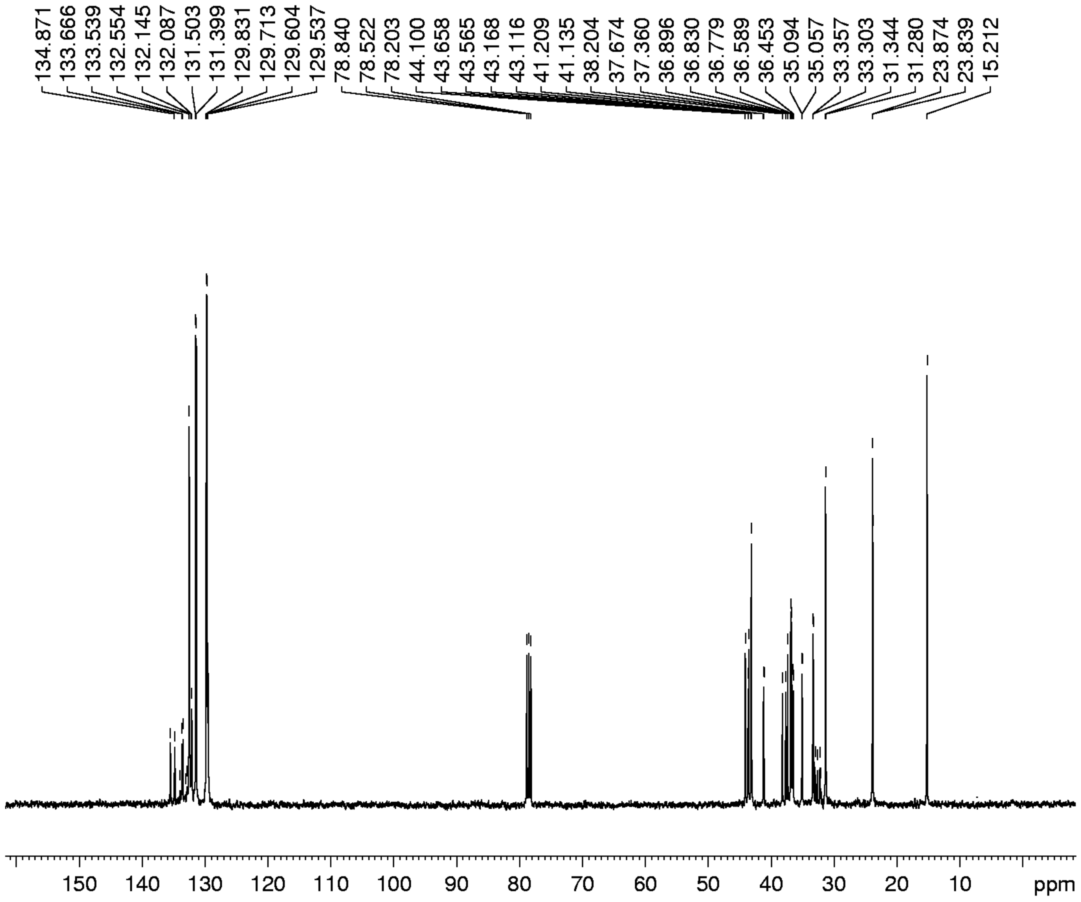

Current Data Parameters
NAME mahamathanova
EXPNO 2
PROCNO 1

F2 - Acquisition Parameters
Date_ 20110428
Time 16.28
INSTRUM spect
PROBHD 5 mm PABBO BB-
PULPROG zgpg30
TD 32144
SOLVENT CDCl3
NS 193
DS 0
SWH 26315.789 Hz
FIDRES 0.818684 Hz
AQ 0.6107360 sec
RG 2050
DW 19.000 usec
DE 30.00 usec
TE 297.6 K
D1 1.00000000 sec
D11 0.03000000 sec
TD0 1

======== CHANNEL f1 ====
NUC1 13C
P1 10.00 usec
PL1 0 dB
PL1W 33.91046524 W
SFO1 100.6233499 MHz

======== CHANNEL f2 ====
CPDPRG[2 waltz16
NUC2 1H
PCPD2 90.00 usec
PL2 0 dB
PL12 15.68 dB
PL13 18.70 dB
PL2W 8.86695957 W
PL12W 0.23975886 W
PL13W 0.11961196 W
SFO2 400.1317606 MHz

F2 - Processing parameters
SI 32768
SF 100.6126514 MHz
WDW EM
SSB 0

^{31}P NMR ($CDCl_3$): **4a**

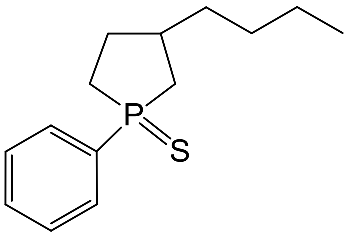

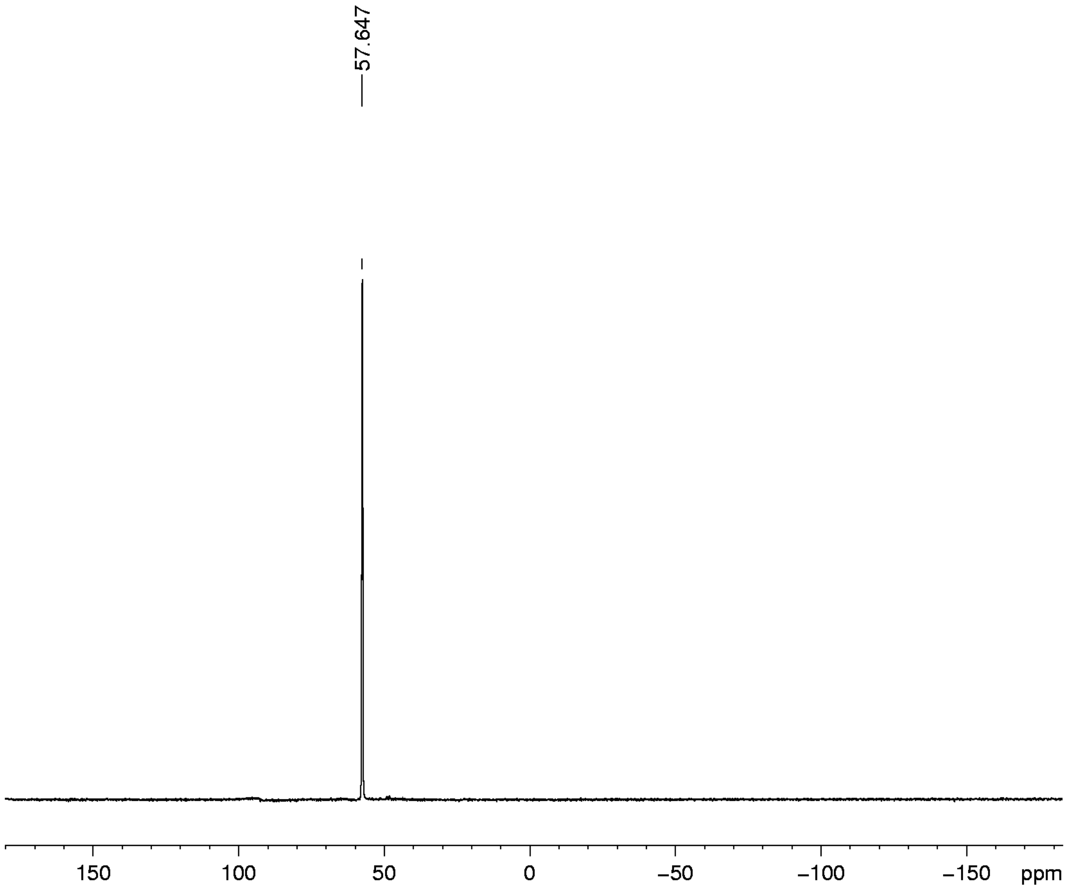

Current Data Parameters
NAME mahamathanova
EXPNO 7
PROCNO 1

F2 – Acquisition Parameters
Date_ 20110428
Time 17.09
INSTRUM spect
PROBHD 5 mm PABBO BE
PULPROG zg
TD 32768
SOLVENT CDCl3
NS 32
DS 0
SWH 64102.563 Hz
FIDRES 1.956255 Hz
AQ 0.2555904 sec
RG 2050
DW 7.800 usec
DE 6.00 usec
TE 297.6 K
D1 2.00000000 sec
TD0 1

======== CHANNEL f1 ===
NUC1 31P
P1 9.10 usec
PL1 0 dB
PL1W 24.94303322 W
SFO1 161.9727429 MH:

F2 – Processing parameters
SI 16384
SF 161.9755930 MHz
WDW EM
SSB 0
LB 5.00 Hz
GB 0
PC 1.40

^{1}H NMR ($CDCl_3$): **4b**

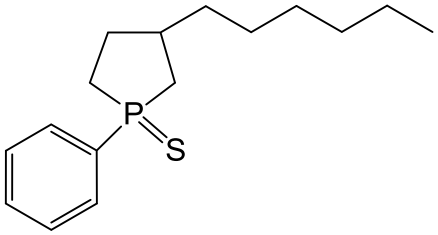

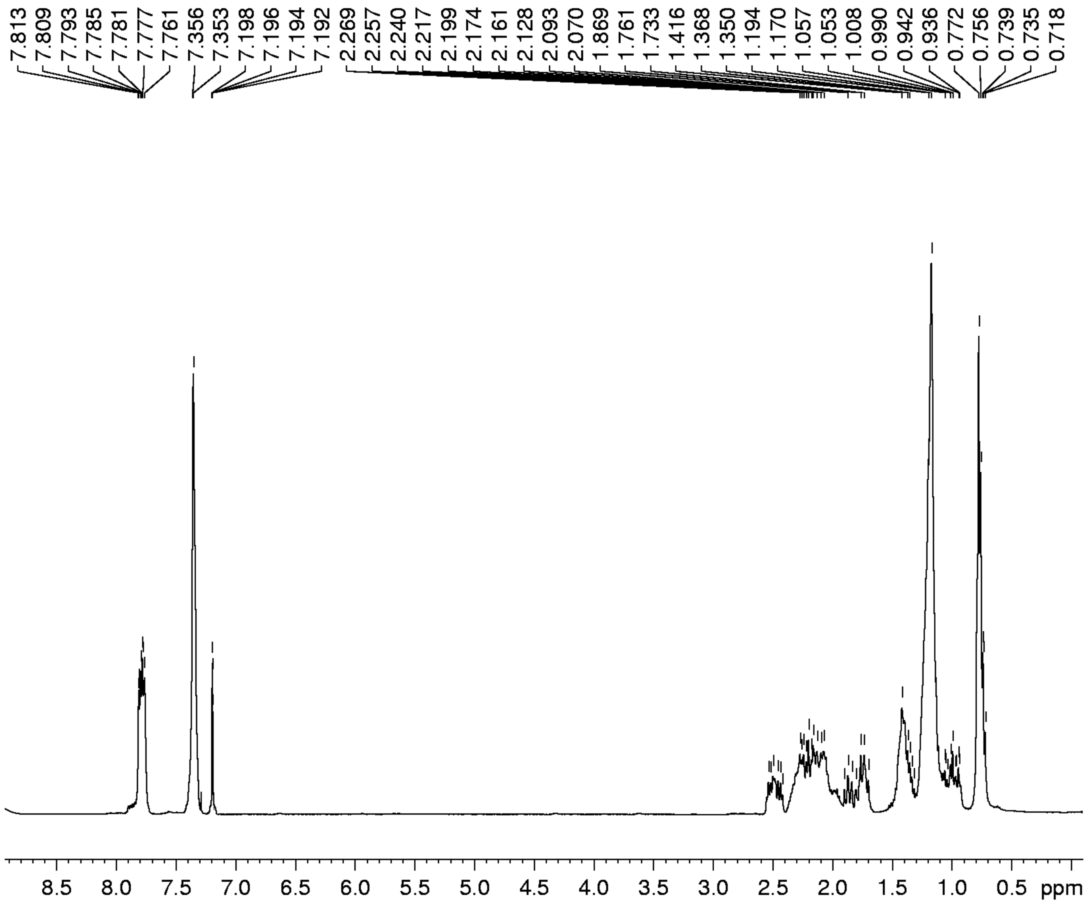

Current Data Parameters
NAME mahamathanova
EXPNO 12
PROCNO 1

F2 – Acquisition Parameters
Date_ 20110428
Time 18.56
INSTRUM spect
PROBHD 5 mm PABBO BB-
PULPROG zg
TD 16384
SOLVENT CDCl3
NS 1
DS 0
SWH 3612.717 Hz
FIDRES 0.220503 Hz
AQ 2.2675457 sec
RG 5.6
DW 138.400 usec
DE 6.00 usec
TE 298.5 K
D1 5.00000000 sec
TD0 1

======== CHANNEL f1 ====
NUC1 1H
P1 14.80 usec
PL1 0 dB
PL1W 8.86695957 W
SFO1 400.1317676 MHz

F2 – Processing parameters
SI 32768
SF 400.1300000 MHz
WDW no
SSB 0
LB 0 Hz
GB 0
PC 1.00

^{13}C NMR ($CDCl_3$): **4b**

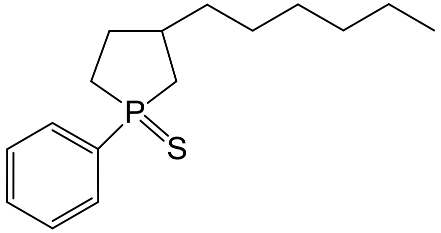

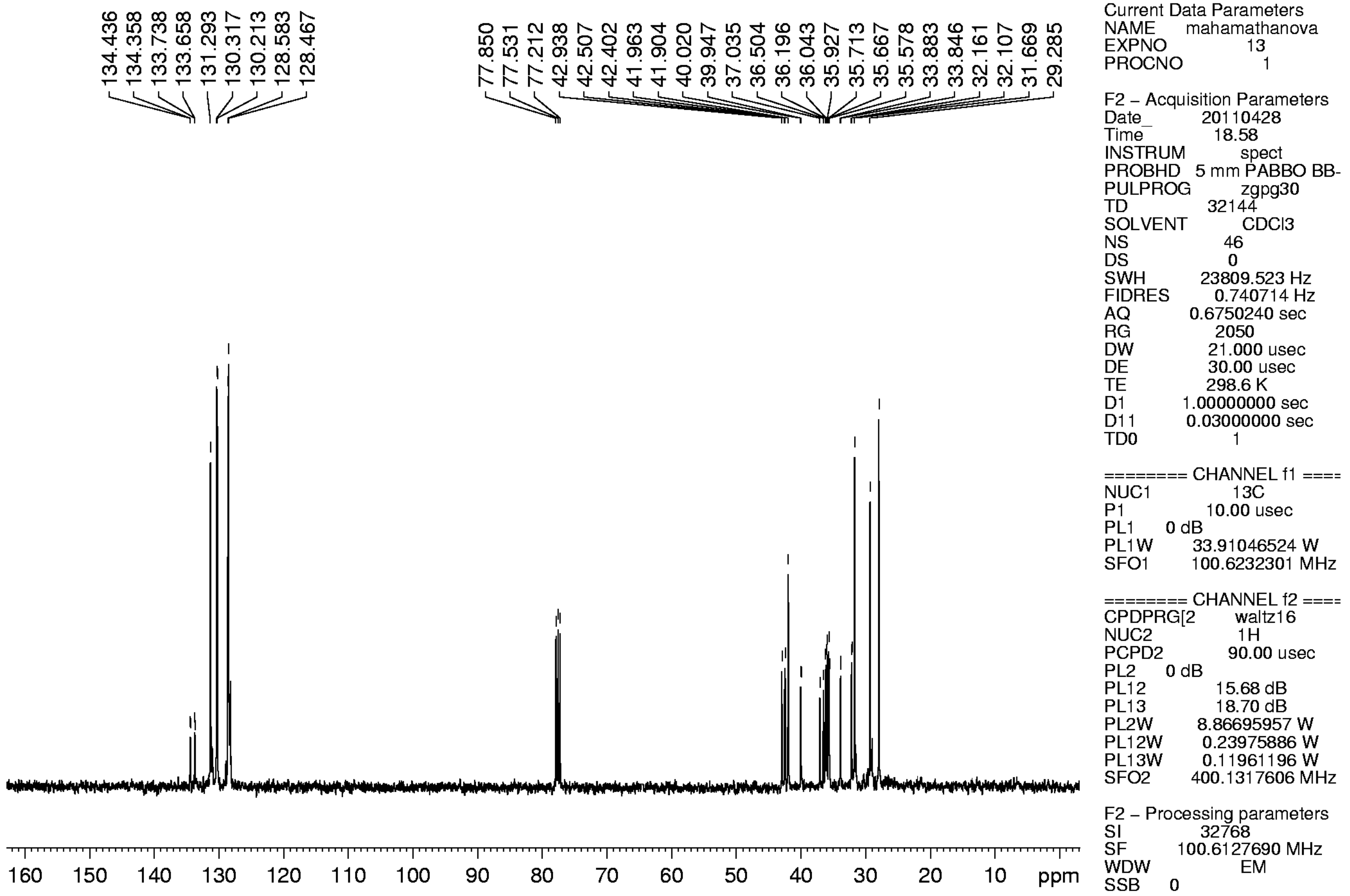

^{31}P NMR ($CDCl_3$): **4b**

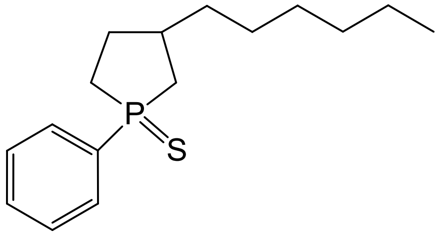

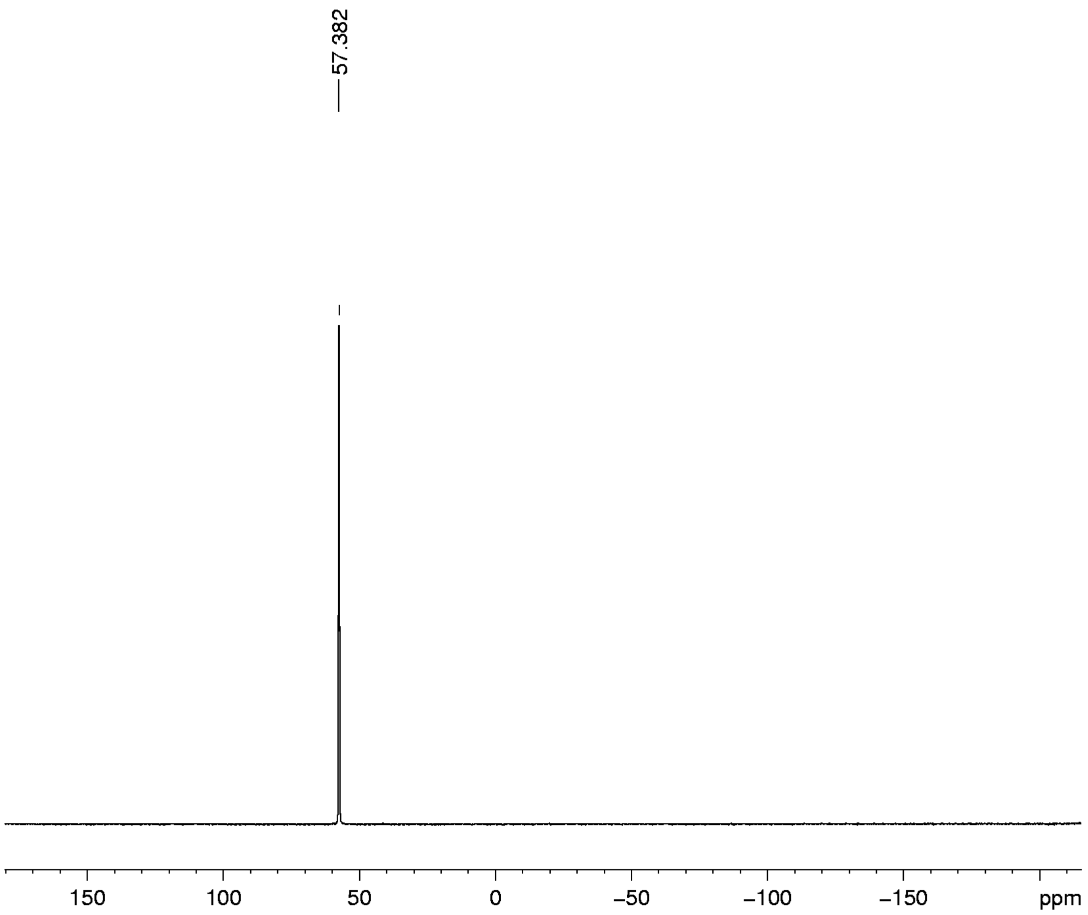

Current Data Parameters
NAME mahamathanova
EXPNO 18
PROCNO 1

F2 – Acquisition Parameters
Date_ 20110428
Time 19.21
INSTRUM spect
PROBHD 5 mm PABBO BE
PULPROG zg
TD 32768
SOLVENT CDCl3
NS 12
DS 0
SWH 64102.563 Hz
FIDRES 1.956255 Hz
AQ 0.2555904 sec
RG 1820
DW 7.800 usec
DE 6.00 usec
TE 297.5 K
D1 2.00000000 sec
TD0 1

======== CHANNEL f1 ===
NUC1 31P
P1 9.10 usec
PL1 0 dB
PL1W 24.94303322 W
SFO1 161.9727429 MH

F2 – Processing parameters
SI 16384
SF 161.9755930 MHz
WDW EM
SSB 0
LB 5.00 Hz
GB 0
PC 1.40

^{1}H NMR ($CDCl_3$): **4c**

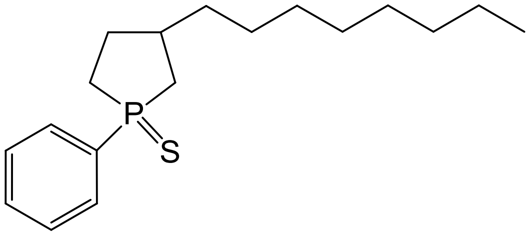

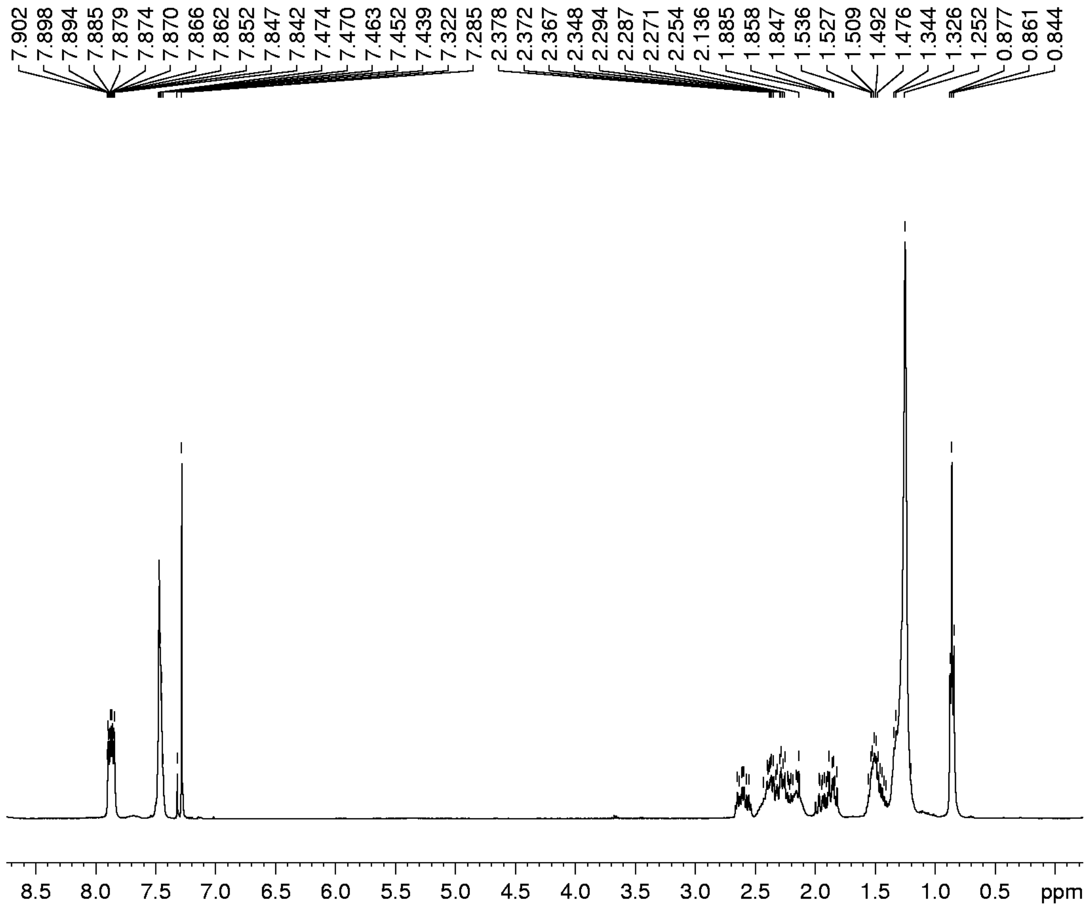

Current Data Parameters
NAME MRC−17
EXPNO 1
PROCNO 1

F2 − Acquisition Parameters
Date_ 20121206
Time 14.27
INSTRUM spect
PROBHD 5 mm PABBO BB
PULPROG zg30
TD 16384
SOLVENT CDCl3
NS 1
DS 0
SWH 3591.954 Hz
FIDRES 0.219235 Hz
AQ 2.2806528 sec
RG 22.6
DW 139.200 usec
DE 6.00 usec
TE 297.9 K
D1 1.00000000 sec
TD0 1

======== CHANNEL f1 ===
NUC1 1H
P1 14.80 usec
PL1 0 dB
PL1W 8.86695957 W
SFO1 400.1317034 MHz

F2 − Processing parameters
SI 32768
SF 400.1300000 MHz
WDW EM
SSB 0
LB 0.10 Hz
GB 0
PC 1.00

^{13}C NMR ($CDCl_3$): **4c**

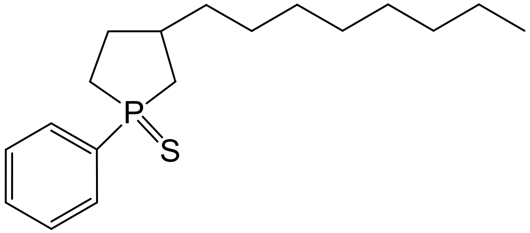

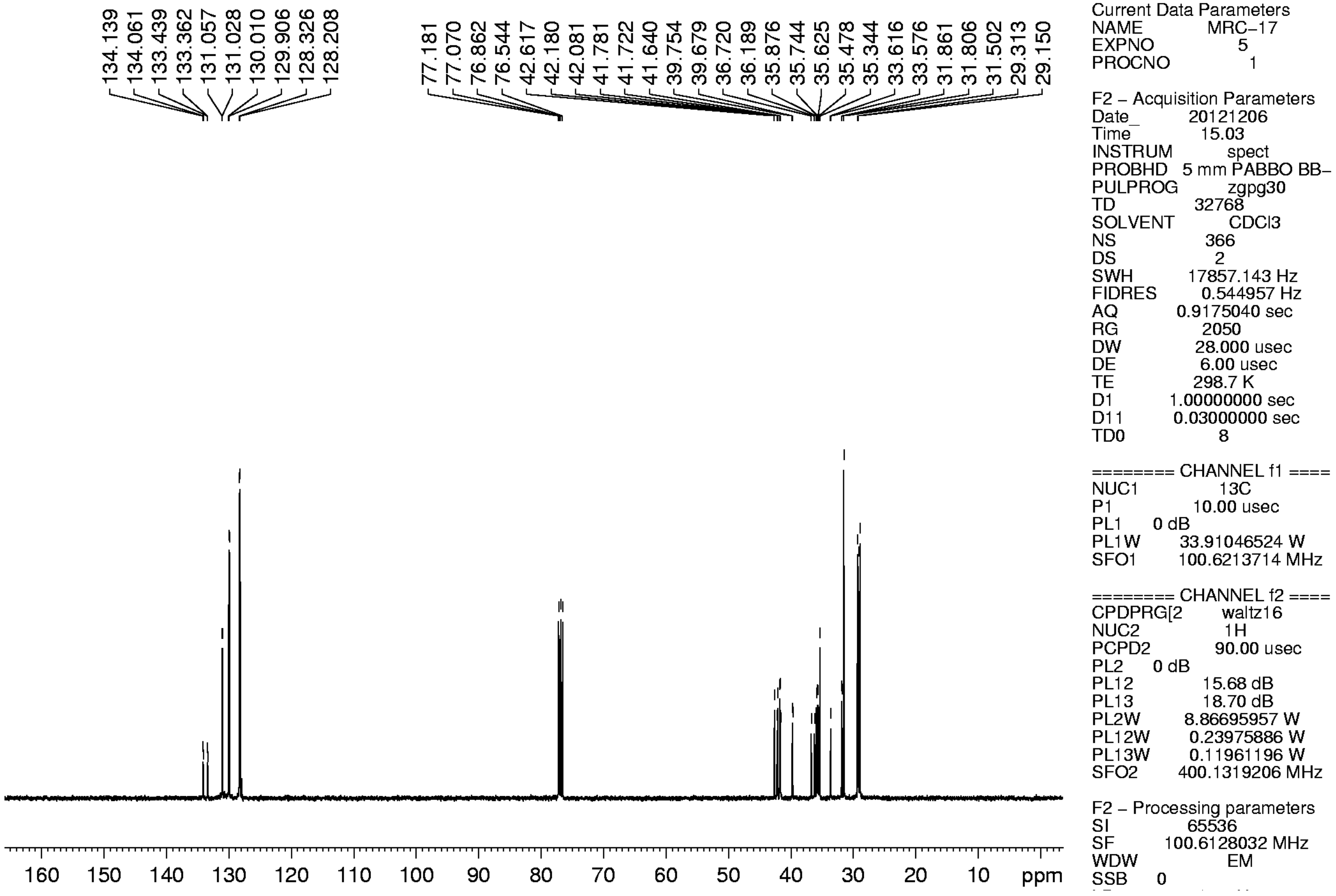

^{31}P NMR ($CDCl_3$): **4c**

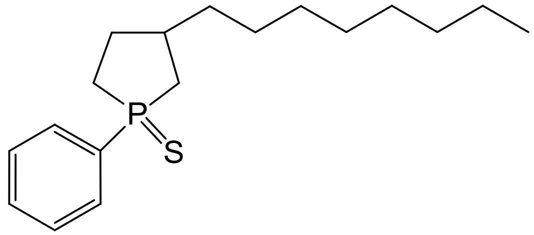

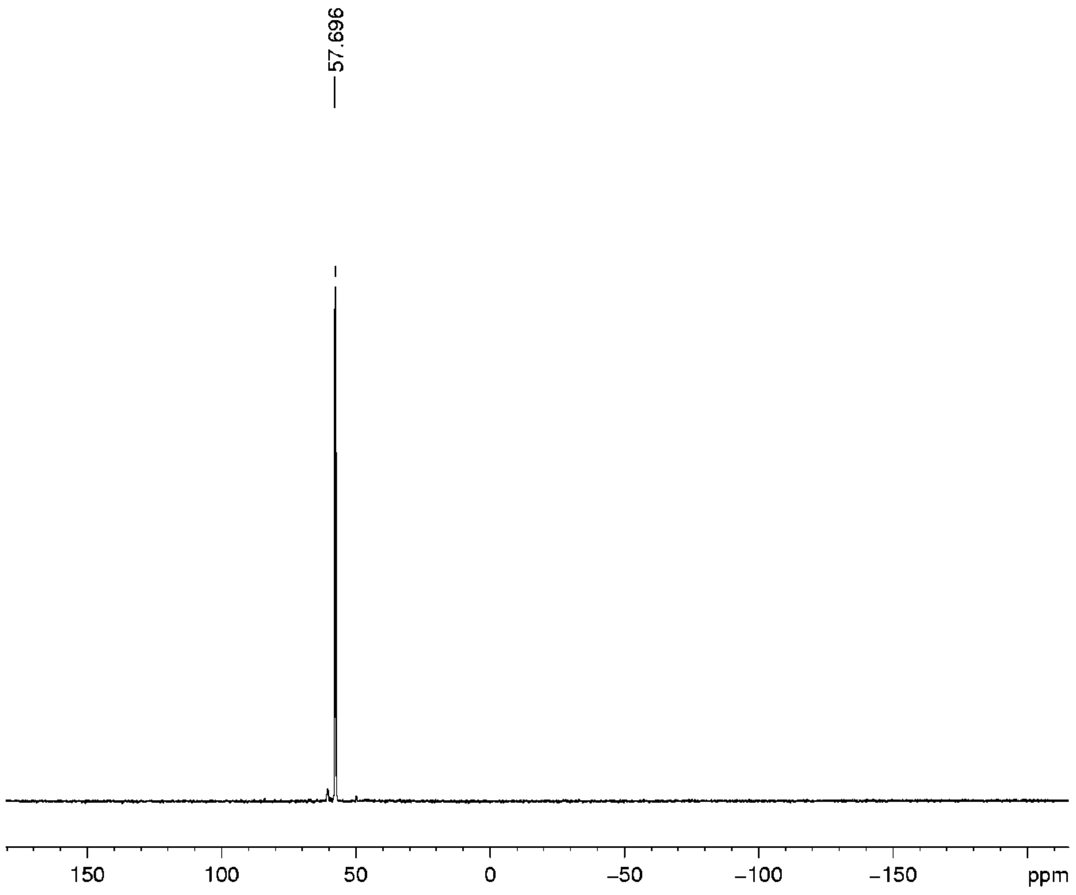

Current Data Parameters
NAME MRC−17
EXPNO 6
PROCNO 1

F2 − Acquisition Parameters
Date_ 20121207
Time 16.03
INSTRUM spect
PROBHD 5 mm PABBO B
PULPROG zg
TD 32768
SOLVENT CDCl3
NS 18
DS 0
SWH 64102.563 Hz
FIDRES 1.956255 Hz
AQ 0.2555904 sec
RG 1820
DW 7.800 usec
DE 6.00 usec
TE 297.8 K
D1 2.00000000 sec
TD0 1

======== CHANNEL f1 ==
NUC1 31P
P1 9.10 usec
PL1 0 dB
PL1W 24.94303322 W
SFO1 161.9727429 MH

F2 − Processing parameters
SI 16384
SF 161.9755930 MHz
WDW EM
SSB 0
LB 5.00 Hz
GB 0
PC 1.40

^{1}H NMR ($CDCl_3$): **4d**

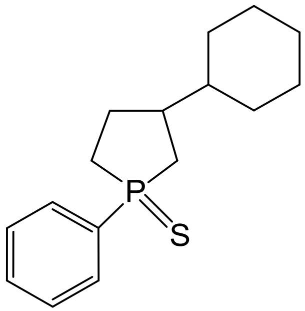

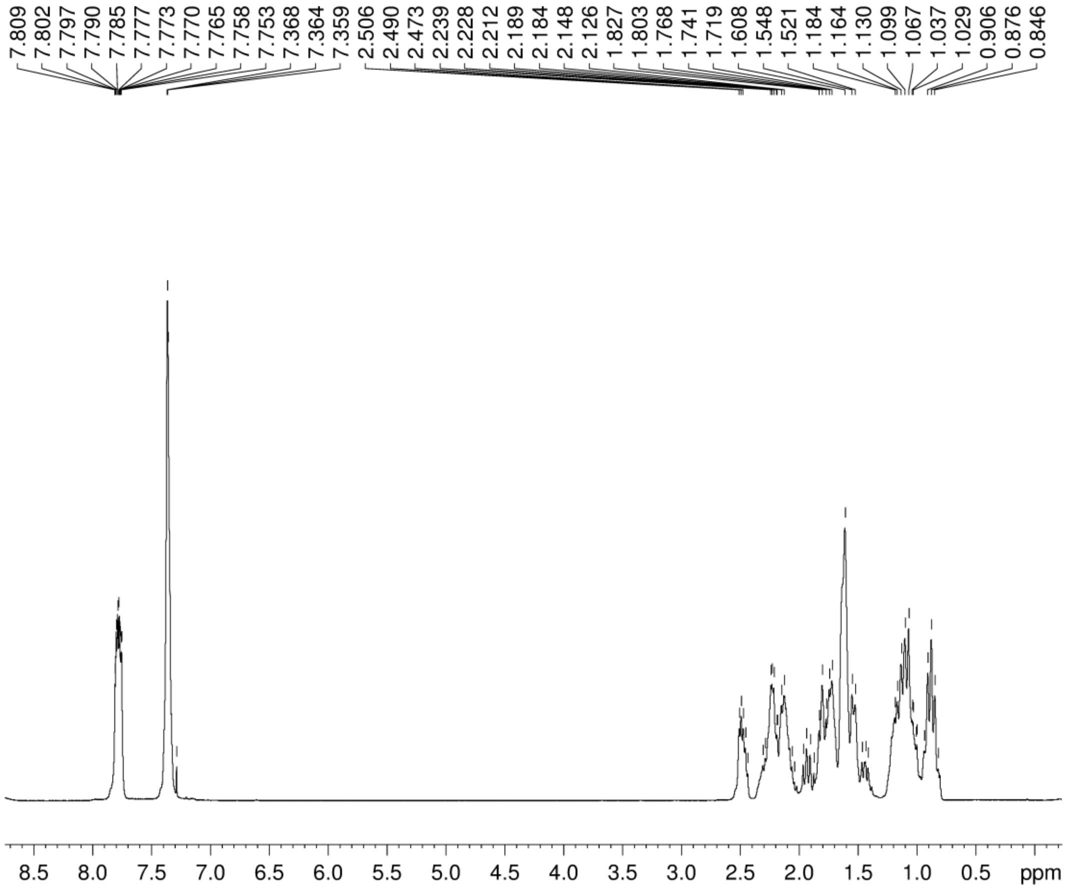

Current Data Parameters
NAME MRC−15
EXPNO 1
PROCNO 1

F2 − Acquisition Parameters
Date_ 20121207
Time 15.25
INSTRUM spect
PROBHD 5 mm PABBO BB
PULPROG zg30
TD 16384
SOLVENT CDCl3
NS 1
DS 0
SWH 3591.954 Hz
FIDRES 0.219235 Hz
AQ 2.2806528 sec
RG 12.7
DW 139.200 usec
DE 6.00 usec
TE 297.8 K
D1 1.00000000 sec
TD0 1

======== CHANNEL f1 ===
NUC1 1H
P1 14.80 usec
PL1 0 dB
PL1W 8.86695957 W
SFO1 400.1317034 MHz

F2 − Processing parameters
SI 32768
SF 400.1300000 MHz
WDW no
SSB 0
LB 0 Hz
GB 0
PC 1.00

^{13}C NMR ($CDCl_3$): **4d**

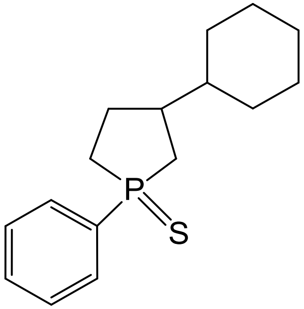

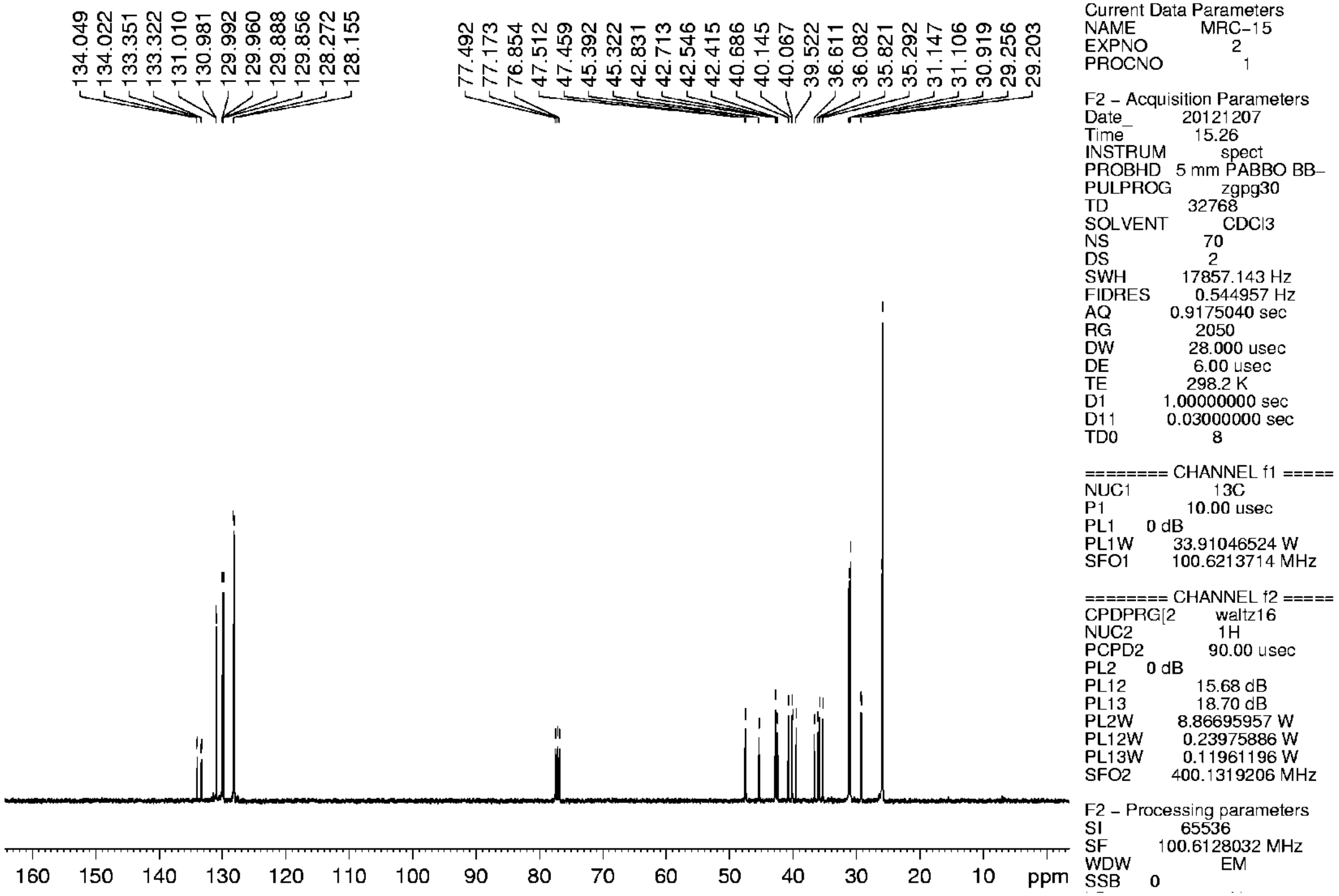

^{31}P NMR ($CDCl_3$): **4d**

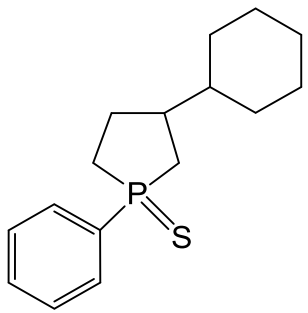

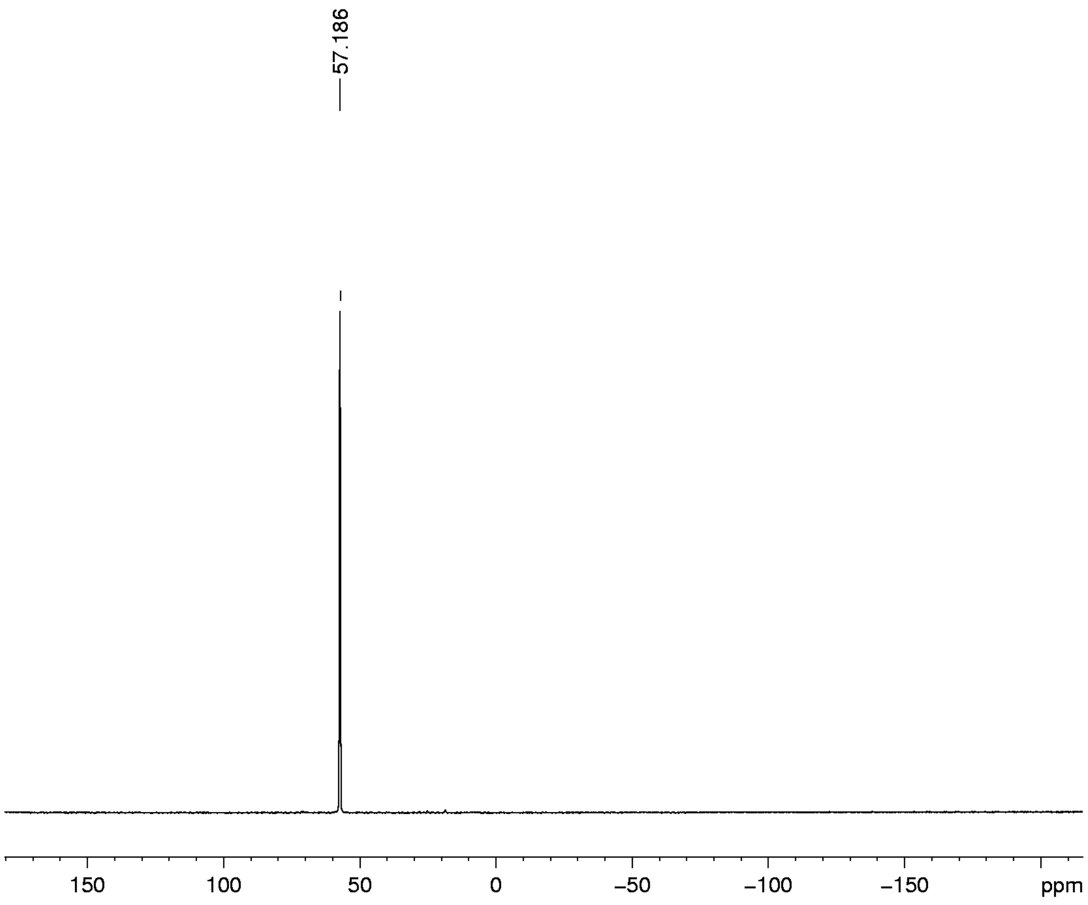

Current Data Parameters
NAME MRC−15
EXPNO 6
PROCNO 1

F2 − Acquisition Parameters
Date_ 20121207
Time 15.57
INSTRUM spect
PROBHD 5 mm PABBO BB
PULPROG zg
TD 32768
SOLVENT CDCl3
NS 21
DS 0
SWH 64102.563 Hz
FIDRES 1.956255 Hz
AQ 0.2555904 sec
RG 1820
DW 7.800 usec
DE 6.00 usec
TE 297.9 K
D1 2.00000000 sec
TD0 1

======== CHANNEL f1 ===
NUC1 31P
P1 9.10 usec
PL1 0 dB
PL1W 24.94303322 W
SFO1 161.9727429 MHz

F2 − Processing parameters
SI 16384
SF 161.9755930 MHz
WDW EM
SSB 0
LB 5.00 Hz
GB 0
PC 1.40

^{1}H NMR ($CDCl_3$): **4e**

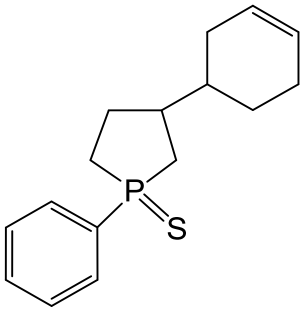

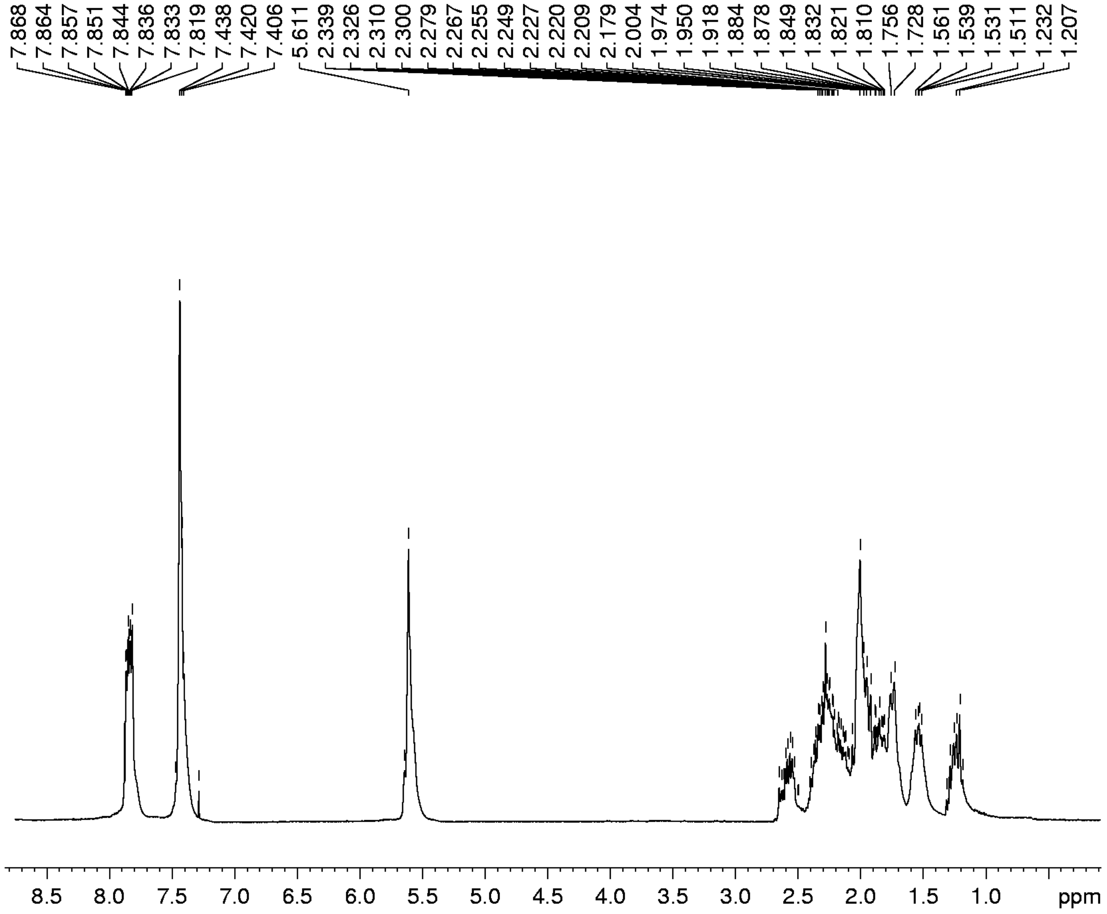

Current Data Parameters
NAME MRC–11
EXPNO 1
PROCNO 1

F2 – Acquisition Parameters
Date_ 20121129
Time 16.06
INSTRUM spect
PROBHD 5 mm PABBO B
PULPROG zg30
TD 16384
SOLVENT CDCl3
NS 1
DS 0
SWH 3591.954 Hz
FIDRES 0.219235 Hz
AQ 2.2806528 sec
RG 22.6
DW 139.200 usec
DE 6.00 usec
TE 297.5 K
D1 1.00000000 sec
TD0 1

======== CHANNEL f1 ==
NUC1 1H
P1 14.80 usec
PL1 0 dB
PL1W 8.86695957 W
SFO1 400.1317034 MH

F2 – Processing parameters
SI 32768
SF 400.1300000 MHz
WDW no
SSB 0
LB 0 Hz
GB 0
PC 1.00

^{13}C NMR ($CDCl_3$): **4e**

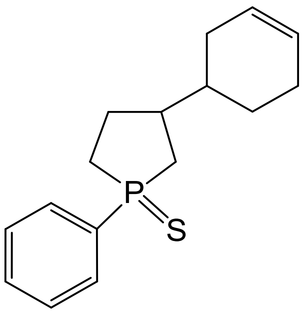

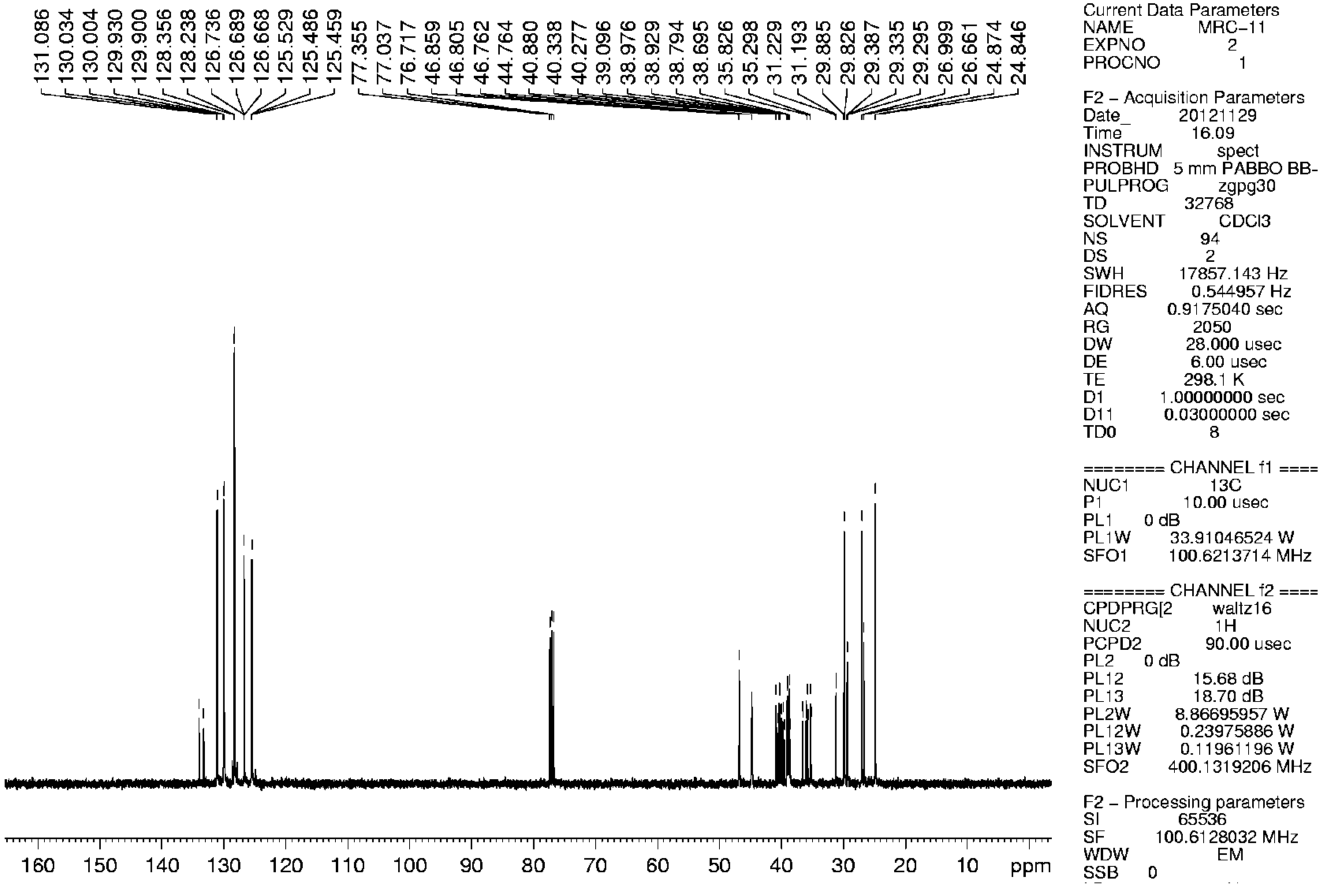

^{31}P NMR ($CDCl_3$): **4e**

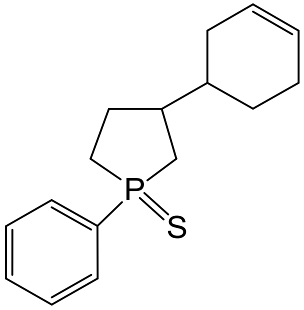

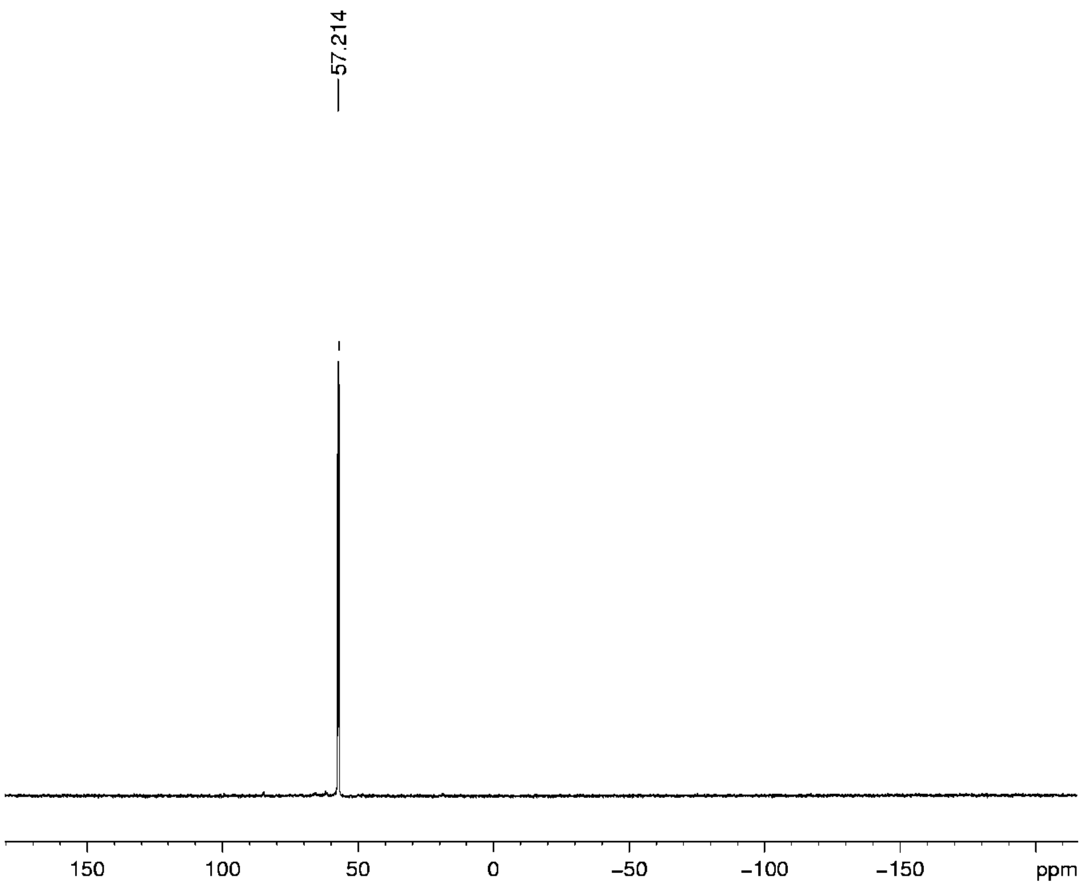

Current Data Parameters
NAME MRC−11
EXPNO 6
PROCNO 1

F2 − Acquisition Parameters
Date_ 20121129
Time 16.39
INSTRUM spect
PROBHD 5 mm PABBO BB-
PULPROG zg
TD 32768
SOLVENT CDCl3
NS 69
DS 0
SWH 64102.563 Hz
FIDRES 1.956255 Hz
AQ 0.2555904 sec
RG 2050
DW 7.800 usec
DE 6.00 usec
TE 297.6 K
D1 2.00000000 sec
TD0 1

======== CHANNEL f1 ========
NUC1 31P
P1 9.10 usec
PL1 0 dB
PL1W 24.94303322 W
SFO1 161.9727429 MHz

F2 − Processing parameters
SI 16384
SF 161.9755930 MHz
WDW EM
SSB 0
LB 5.00 Hz
GB 0
PC 1.40

^{1}H NMR ($CDCl_3$): **4f**

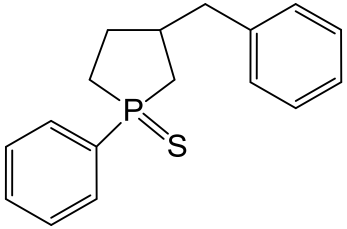

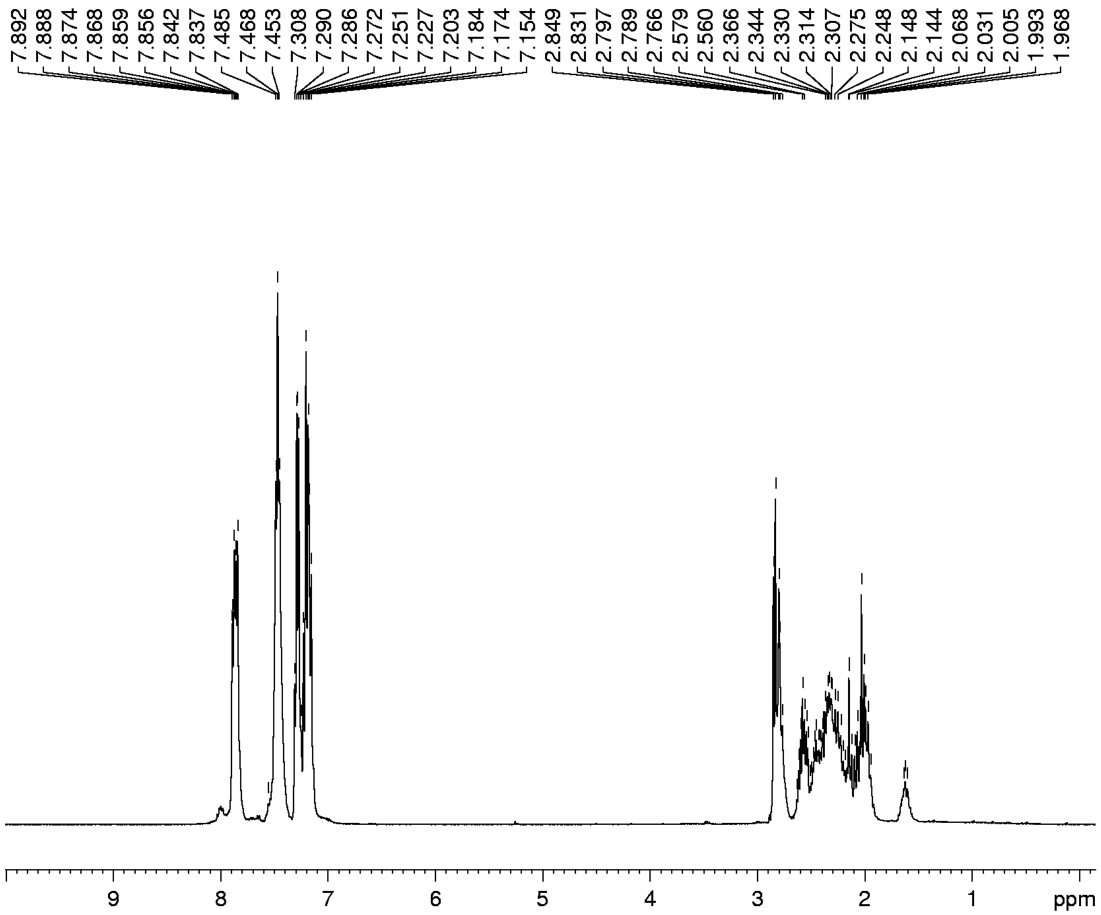

Current Data Parameters
NAME MAL–134–2
EXPNO 1
PROCNO 1

F2 – Acquisition Parameters
Date_ 20120305
Time 12.38
INSTRUM spect
PROBHD 5 mm PABBO BB
PULPROG zg30
TD 16384
SOLVENT CDCl3
NS 1
DS 0
SWH 6009.615 Hz
FIDRES 0.366798 Hz
AQ 1.3631488 sec
RG 28.5
DW 83.200 usec
DE 6.00 usec
TE 298.8 K
D1 1.00000000 sec
TD0 1

======== CHANNEL f1 ===
NUC1 1H
P1 14.80 usec
PL1 0 dB
PL1W 8.86695957 W
SFO1 400.1324008 MHz

F2 – Processing parameters
SI 32768
SF 400.1300000 MHz
WDW EM
SSB 0
LB 0.10 Hz
GB 0
PC 1.00

^{13}C NMR ($CDCl_3$): **4f**

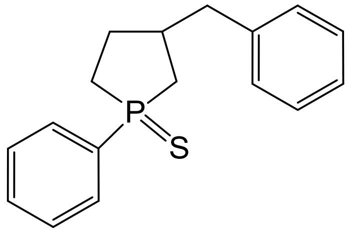

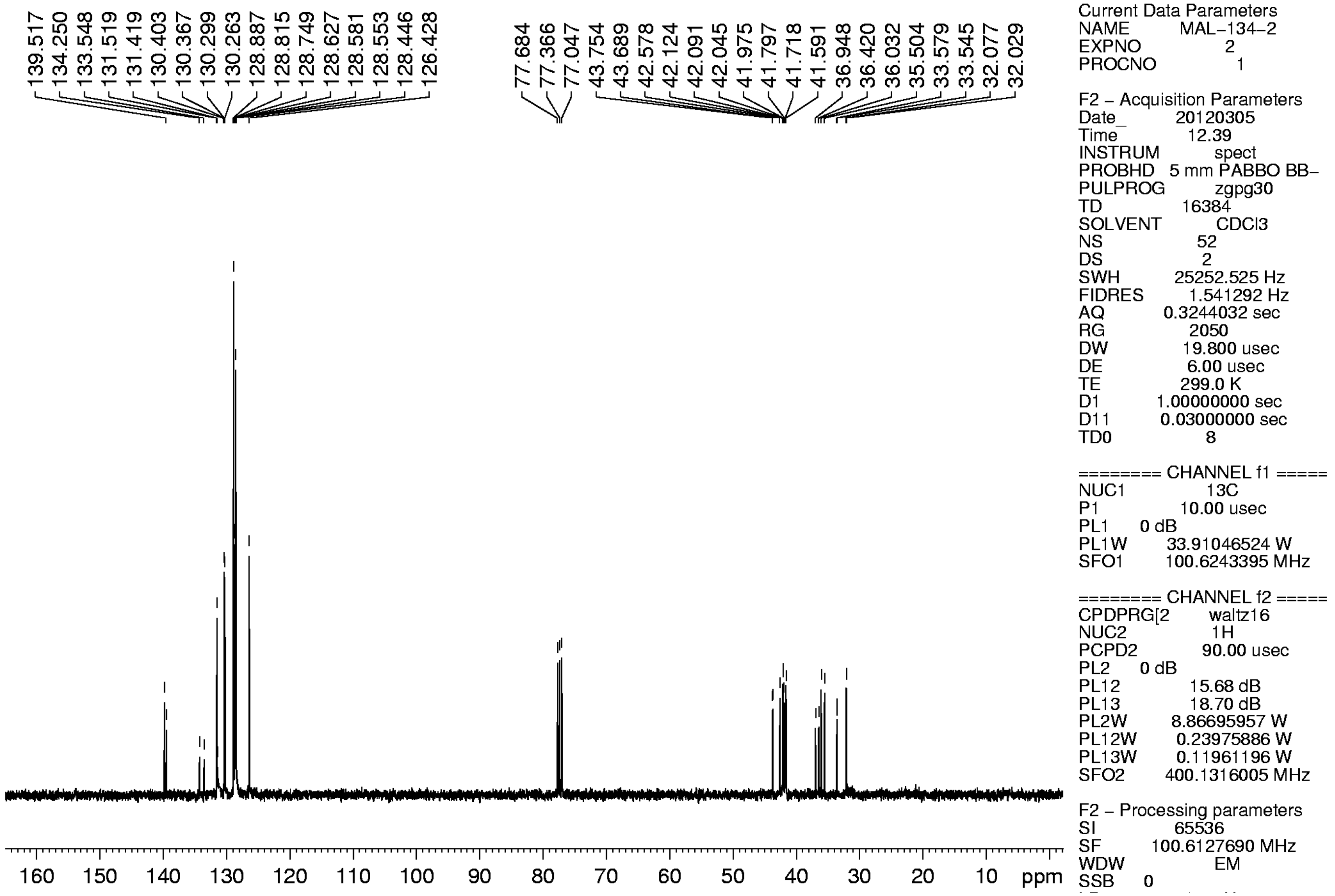

^{31}P NMR ($CDCl_3$): **4f**

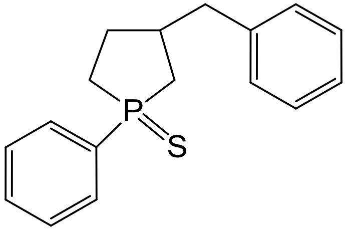

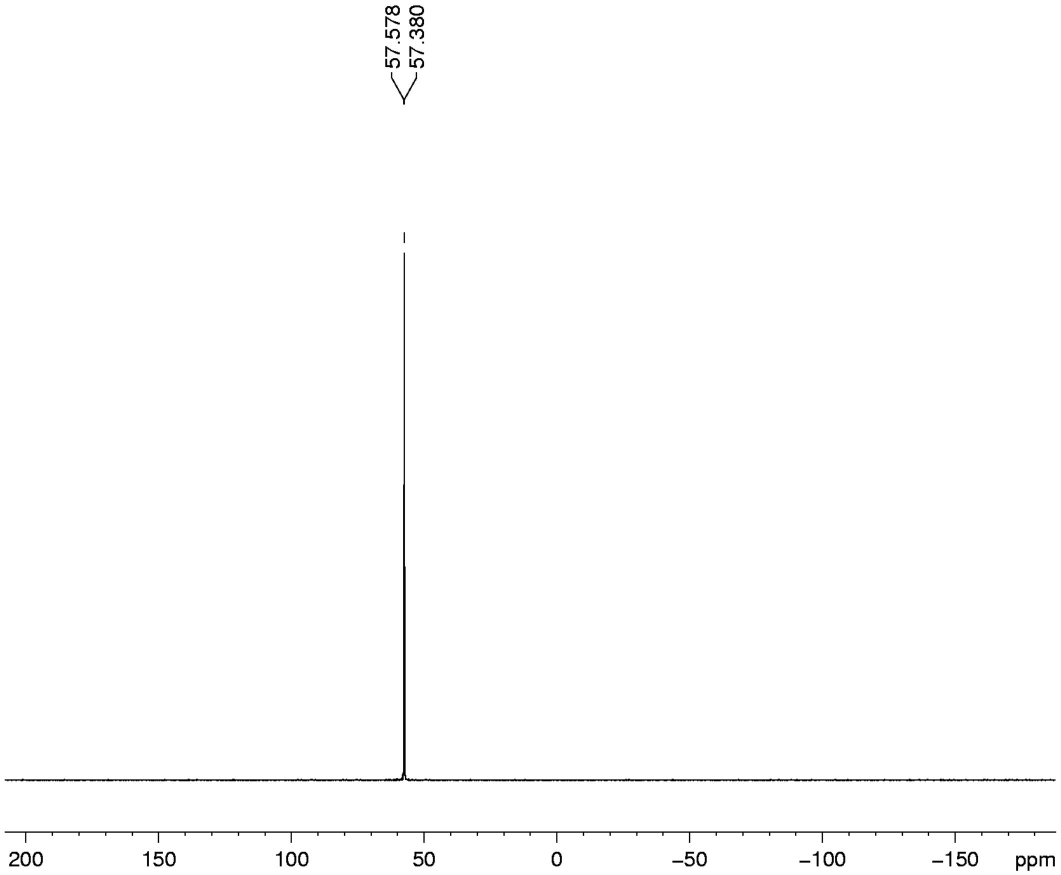

Current Data Parameters
NAME MAL−134−2
EXPNO 31
PROCNO 1

F2 − Acquisition Parameters
Date_ 20120305
Time 12.29
INSTRUM spect
PROBHD 5 mm PABBO BB
PULPROG zgpg
TD 16384
SOLVENT CDCl3
NS 1
DS 0
SWH 64102.563 Hz
FIDRES 3.912510 Hz
AQ 0.1277952 sec
RG 1290
DW 7.800 usec
DE 6.50 usec
TE 298.9 K
D1 10.00000000 sec
D11 0.03000000 sec
TD0 1

======== CHANNEL f1 ===
NUC1 31P
P1 9.10 usec
PL1 0 dB
PL1W 24.94303322 W
SFO1 161.9772128 MHz

======== CHANNEL f2 ===
CPDPRG[2 waltz16
NUC2 1H
PCPD2 90.00 usec
PL2 0 dB
PL12 15.68 dB
PL13 18.70 dB
PL2W 8.86695957 W

^{1}H NMR ($CDCl_3$): **4g**

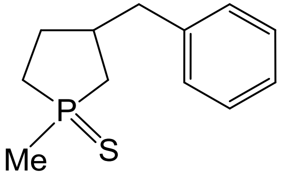

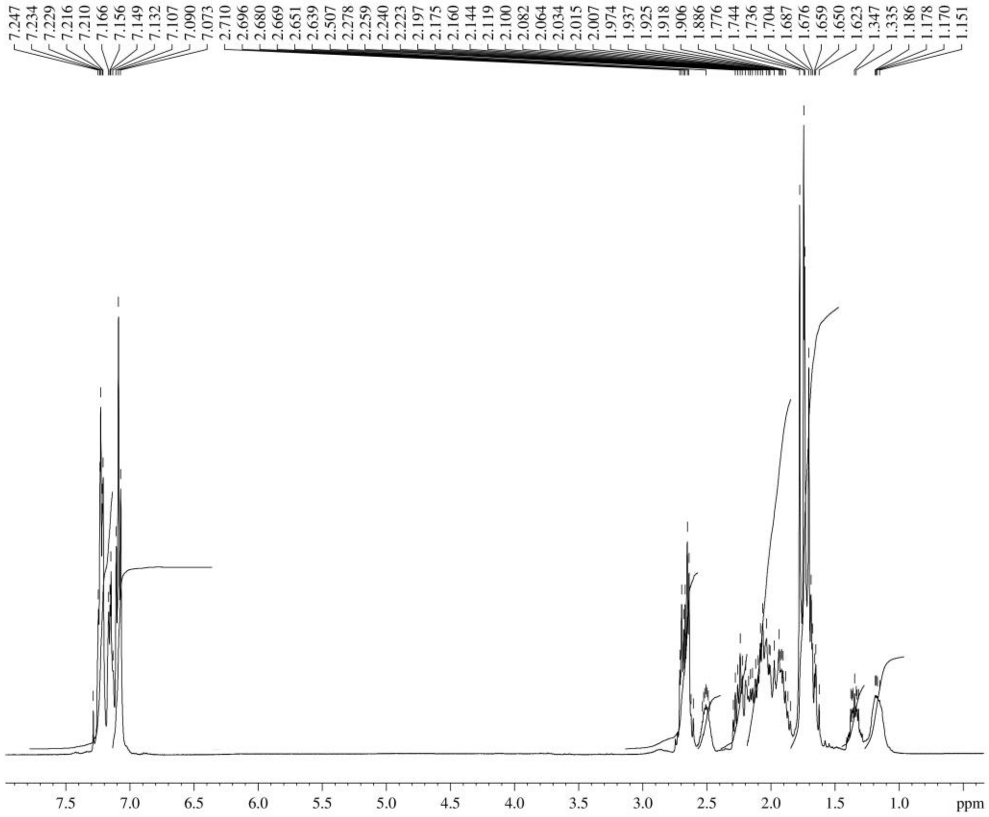

^{13}C NMR ($CDCl_3$): **4g**

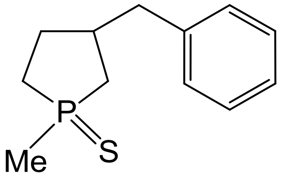

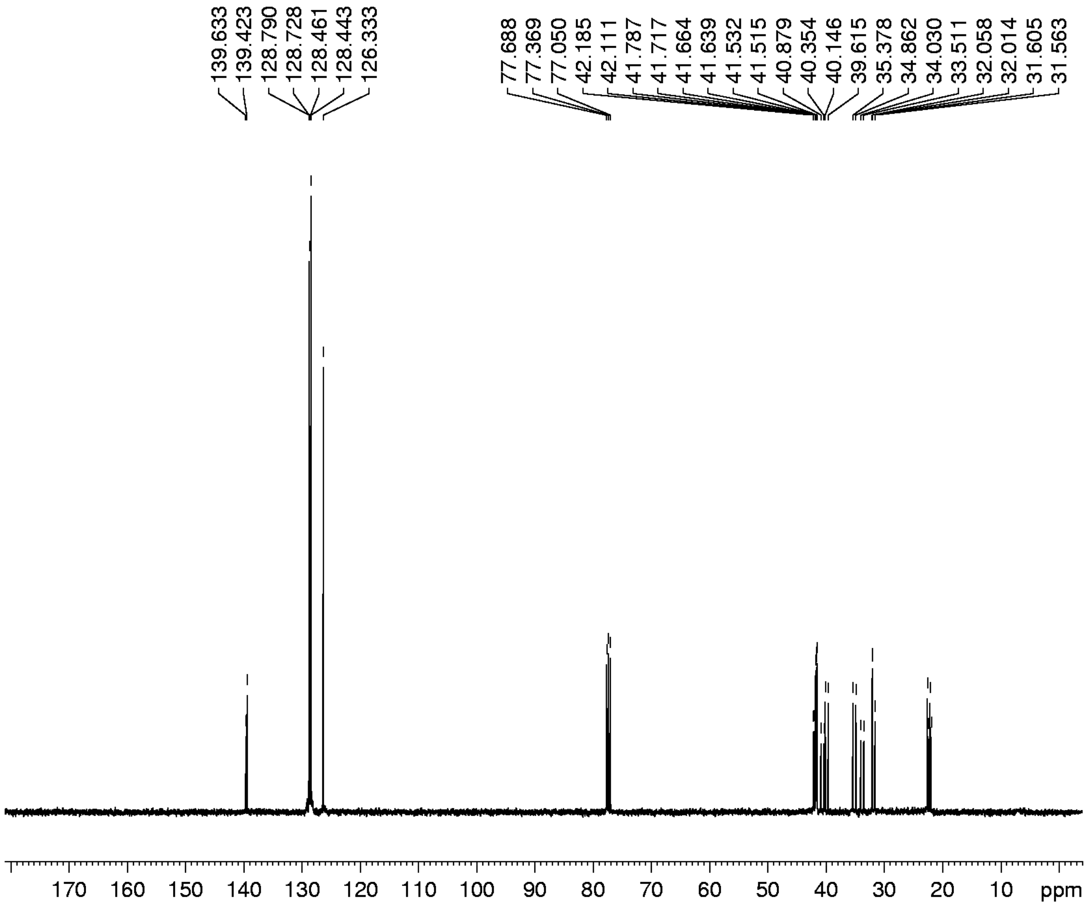

Current Data Parameters
NAME MAL–146S
EXPNO 2
PROCNO 1

F2 – Acquisition Parameters
Date_ 20120426
Time 16.14
INSTRUM spect
PROBHD 5 mm PABBO BB-
PULPROG zgpg30
TD 32768
SOLVENT CDCl3
NS 134
DS 2
SWH 23809.523 Hz
FIDRES 0.726609 Hz
AQ 0.6881280 sec
RG 2050
DW 21.000 usec
DE 6.00 usec
TE 298.9 K
D1 1.00000000 sec
D11 0.03000000 sec
TD0 8

======== CHANNEL f1 ====
NUC1 13C
P1 10.00 usec
PL1 0 dB
PL1W 33.91046524 W
SFO1 100.6242392 MHz

======== CHANNEL f2 ====
CPDPRG[2 waltz16
NUC2 1H
PCPD2 90.00 usec
PL2 0 dB
PL12 15.68 dB
PL13 18.70 dB
PL2W 8.86695957 W
PL12W 0.23975886 W
PL13W 0.11961196 W
SFO2 400.1316005 MHz

F2 – Processing parameters
SI 65536
SF 100.6127727 MHz
WDW EM
SSB 0

^{31}P NMR ($CDCl_3$): **4g**

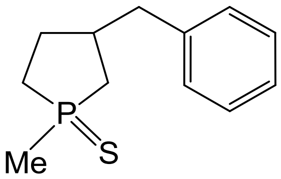

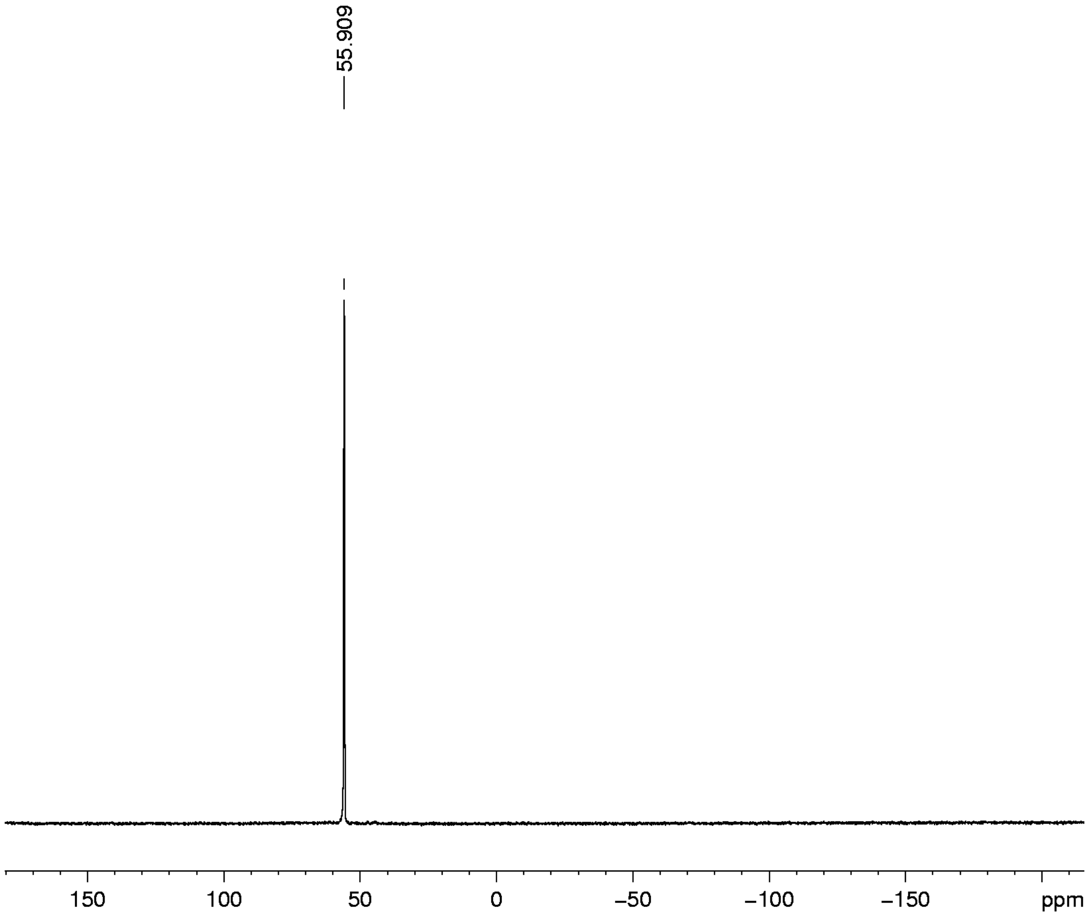

Current Data Parameters
NAME MAL−146S
EXPNO 7
PROCNO 1

F2 − Acquisition Parameters
Date_ 20120426
Time 16.43
INSTRUM spect
PROBHD 5 mm PABBO BB-
PULPROG zg
TD 32768
SOLVENT CDCl3
NS 4
DS 0
SWH 64102.563 Hz
FIDRES 1.956255 Hz
AQ 0.2555904 sec
RG 2050
DW 7.800 usec
DE 6.00 usec
TE 299.0 K
D1 2.00000000 sec
TD0 1

======== CHANNEL f1 ====
NUC1 31P
P1 9.10 usec
PL1 0 dB
PL1W 24.94303322 W
SFO1 161.9727429 MHz

F2 − Processing parameters
SI 16384
SF 161.9755930 MHz
WDW EM
SSB 0
LB 5.00 Hz
GB 0
PC 1.40

^{1}H NMR ($CDCl_3$): **4h**

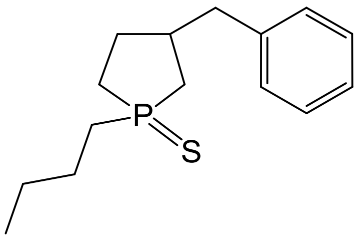

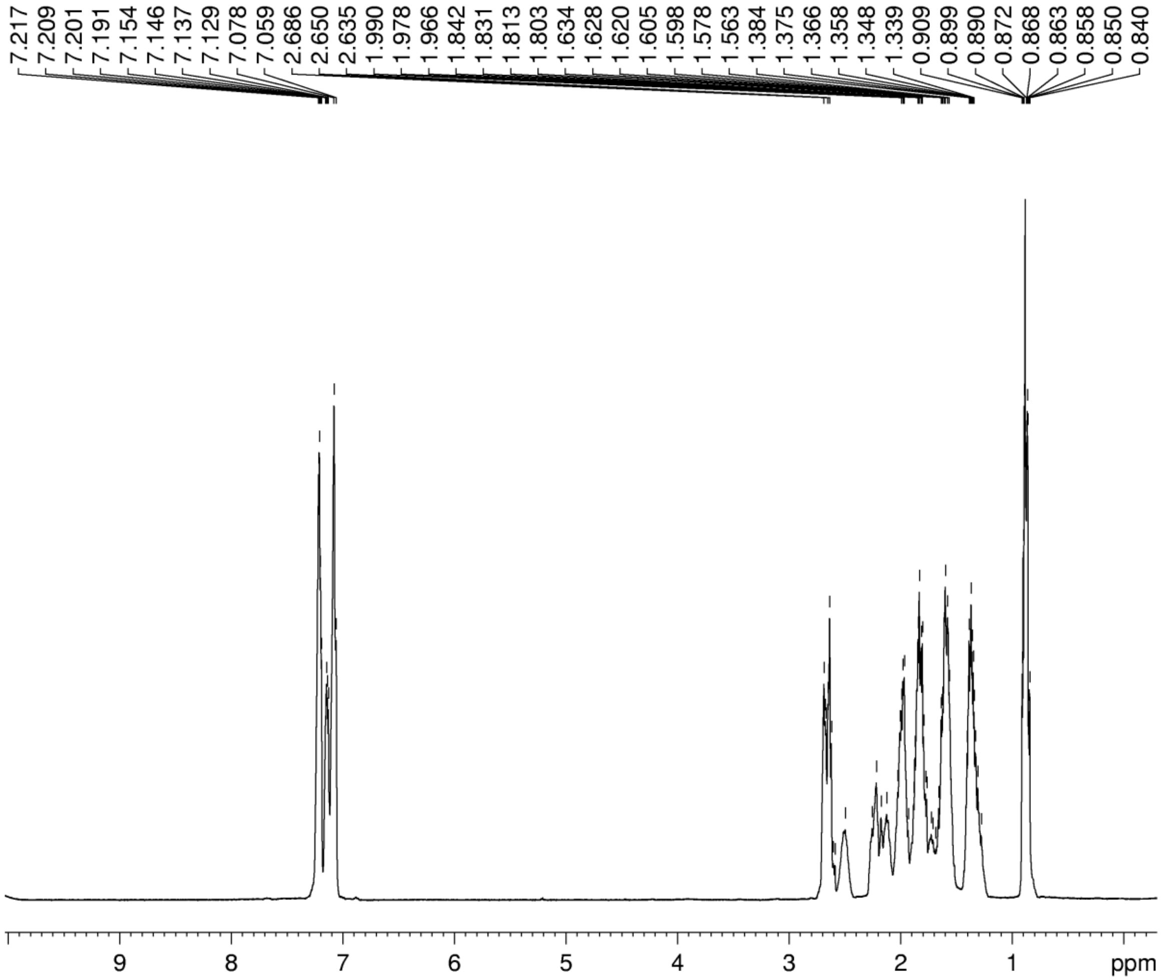

Current Data Parameters
NAME KRA−289−1_1_13
EXPNO 1
PROCNO 1

F2 − Acquisition Parameters
Date_ 20150219
Time 13.23
INSTRUM spect
PROBHD 5 mm PABBO BB−
PULPROG zg30
TD 16384
SOLVENT CDCl3
NS 1
DS 0
SWH 4132.231 Hz
FIDRES 0.252211 Hz
AQ 1.9824640 sec
RG 16
DW 121.000 usec
DE 6.50 usec
TE 298.5 K
D1 1.00000000 sec
TD0 1

======== CHANNEL f1 ======
SFO1 400.1319468 MHz
NUC1 1H
P1 14.80 usec
PLW1 8.89999962 W

F2 − Processing parameters
SI 16384
SF 400.1300000 MHz
WDW no
SSB 0
LB 0 Hz
GB 0
PC 1.00

^{13}C NMR ($CDCl_3$): **4h**

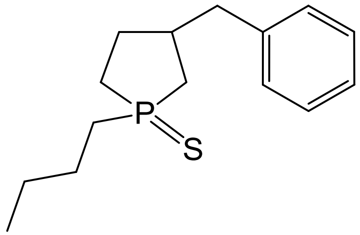

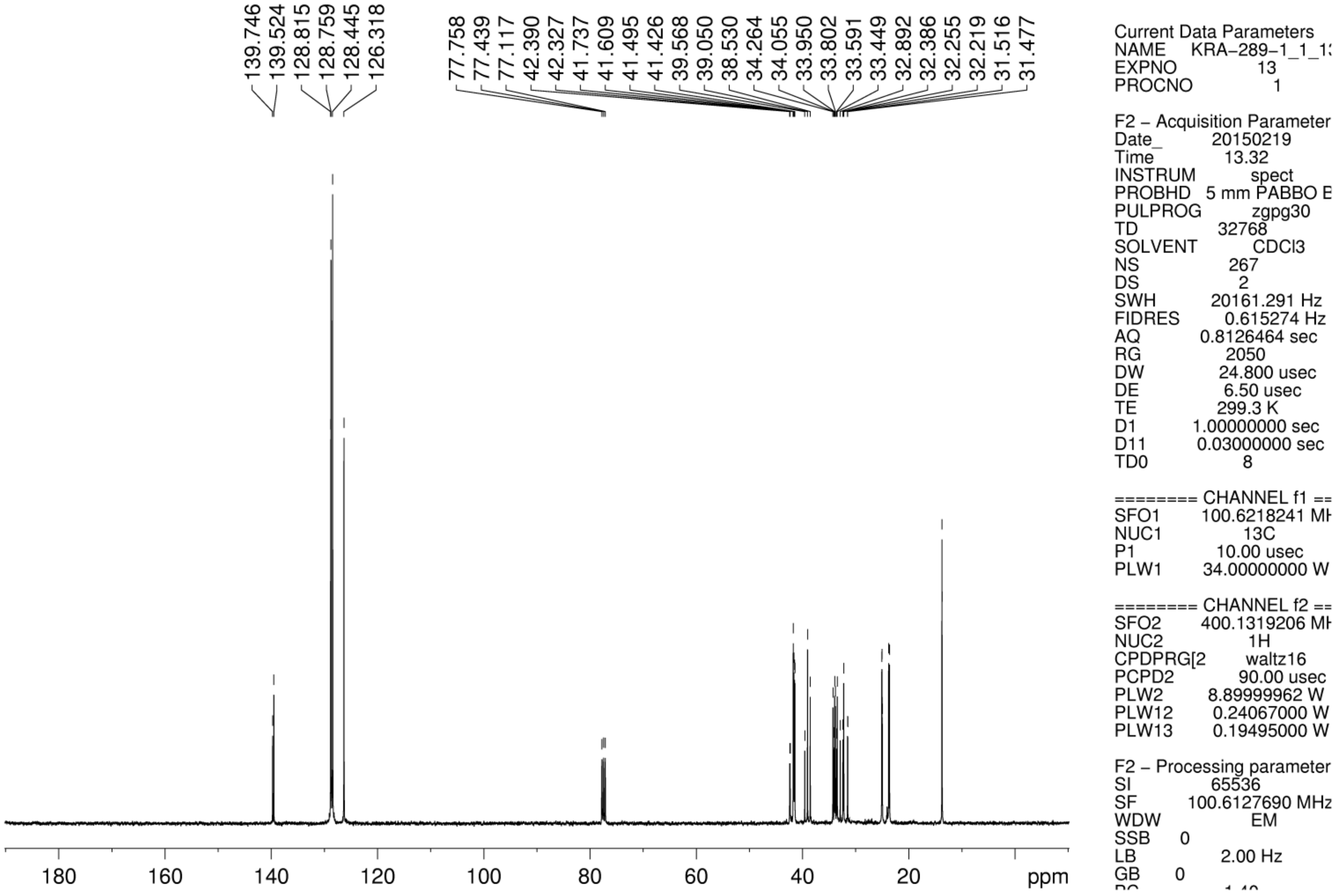

^{31}P NMR ($CDCl_3$): **4h**

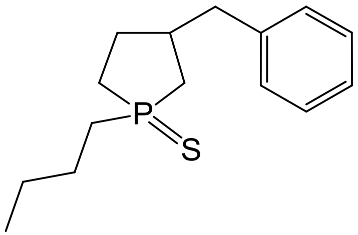

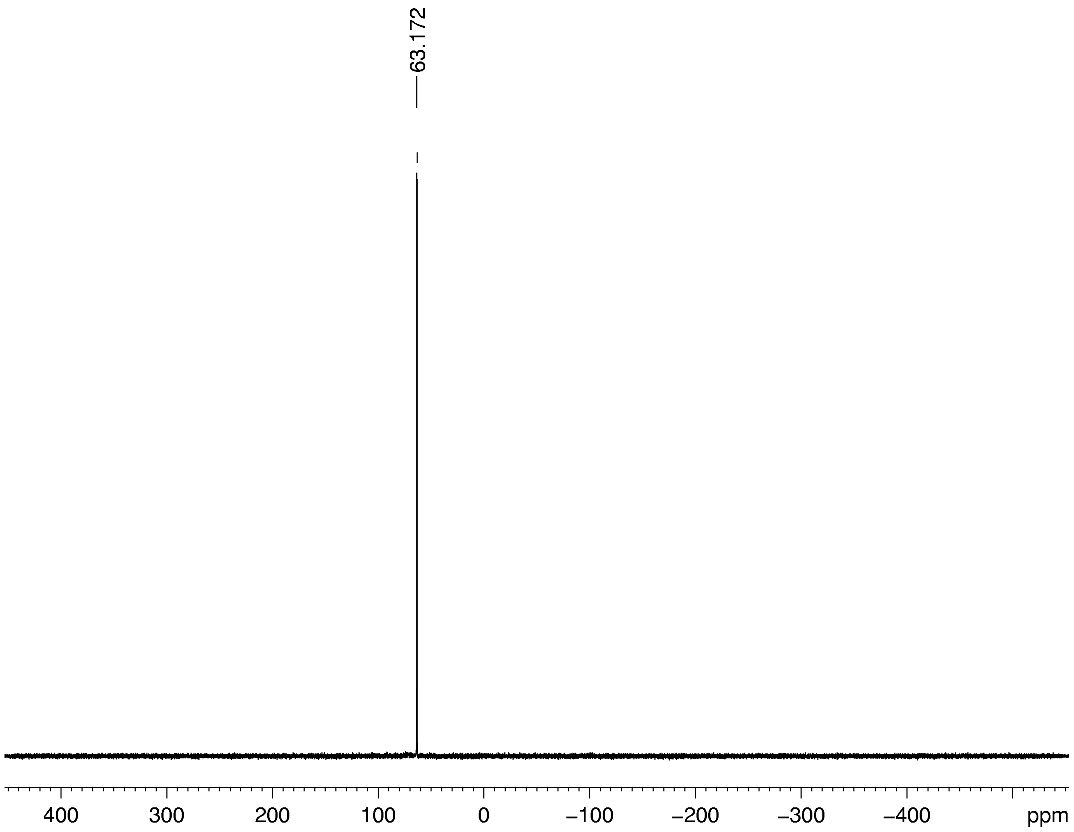

Current Data Parameters
NAME KRA–289–1_1_13
EXPNO 31
PROCNO 1

F2 – Acquisition Parameters
Date_ 20150219
Time 13.19
INSTRUM spect
PROBHD 5 mm PABBO BB–
PULPROG zg30
TD 65536
SOLVENT CDCl3
NS 25
DS 4
SWH 163043.484 Hz
FIDRES 2.487846 Hz
AQ 0.2009771 sec
RG 2050
DW 3.067 usec
DE 6.50 usec
TE 298.5 K
D1 2.00000000 sec
TD0 1

======== CHANNEL f1 ====
SFO1 161.9674942 MHz
NUC1 31P
P1 10.00 usec
PLW1 25.00000000 W

F2 – Processing parameters
SI 32768
SF 161.9755930 MHz
WDW EM
SSB 0
LB 1.00 Hz
GB 0
PC 1.40

^{1}H NMR ($CDCl_3$): **10a**

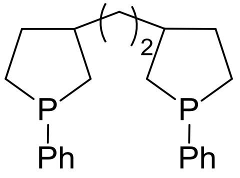

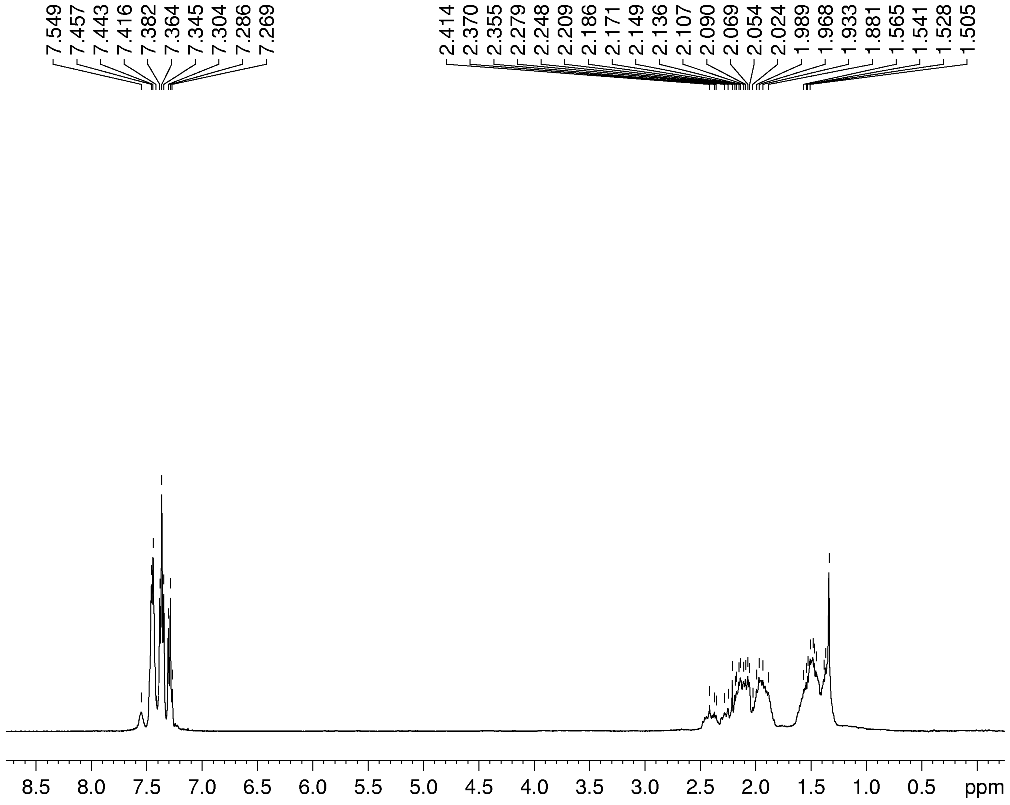

Current Data Parameters
NAME KRA–335_1_13_11
EXPNO 1
PROCNO 1

F2 – Acquisition Parameters
Date_ 20150326
Time 15.17
INSTRUM spect
PROBHD 5 mm PABBO BB-
PULPROG zg30
TD 16384
SOLVENT CDCl3
NS 1
DS 0
SWH 5081.301 Hz
FIDRES 0.310138 Hz
AQ 1.6121856 sec
RG 36
DW 98.400 usec
DE 6.50 usec
TE 298.3 K
D1 1.00000000 sec
TD0 1

======== CHANNEL f1 ====
SFO1 400.1322666 MHz
NUC1 1H
P1 14.80 usec
PLW1 8.89999962 W

F2 – Processing parameters
SI 16384
SF 400.1300000 MHz
WDW no
SSB 0
LB 0 Hz
GB 0
PC 1.00

^{13}C NMR ($CDCl_3$): **10a**

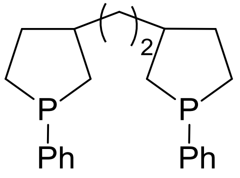

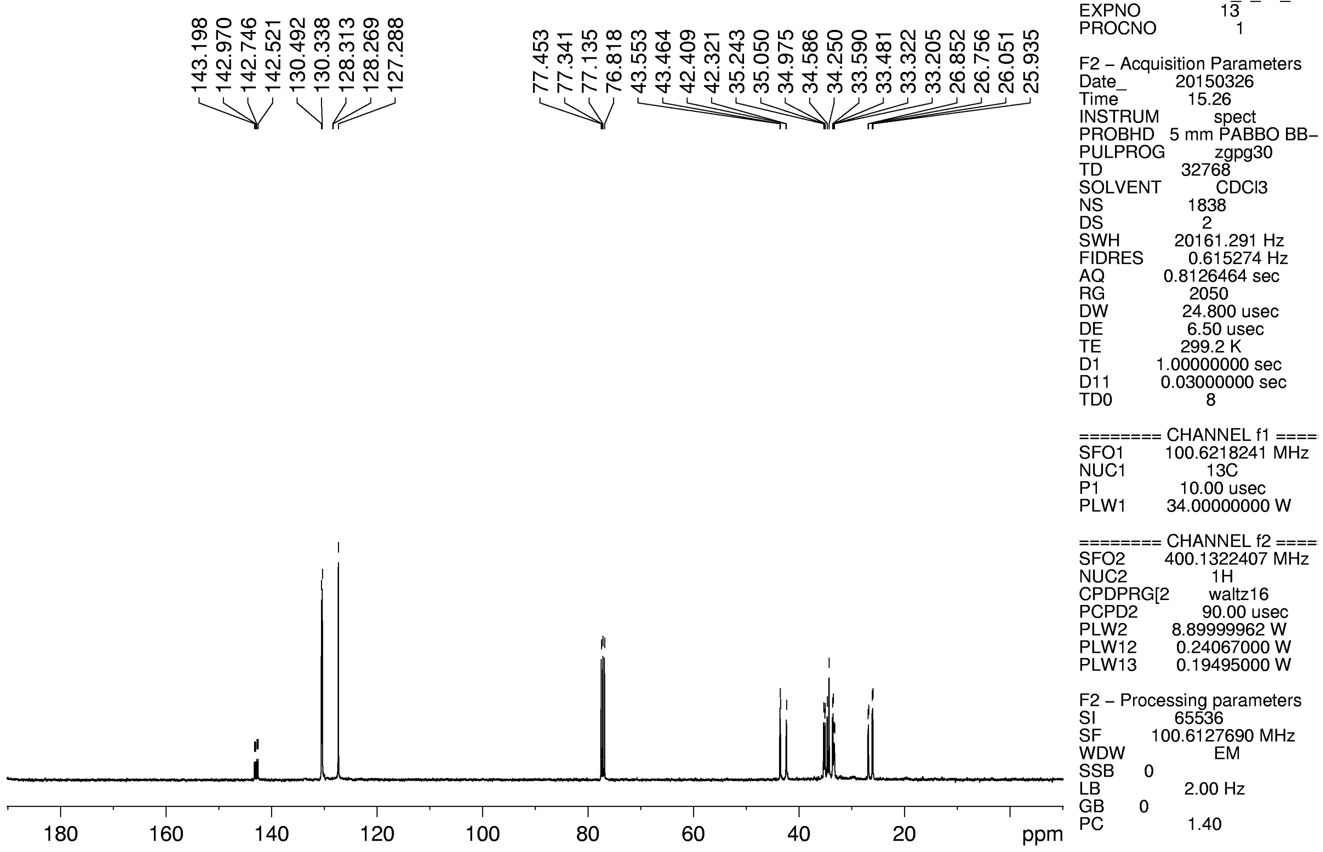

^{31}P NMR (CDCl$_3$): **10a**

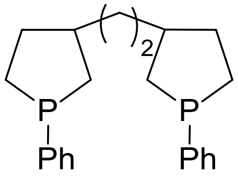

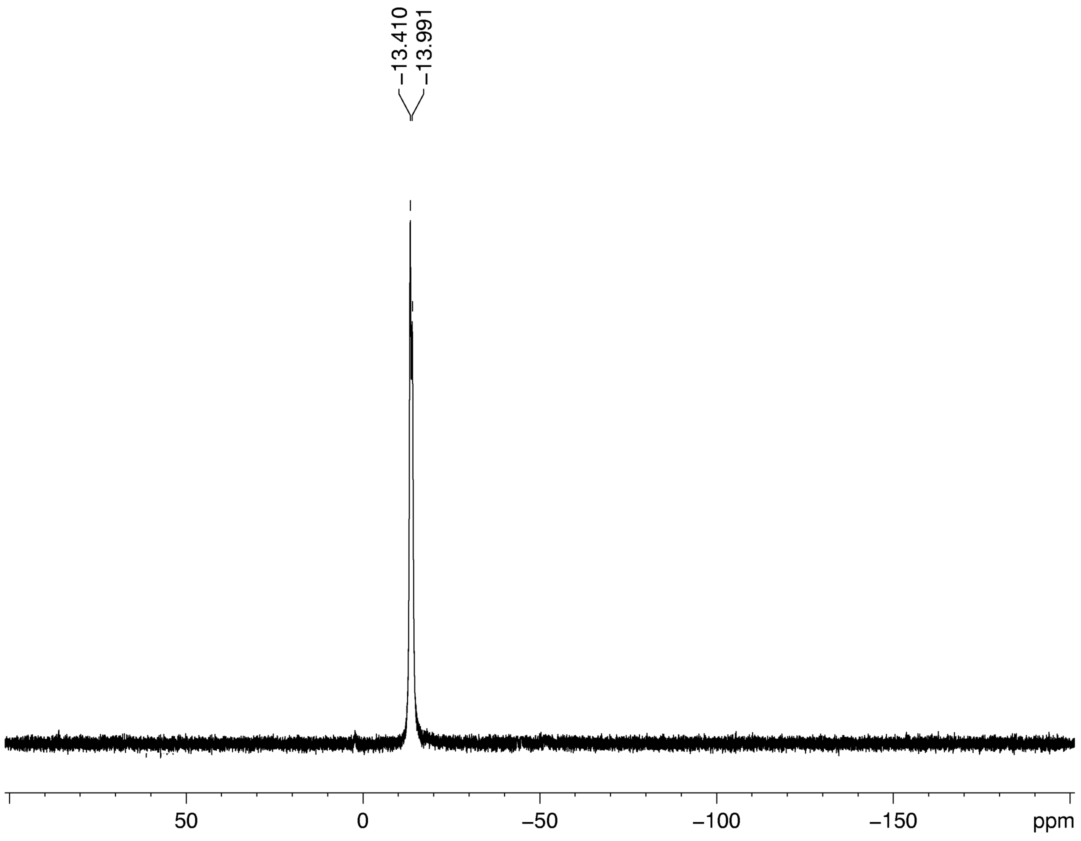

Current Data Parameters
NAME KRA−335_1_13_11
EXPNO 31
PROCNO 1

F2 − Acquisition Parameters
Date_ 20150326
Time 14.57
INSTRUM spect
PROBHD 5 mm PABBO BB-
PULPROG zg30
TD 65536
SOLVENT CDCl3
NS 32
DS 4
SWH 163043.484 Hz
FIDRES 2.487846 Hz
AQ 0.2009771 sec
RG 2050
DW 3.067 usec
DE 6.50 usec
TE 298.4 K
D1 2.00000000 sec
TD0 1

======== CHANNEL f1 ====
SFO1 161.9674942 MHz
NUC1 31P
P1 10.00 usec
PLW1 25.00000000 W

F2 − Processing parameters
SI 32768
SF 161.9755930 MHz
WDW EM
SSB 0
LB 1.00 Hz
GB 0
PC 1.40

^{13}C NMR ($CDCl_3$): **10e**

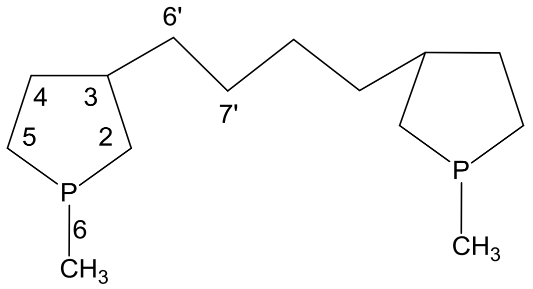

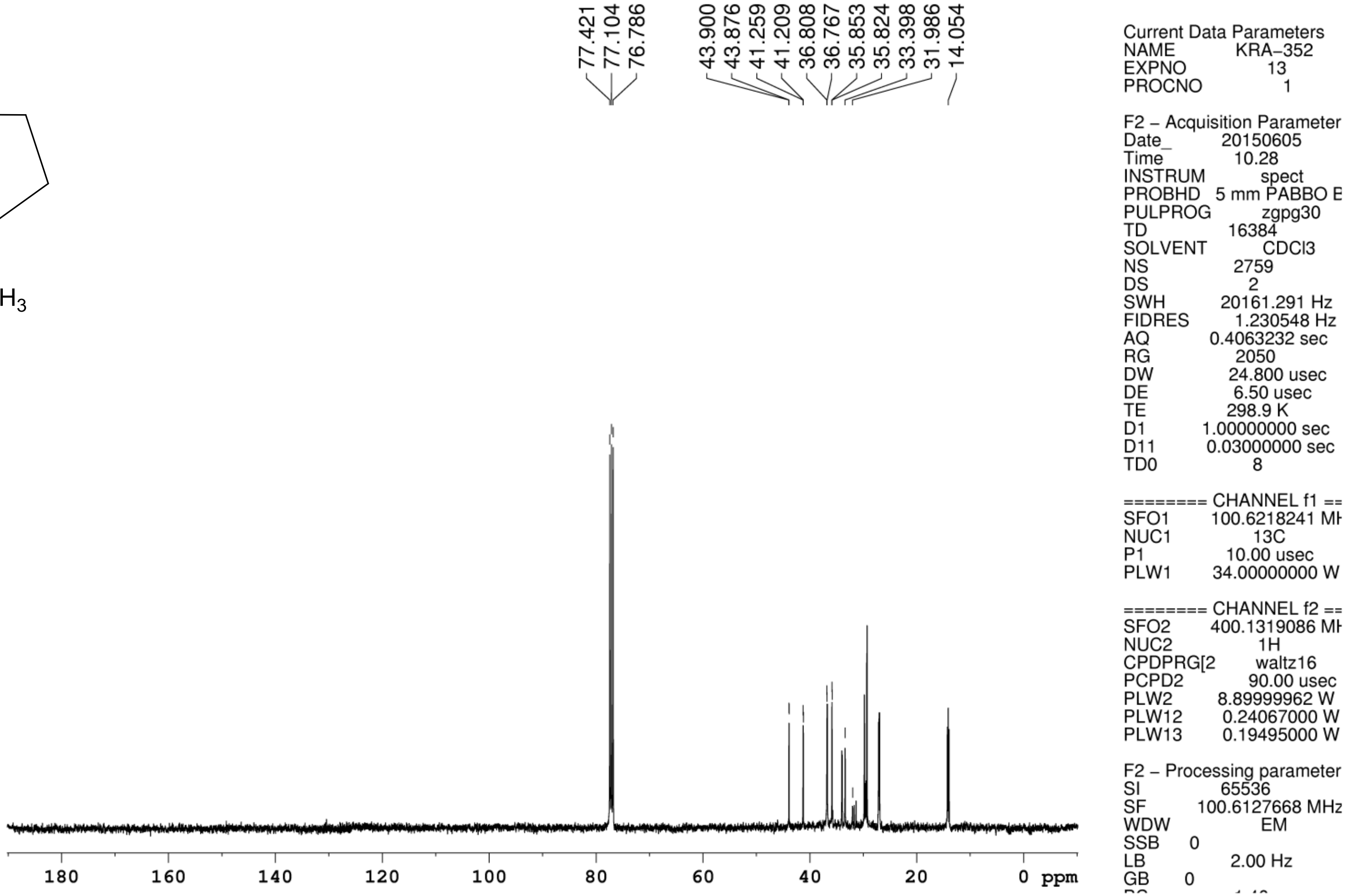

^{31}P NMR ($CDCl_3$): **10e**

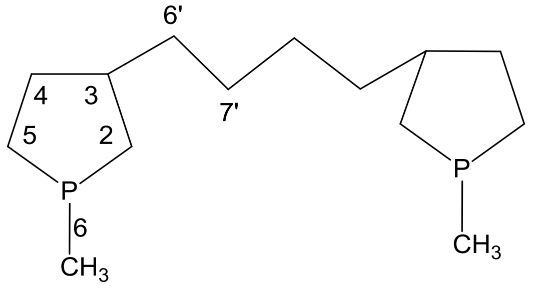

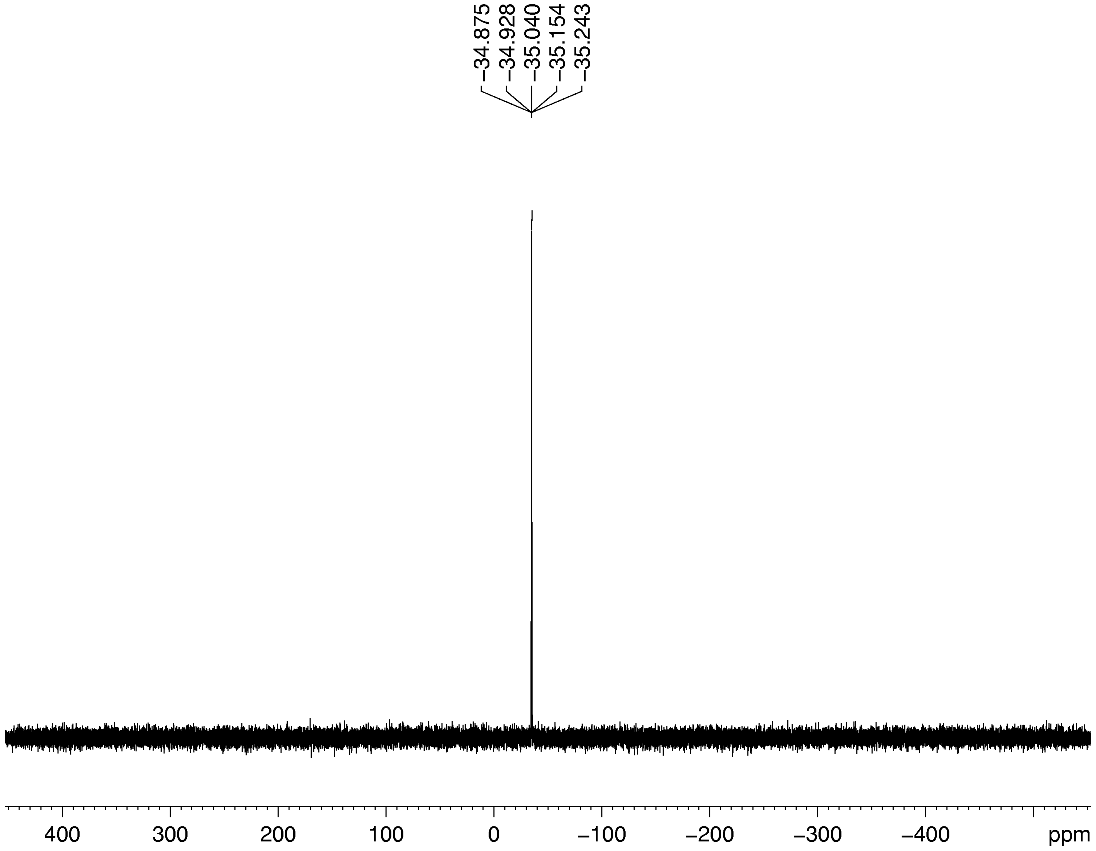

Current Data Parameters
NAME KRA−352
EXPNO 31
PROCNO 1

F2 − Acquisition Parameters
Date_ 20150604
Time 21.34
INSTRUM spect
PROBHD 5 mm PABBO BB−
PULPROG zg30
TD 65536
SOLVENT CDCl3
NS 64
DS 4
SWH 163043.484 Hz
FIDRES 2.487846 Hz
AQ 0.2009771 sec
RG 2050
DW 3.067 usec
DE 6.50 usec
TE 298.4 K
D1 2.00000000 sec
TD0 1

======== CHANNEL f1 ====
SFO1 161.9674942 MHz
NUC1 31P
P1 10.00 usec
PLW1 25.00000000 W

F2 − Processing parameters
SI 32768
SF 161.9755930 MHz
WDW no
SSB 0
LB 0 Hz
GB 0
PC 1.40

HPLC analysis: **11b**

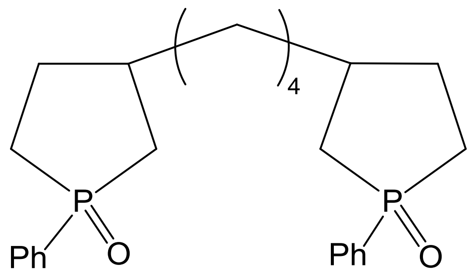

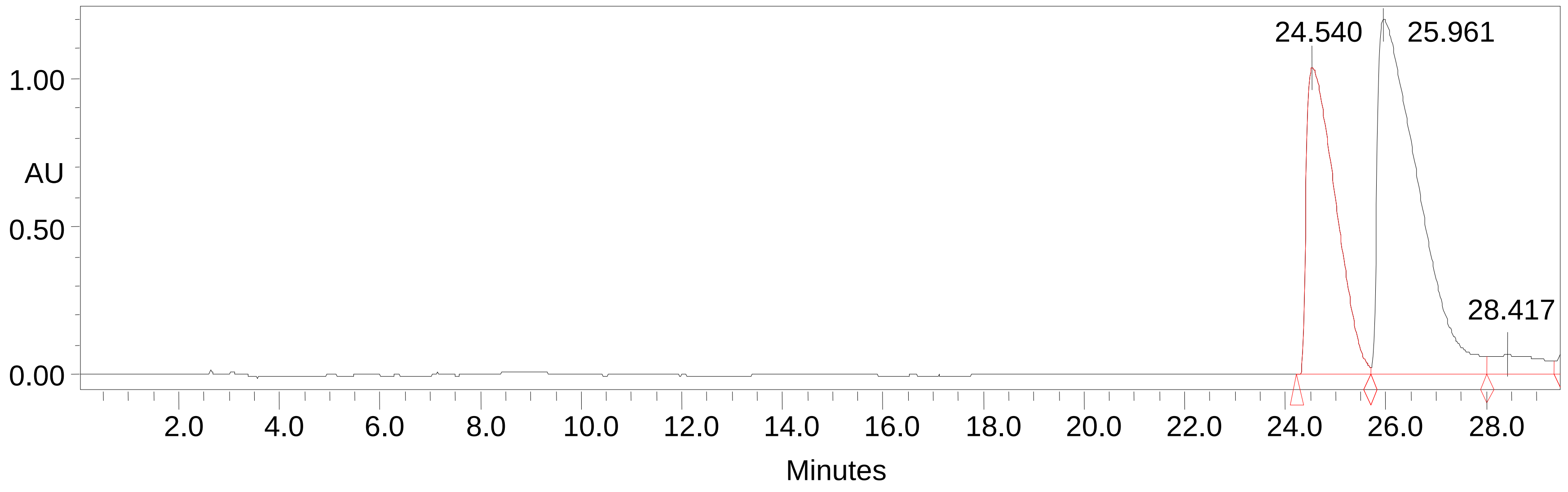

Figure S1. HPLC analysis for compound **11b**.

^{1}H NMR ($CDCl_3$): **11b**

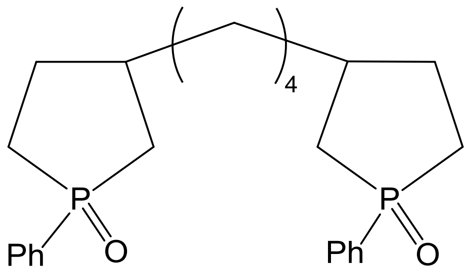

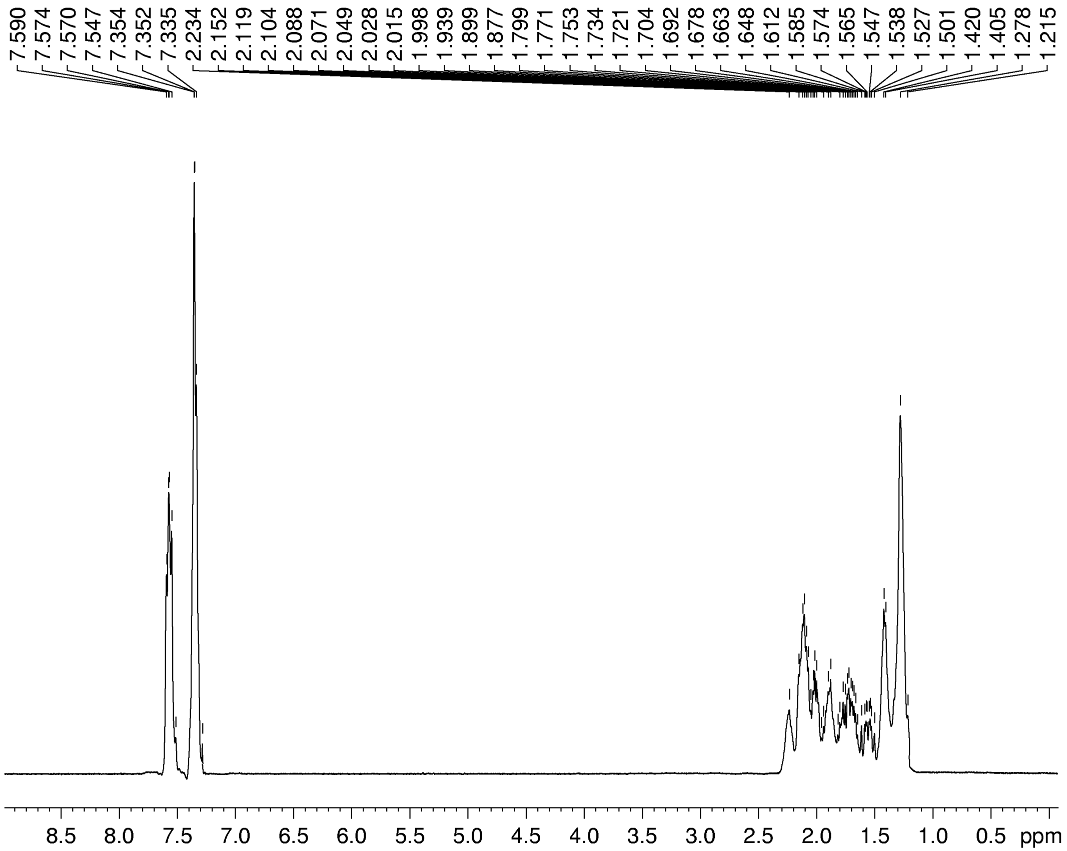

Current Data Parameters
NAME KRA–331–3_1_13
EXPNO 1
PROCNO 1

F2 – Acquisition Parameters
Date_ 20150403
Time 10.09
INSTRUM spect
PROBHD 5 mm PABBO BB-
PULPROG zg30
TD 16384
SOLVENT CDCl3
NS 1
DS 0
SWH 8012.820 Hz
FIDRES 0.489064 Hz
AQ 1.0223616 sec
RG 36
DW 62.400 usec
DE 6.50 usec
TE 298.5 K
D1 1.00000000 sec
TD0 1

======== CHANNEL f1 ====
SFO1 400.1332010 MHz
NUC1 1H
P1 14.80 usec
PLW1 8.89999962 W

F2 – Processing parameters
SI 16384
SF 400.1300000 MHz
WDW no
SSB 0
LB 0 Hz
GB 0
PC 1.00

^{13}C NMR ($CDCl_3$): **11b**

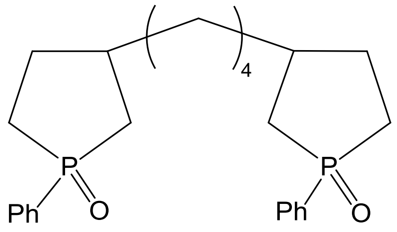

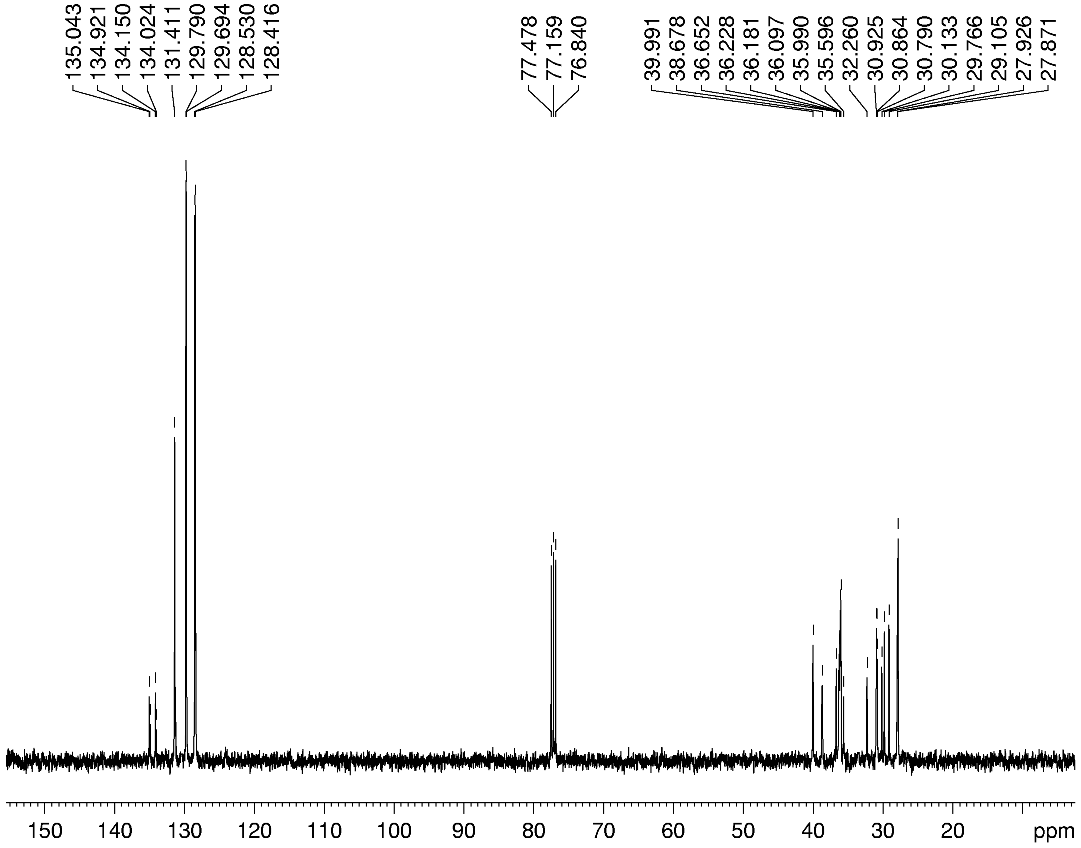

Current Data Parameters
NAME KRA-331-3_1_13
EXPNO 13
PROCNO 1

F2 - Acquisition Parameters
Date_ 20150403
Time 10.13
INSTRUM spect
PROBHD 5 mm PABBO BB-
PULPROG zgpg30
TD 32768
SOLVENT CDCl3
NS 125
DS 2
SWH 25252.525 Hz
FIDRES 0.770646 Hz
AQ 0.6488064 sec
RG 2050
DW 19.800 usec
DE 6.50 usec
TE 299.1 K
D1 1.00000000 sec
D11 0.03000000 sec
TD0 8

======== CHANNEL f1 ====
SFO1 100.6248425 MHz
NUC1 13C
P1 10.00 usec
PLW1 34.00000000 W

======== CHANNEL f2 ====
SFO2 400.1316005 MHz
NUC2 1H
CPDPRG[2 waltz16
PCPD2 90.00 usec
PLW2 8.89999962 W
PLW12 0.24067000 W
PLW13 0.19495000 W

F2 - Processing parameters
SI 65536
SF 100.6127690 MHz
WDW EM
SSB 0
LB 2.00 Hz
GB 0
PC 1.40

^{31}P NMR ($CDCl_3$): **11b**

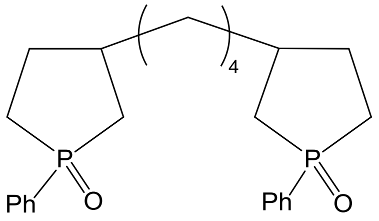

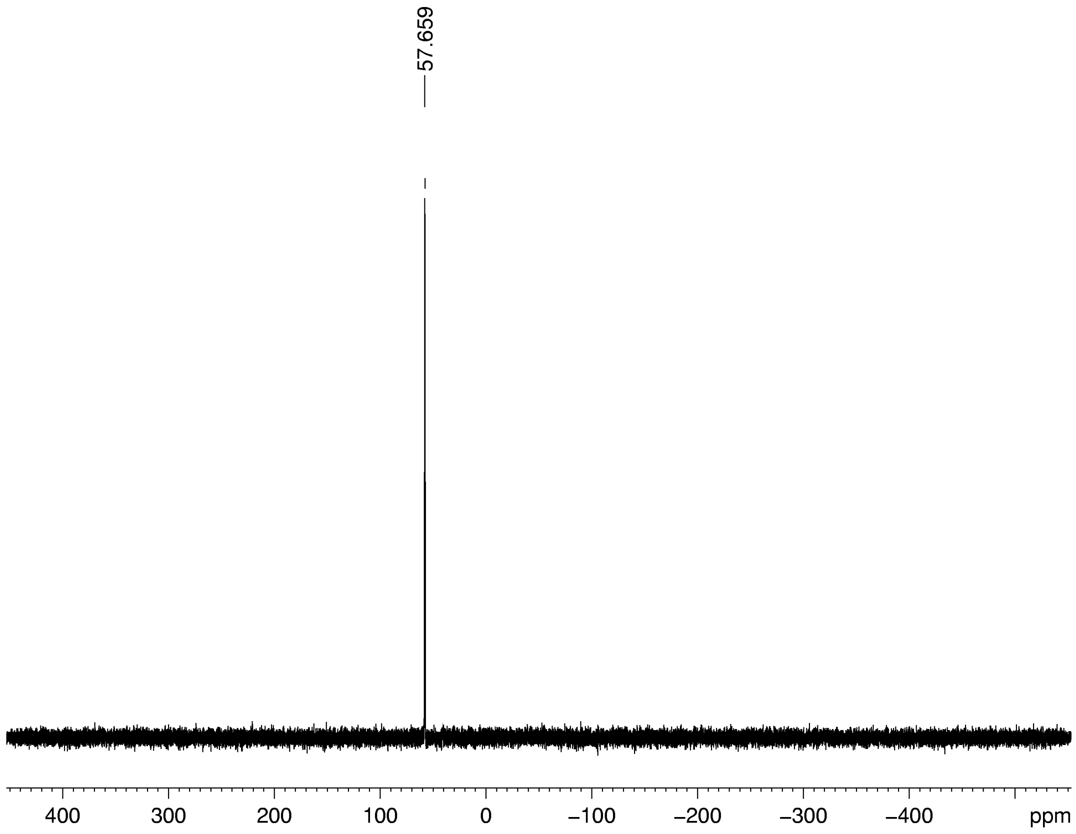

Current Data Parameters
NAME KRA–331–3_1_13
EXPNO 31
PROCNO 1

F2 – Acquisition Parameters
Date_ 20150403
Time 10.06
INSTRUM spect
PROBHD 5 mm PABBO BB–
PULPROG zg30
TD 65536
SOLVENT CDCl3
NS 32
DS 4
SWH 163043.484 Hz
FIDRES 2.487846 Hz
AQ 0.2009771 sec
RG 2050
DW 3.067 usec
DE 6.50 usec
TE 298.6 K
D1 2.00000000 sec
TD0 1

======== CHANNEL f1 ====
SFO1 161.9674942 MHz
NUC1 31P
P1 10.00 usec
PLW1 25.00000000 W

F2 – Processing parameters
SI 32768
SF 161.9755930 MHz
WDW EM
SSB 0
LB 1.00 Hz
GB 0
PC 1.40

^{1}H NMR ($CDCl_3$): **12c**

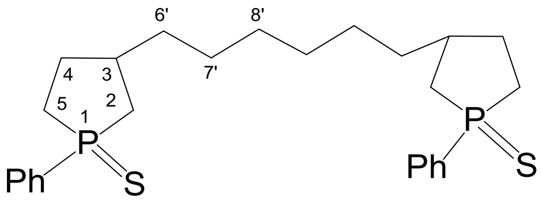

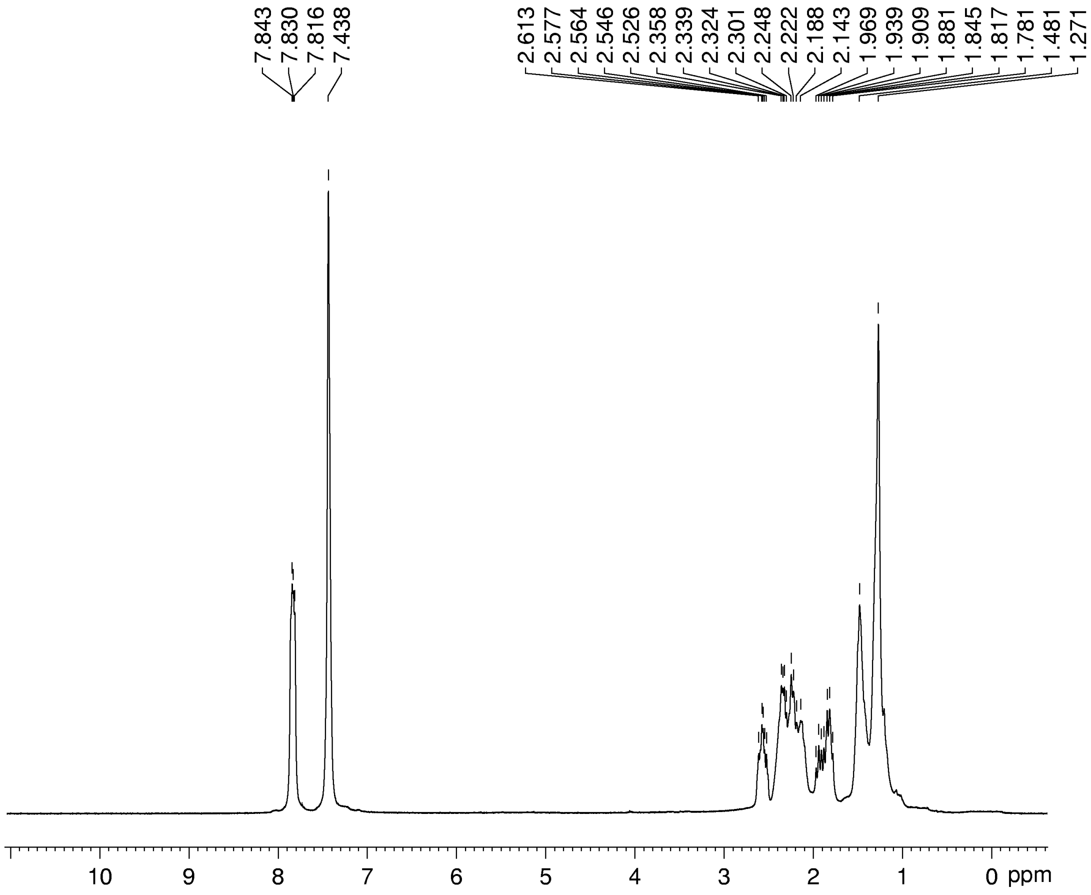

Current Data Parameters
NAME KRA–328–2_1_13
EXPNO 1
PROCNO 1

F2 – Acquisition Parameters
Date_ 20150219
Time 14.54
INSTRUM spect
PROBHD 5 mm PABBO BB-
PULPROG zg30
TD 16384
SOLVENT CDCl3
NS 1
DS 0
SWH 4973.475 Hz
FIDRES 0.303557 Hz
AQ 1.6471381 sec
RG 20.2
DW 100.533 usec
DE 6.50 usec
TE 298.5 K
D1 1.00000000 sec
TD0 1

======== CHANNEL f1 ====
SFO1 400.1319409 MHz
NUC1 1H
P1 14.80 usec
PLW1 8.89999962 W

F2 – Processing parameters
SI 16384
SF 400.1300000 MHz
WDW no
SSB 0
LB 0 Hz
GB 0
PC 1.00

^{13}C NMR ($CDCl_3$): **12c**

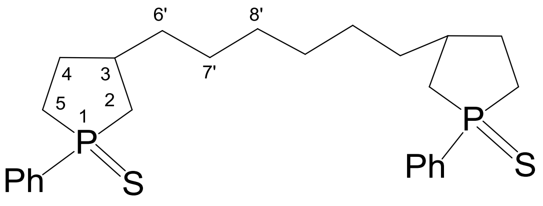

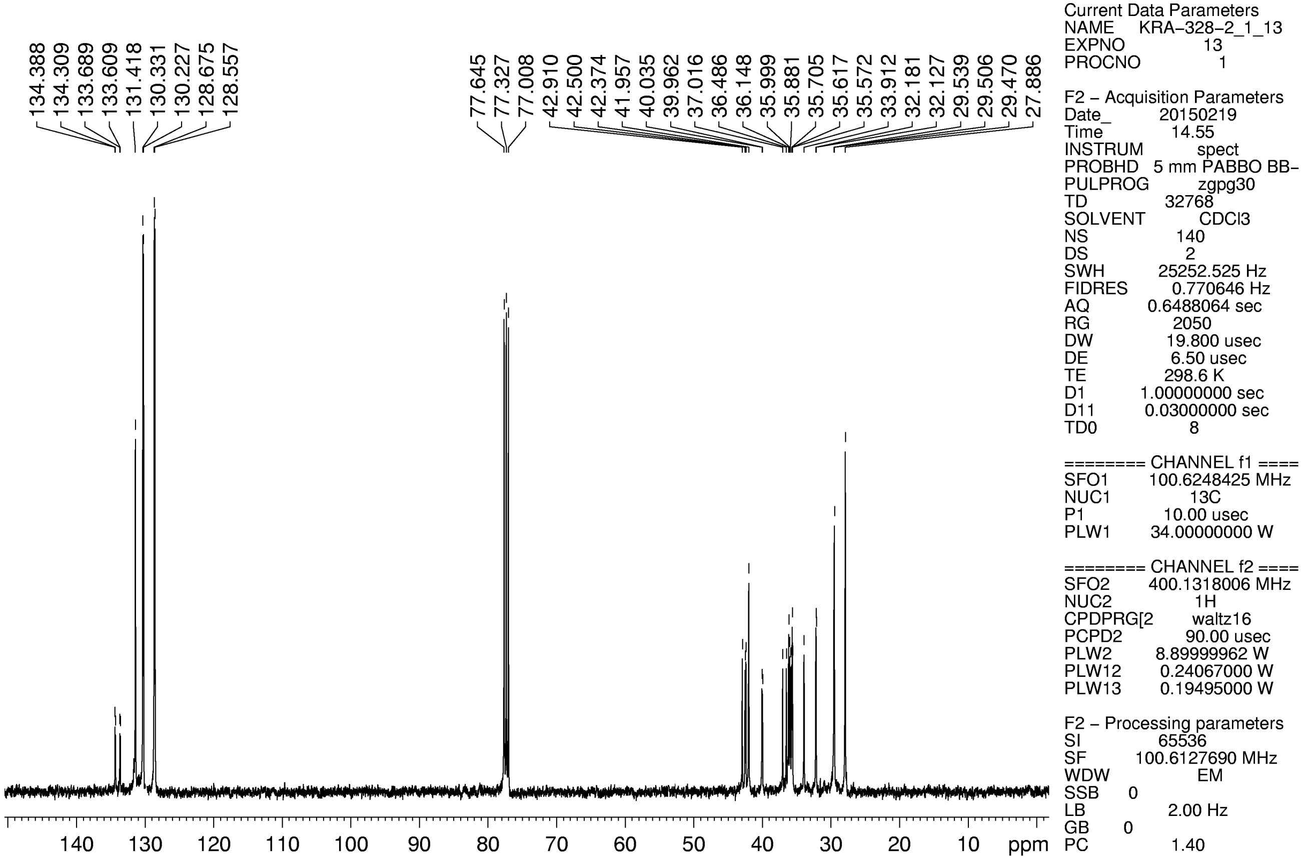

^{31}P NMR ($CDCl_3$): **12c**

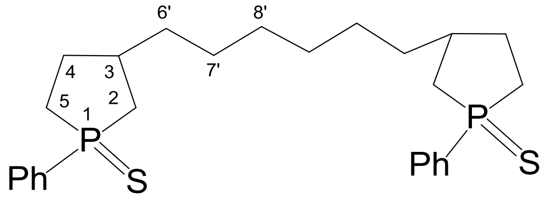

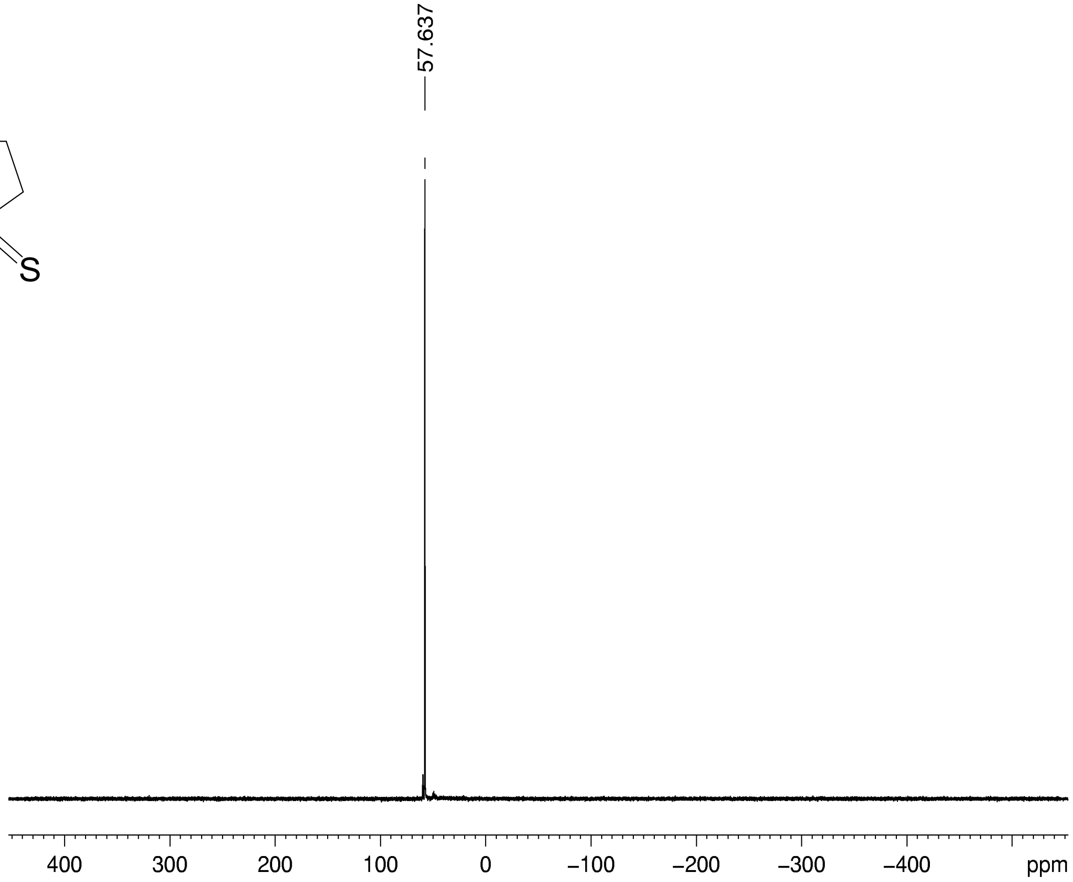

Current Data Parameters
NAME KRA−328−2_1_13
EXPNO 31
PROCNO 1

F2 − Acquisition Parameters
Date_ 20150219
Time 14.50
INSTRUM spect
PROBHD 5 mm PABBO BB−
PULPROG zg30
TD 65536
SOLVENT CDCl3
NS 29
DS 4
SWH 163043.484 Hz
FIDRES 2.487846 Hz
AQ 0.2009771 sec
RG 2050
DW 3.067 usec
DE 6.50 usec
TE 298.5 K
D1 2.00000000 sec
TD0 1

======== CHANNEL f1 ====
SFO1 161.9674942 MHz
NUC1 31P
P1 10.00 usec
PLW1 25.00000000 W

F2 − Processing parameters
SI 32768
SF 161.9755930 MHz
WDW EM
SSB 0
LB 1.00 Hz
GB 0
PC 1.40

^{1}H NMR ($CDCl_3$): **13b**

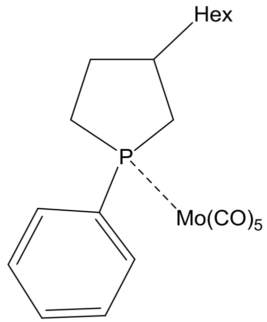

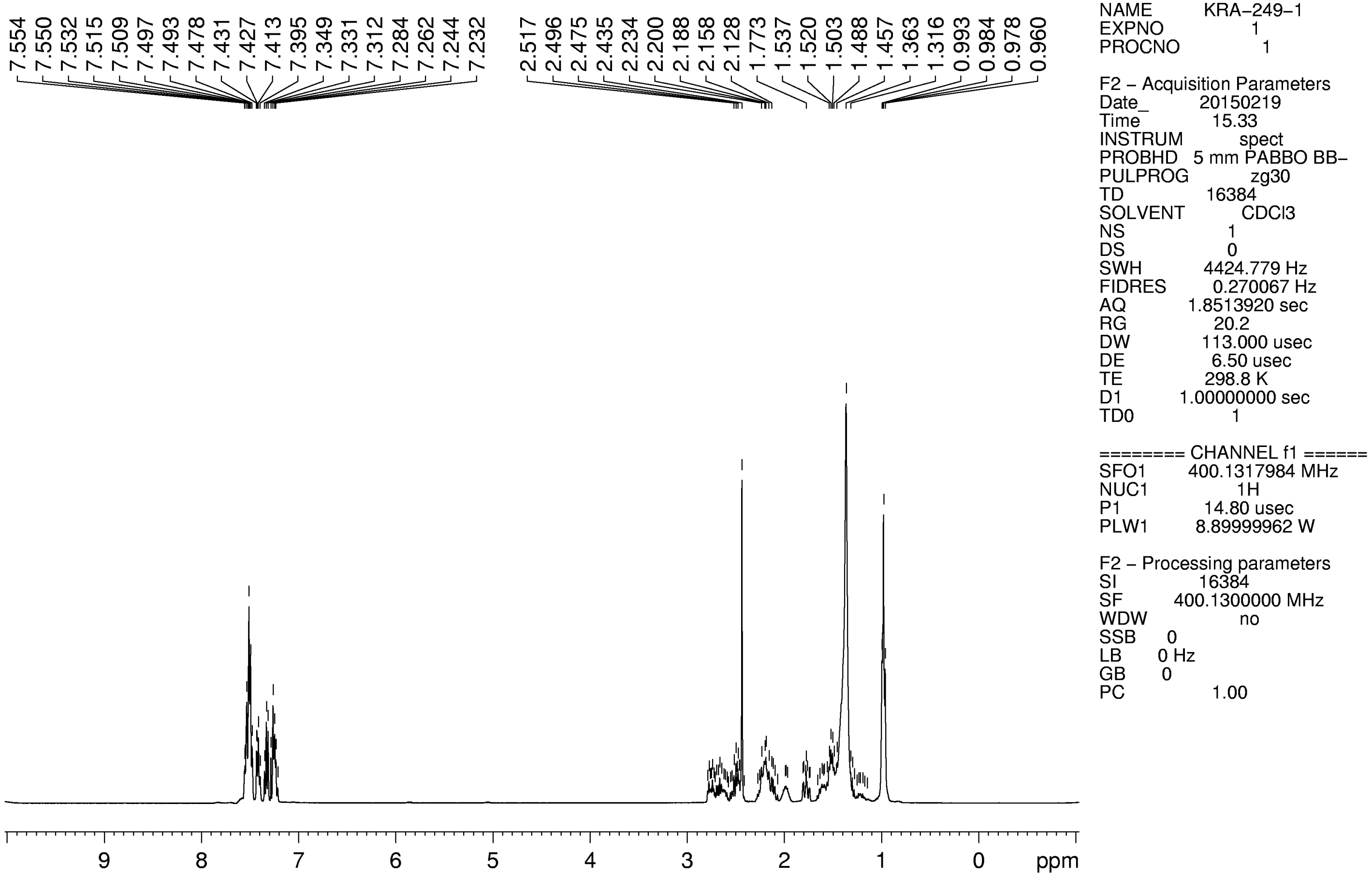

^{13}C NMR ($CDCl_3$): **13b**

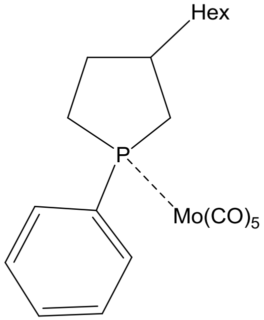

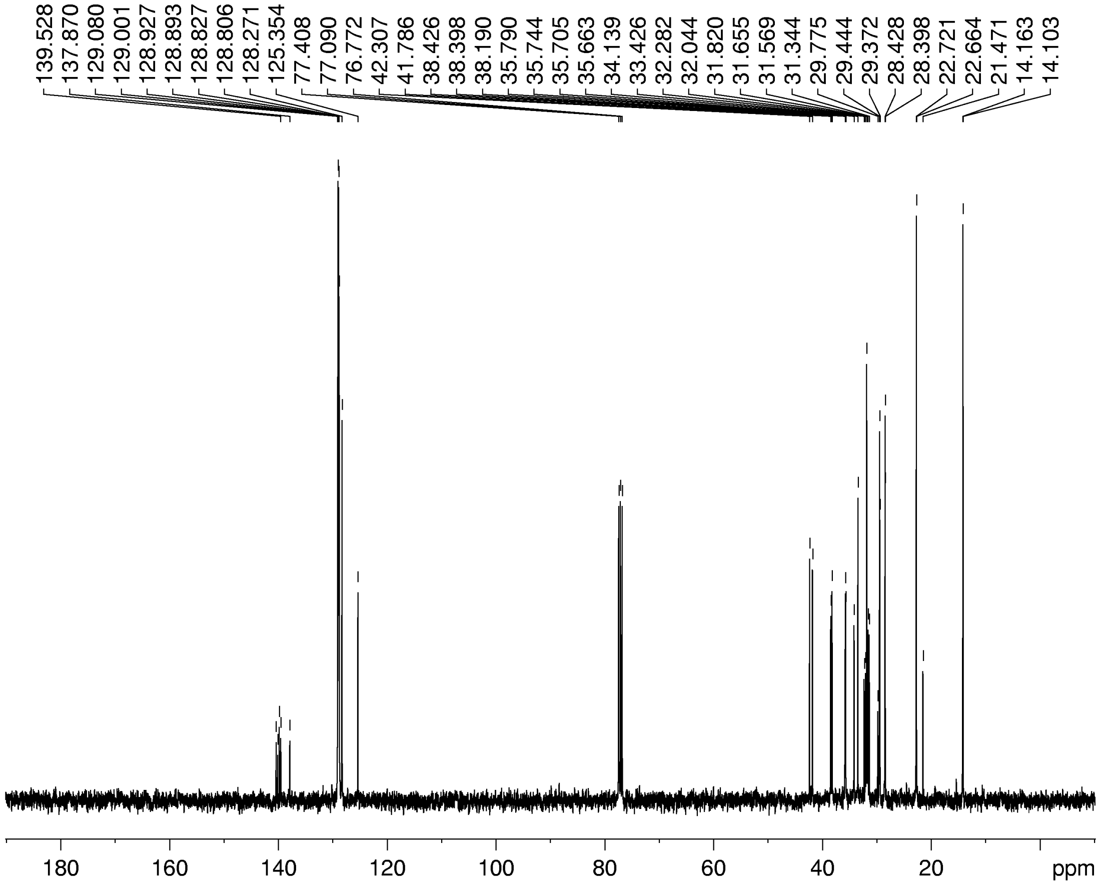

Current Data Parameters
NAME KRA-249-1
EXPNO 13
PROCNO 1

F2 - Acquisition Parameters
Date_ 20150219
Time 15.36
INSTRUM spect
PROBHD 5 mm PABBO BB-
PULPROG zgpg30
TD 32768
SOLVENT CDCl3
NS 97
DS 2
SWH 20161.291 Hz
FIDRES 0.615274 Hz
AQ 0.8126464 sec
RG 1440
DW 24.800 usec
DE 6.50 usec
TE 299.4 K
D1 1.00000000 sec
D11 0.03000000 sec
TD0 8

======== CHANNEL f1 ====
SFO1 100.6218241 MHz
NUC1 13C
P1 10.00 usec
PLW1 34.00000000 W

======== CHANNEL f2 ====
SFO2 400.1318006 MHz
NUC2 1H
CPDPRG[2 waltz16
PCPD2 90.00 usec
PLW2 8.89999962 W
PLW12 0.24067000 W
PLW13 0.19495000 W

F2 - Processing parameters
SI 65536
SF 100.6127690 MHz
WDW EM
SSB 0
LB 2.00 Hz
GB 0
[illegible]

^{31}P NMR ($CDCl_3$): **13b**

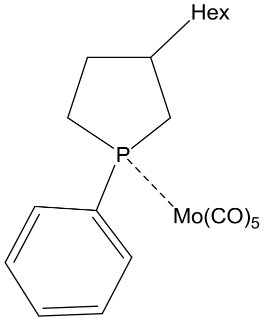

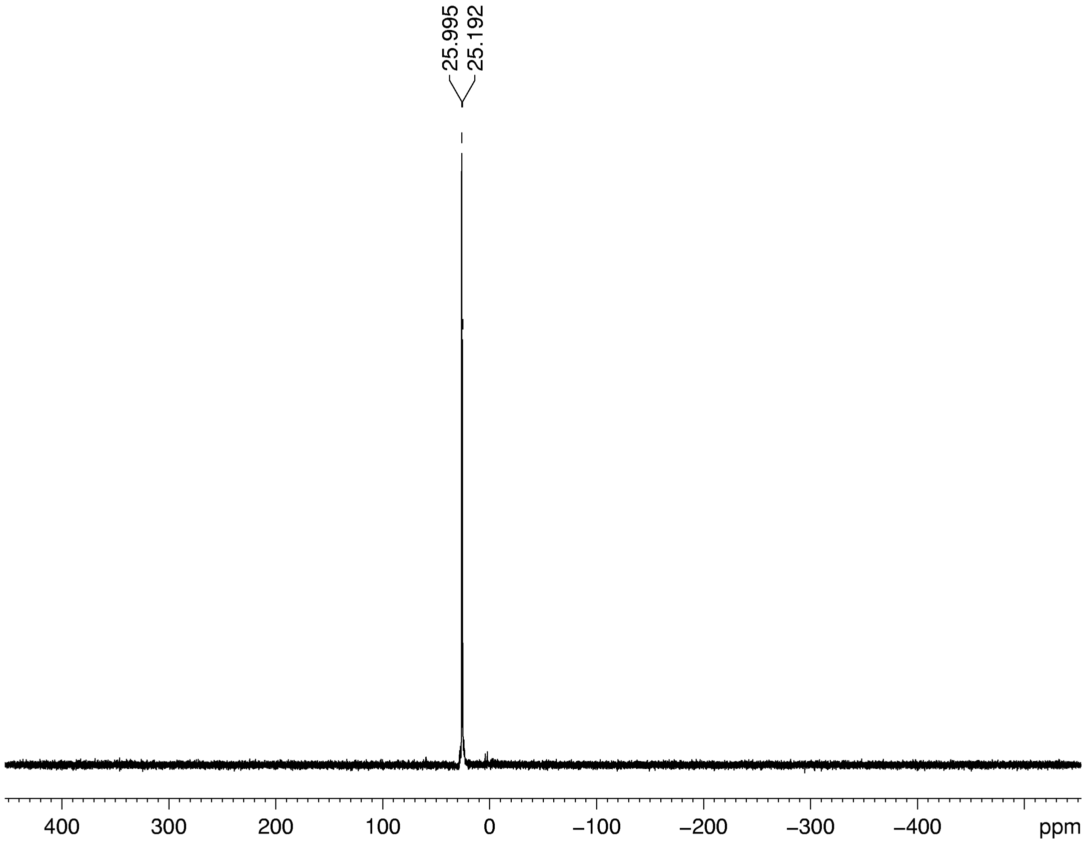

Current Data Parameters
NAME KRA−249−1
EXPNO 31
PROCNO 1

F2 − Acquisition Parameters
Date_ 20150219
Time 15.30
INSTRUM spect
PROBHD 5 mm PABBO BB-
PULPROG zg30
TD 65536
SOLVENT CDCl3
NS 32
DS 4
SWH 163043.484 Hz
FIDRES 2.487846 Hz
AQ 0.2009771 sec
RG 2050
DW 3.067 usec
DE 6.50 usec
TE 298.8 K
D1 2.00000000 sec
TD0 1

======== CHANNEL f1 ====
SFO1 161.9674942 MHz
NUC1 31P
P1 10.00 usec
PLW1 25.00000000 W

F2 − Processing parameters
SI 32768
SF 161.9755930 MHz
WDW EM
SSB 0
LB 1.00 Hz
GB 0
PC 1.40

^{1}H NMR ($CDCl_3$): **14g**

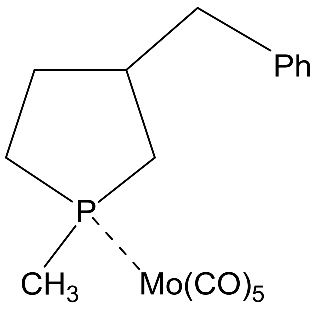

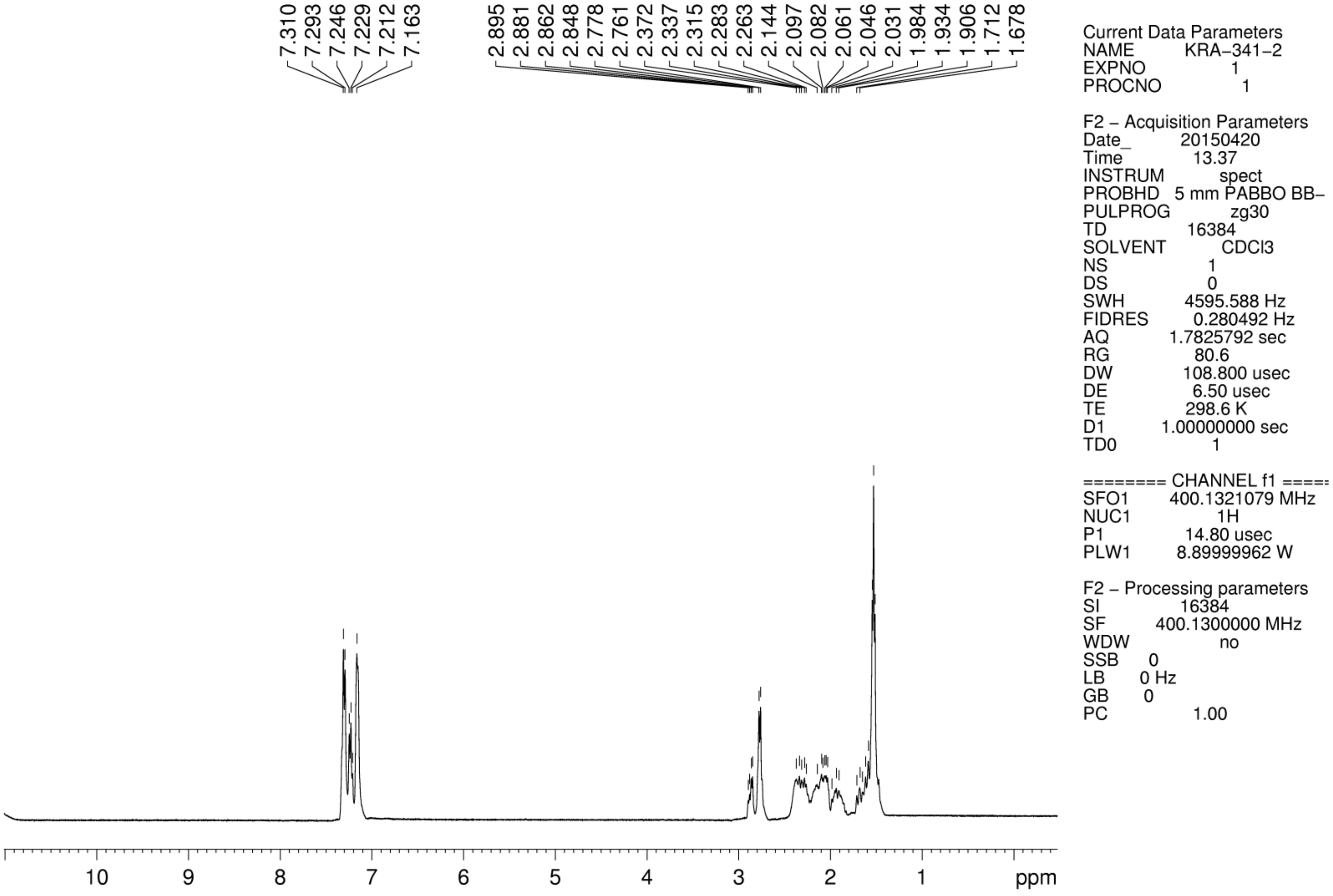

^{13}C NMR ($CDCl_3$): **14g**

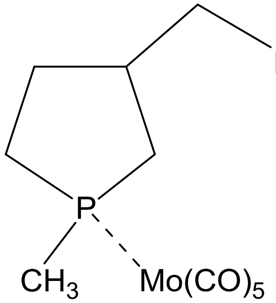

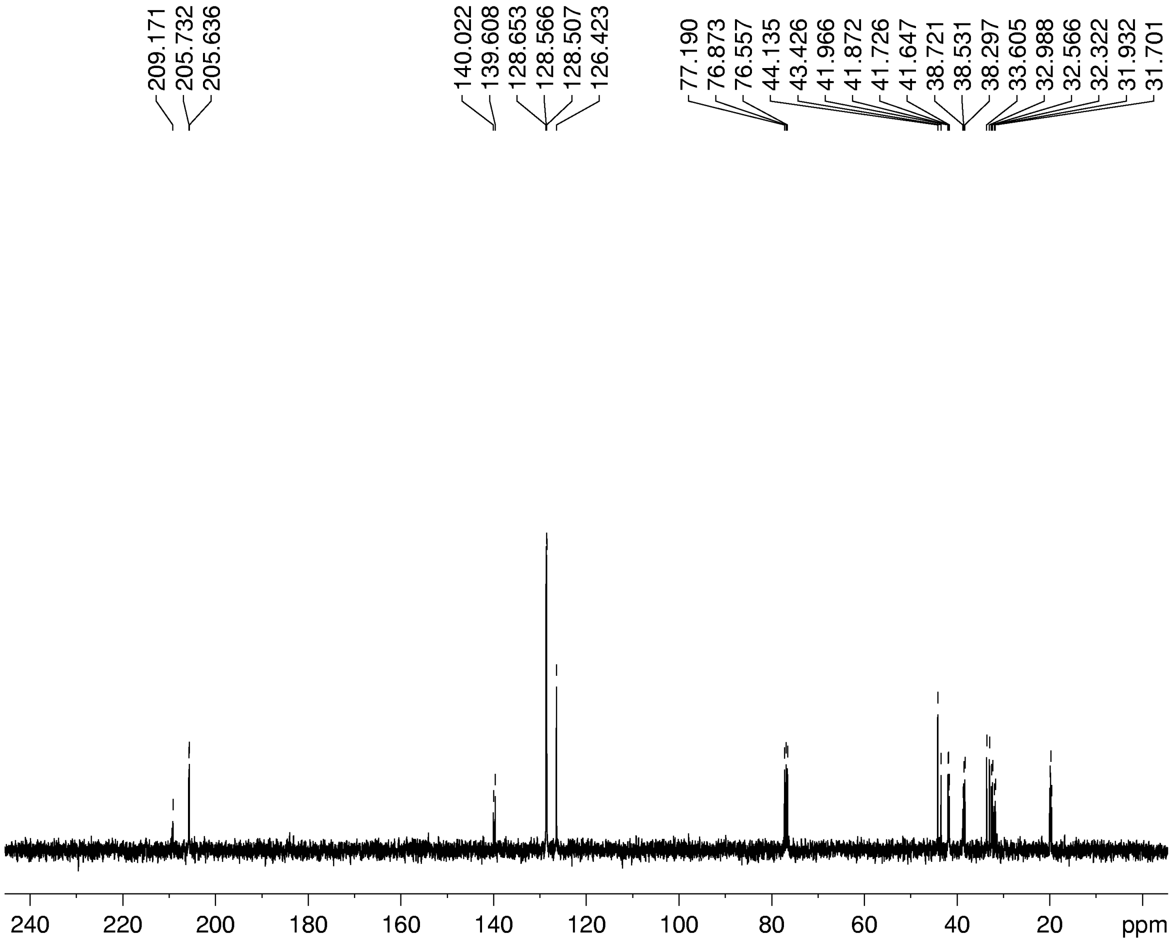

Current Data Parameters
NAME KRA−341−2_1_13_31
EXPNO 13
PROCNO 1

F2 − Acquisition Parameters
Date_ 20150420
Time 13.41
INSTRUM spect
PROBHD 5 mm PABBO BB−
PULPROG zgpg30
TD 32768
SOLVENT CDCl3
NS 100
DS 2
SWH 25252.525 Hz
FIDRES 0.770646 Hz
AQ 0.6488064 sec
RG 2050
DW 19.800 usec
DE 6.50 usec
TE 299.3 K
D1 1.00000000 sec
D11 0.03000000 sec
TD0 8

======== CHANNEL f1 ======
SFO1 100.6248425 MHz
NUC1 13C
P1 10.00 usec
PLW1 34.00000000 W

======== CHANNEL f2 ======
SFO2 400.1320007 MHz
NUC2 1H
CPDPRG[2 waltz16
PCPD2 90.00 usec
PLW2 8.89999962 W
PLW12 0.24067000 W
PLW13 0.19495000 W

F2 − Processing parameters
SI 65536
SF 100.6127690 MHz
WDW EM
SSB 0
LB 2.00 Hz
GB 0

^{31}P NMR ($CDCl_3$): **14g**

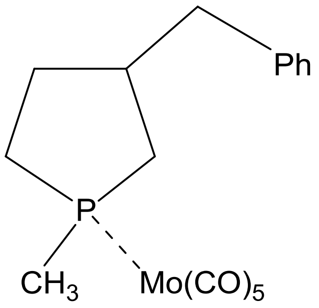

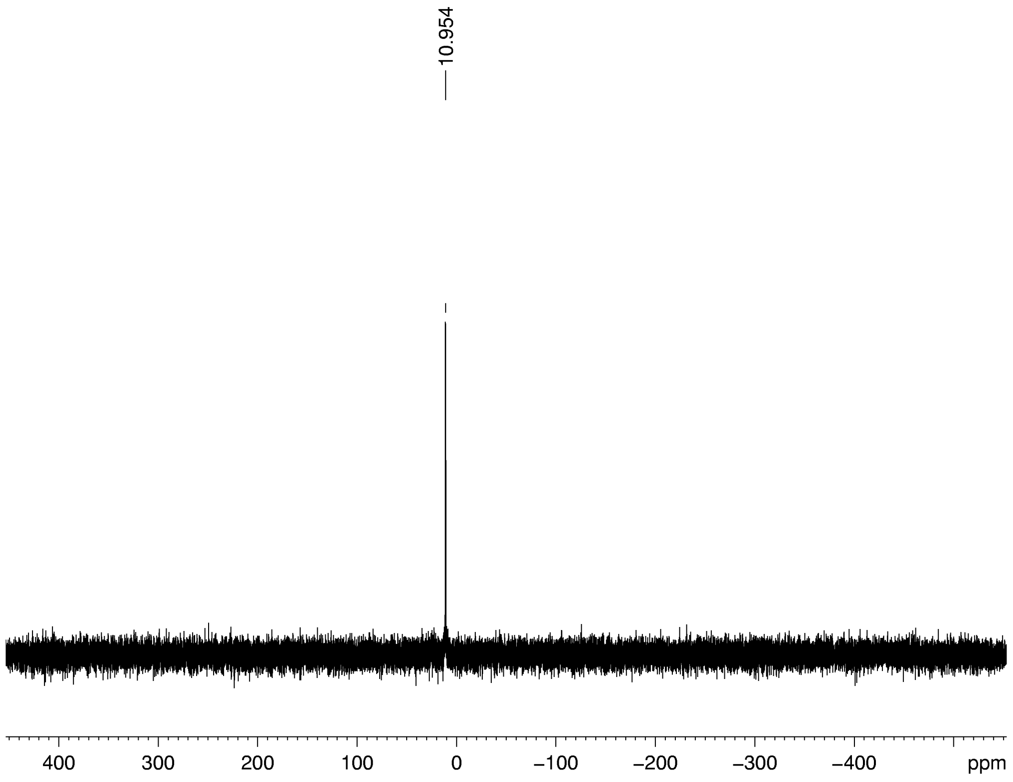

Current Data Parameters
NAME KRA−341−2_1_13_3
EXPNO 31
PROCNO 1

F2 − Acquisition Parameters
Date_ 20150420
Time 13.32
INSTRUM spect
PROBHD 5 mm PABBO BB−
PULPROG zg30
TD 65536
SOLVENT CDCl3
NS 32
DS 4
SWH 163043.484 Hz
FIDRES 2.487846 Hz
AQ 0.2009771 sec
RG 2050
DW 3.067 usec
DE 6.50 usec
TE 298.8 K
D1 2.00000000 sec
TD0 1

======== CHANNEL f1 ====
SFO1 161.9674942 MHz
NUC1 31P
P1 10.00 usec
PLW1 25.00000000 W

F2 − Processing parameters
SI 32768
SF 161.9755930 MHz
WDW EM
SSB 0
LB 1.00 Hz
GB 0
PC 1.40

^{1}H NMR ($CDCl_3$): **15a**

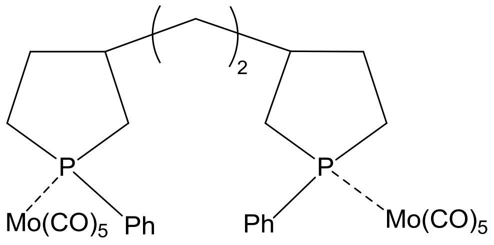

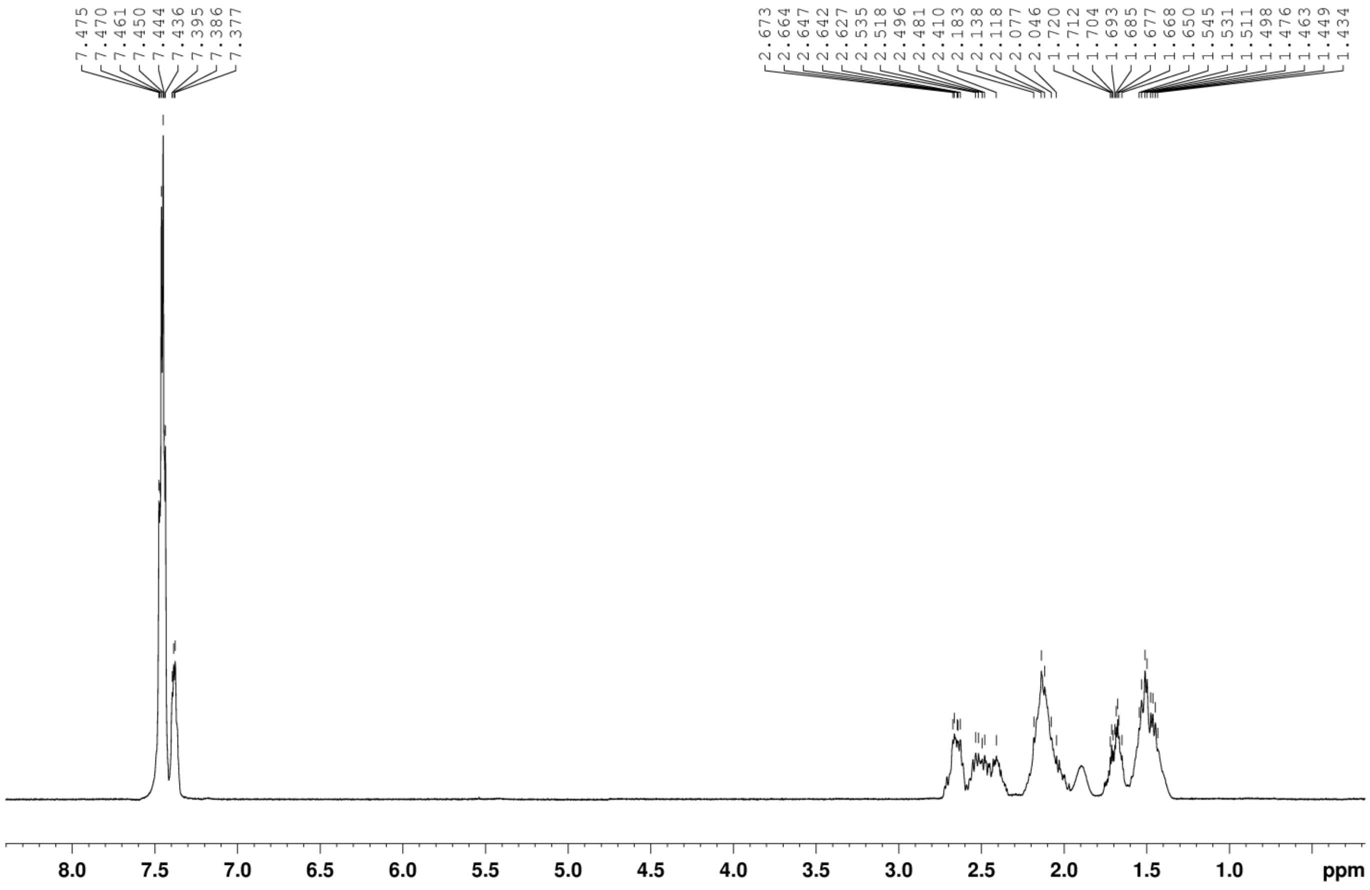

^{13}C NMR ($CDCl_3$): **15a**

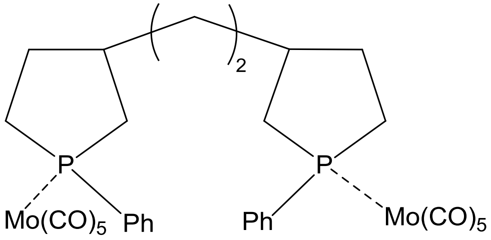

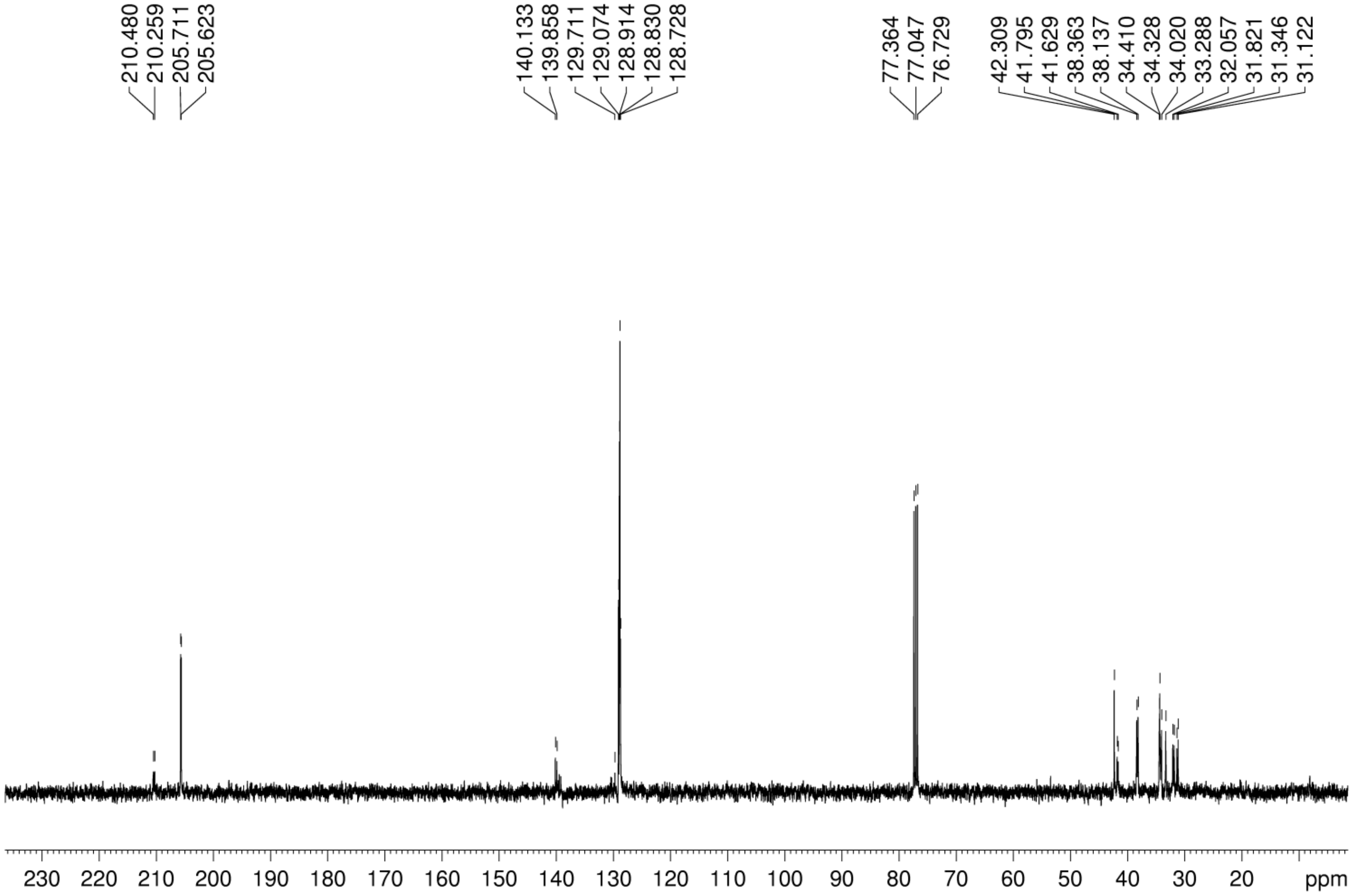

^{31}P NMR ($CDCl_3$): **15a**

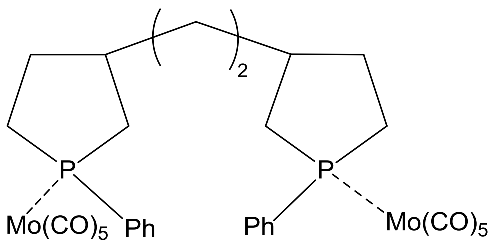

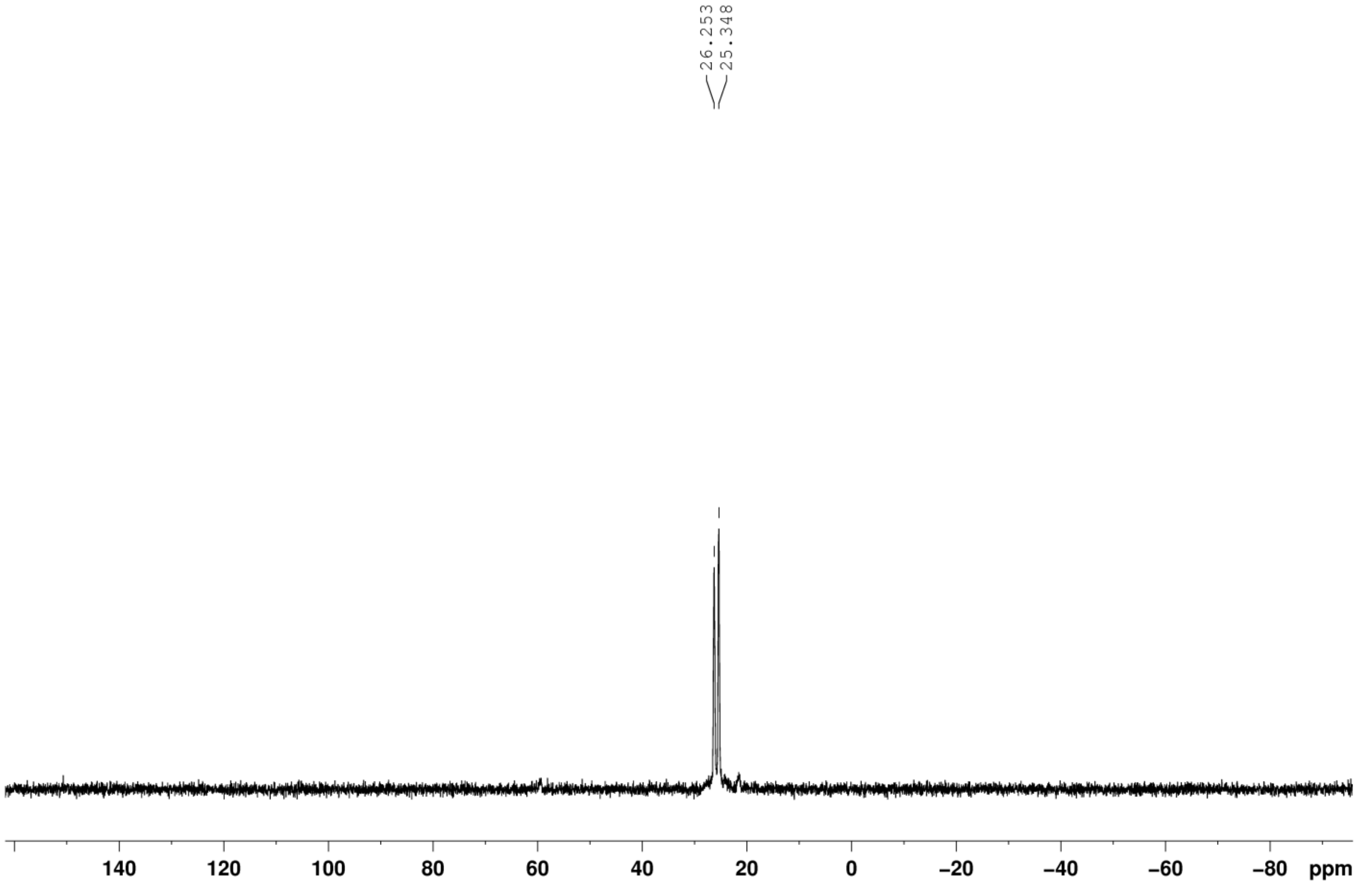

Supplement: File 2 — NMR spectra. [file Beilstein_J_Org_Chem-12-406-s002.pdf]
